# Supplementary material for: 8-Hydroxyquinoline Glycoconjugates: Modifications in the Linker Structure and Their Effect on the Cytotoxicity of the Obtained Compounds
Source: Molecules. 2019 Nov 18;24(22):4181. doi: 10.3390/molecules24224181 (PMC6891455; doi:10.3390/molecules24224181)
Supplement: Supplementary file 1 [file molecules-24-04181-s001.pdf]

Supplementary Materials

8-Hydroxyquinoline glycoconjugates: modifications in the linker structure and their effect on the cytotoxicity of the obtained compounds

Monika Krawczyk [1,2,*], Gabriela Pastuch-Gawołek [1,2], Aleksandra Pluta [1], Karol Erfurt [3], Adrian Domiński [4] and Piotr Kurcok [4]

[1] Department of Organic Chemistry, Bioorganic Chemistry and Biotechnology, Silesian University of Technology, B. Krzywoustego 4, 44-100 Gliwice, Poland; gabriela.pastuch@polsl.pl (G.P.-G.); aleksandrapluta93@o2.pl (A.P.)

[2] Biotechnology Centre, Silesian University of Technology, B. Krzywoustego 8, 44-100 Gliwice, Poland

[3] Department of Chemical Organic Technology and Petrochemistry, Silesian University of Technology, B. Krzywoustego 4, 44-100 Gliwice, Poland; karol.erfurt@polsl.pl (K.E.)

[4] Centre of Polymer and Carbon Materials, Polish Academy of Sciences, M. Curie-Sklodowskiej 34, 41-819 Zabrze, Poland; adrian.dominski@cmpw-pan.edu.pl (A.D.); piotr.kurcok@cmpw-pan.edu.pl (P.K.)

* Correspondence: monika.krawczyk@polsl.pl (M.K.); Tel.: +48-32-237-1759

1. Spectra

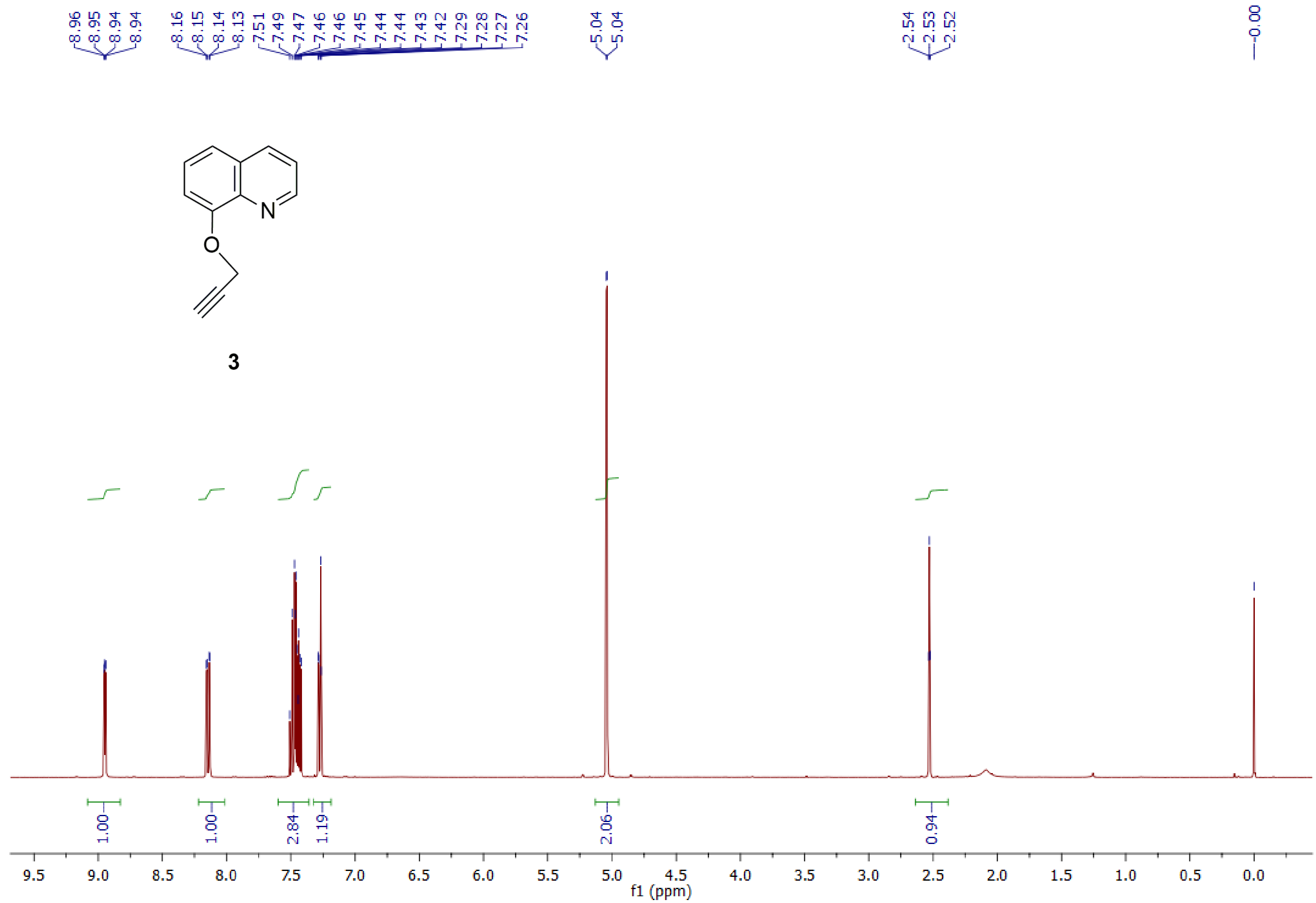

Fig. S1: ^{1}H NMR spectrum of compound **3**.

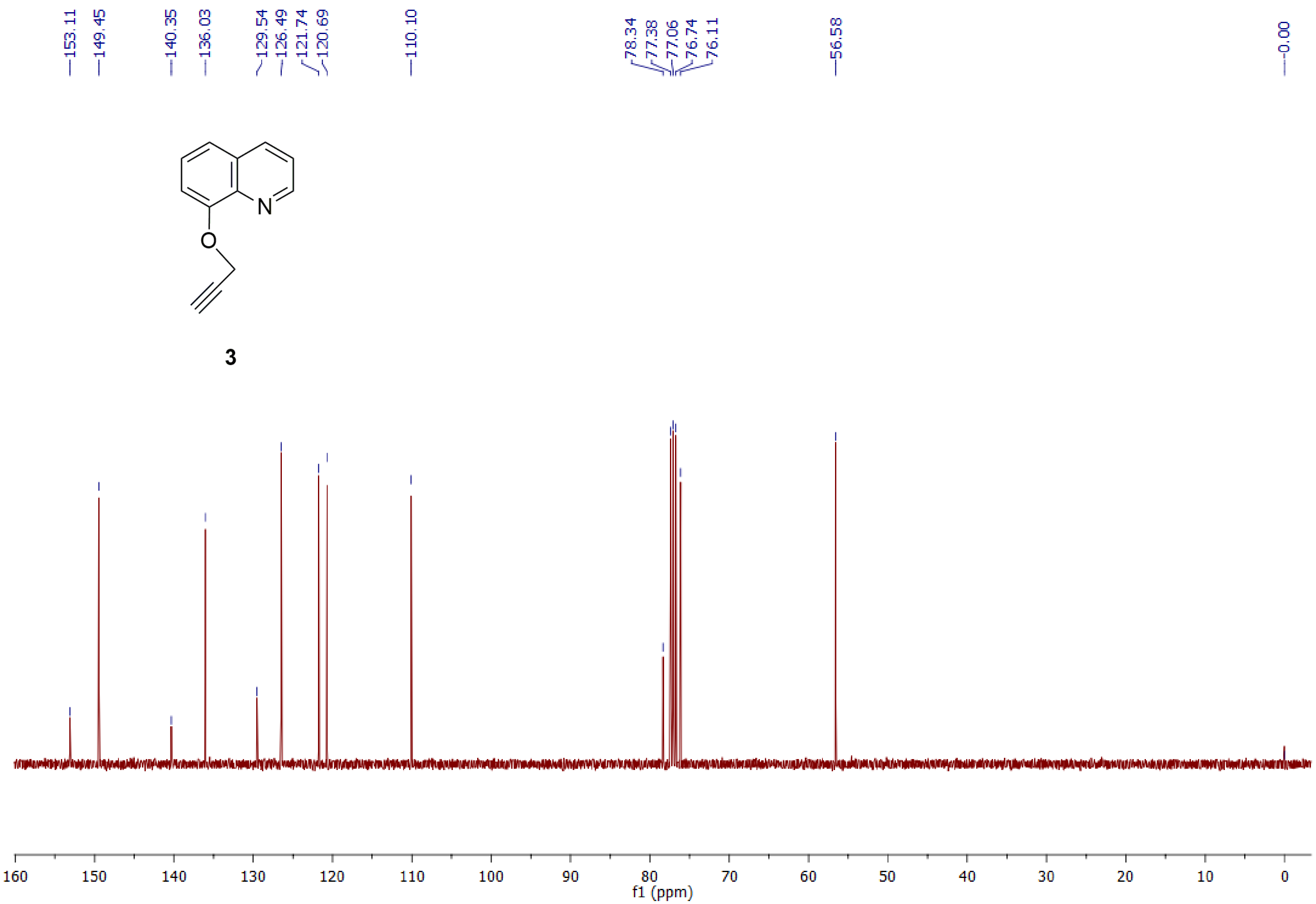

Fig. S2: ^{13}C NMR spectrum of compound **3**.

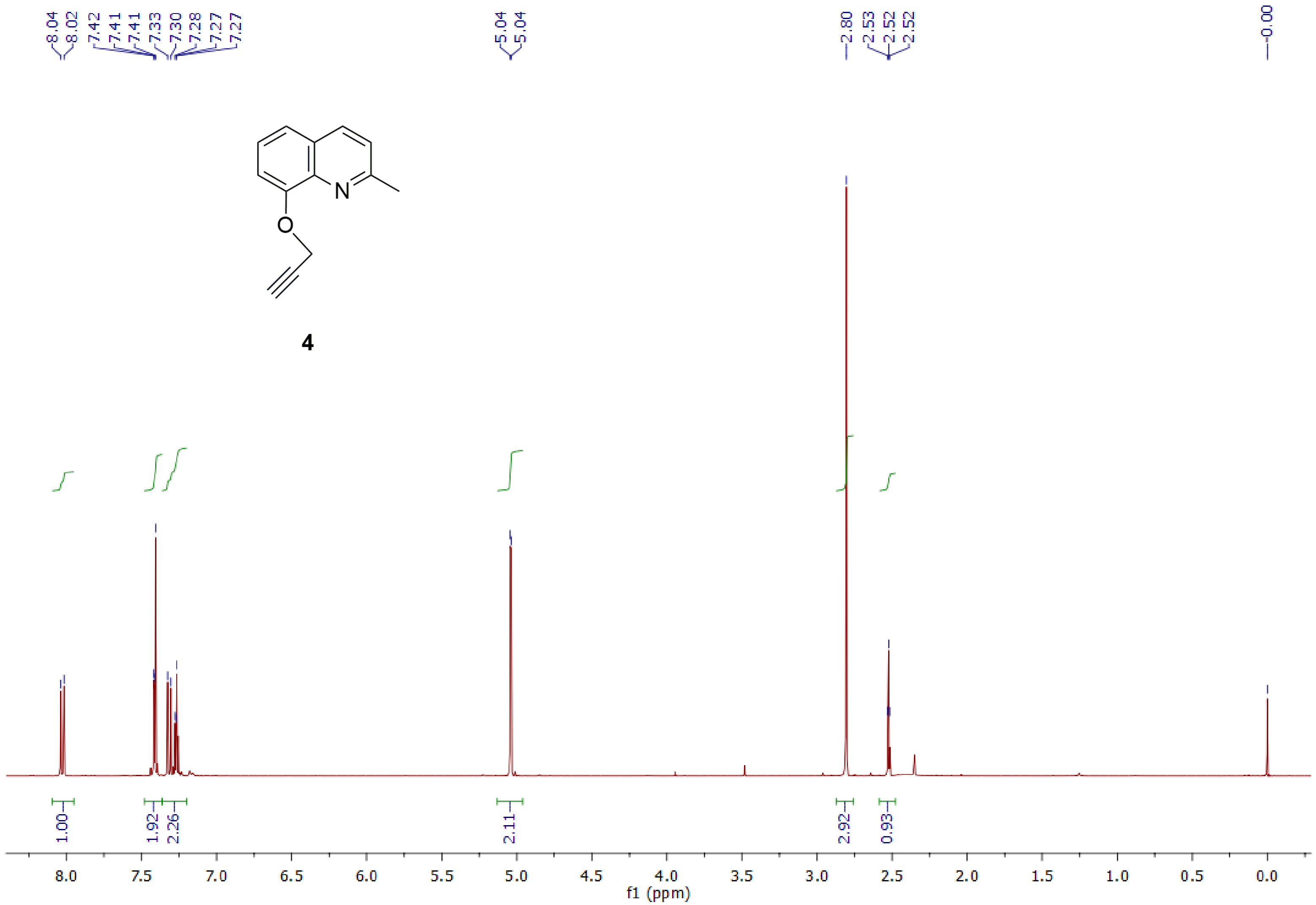

Fig. S3: ^{1}H NMR spectrum of compound **4**.

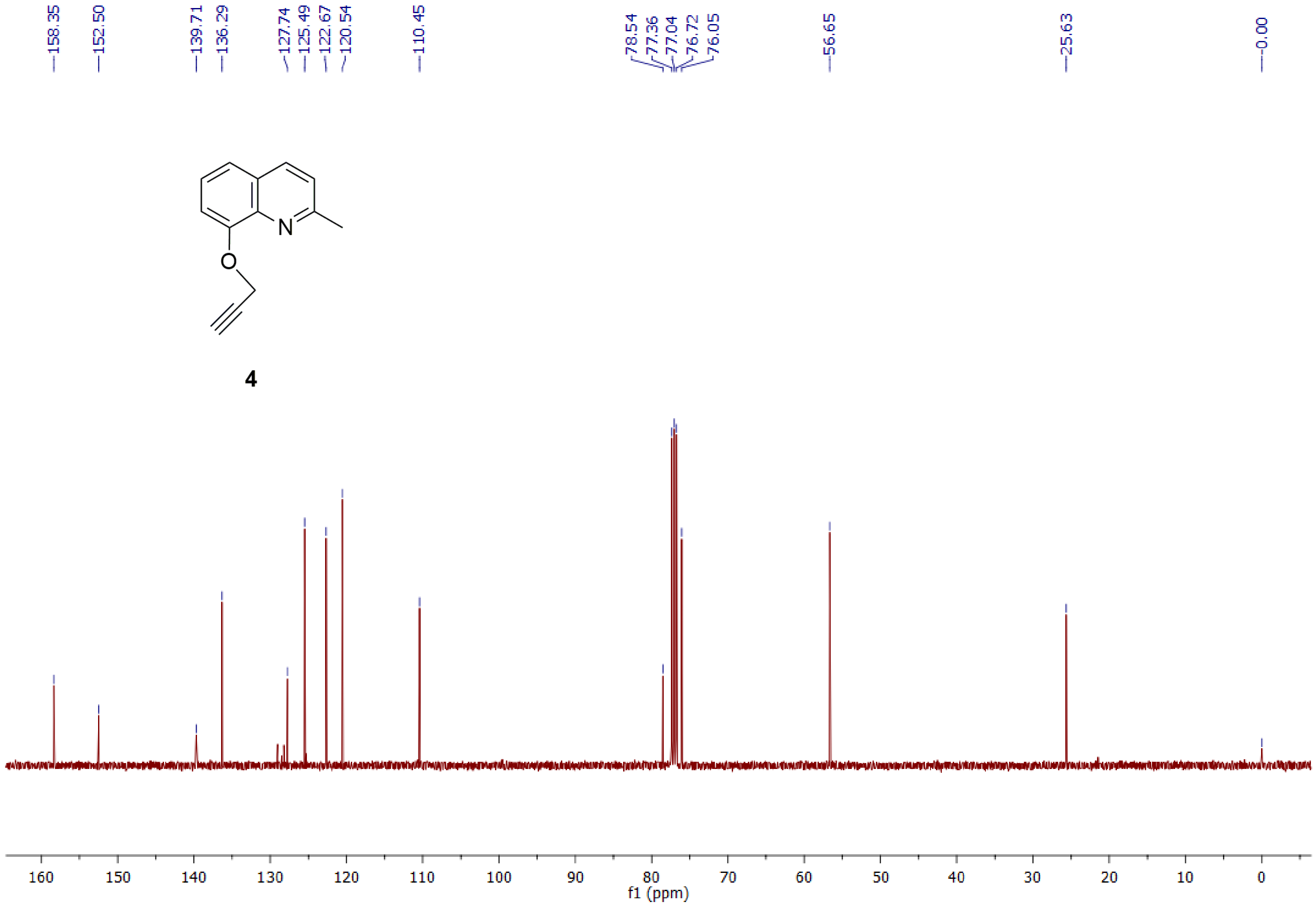

Fig. S4: ^{13}C NMR spectrum of compound **4**.

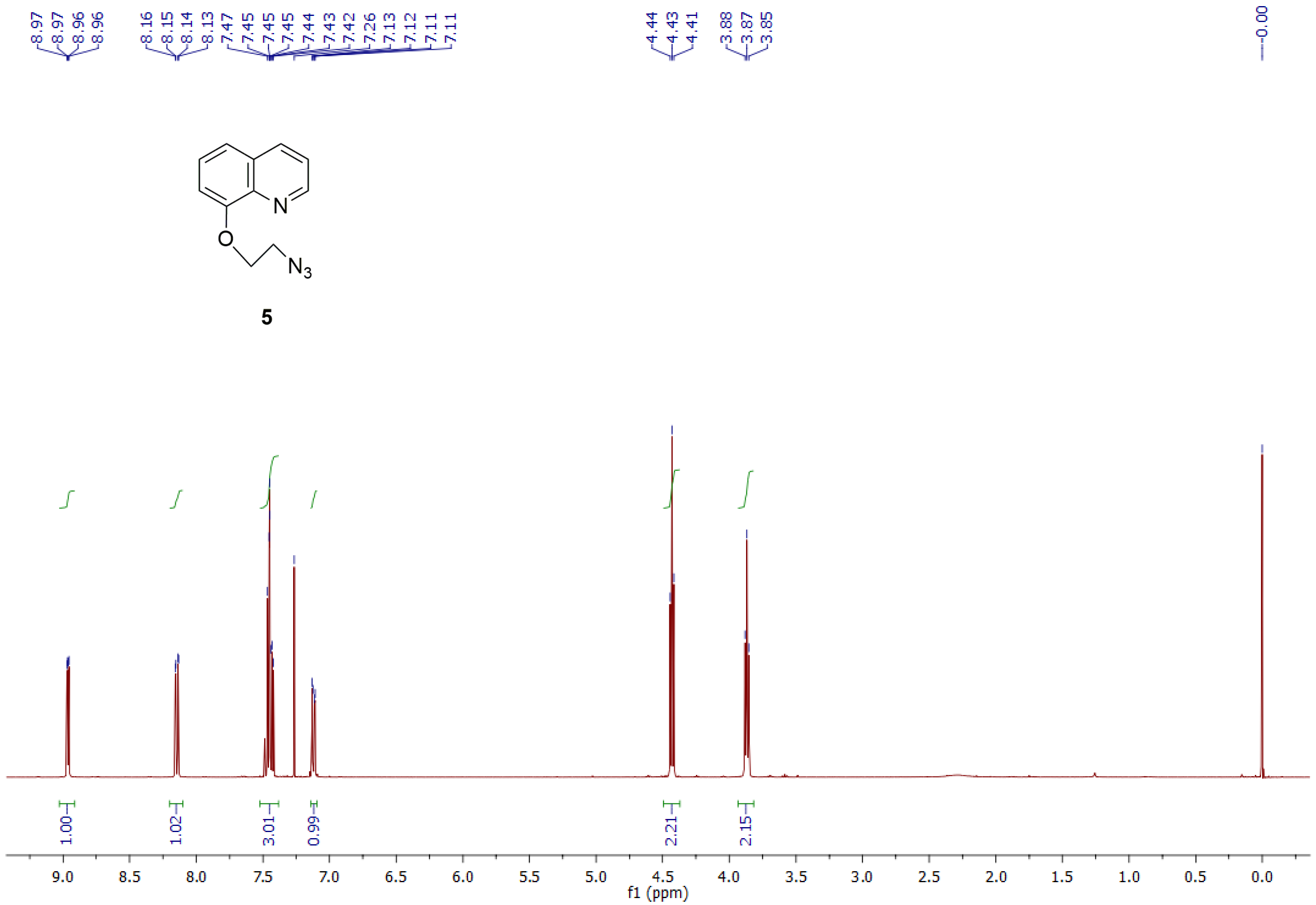

Fig. S5: ^{1}H NMR spectrum of compound **5**.

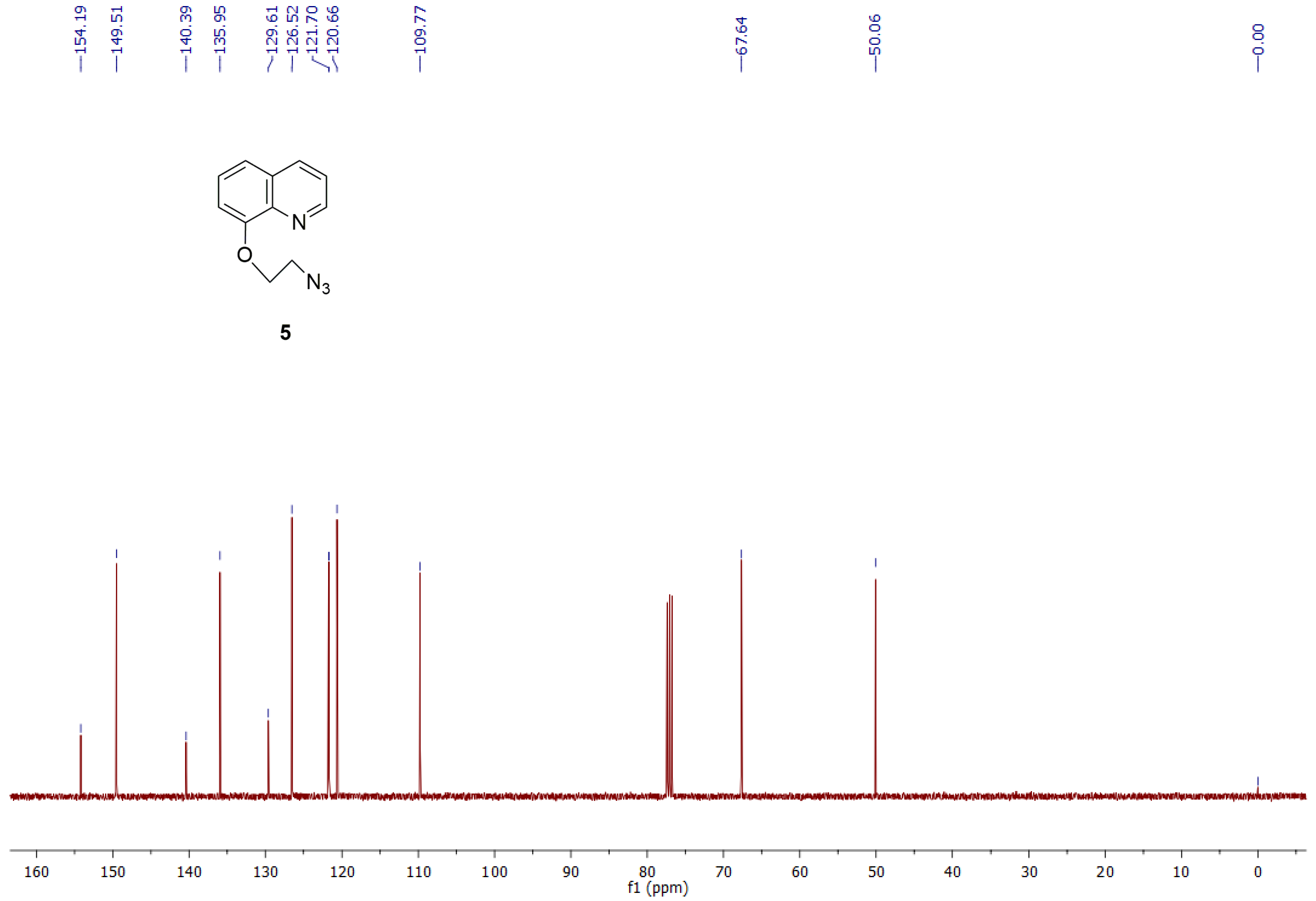

Fig. S6: ^{13}C NMR spectrum of compound **5**.

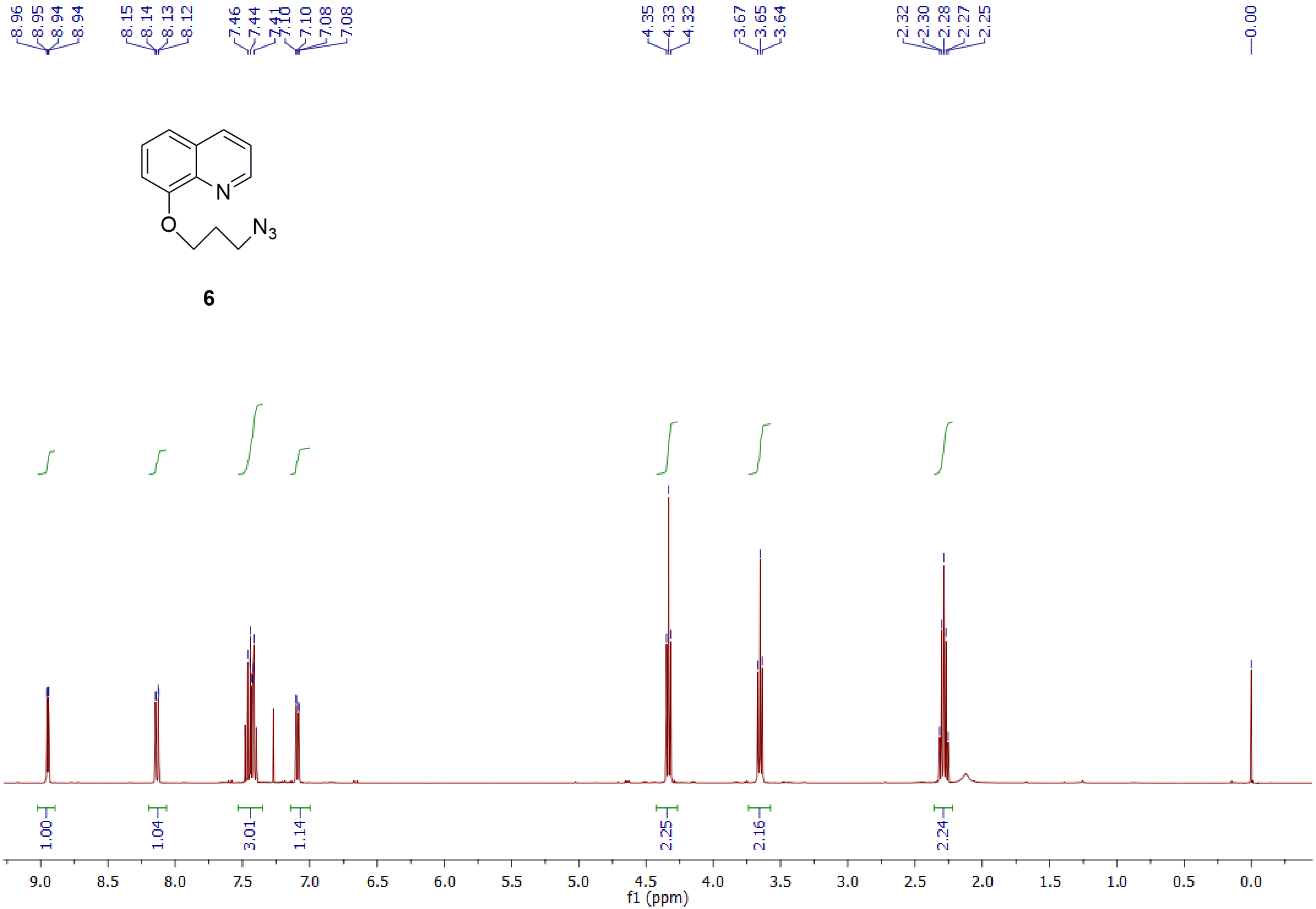

Fig. S7: ^{1}H NMR spectrum of compound **6**.

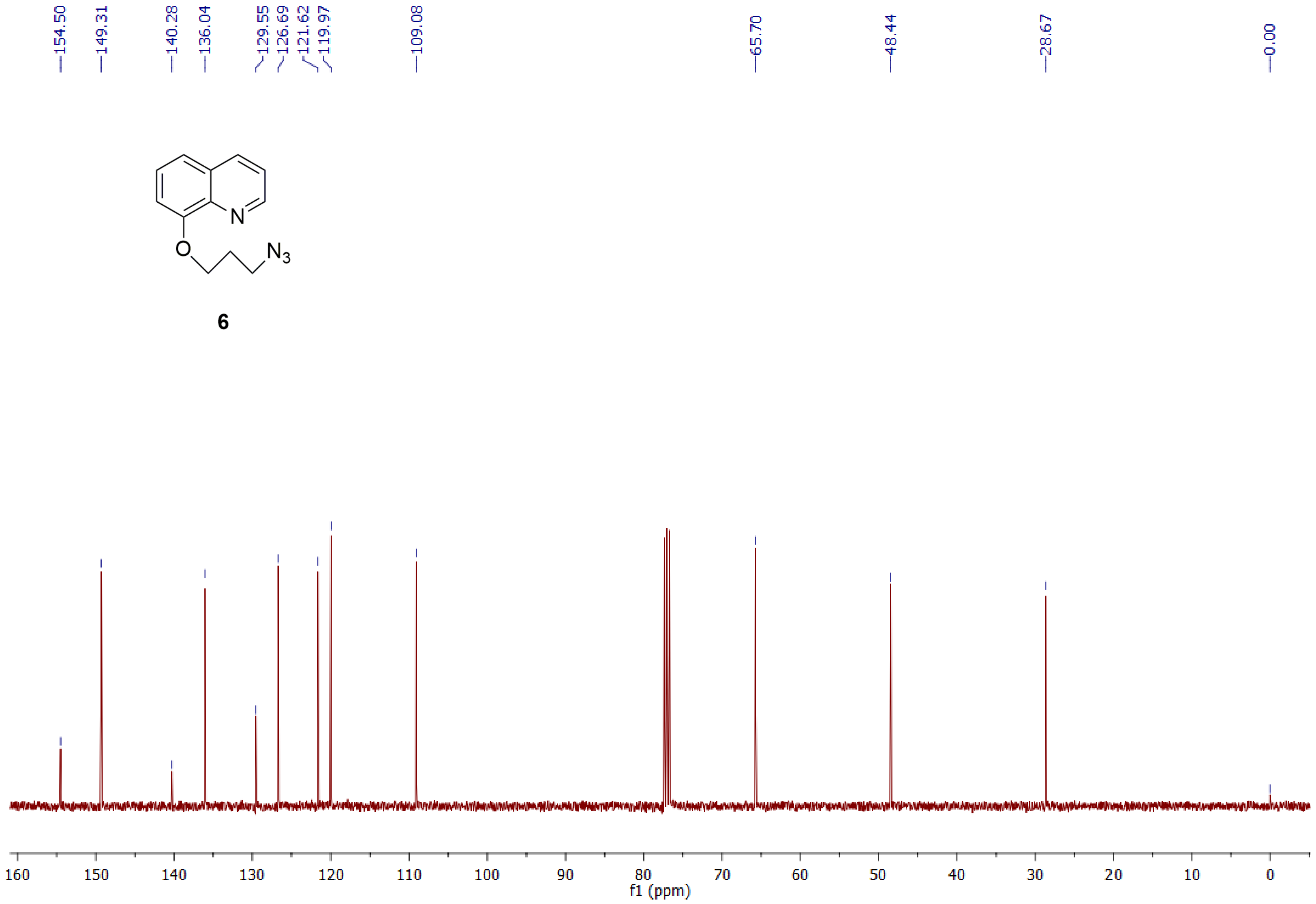

Fig. S8: ^{13}C NMR spectrum of compound **6**.

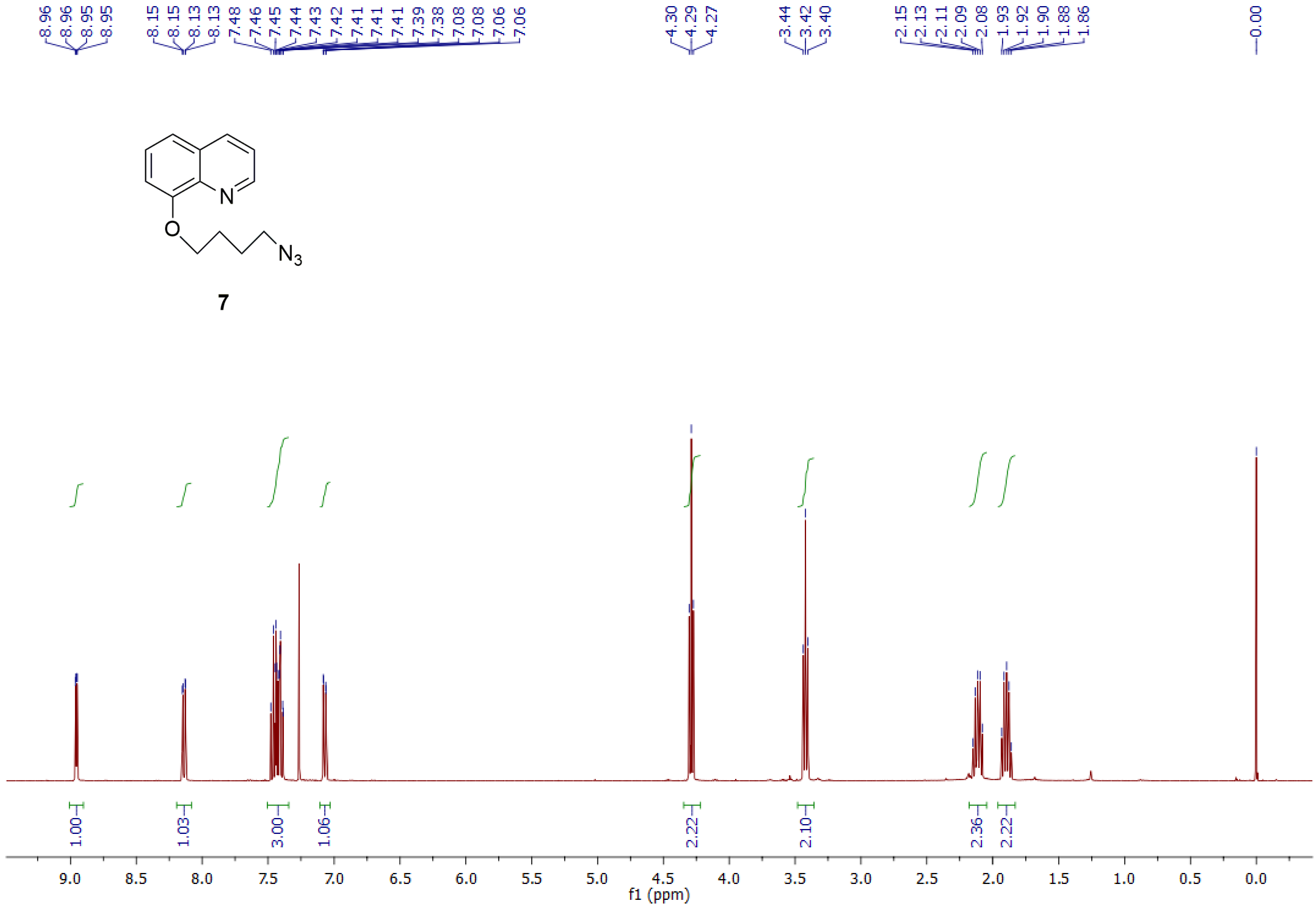

Fig. S9: ^{1}H NMR spectrum of compound **7**.

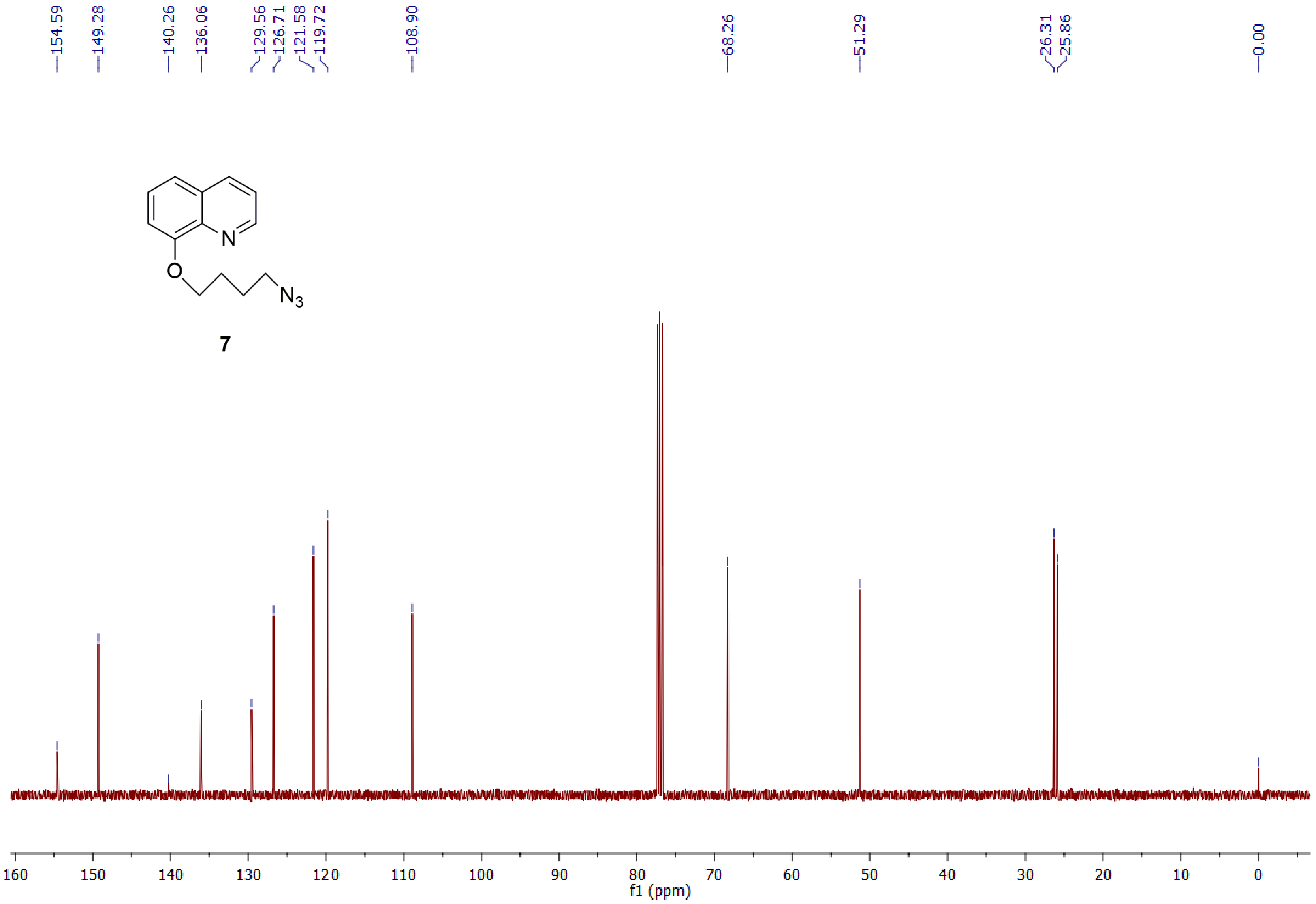

Fig. S10: ^{13}C NMR spectrum of compound **7**.

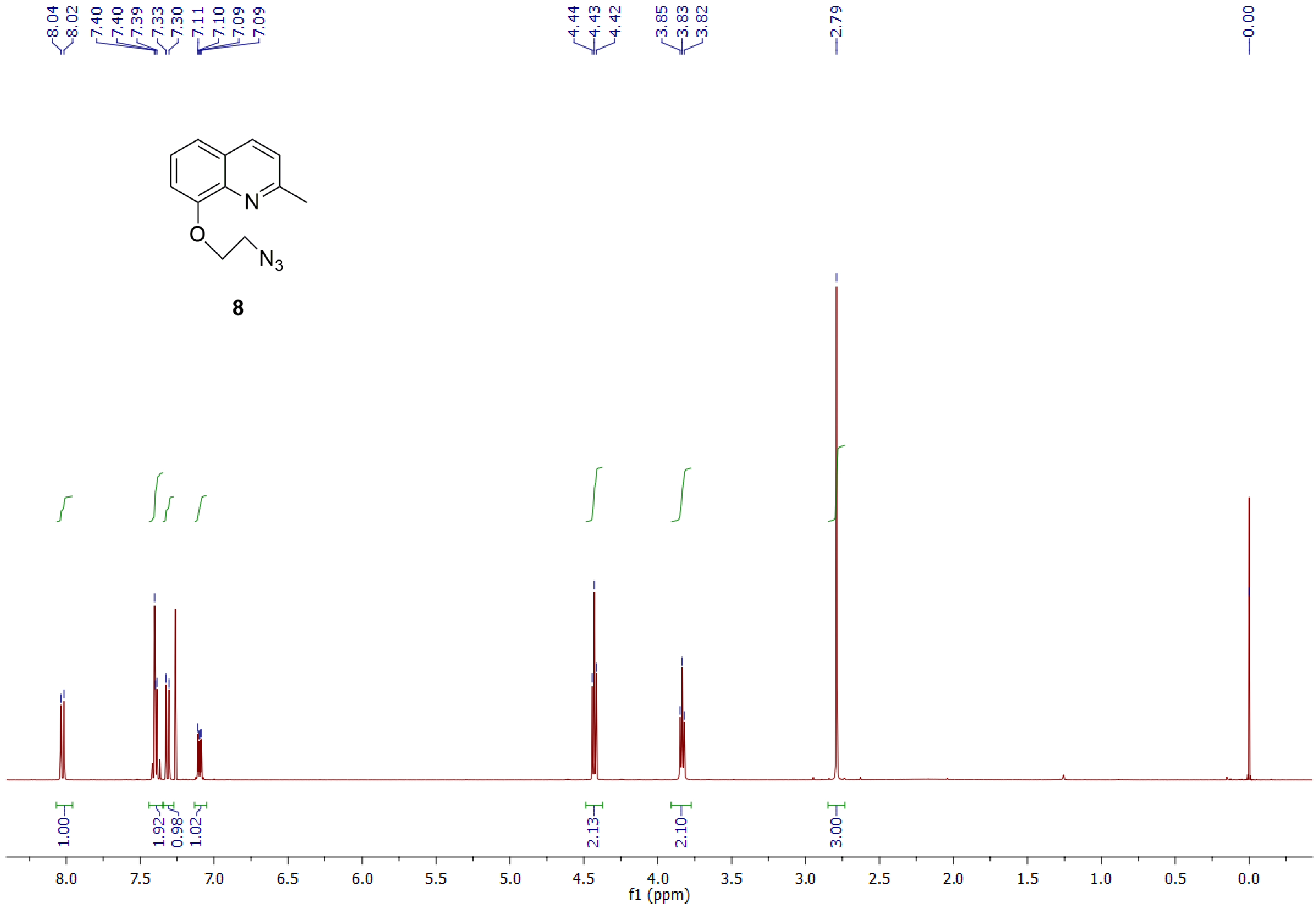

Fig. S11: ^{1}H NMR spectrum of compound **8**.

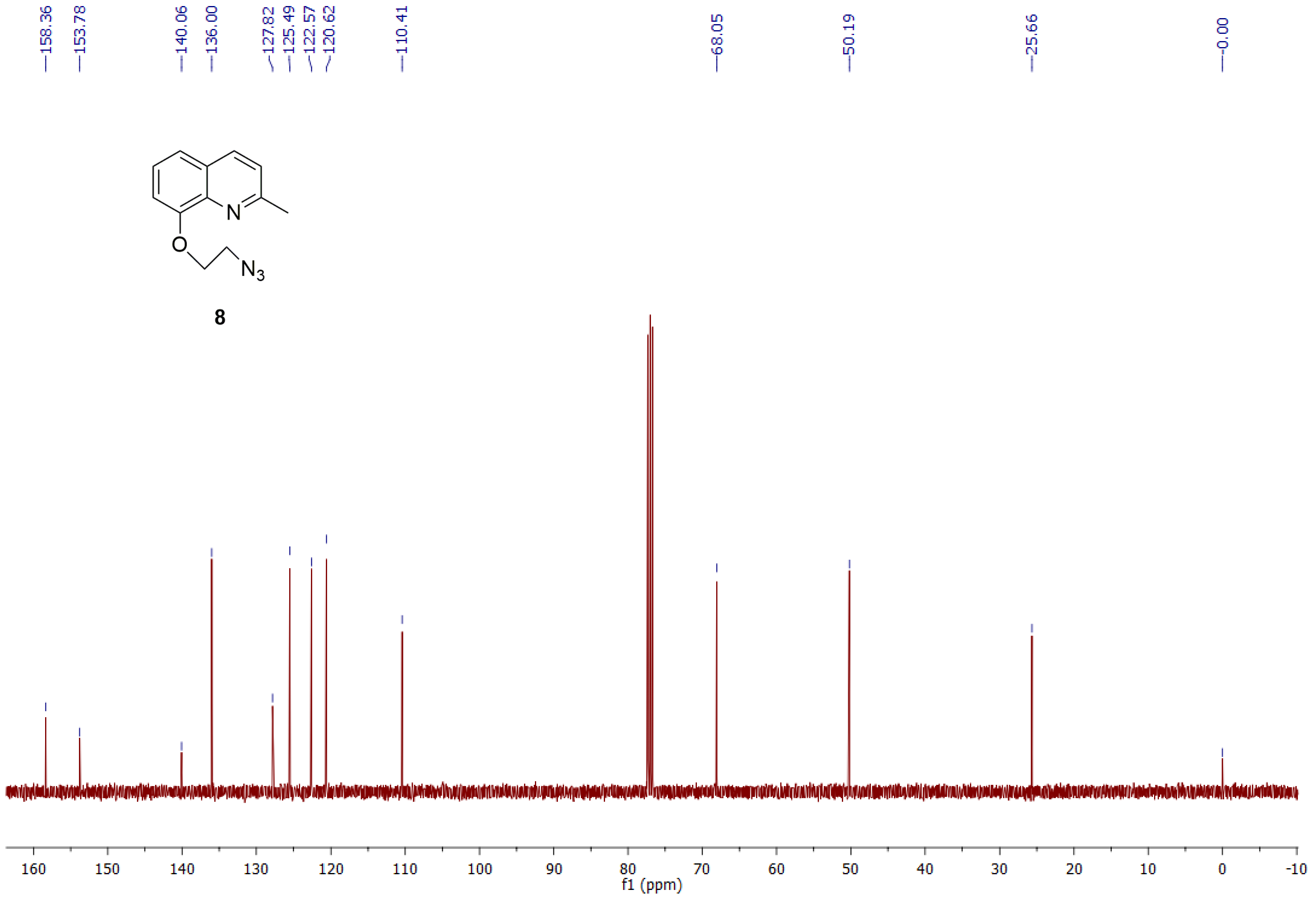

Fig. S12: ^{13}C NMR spectrum of compound **8**.

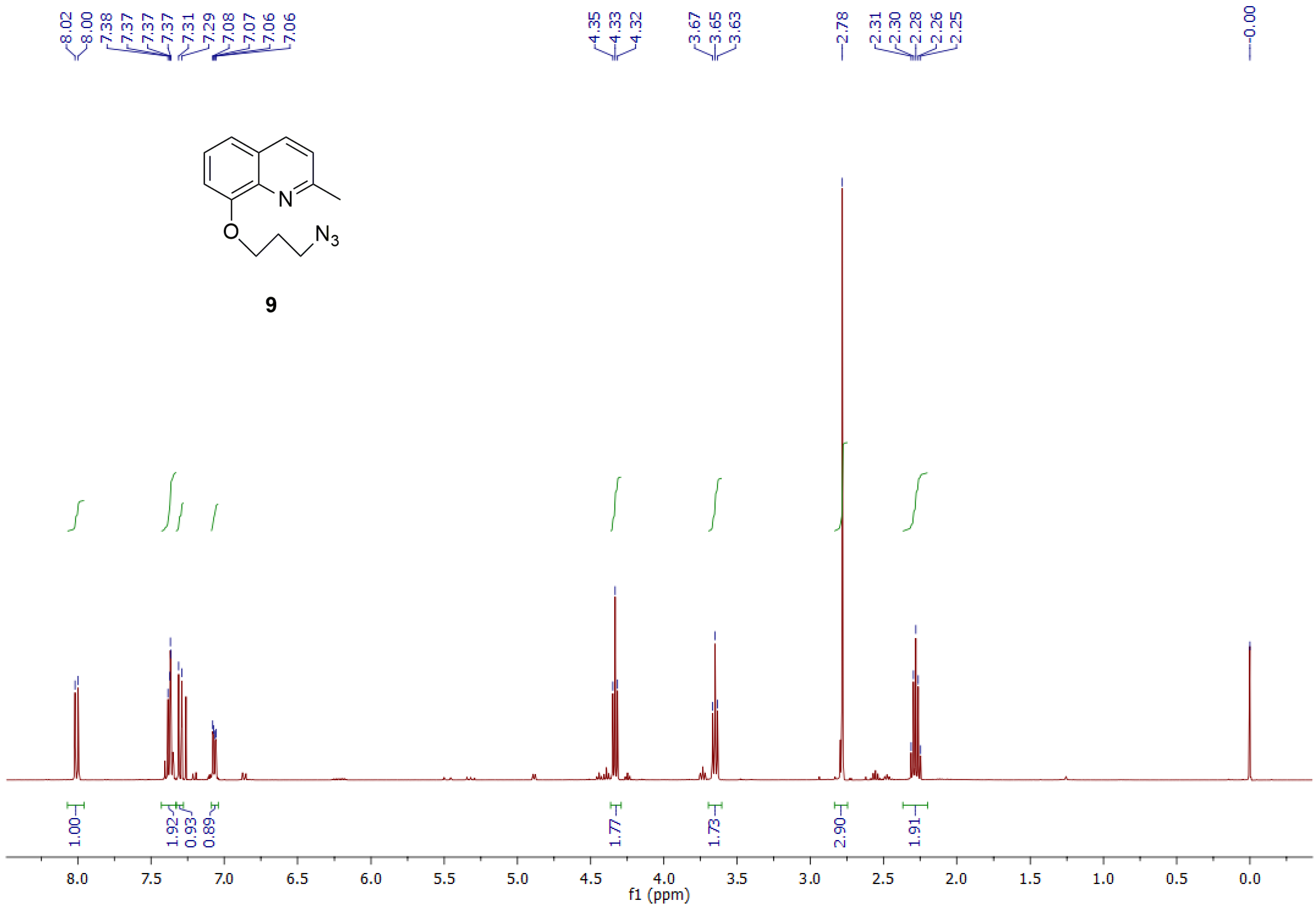

Fig. S13: ^{1}H NMR spectrum of compound **9**.

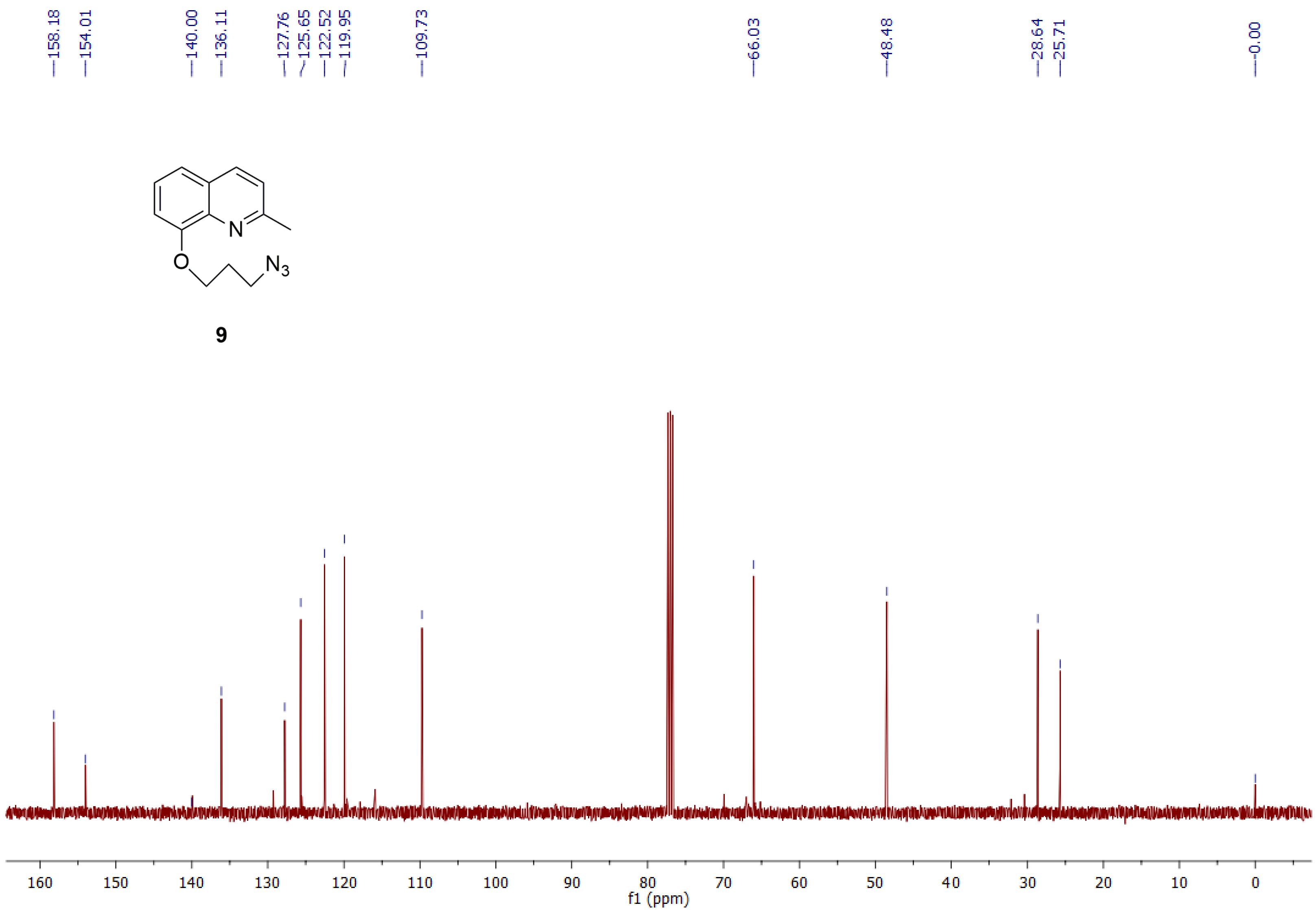

Fig. S14: ^{13}C NMR spectrum of compound **9**.

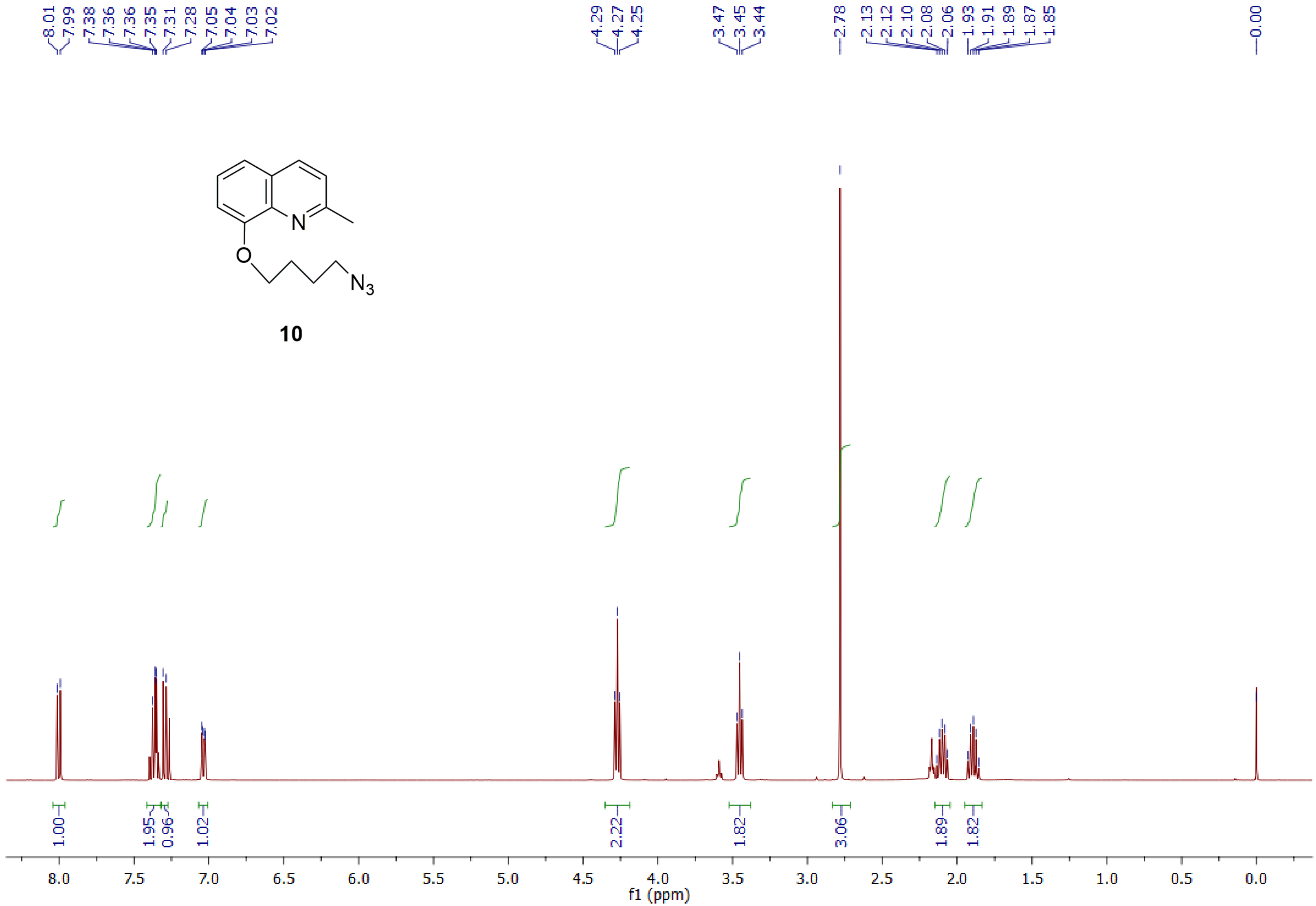

Fig. S15: ^{1}H NMR spectrum of compound **10**.

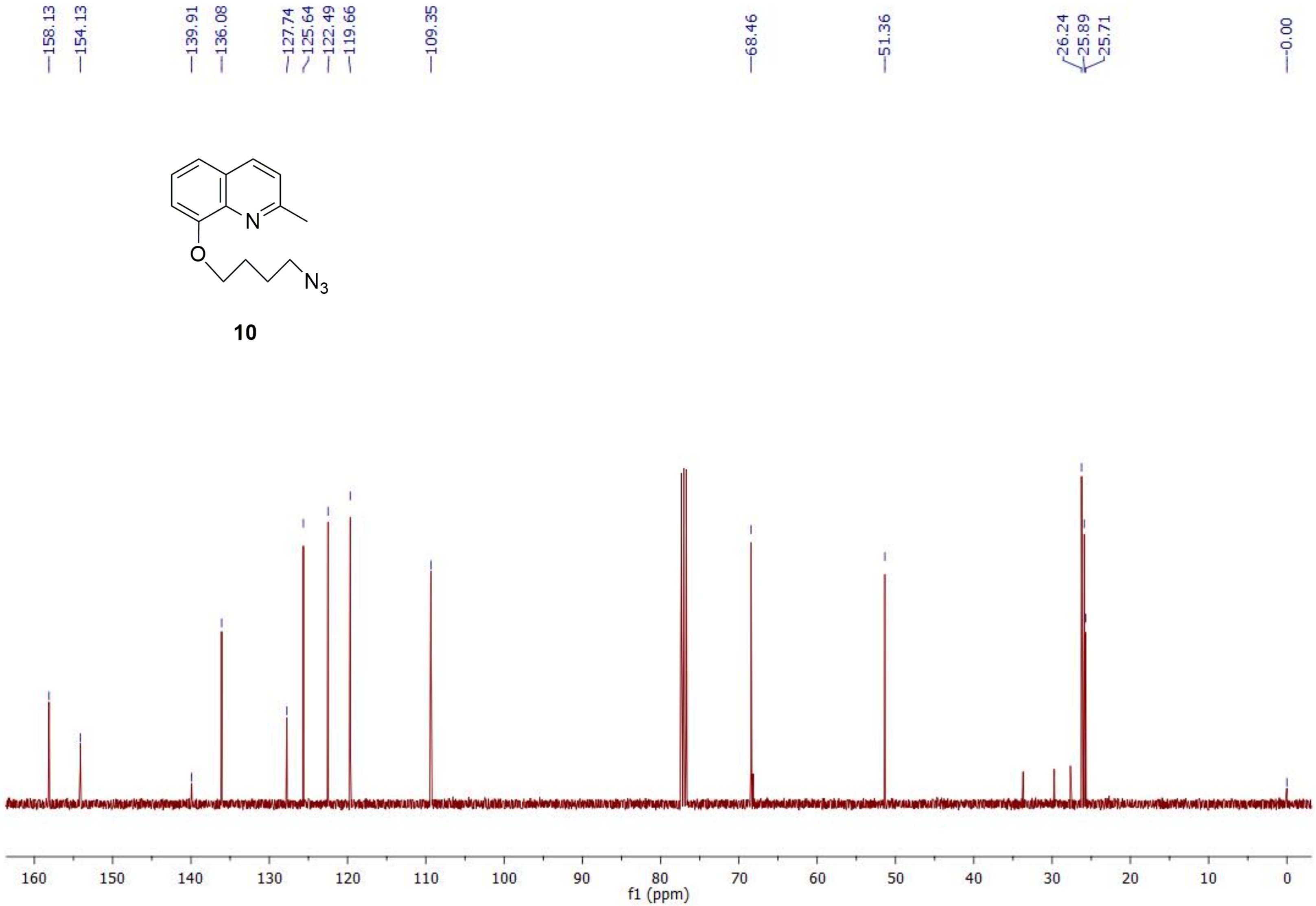

Fig. S16: ^{13}C NMR spectrum of compound **10**.

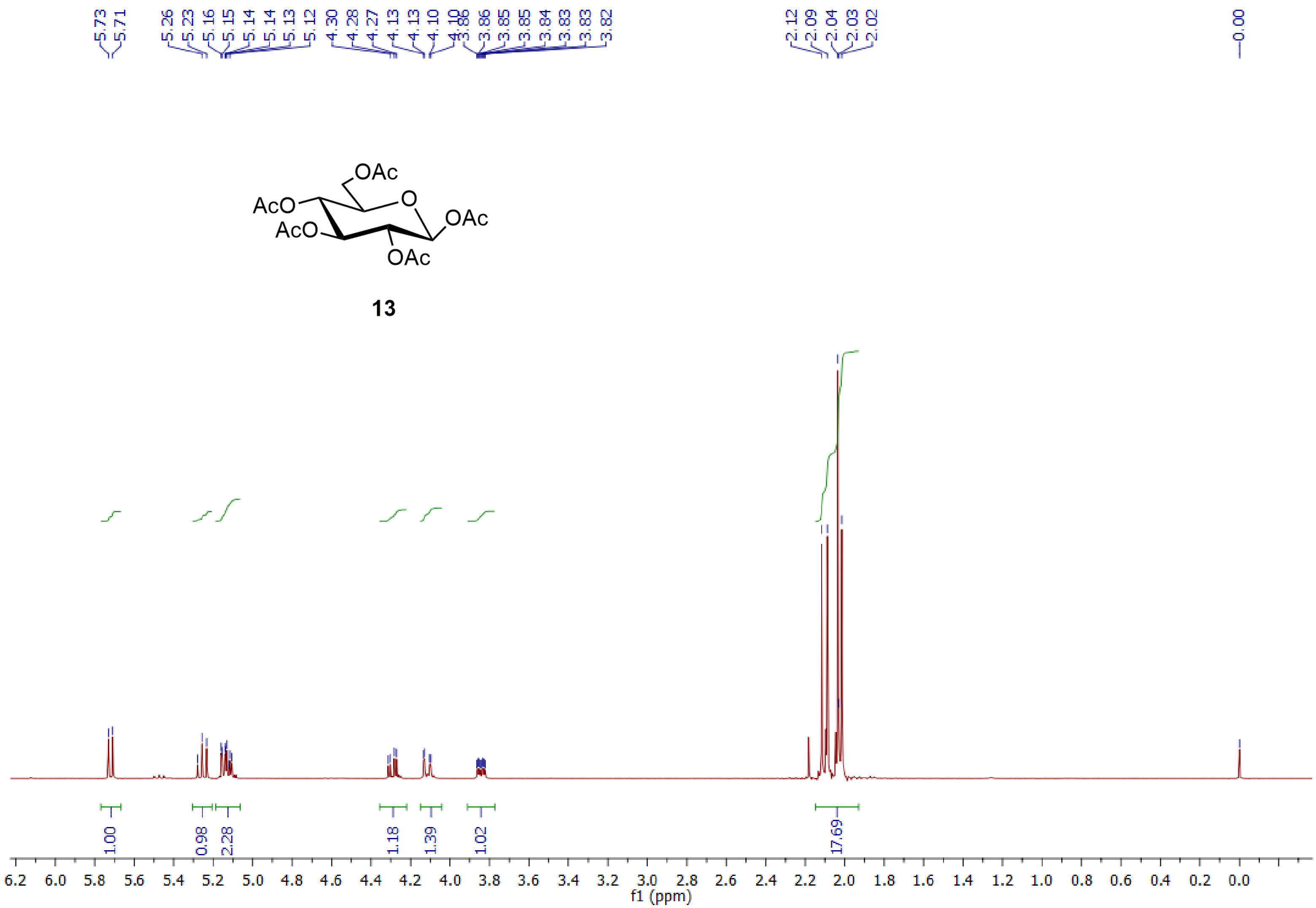

Fig. S17: ^{1}H NMR spectrum of compound **13**.

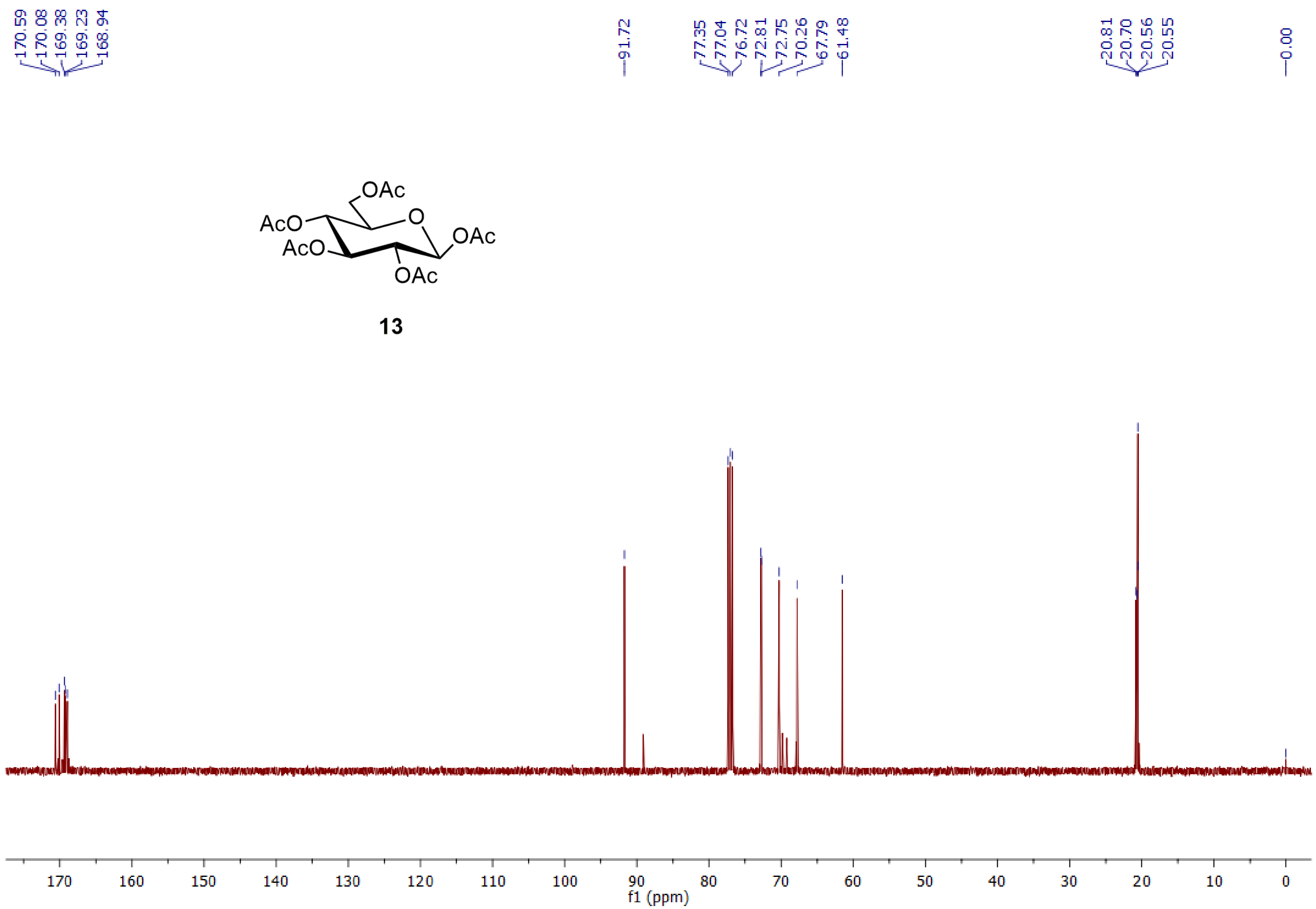

13

Fig. S18: ^{13}C NMR spectrum of compound **13**.

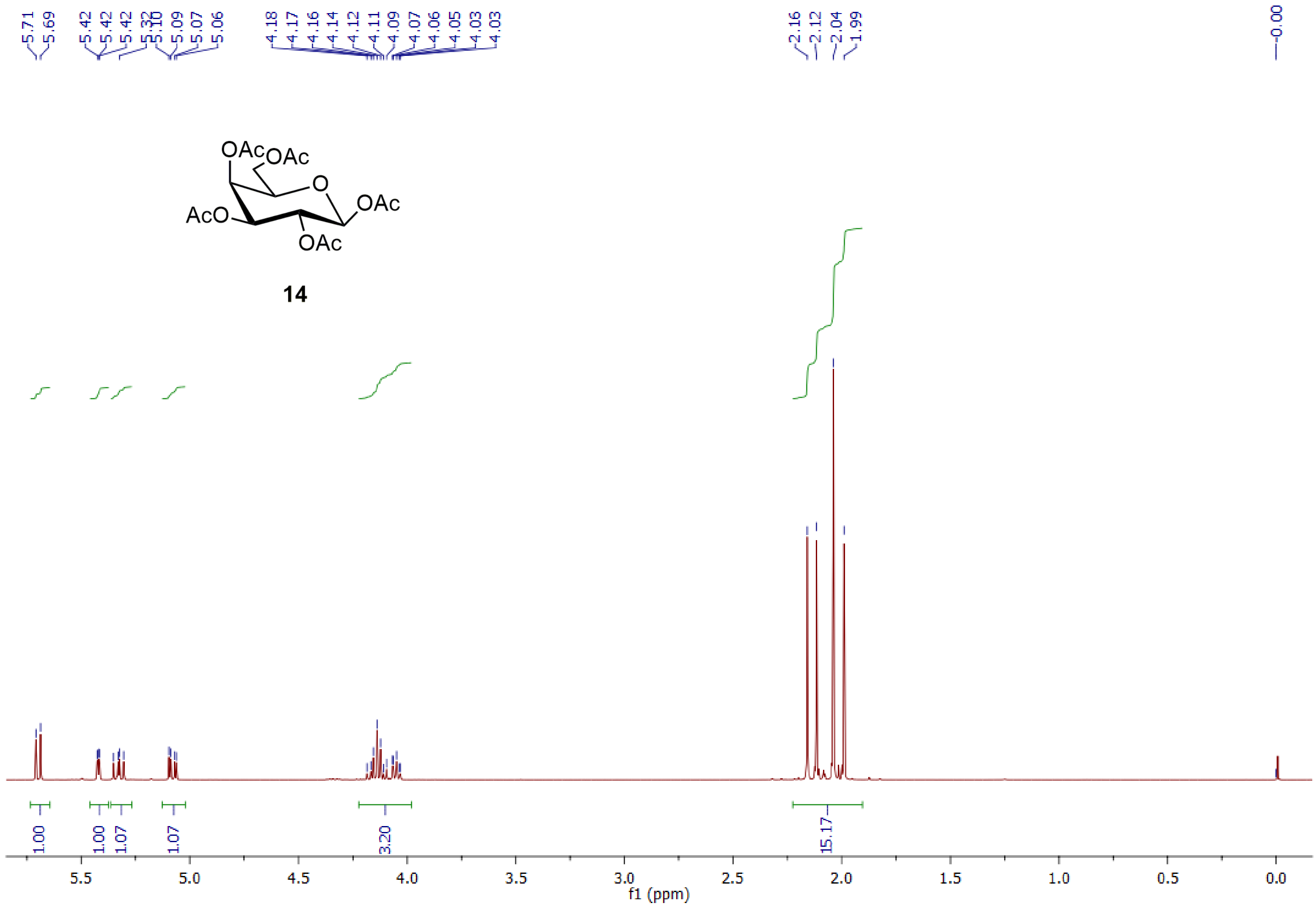

Fig. S19: ^{1}H NMR spectrum of compound **14**.

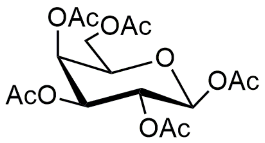

14

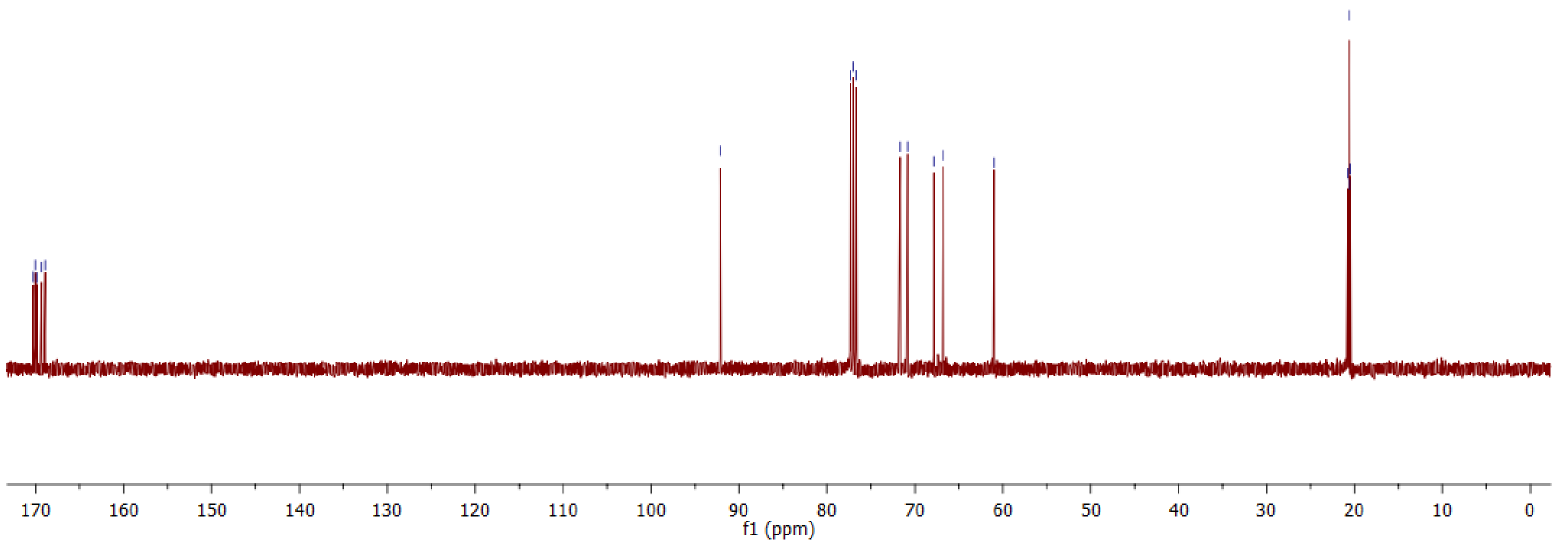

Fig. S20: ^{13}C NMR spectrum of compound **14**.

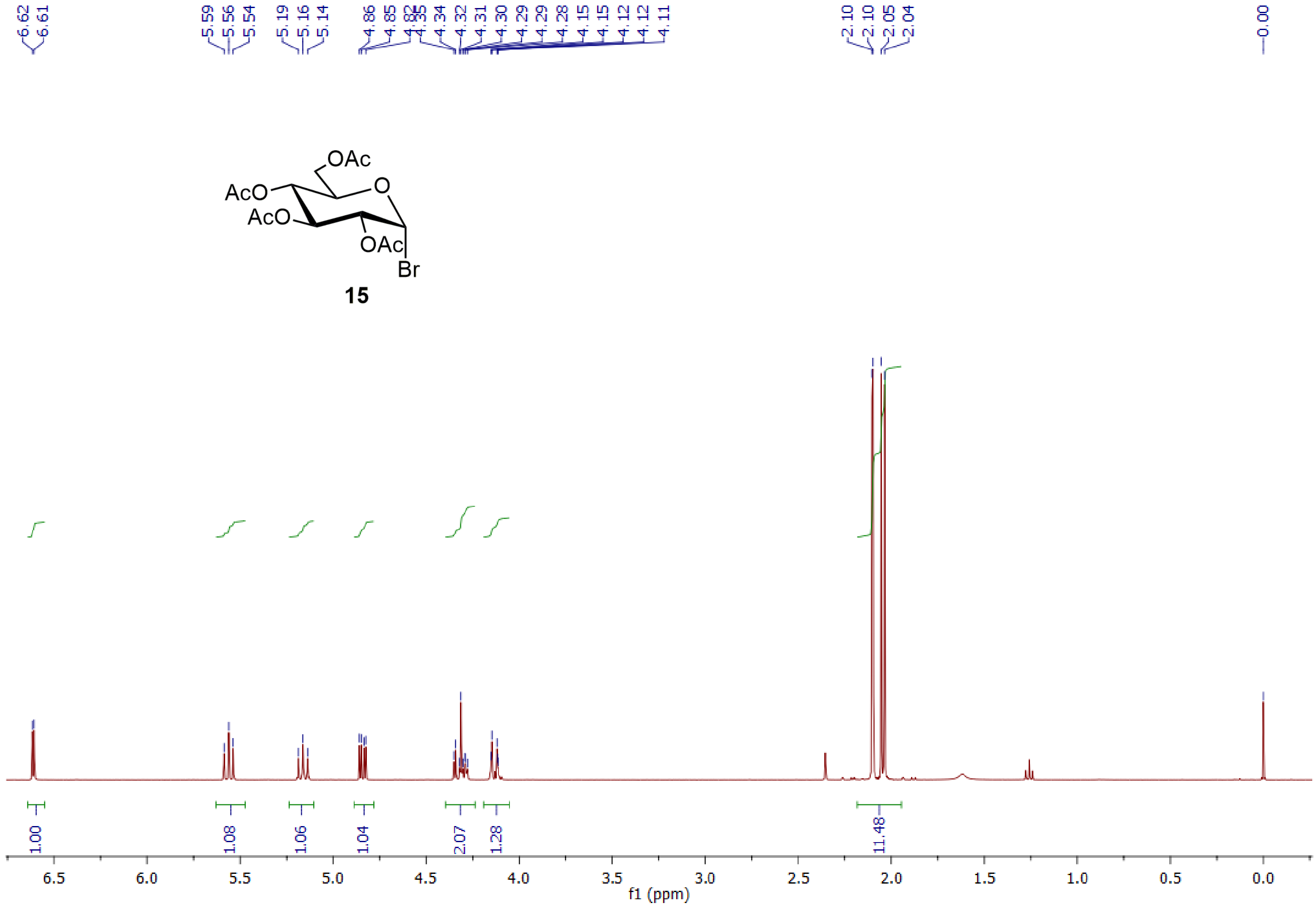

Fig. S21: ^{1}H NMR spectrum of compound **15**.

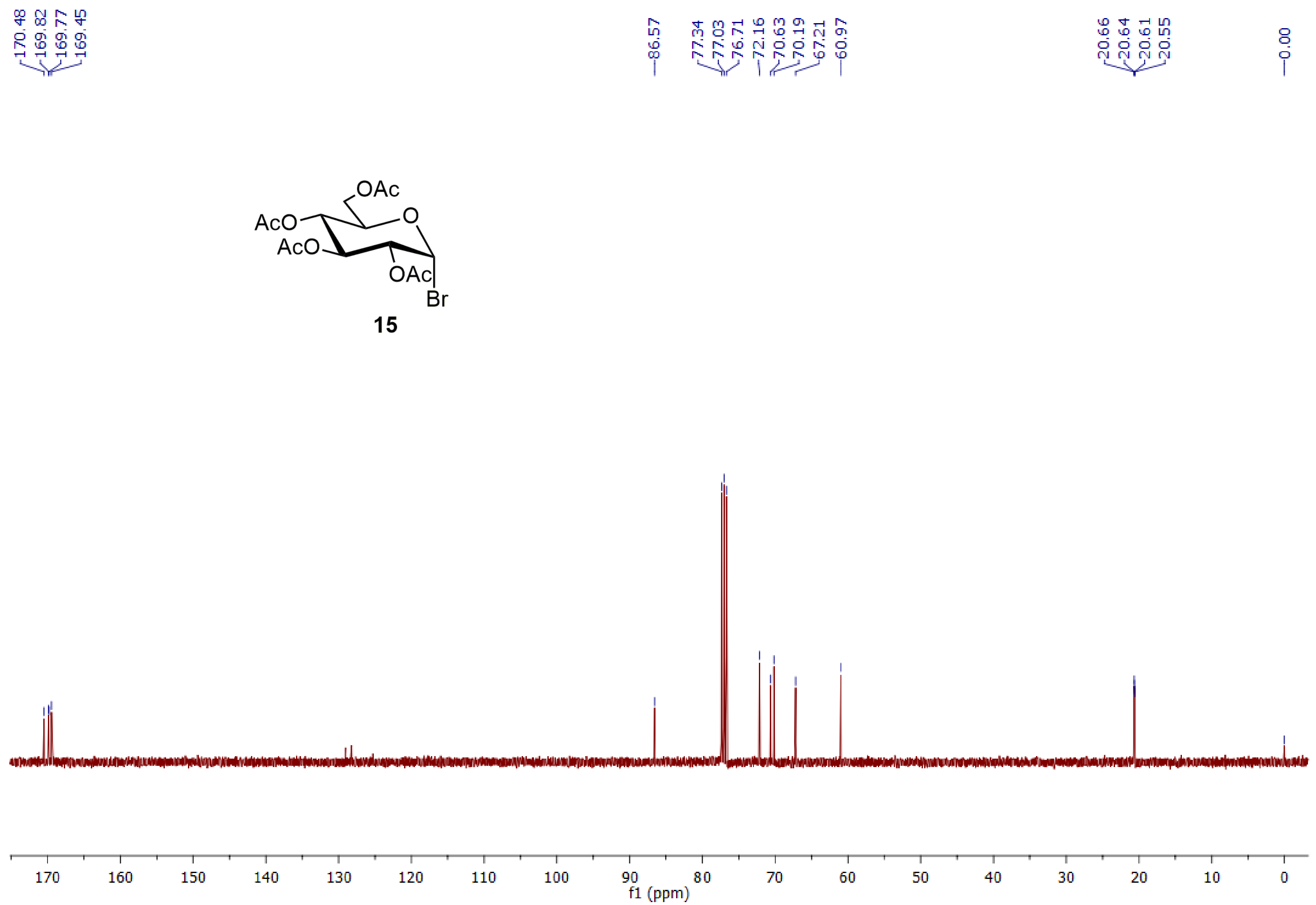

Fig. S22: ^{13}C NMR spectrum of compound **15**.

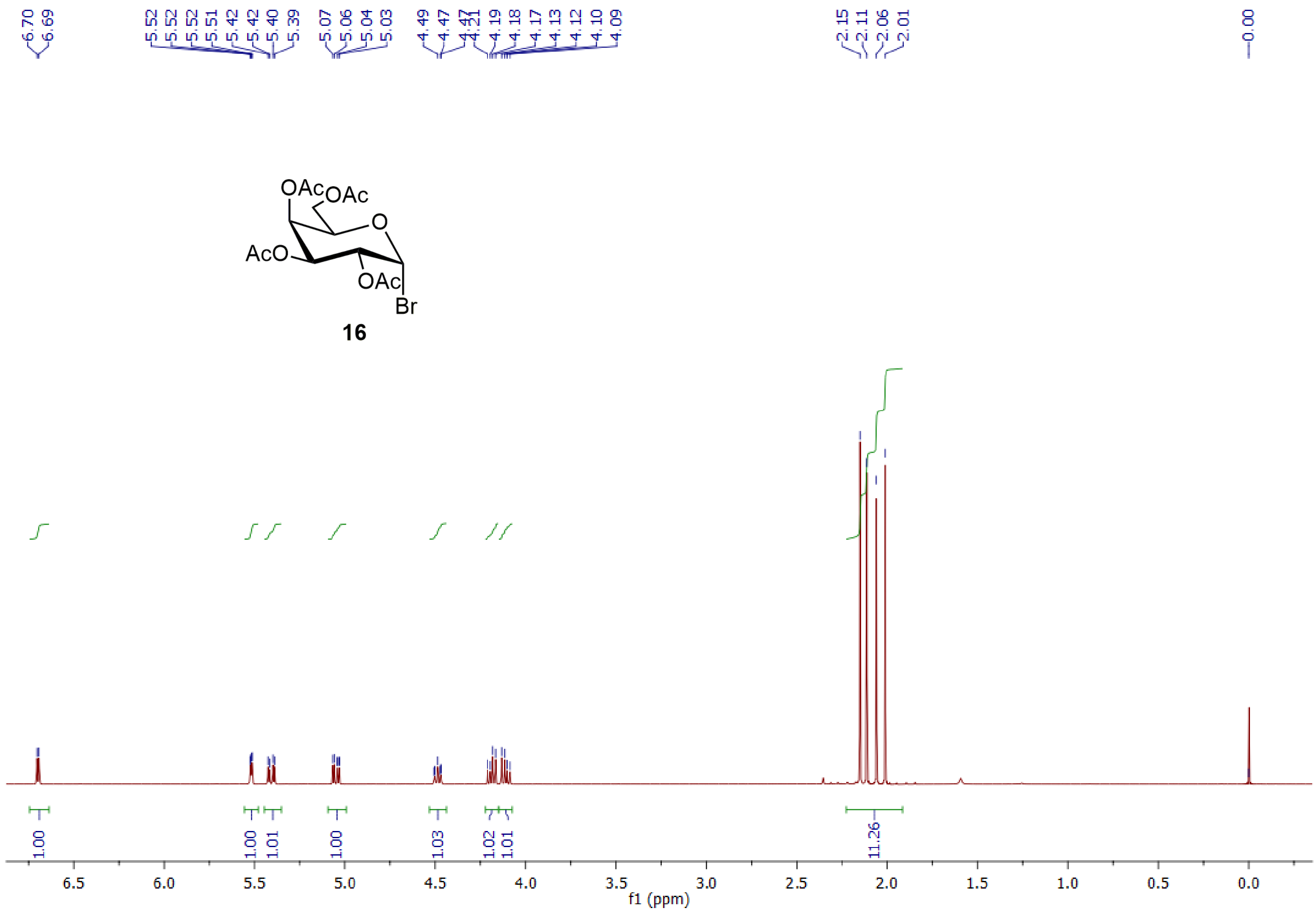

Fig. S23: ^{1}H NMR spectrum of compound **16**.

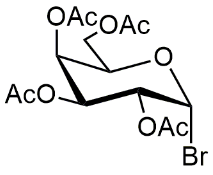

16

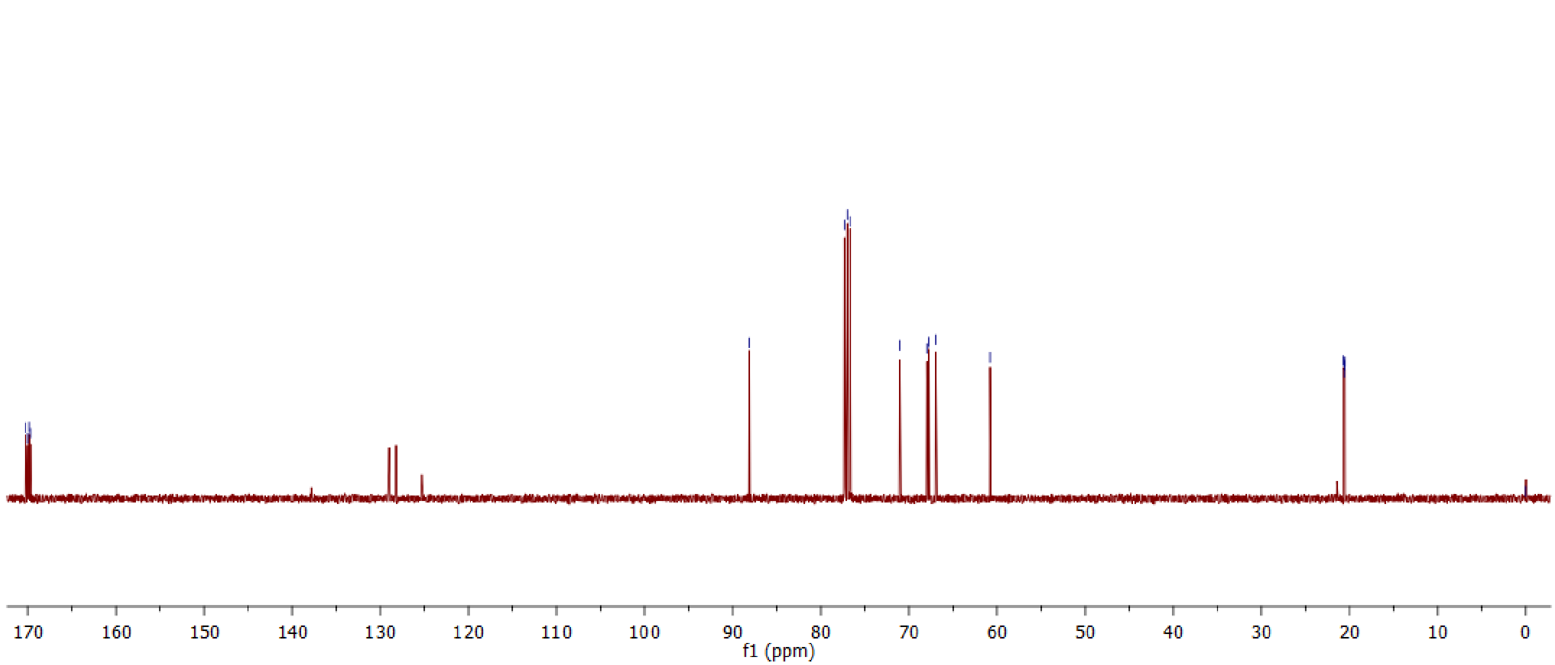

Fig. S24: ^{13}C NMR spectrum of compound **16**.

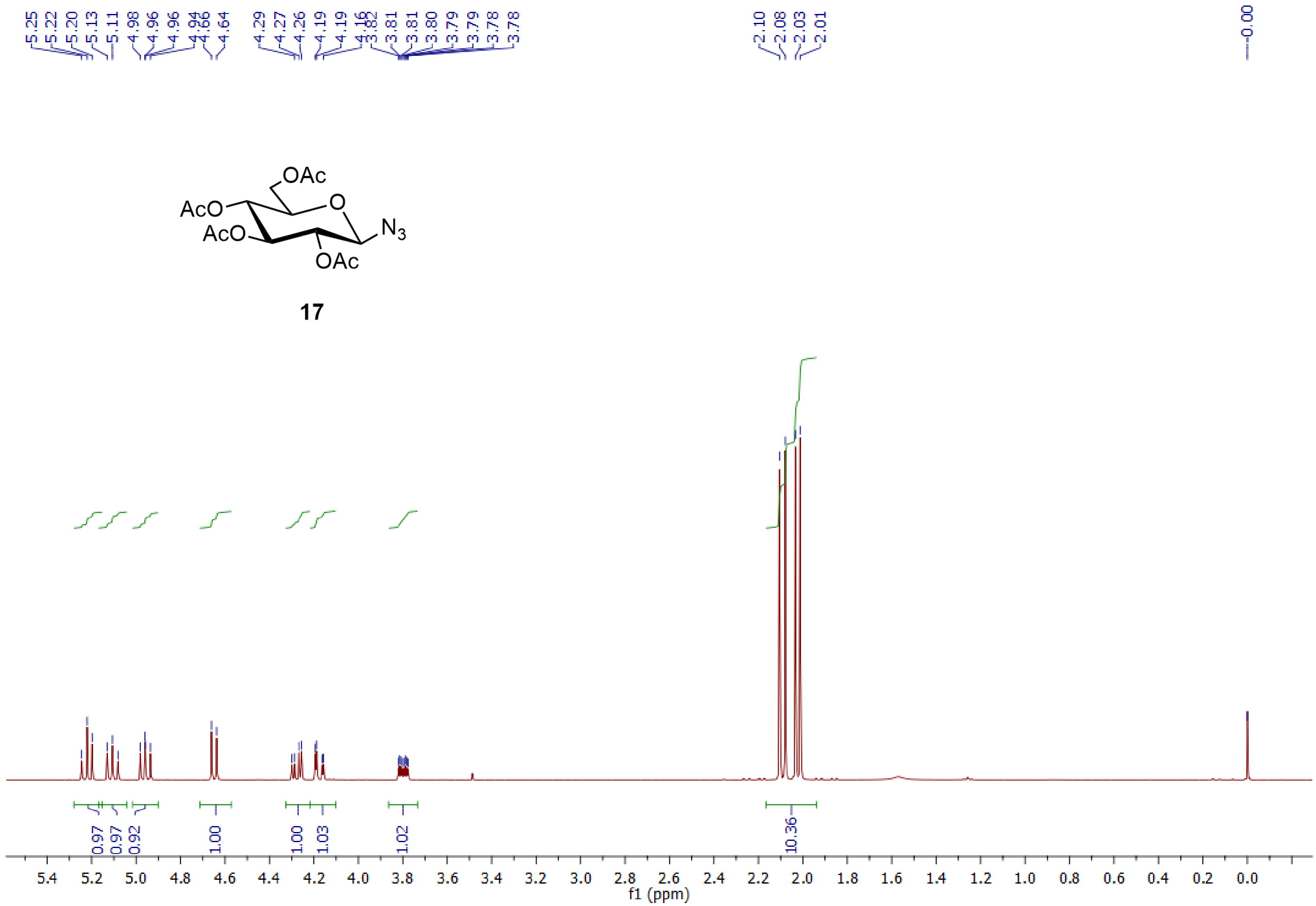

Fig. S25: ^{1}H NMR spectrum of compound **17**.

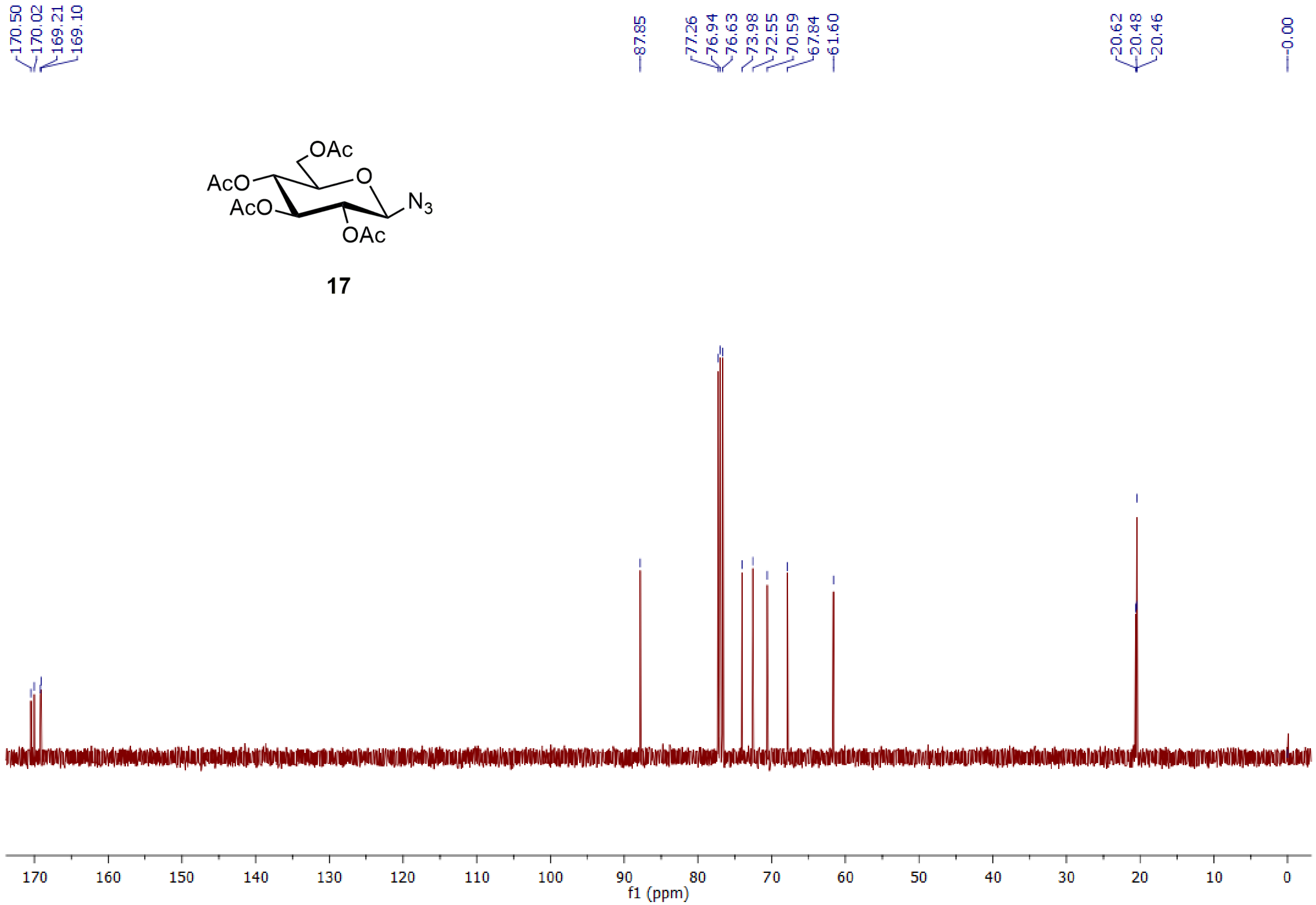

Fig. S26: ^{13}C NMR spectrum of compound **17**.

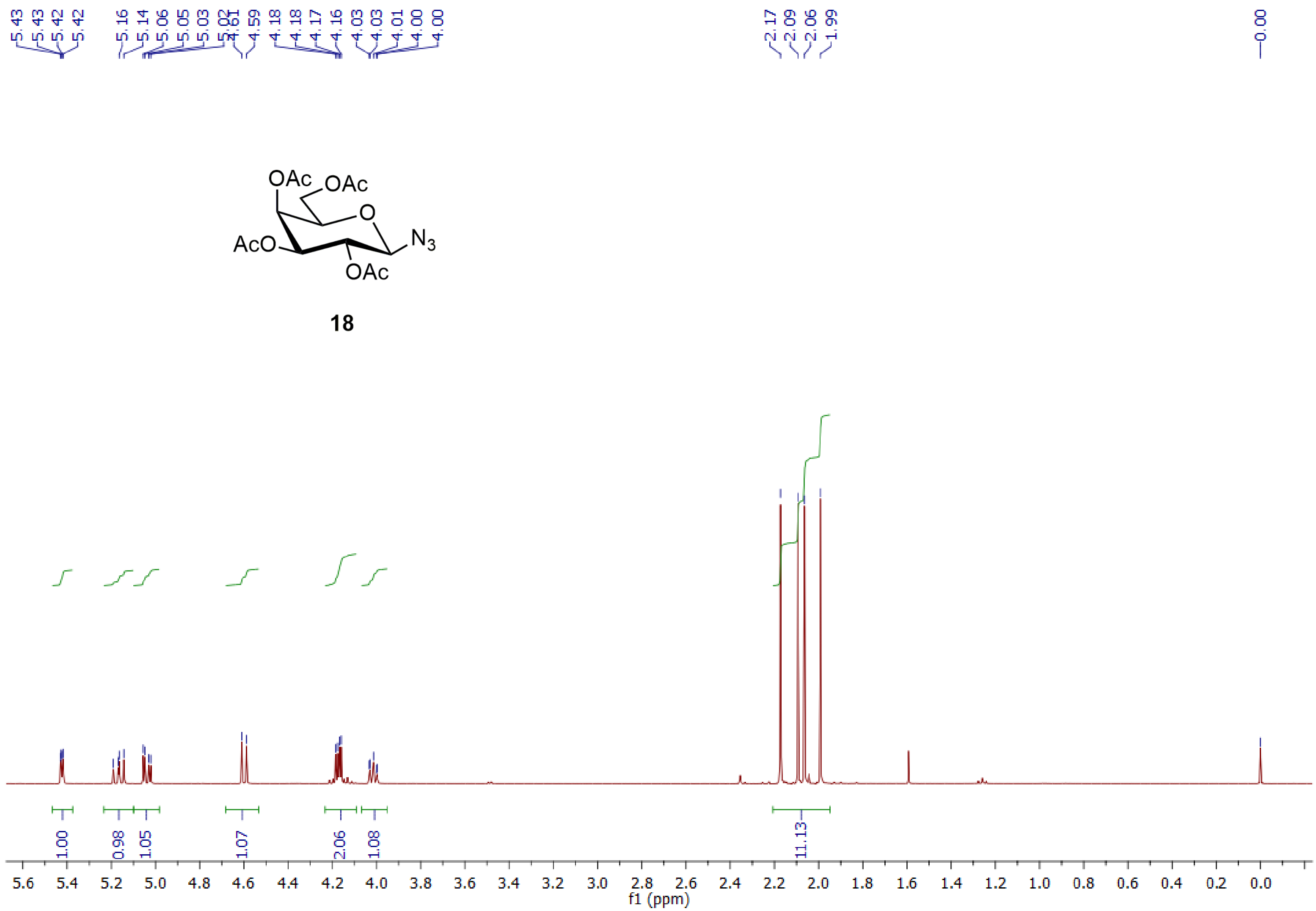

Fig. S27: ^{1}H NMR spectrum of compound **18**.

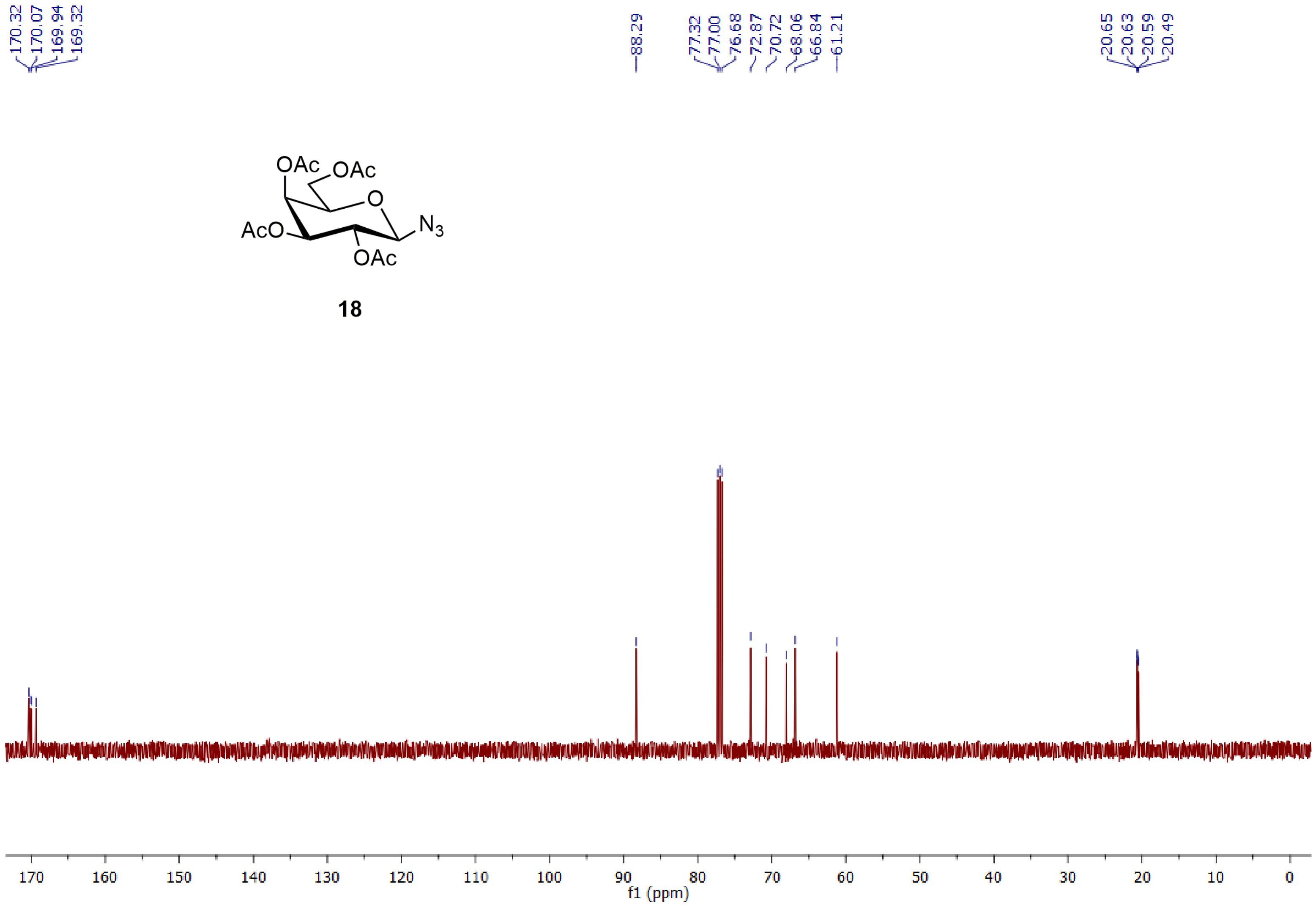

Fig. S28: ^{13}C NMR spectrum of compound **18**.

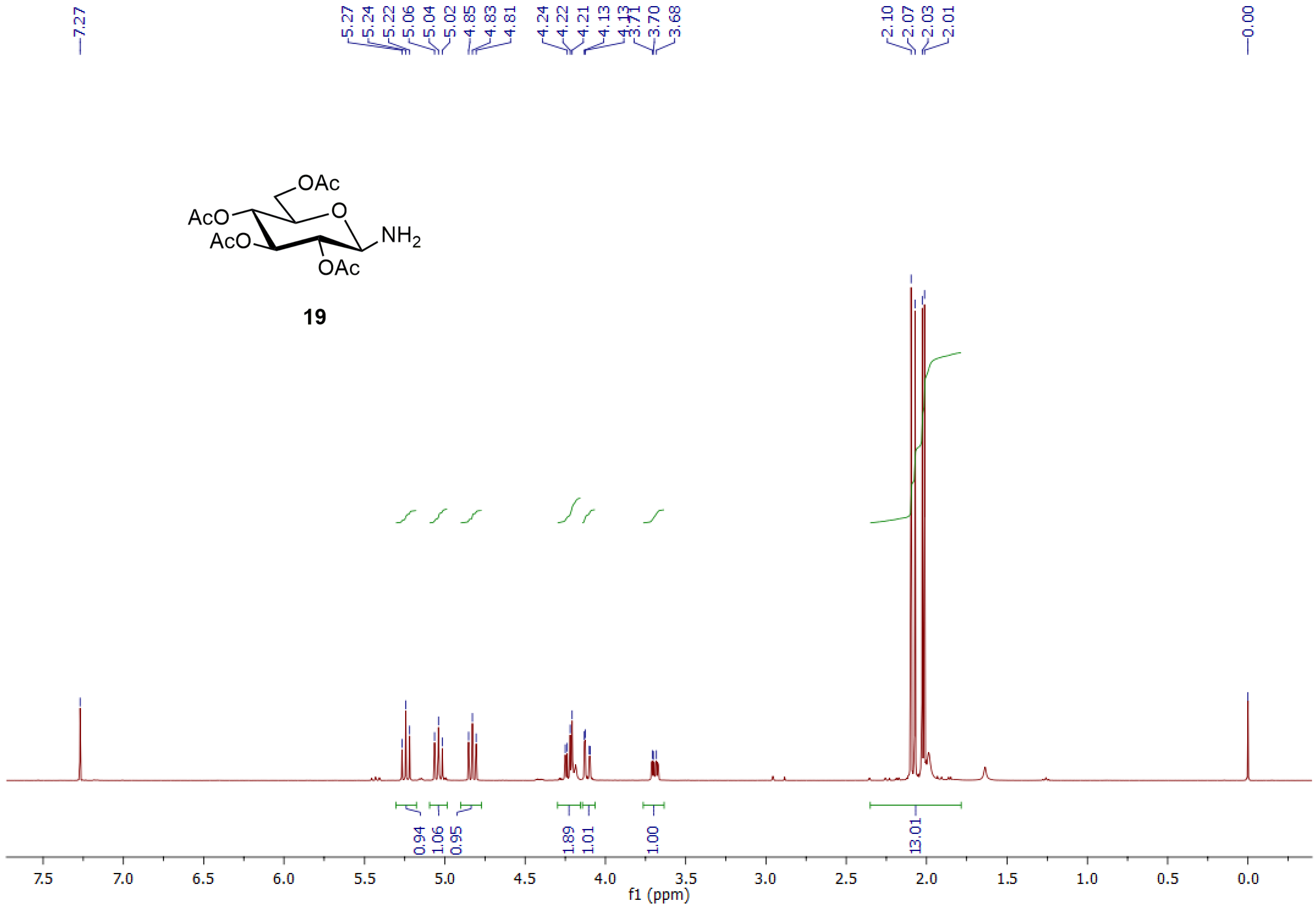

Fig. S29: ^{1}H NMR spectrum of compound **19**.

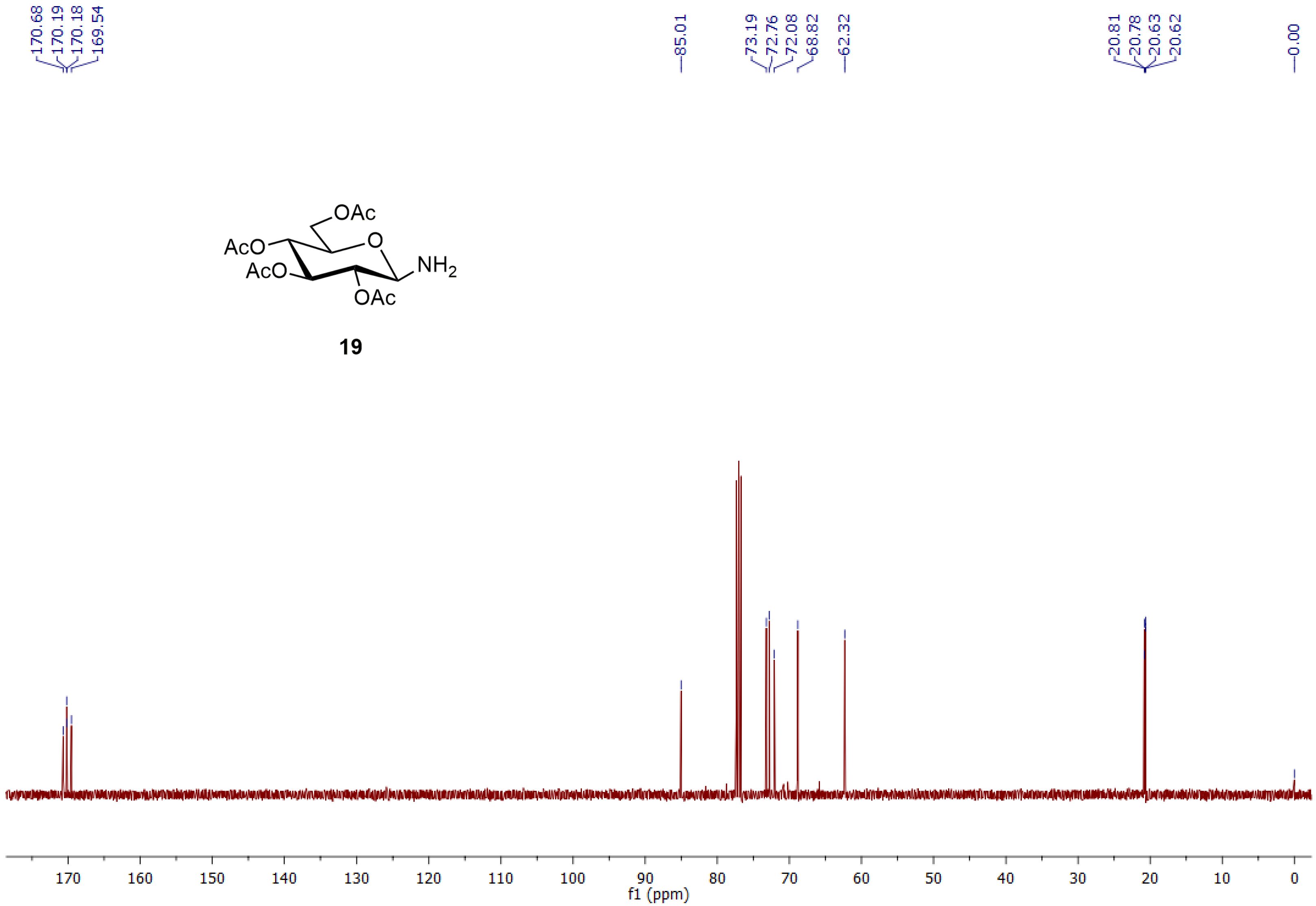

Fig. S30: ^{13}C NMR spectrum of compound **19**.

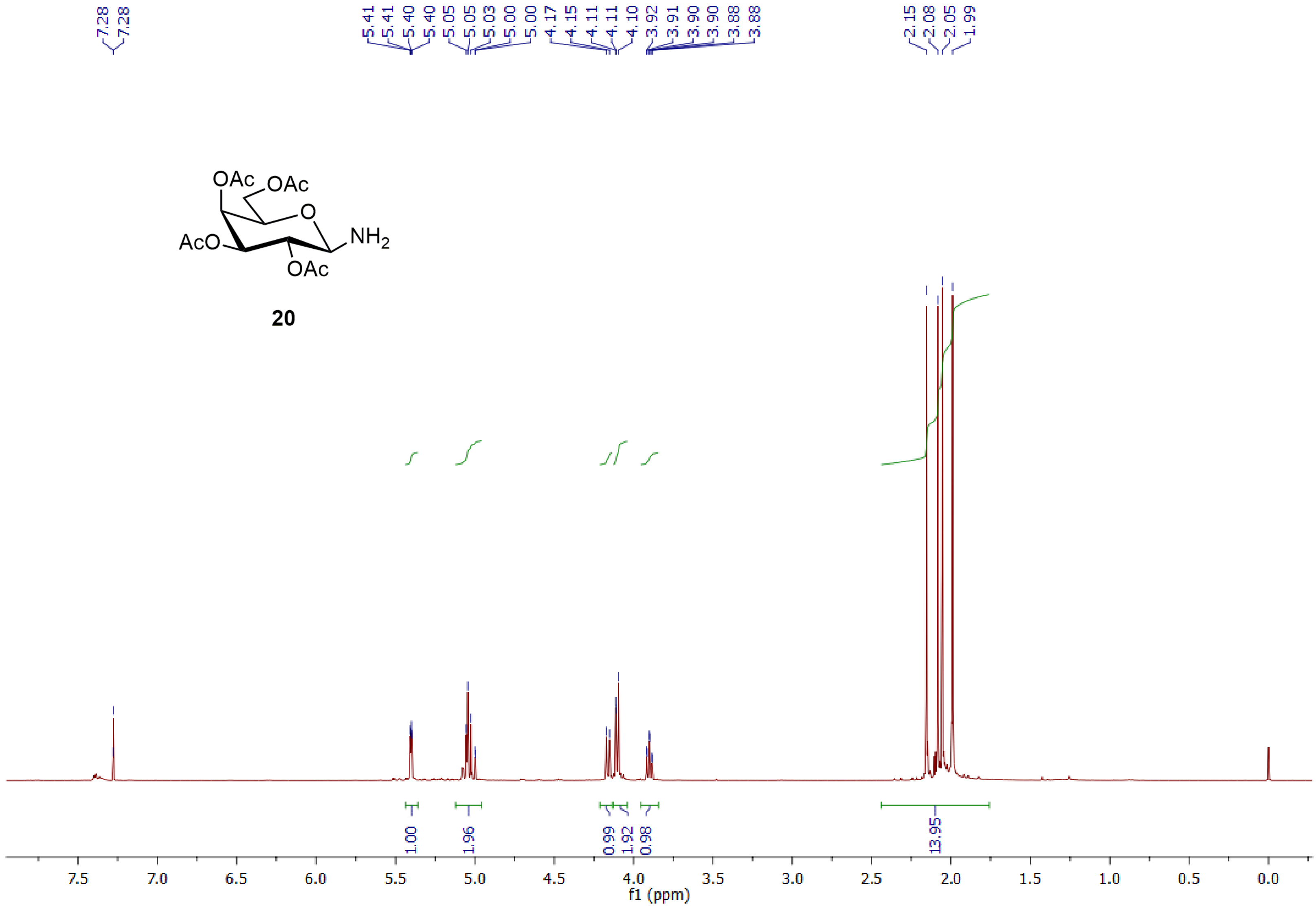

Fig. S31: ^{1}H NMR spectrum of compound **20**.

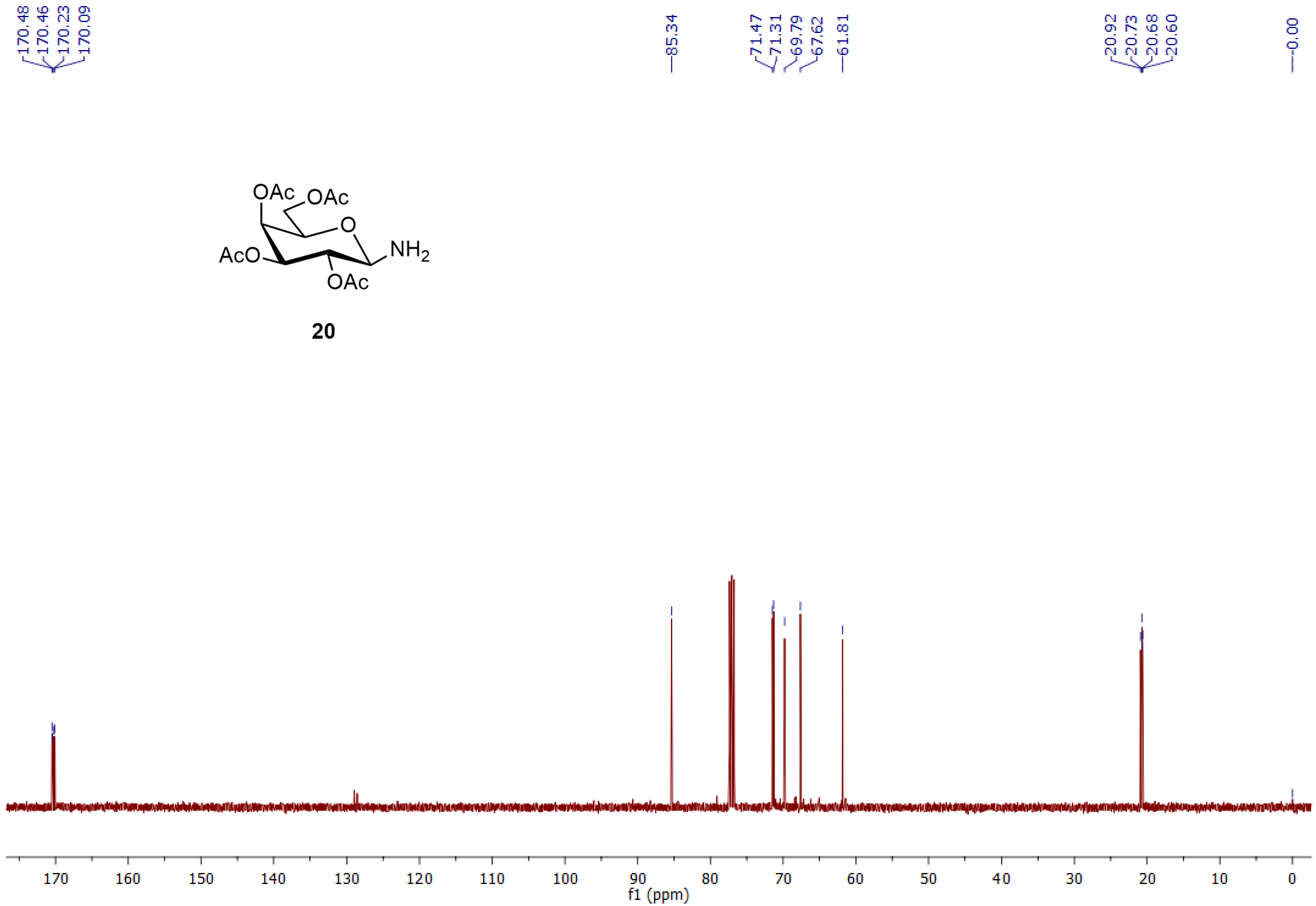

Fig. S32: ^{13}C NMR spectrum of compound **20**.

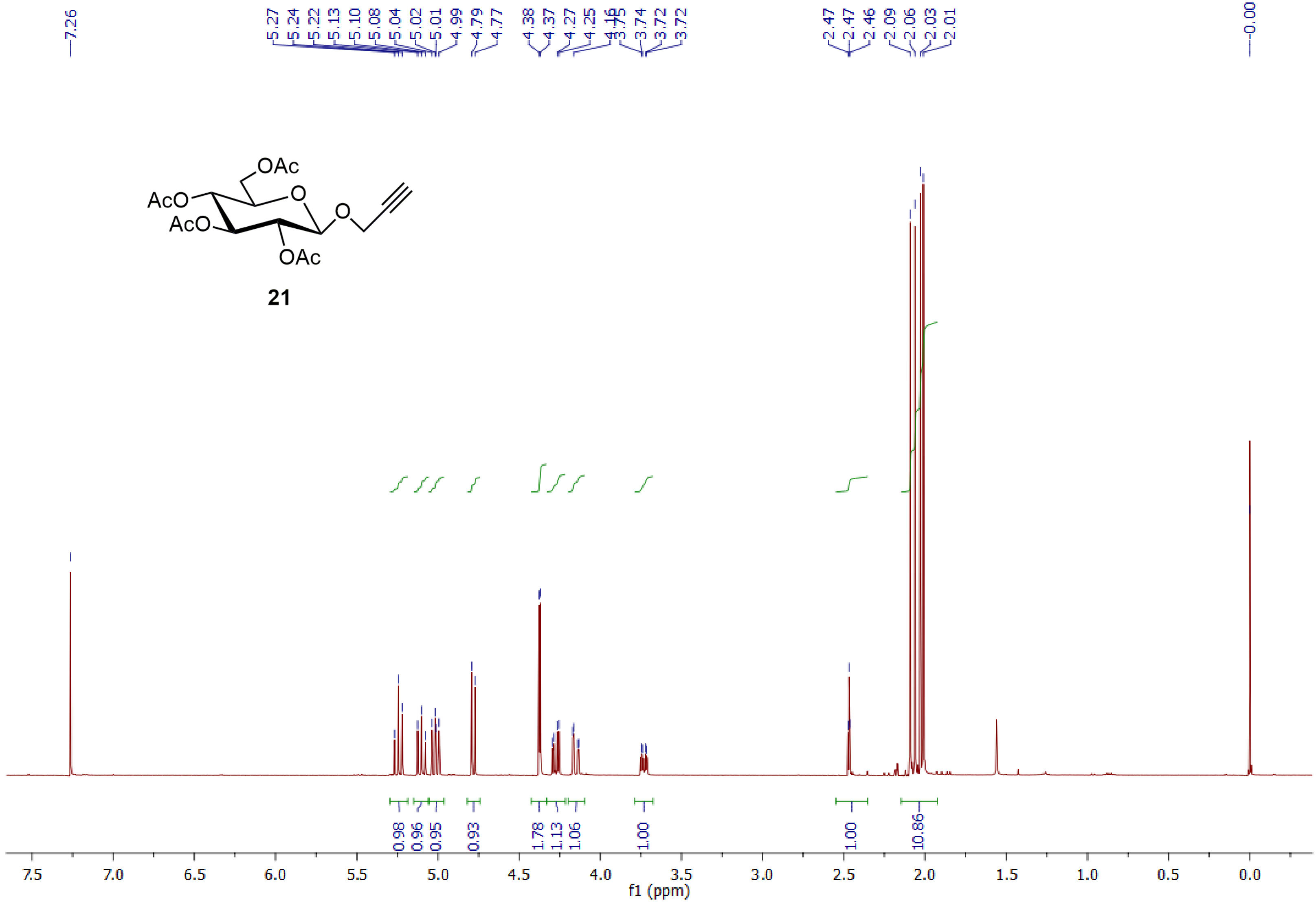

Fig. S33: ^{1}H NMR spectrum of compound **21**.

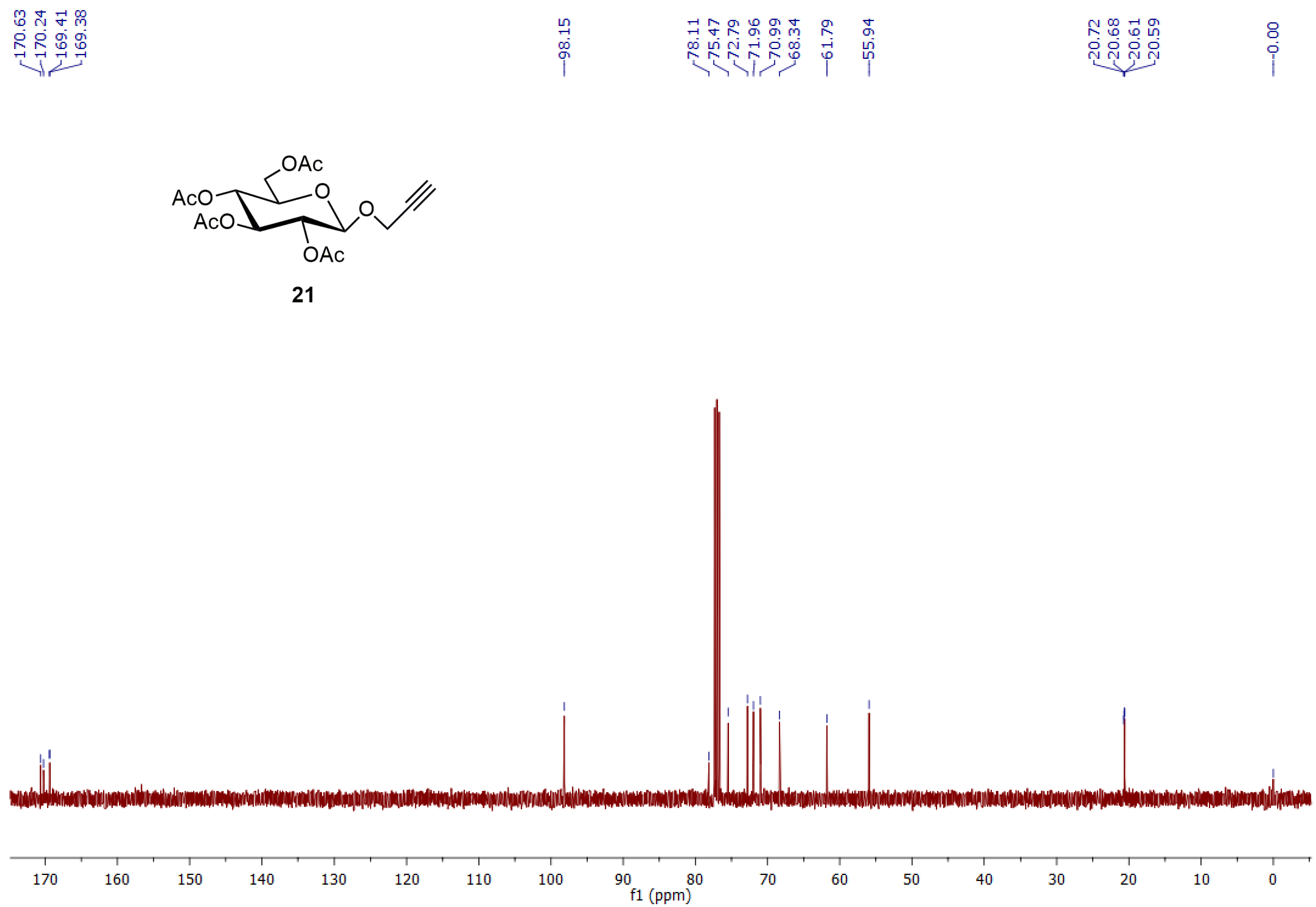

Fig. S34: ^{13}C NMR spectrum of compound **21**.

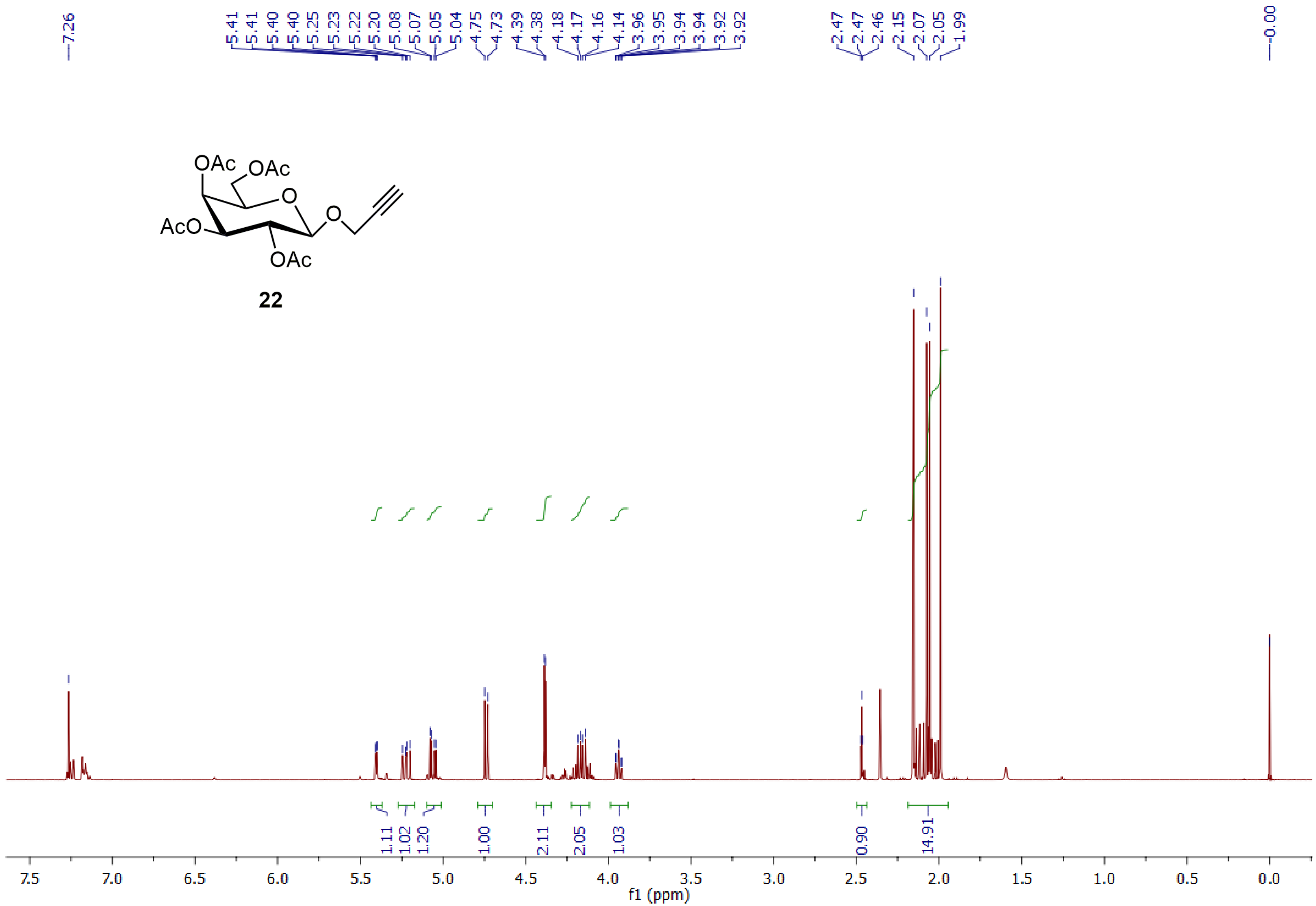

Fig. S35: ^{1}H NMR spectrum of compound **22**.

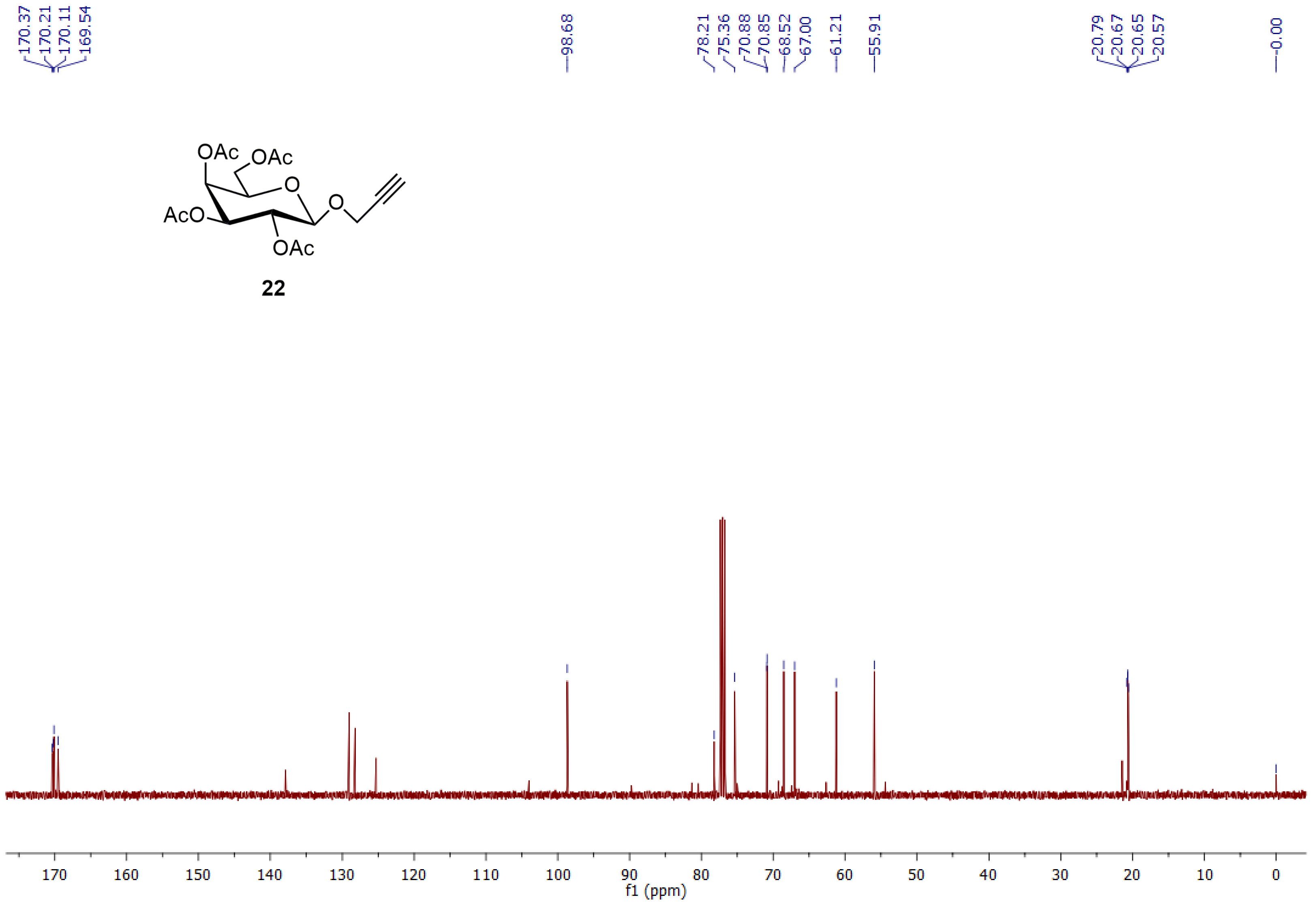

Fig. S36: ^{13}C NMR spectrum of compound **22**.

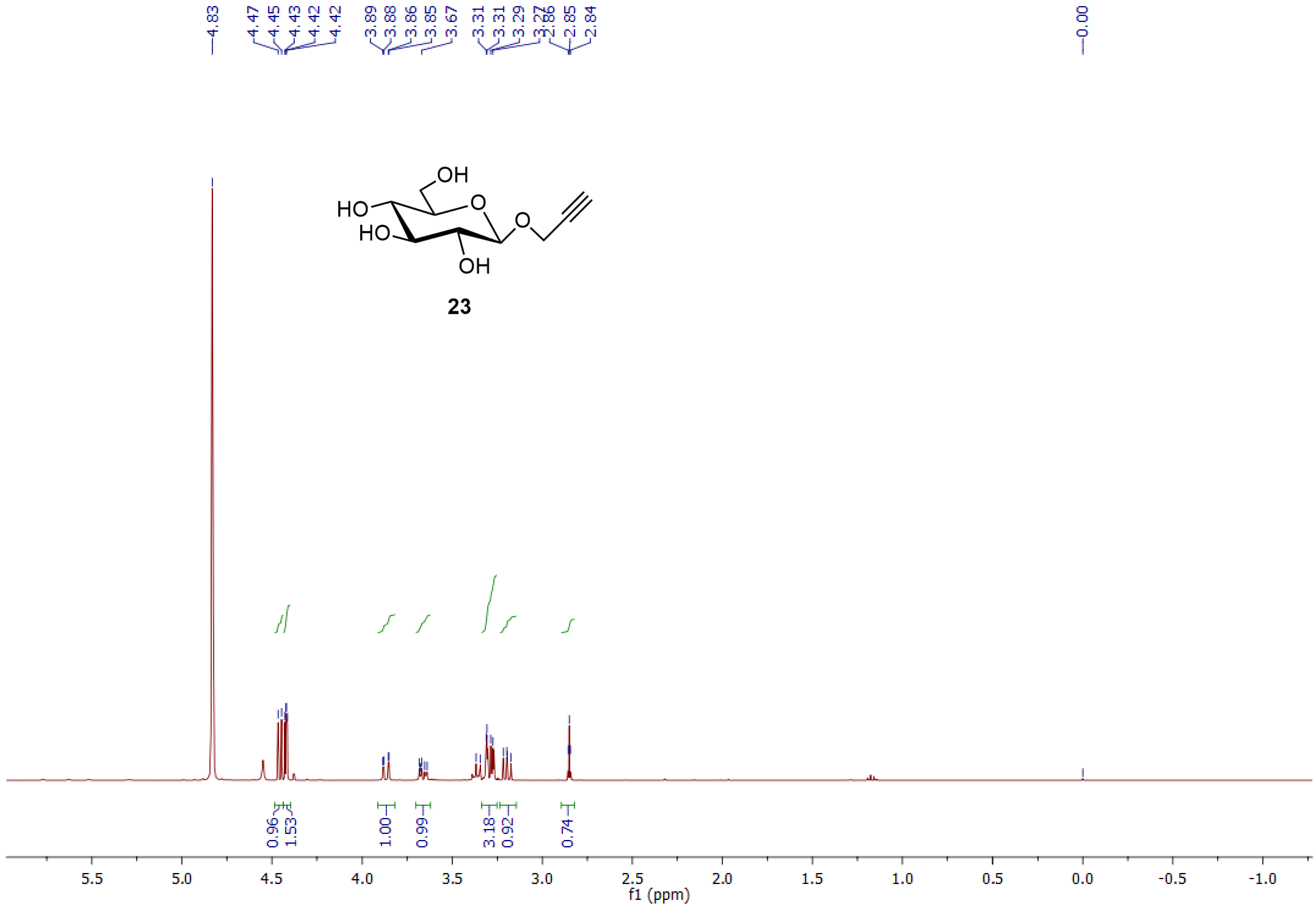

Fig. S37: ^{1}H NMR spectrum of compound **23**.

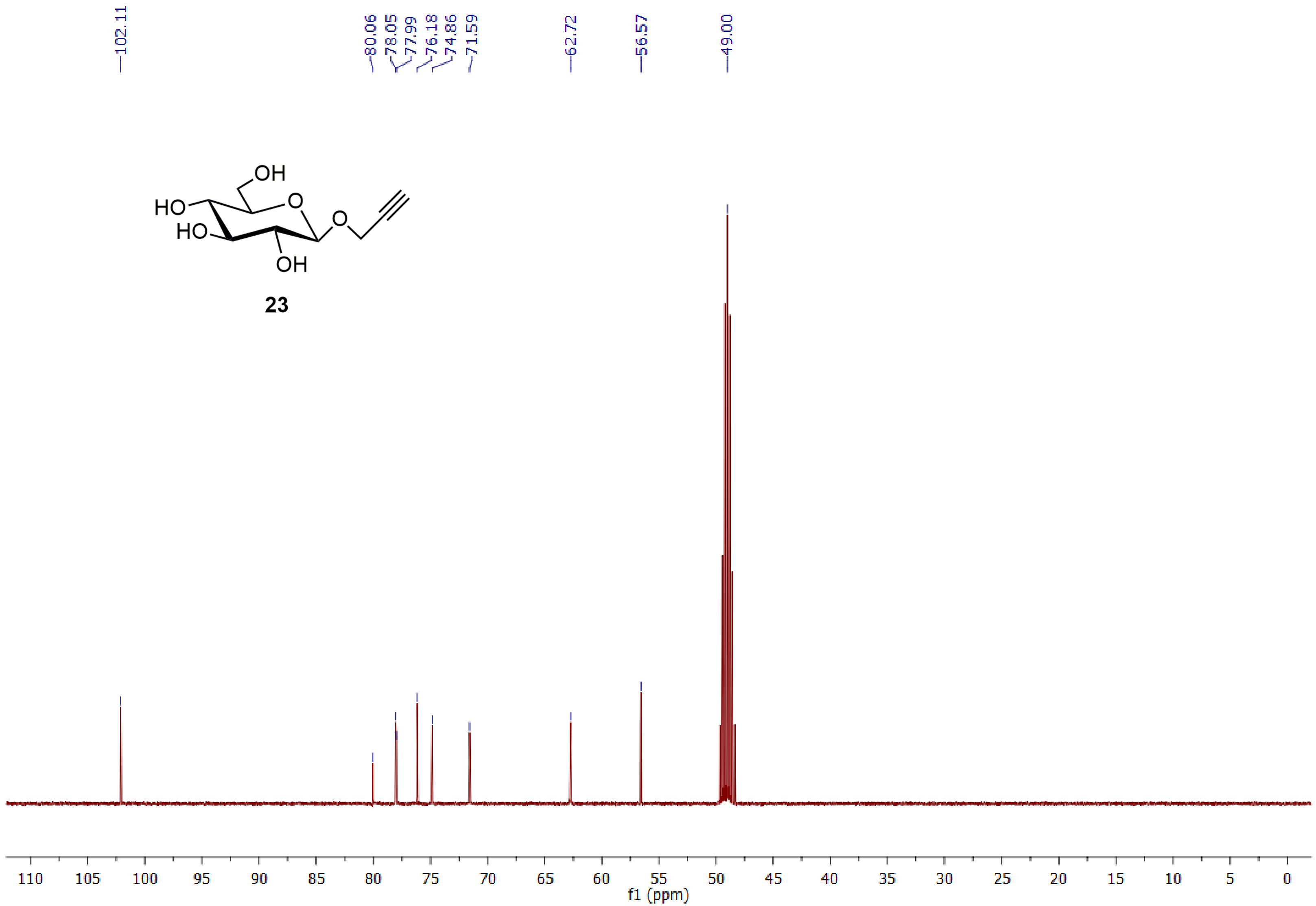

Fig. S38: ^{13}C NMR spectrum of compound **23**.

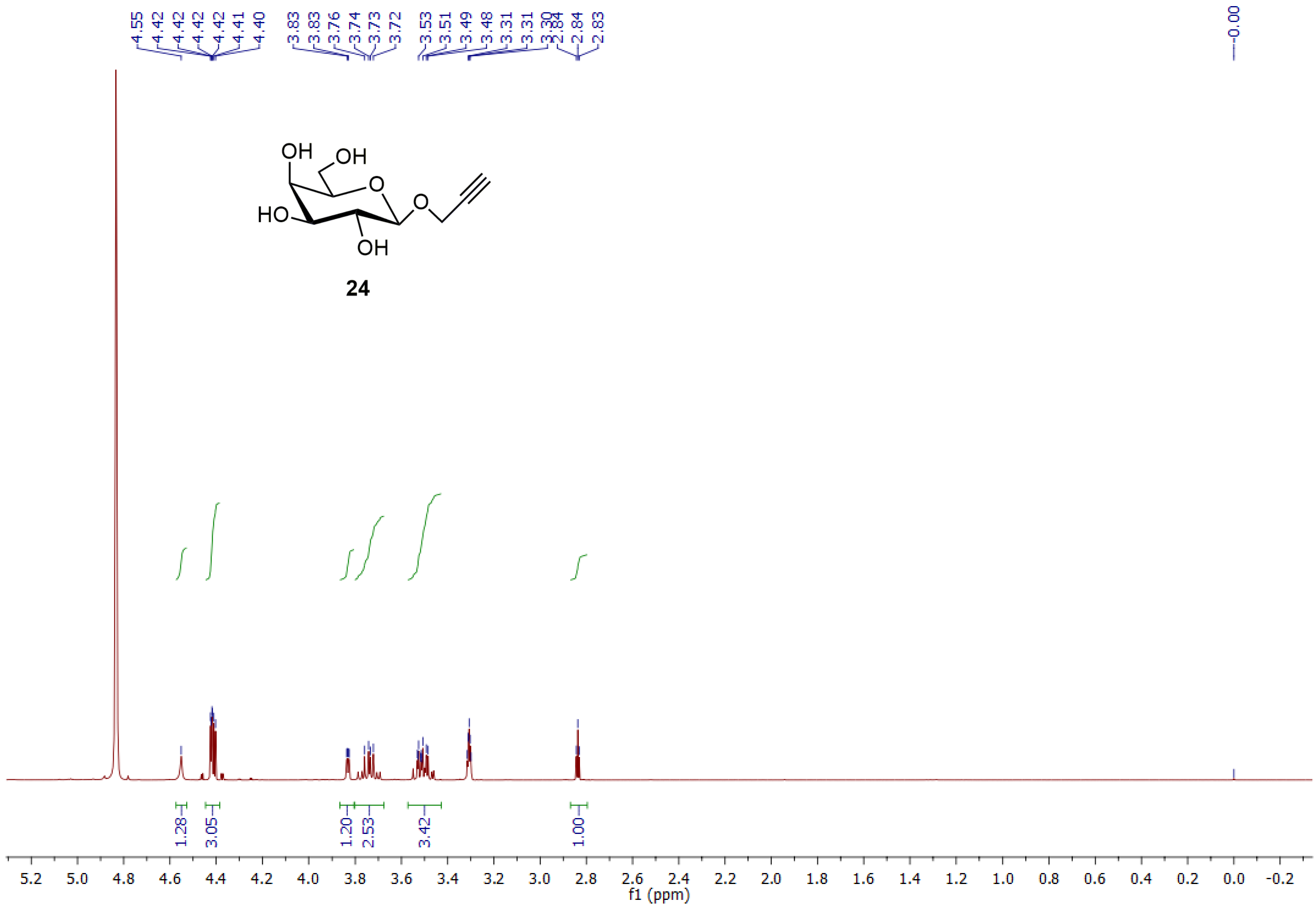

Fig. S39: ^{1}H NMR spectrum of compound **24**.

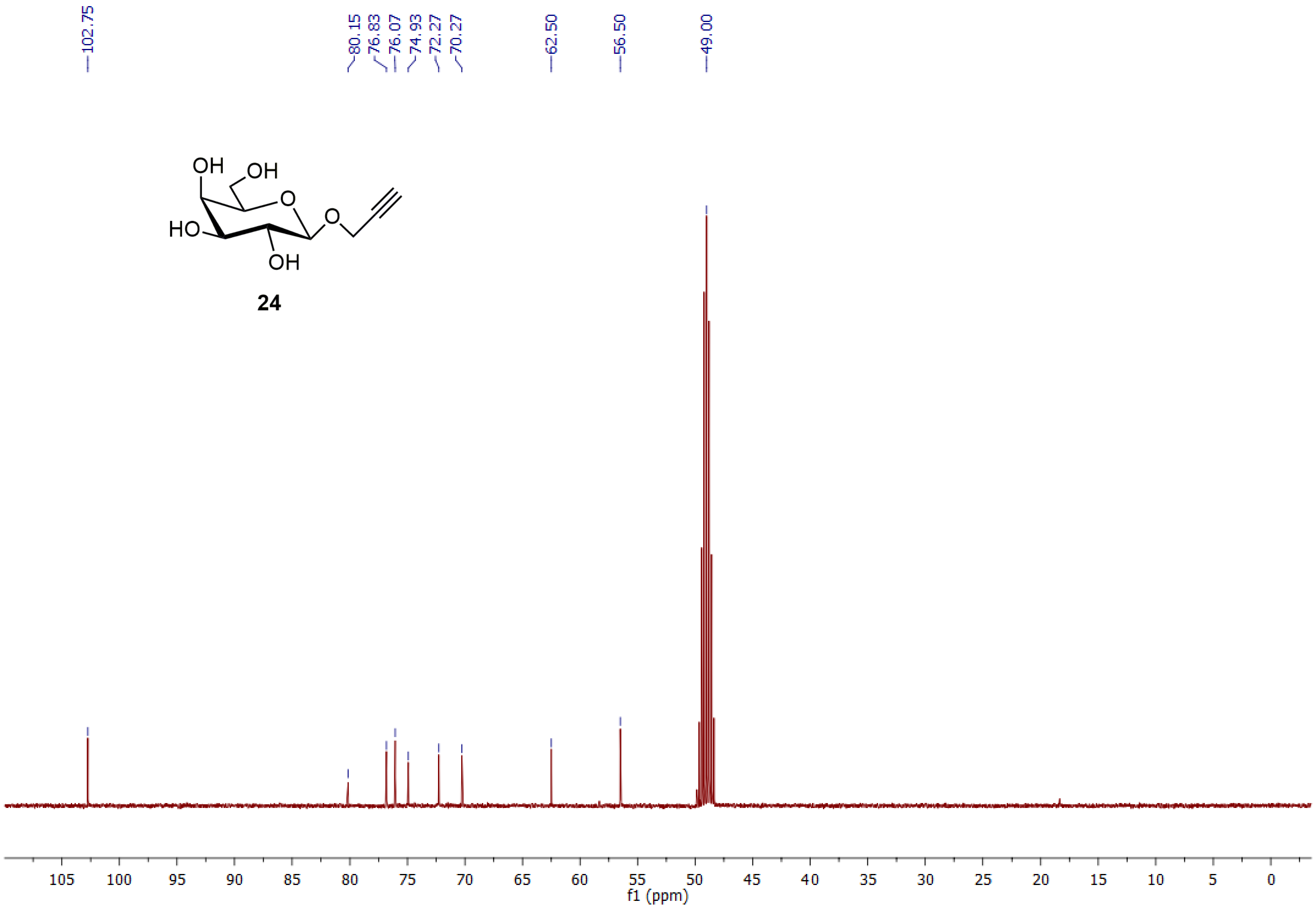

Fig. S40: ^{13}C NMR spectrum of compound **24**.

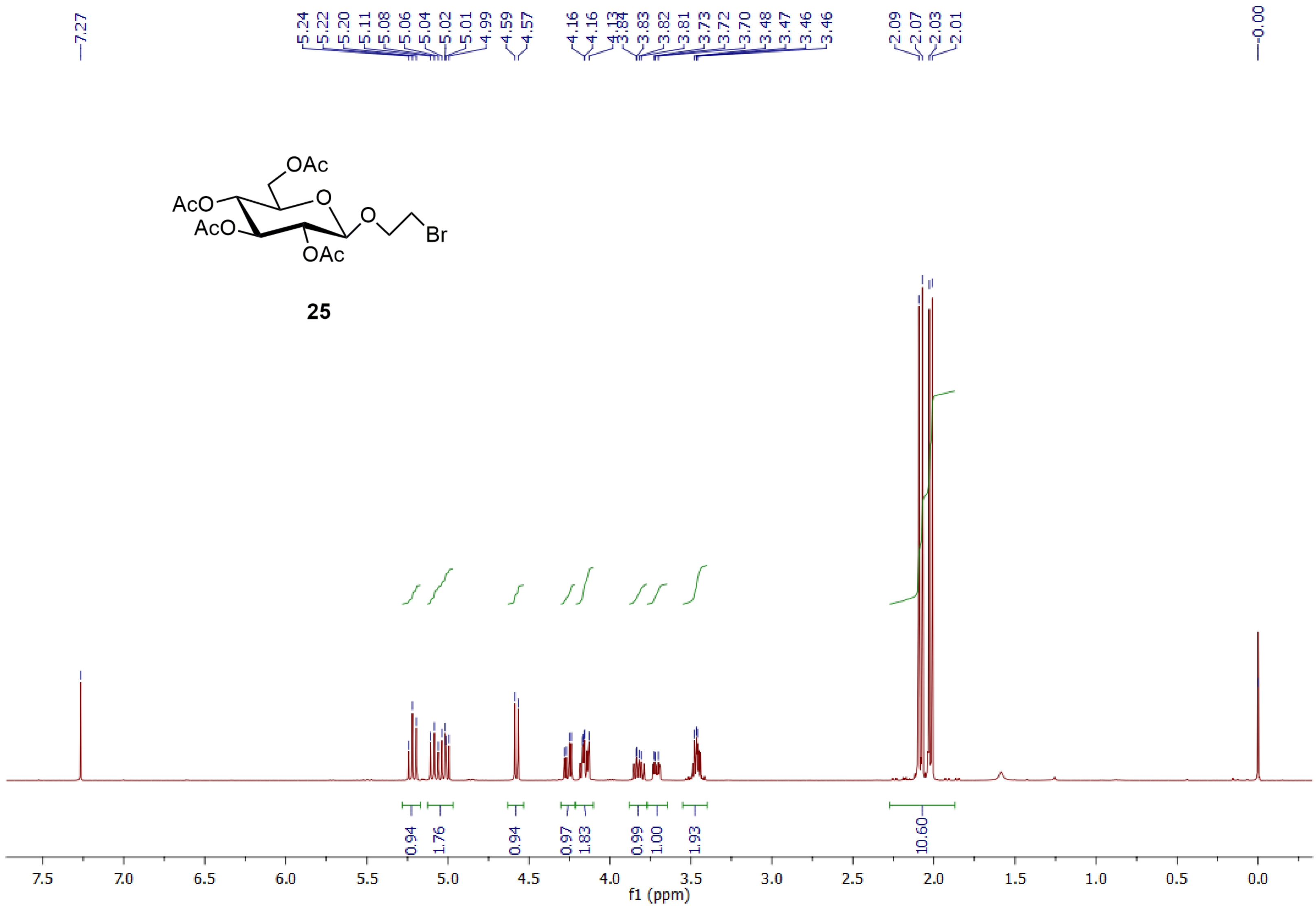

Fig. S41: ^{1}H NMR spectrum of compound **25**.

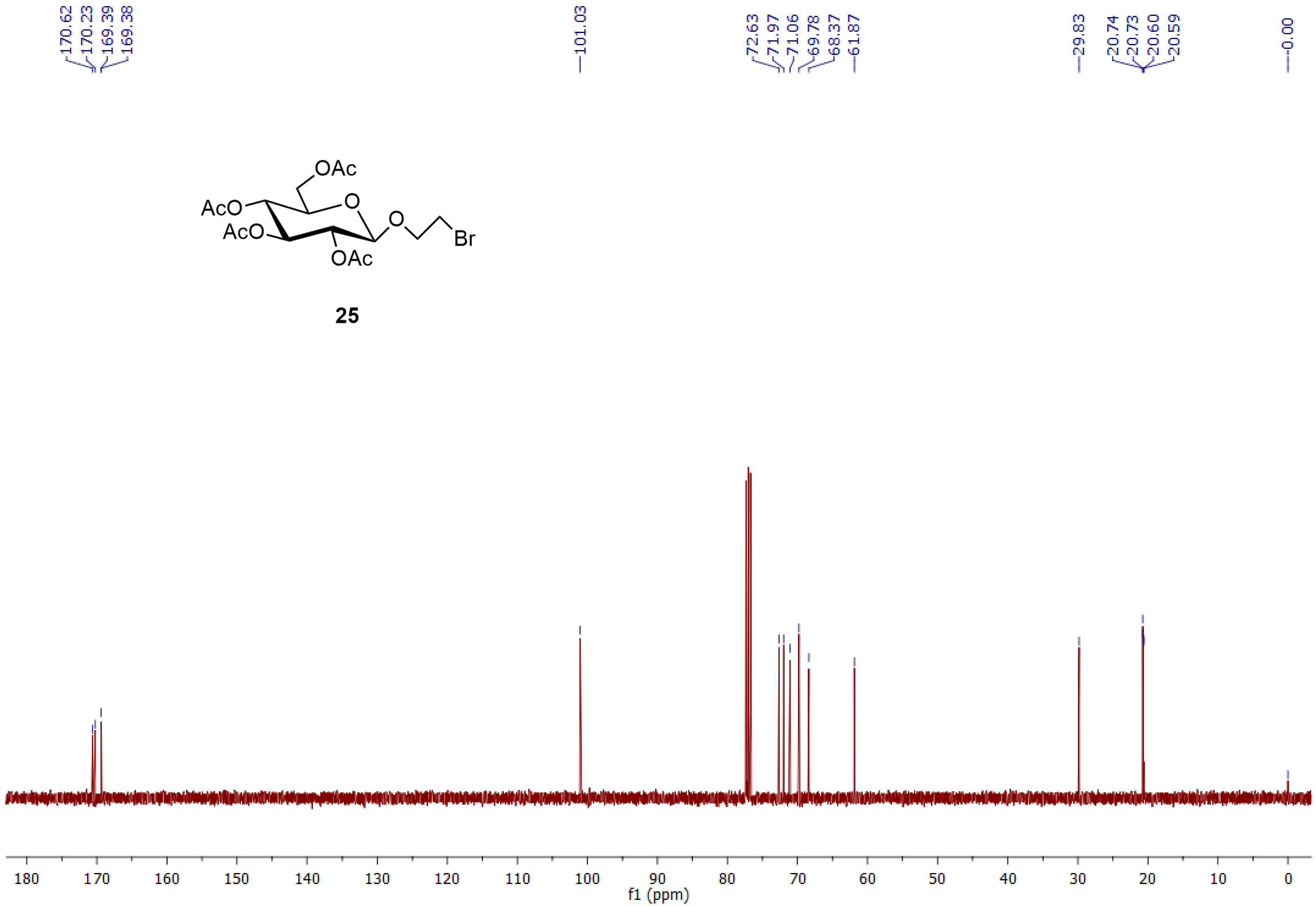

Fig. S42: ^{13}C NMR spectrum of compound **25**.

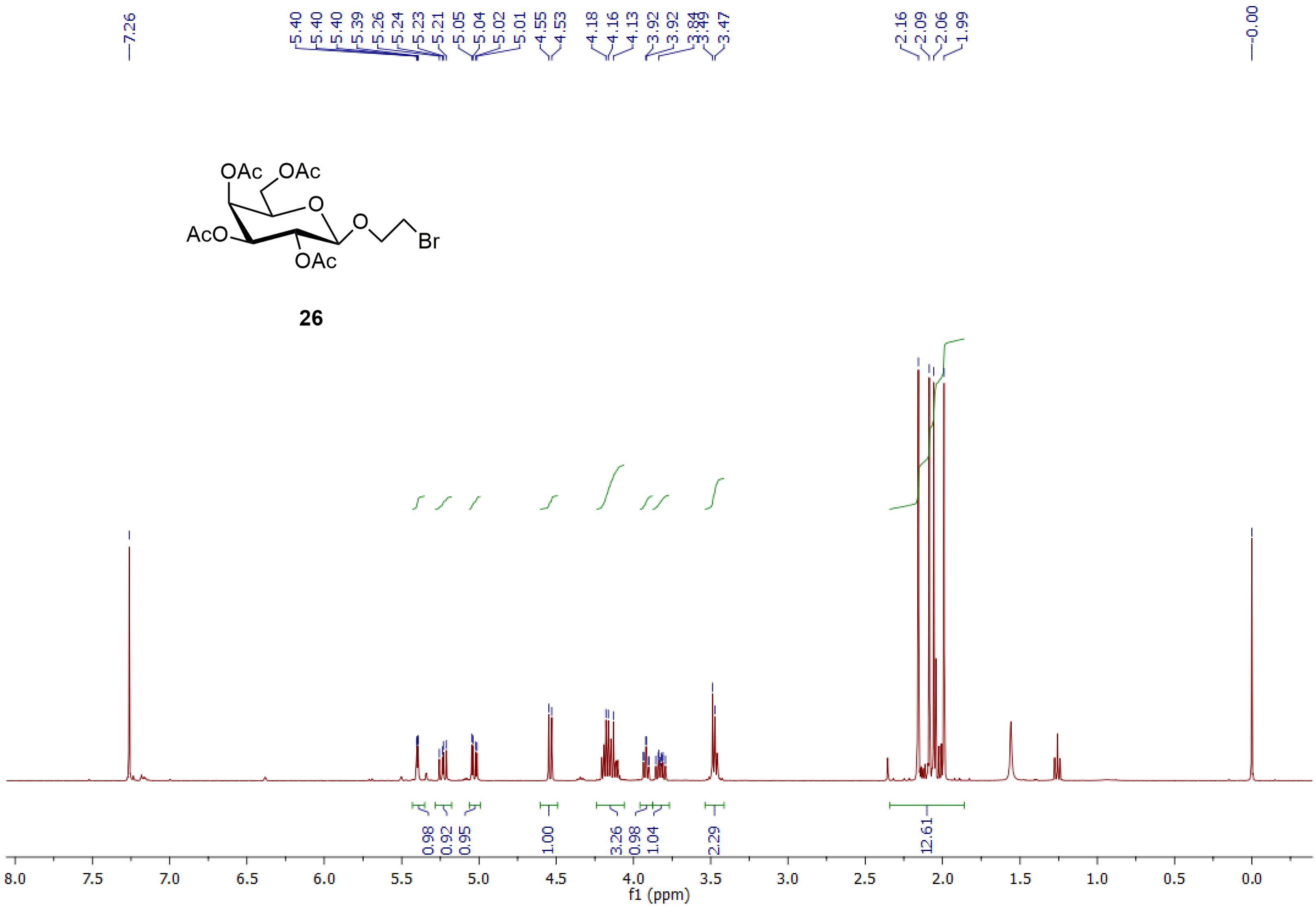

Fig. S43: ^{1}H NMR spectrum of compound **26**.

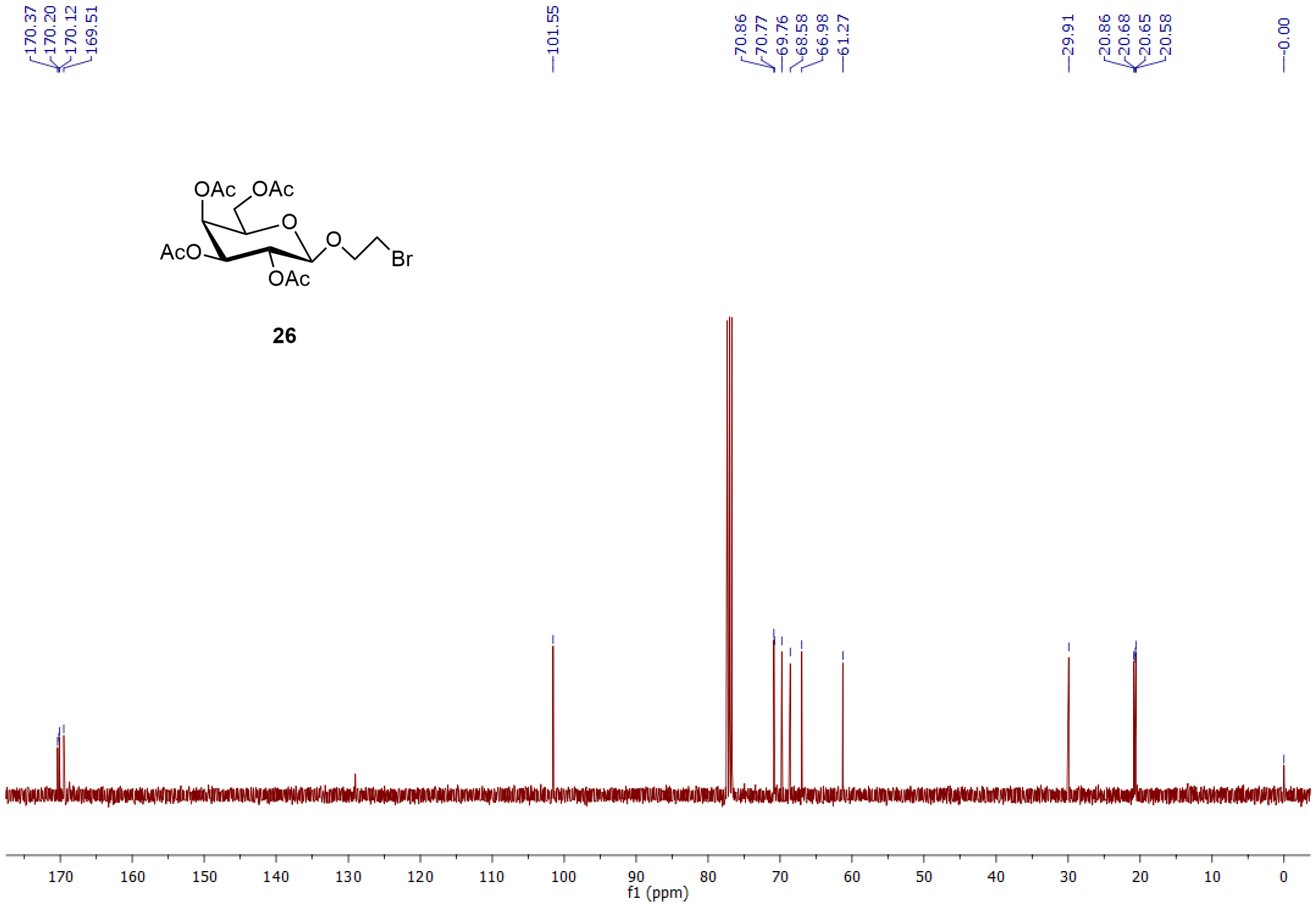

Fig. S44: ^{13}C NMR spectrum of compound **26**.

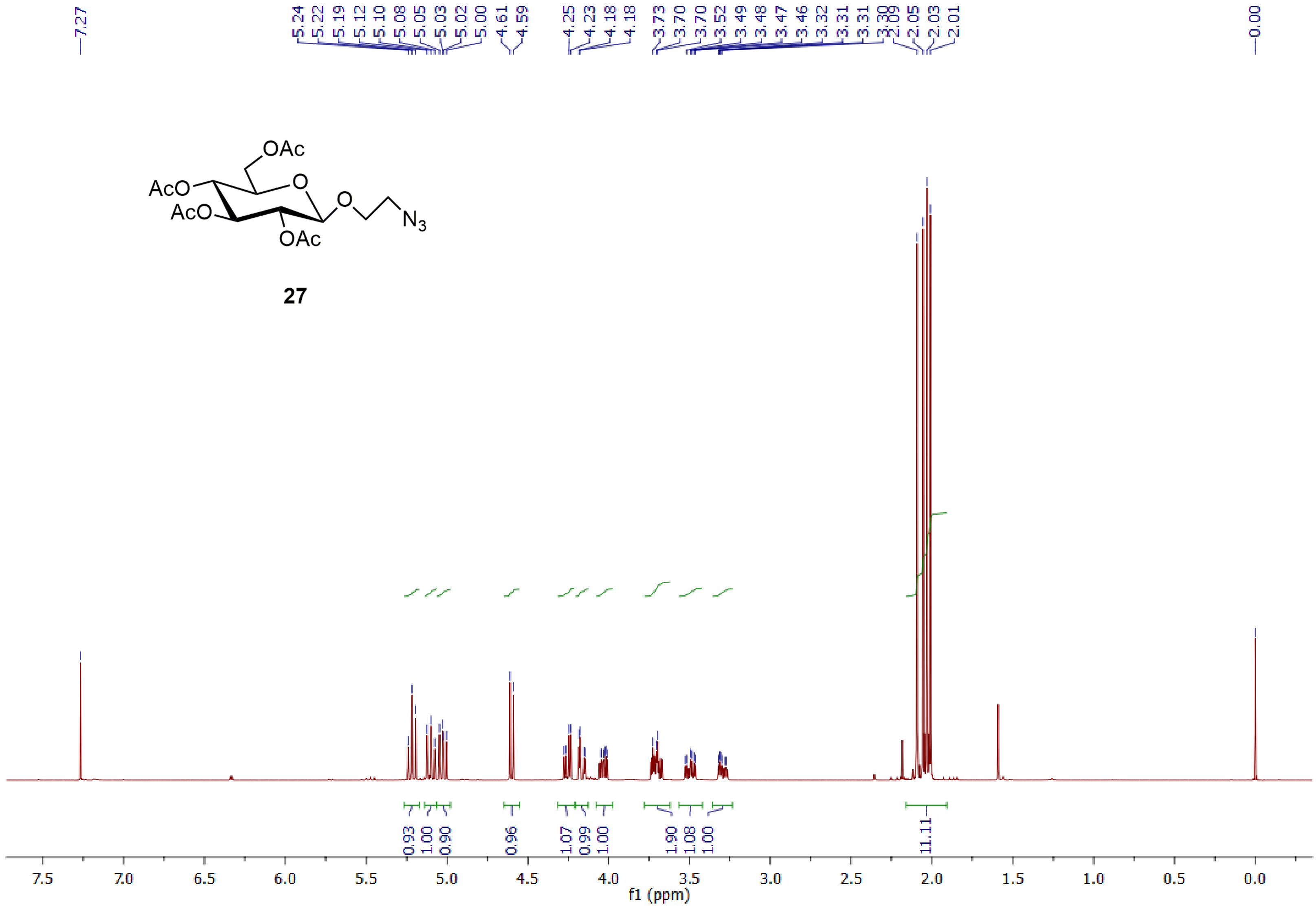

Fig. S45: ^{1}H NMR spectrum of compound **27**.

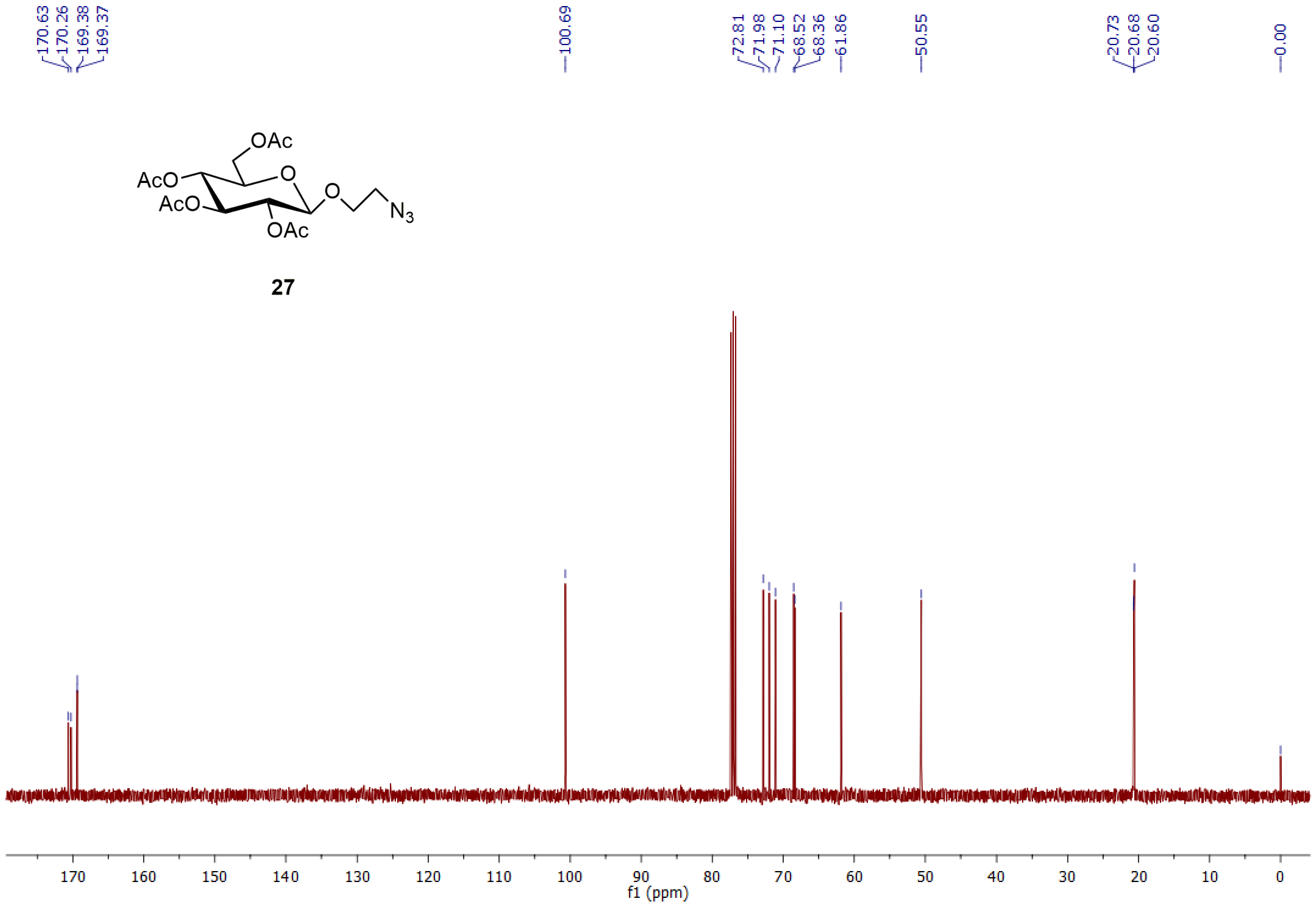

Fig. S46: ^{13}C NMR spectrum of compound **27**.

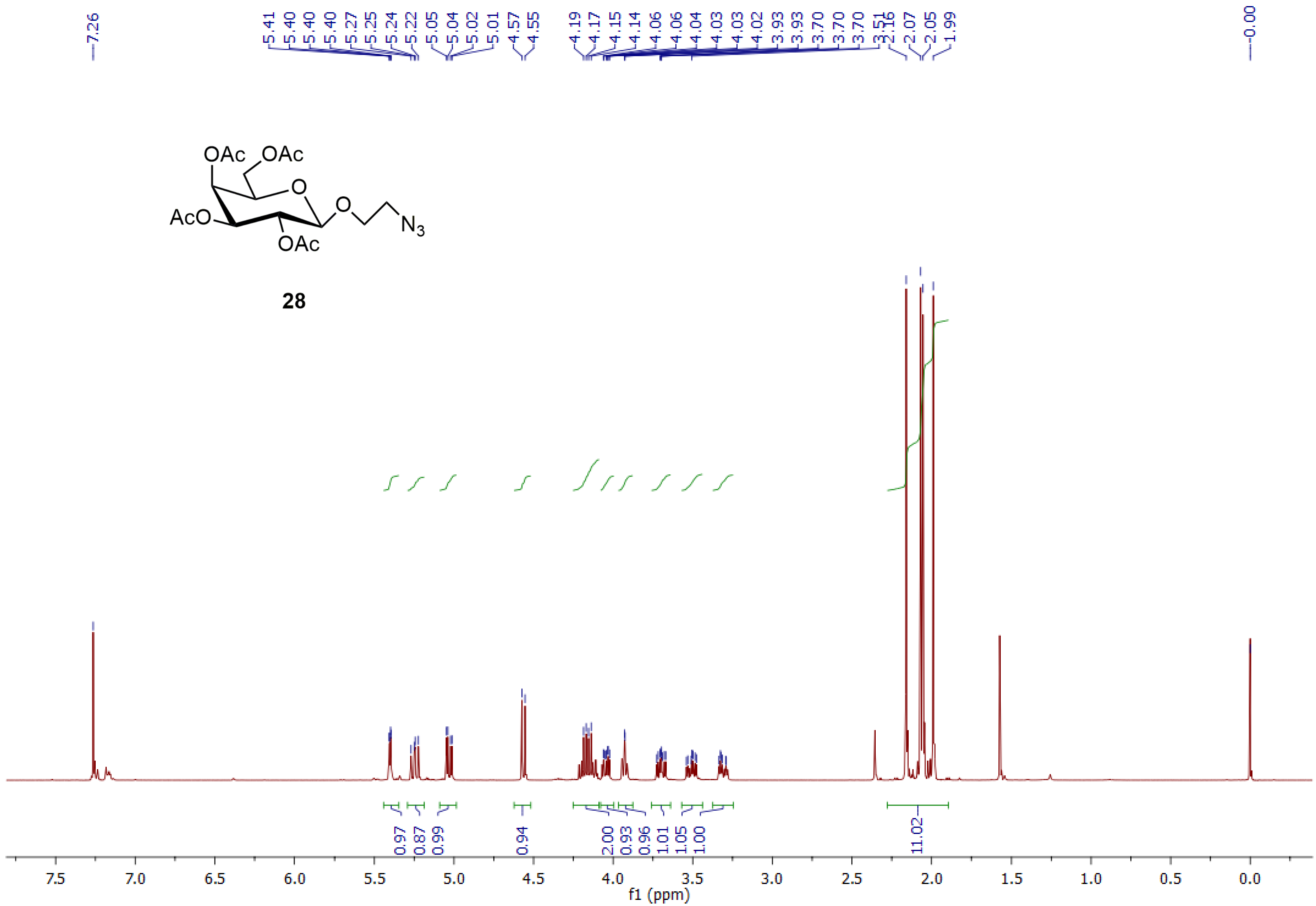

Fig. S47: ^{1}H NMR spectrum of compound **28**.

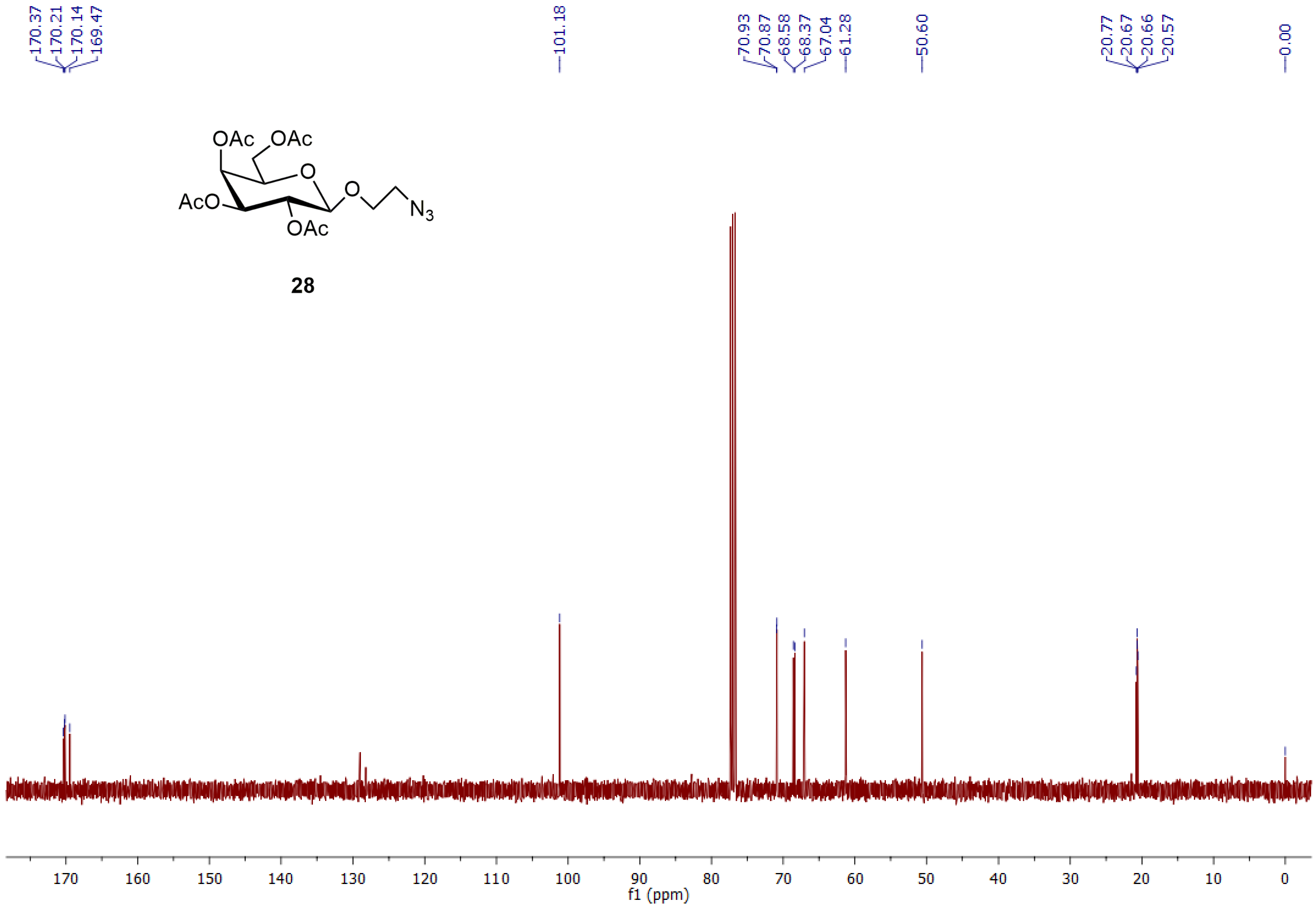

Fig. S48: ^{13}C NMR spectrum of compound **28**.

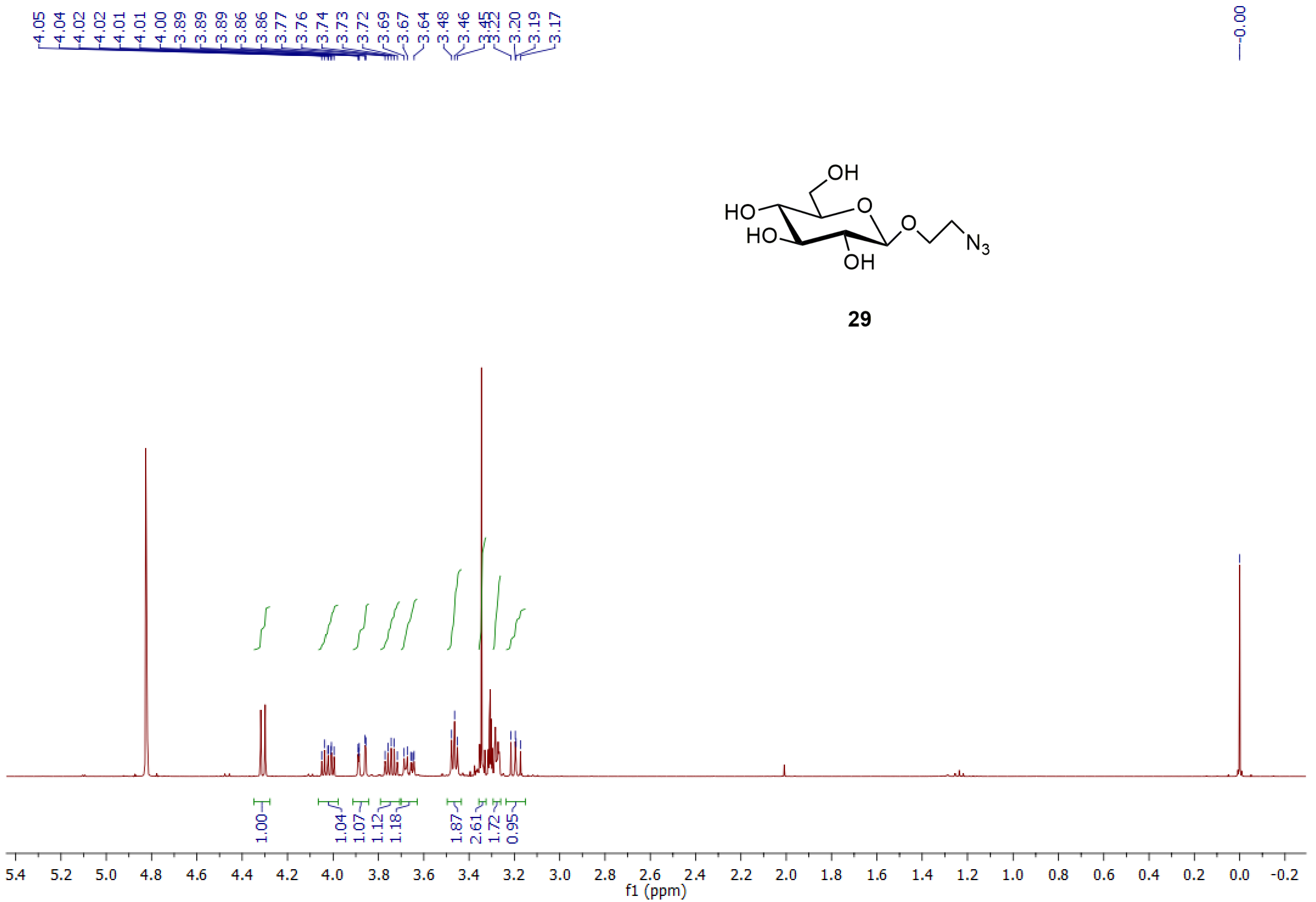

Fig. S49: ^{1}H NMR spectrum of compound **29**.

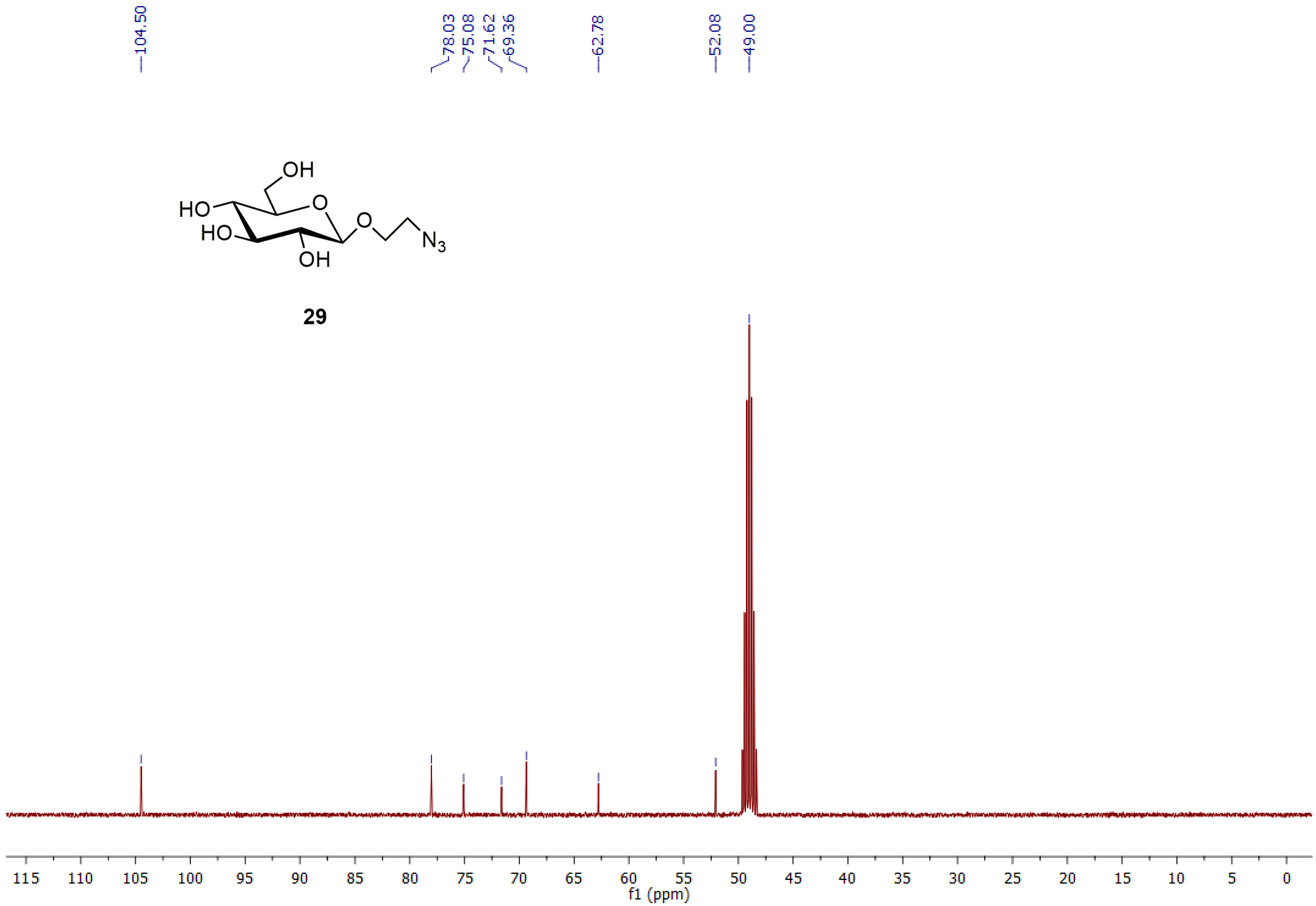

Fig. S50: ^{13}C NMR spectrum of compound **29**.

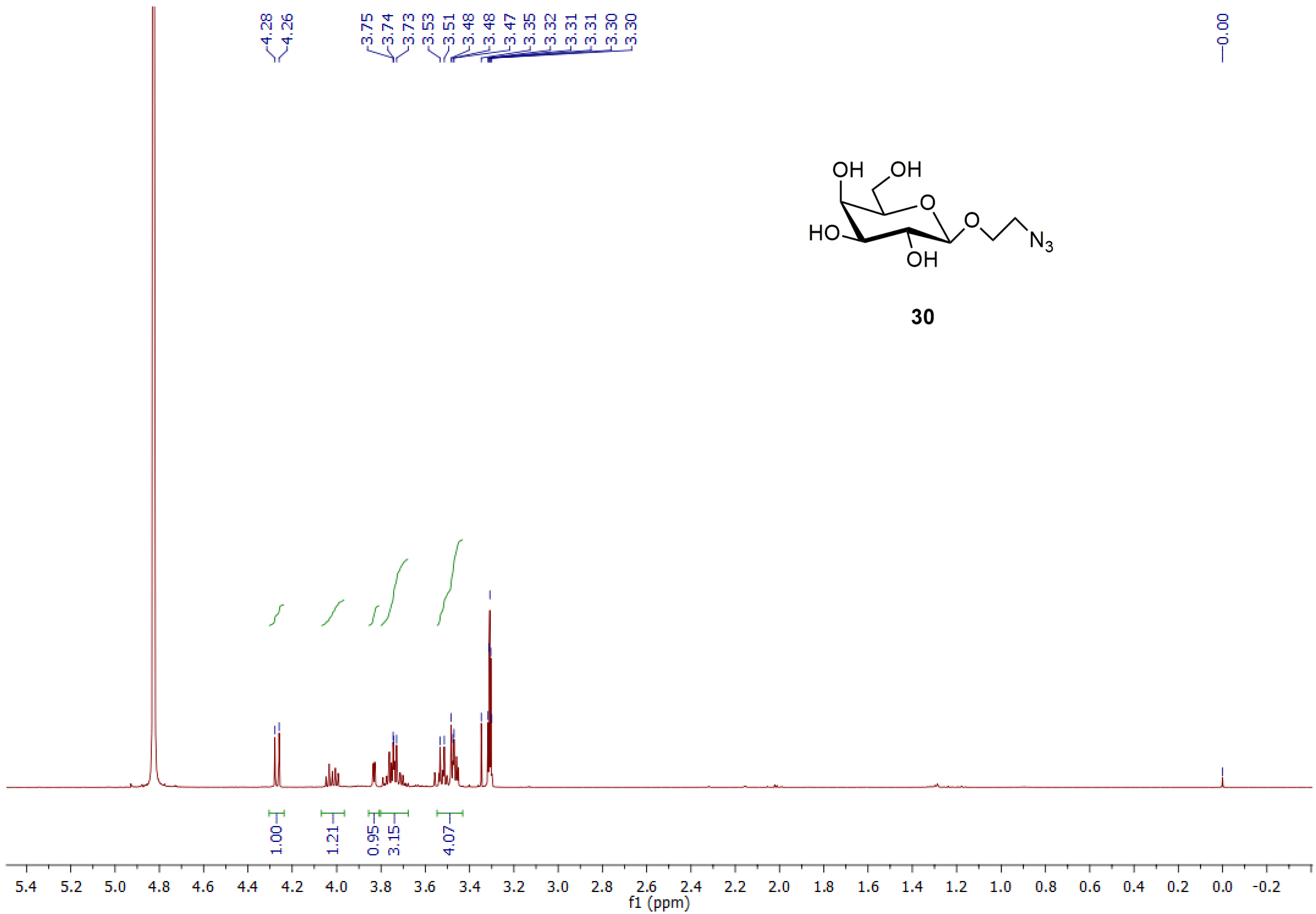

Fig. S51: ^{1}H NMR spectrum of compound **30**.

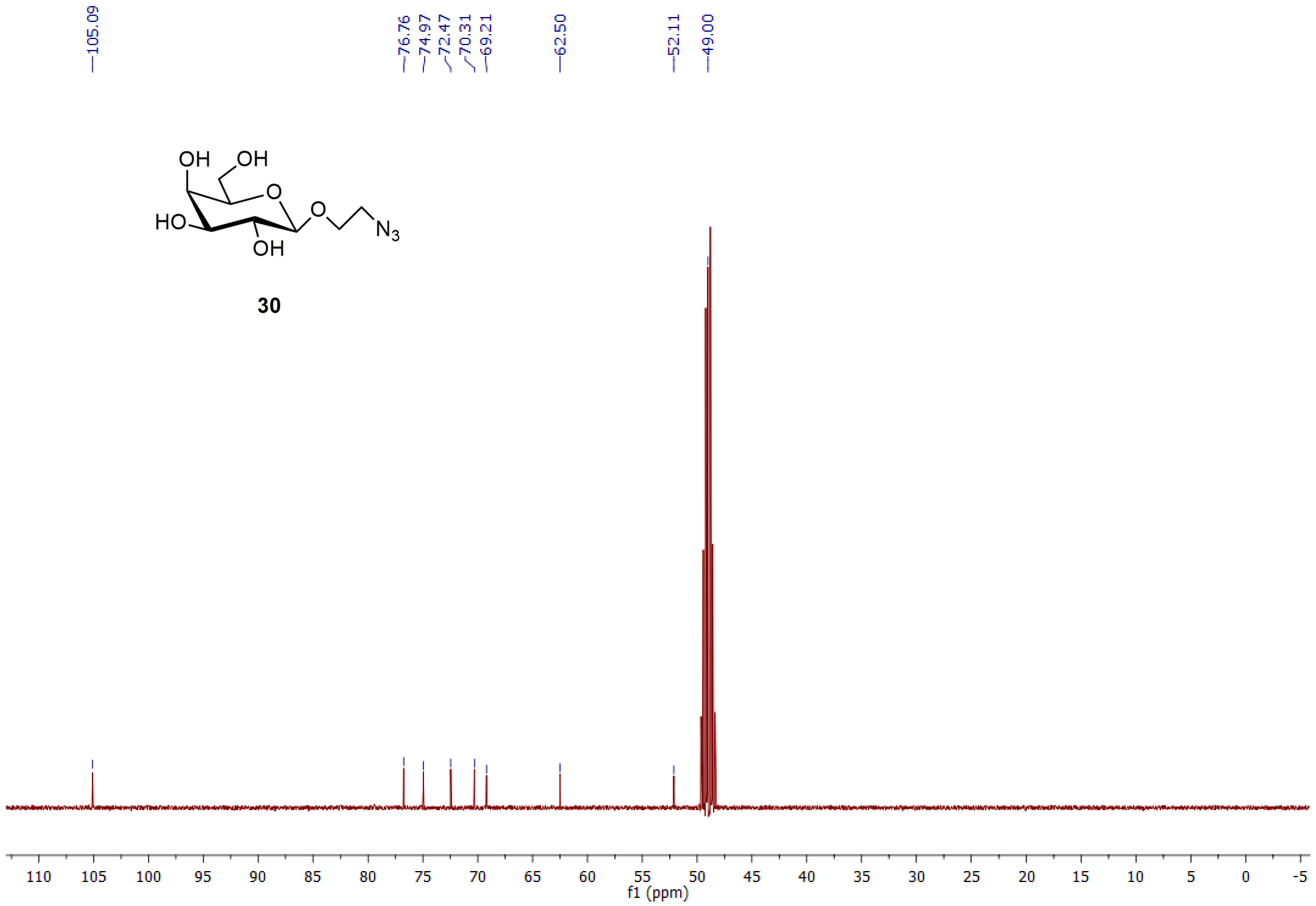

Fig. S52: ^{13}C NMR spectrum of compound **30**.

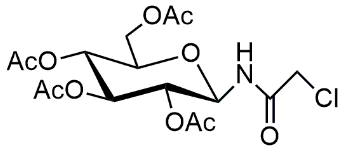

31

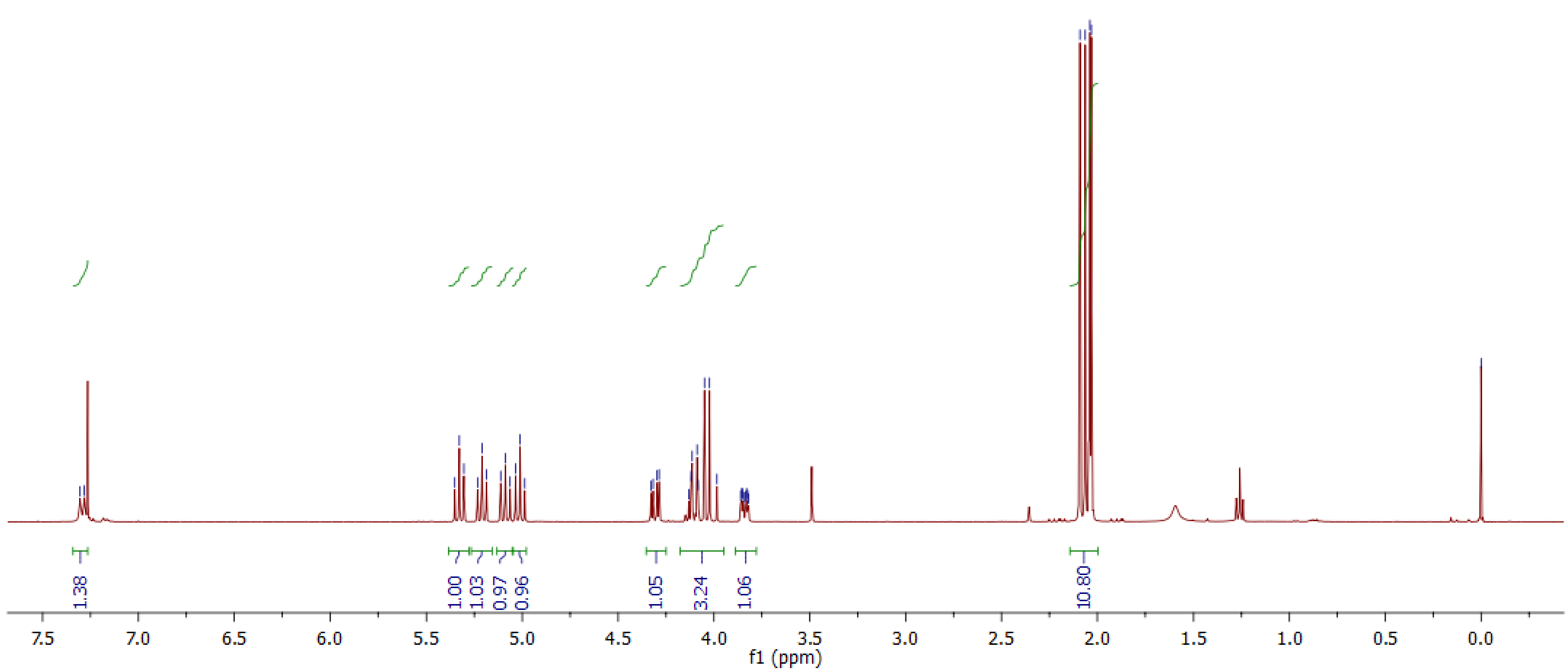

Fig. S53: ^{1}H NMR spectrum of compound **31**.

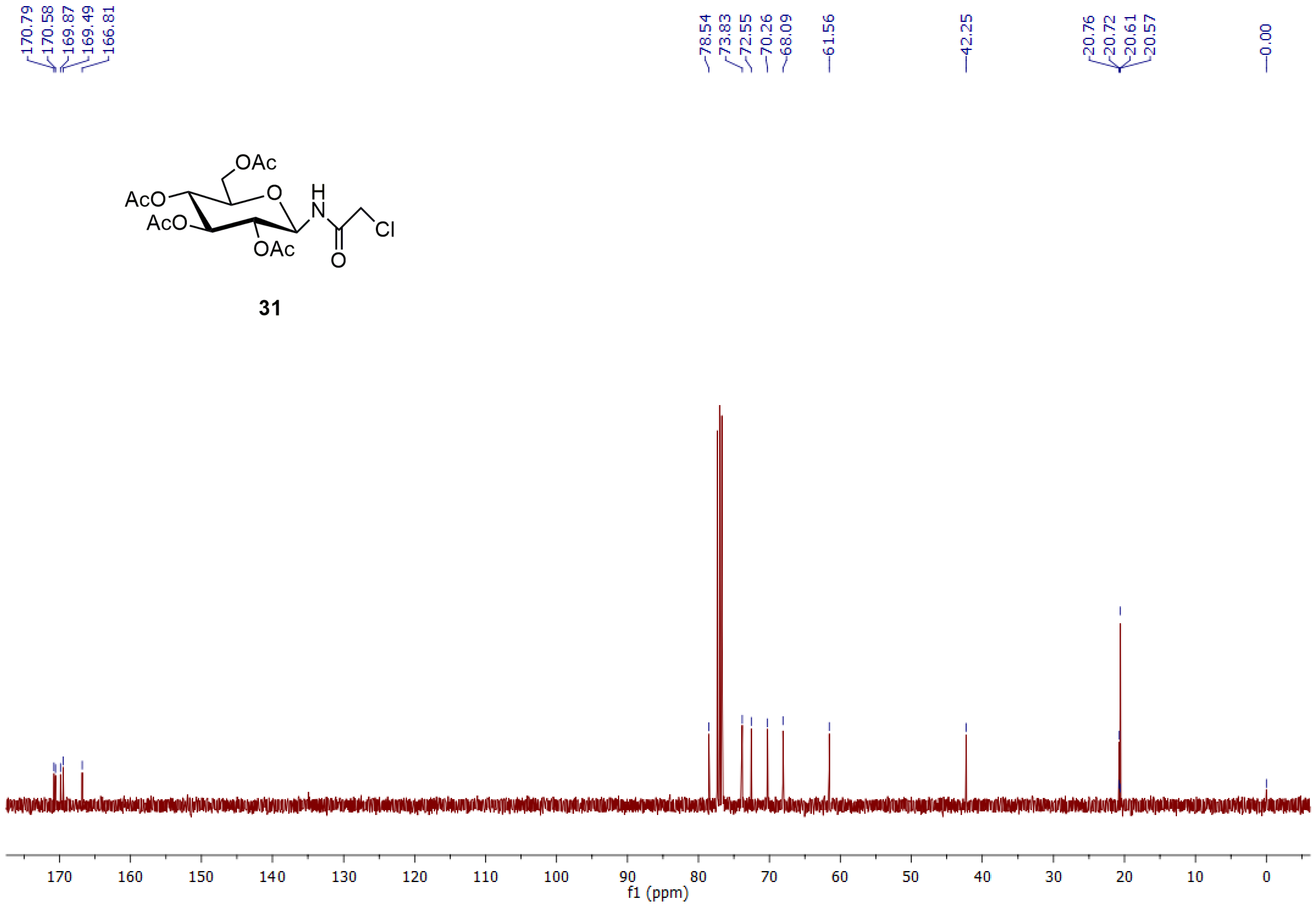

Fig. S54: ^{13}C NMR spectrum of compound **31**.

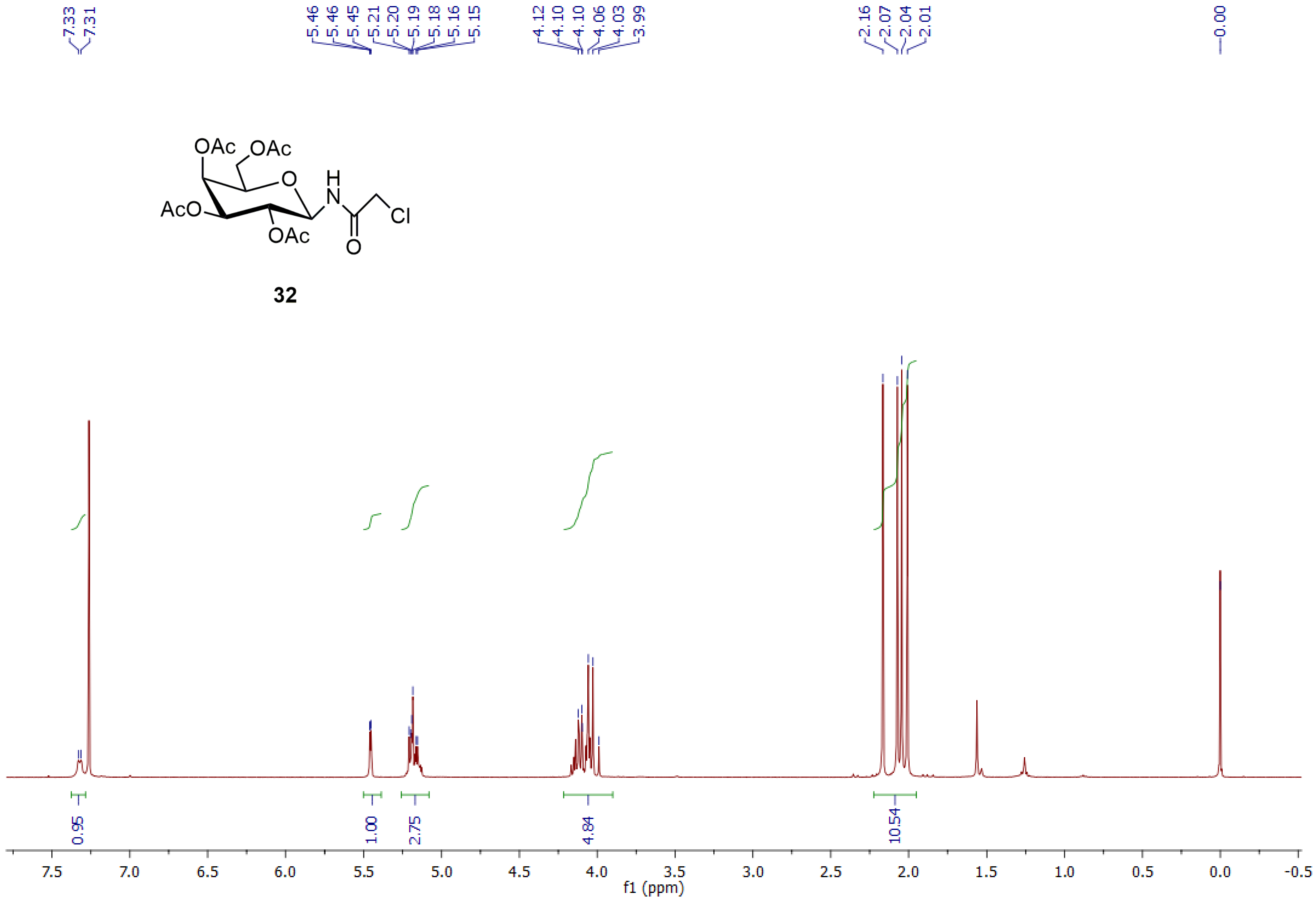

Fig. S55: ^{1}H NMR spectrum of compound **32**.

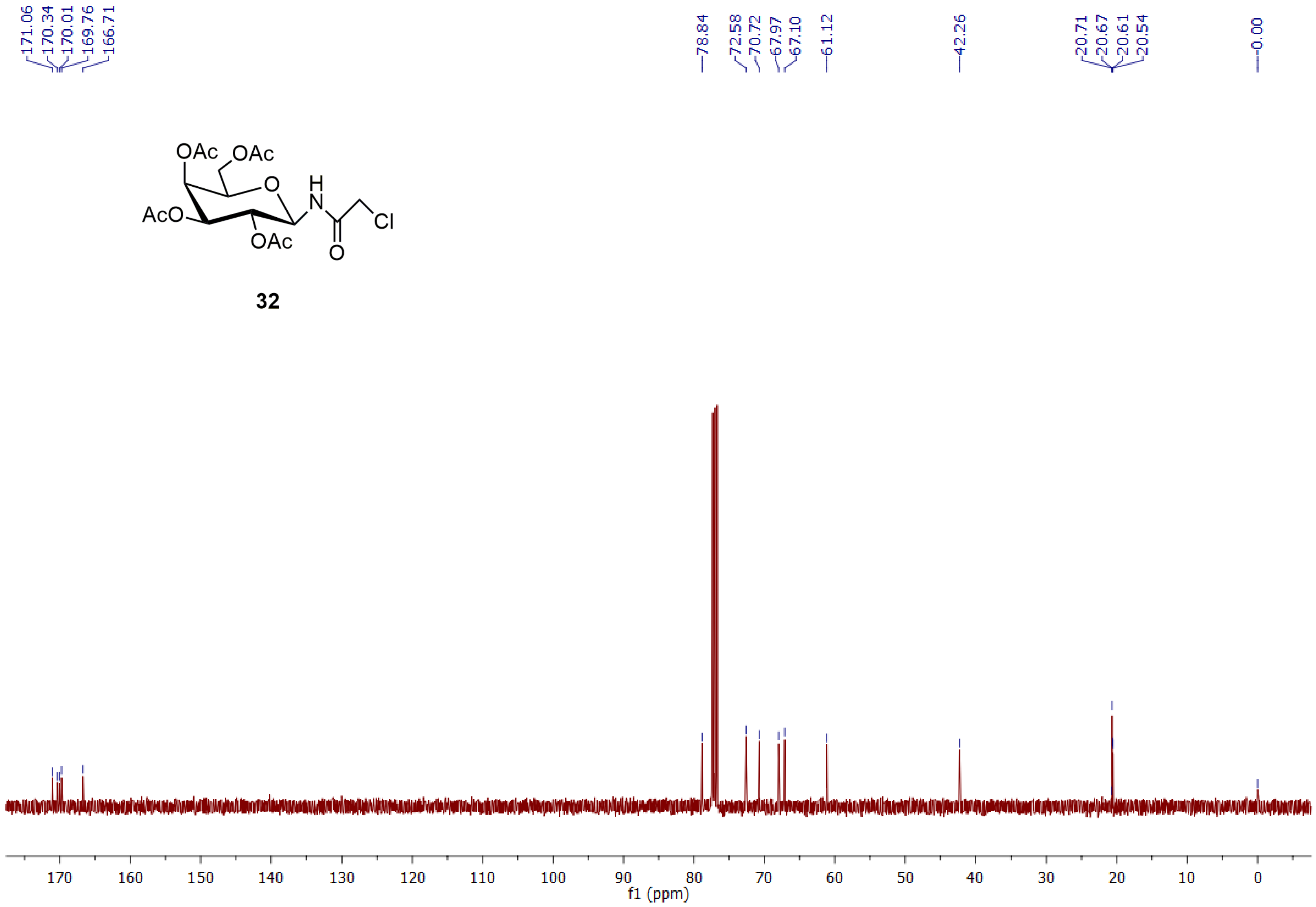

32

Fig. S56: ^{13}C NMR spectrum of compound **32**.

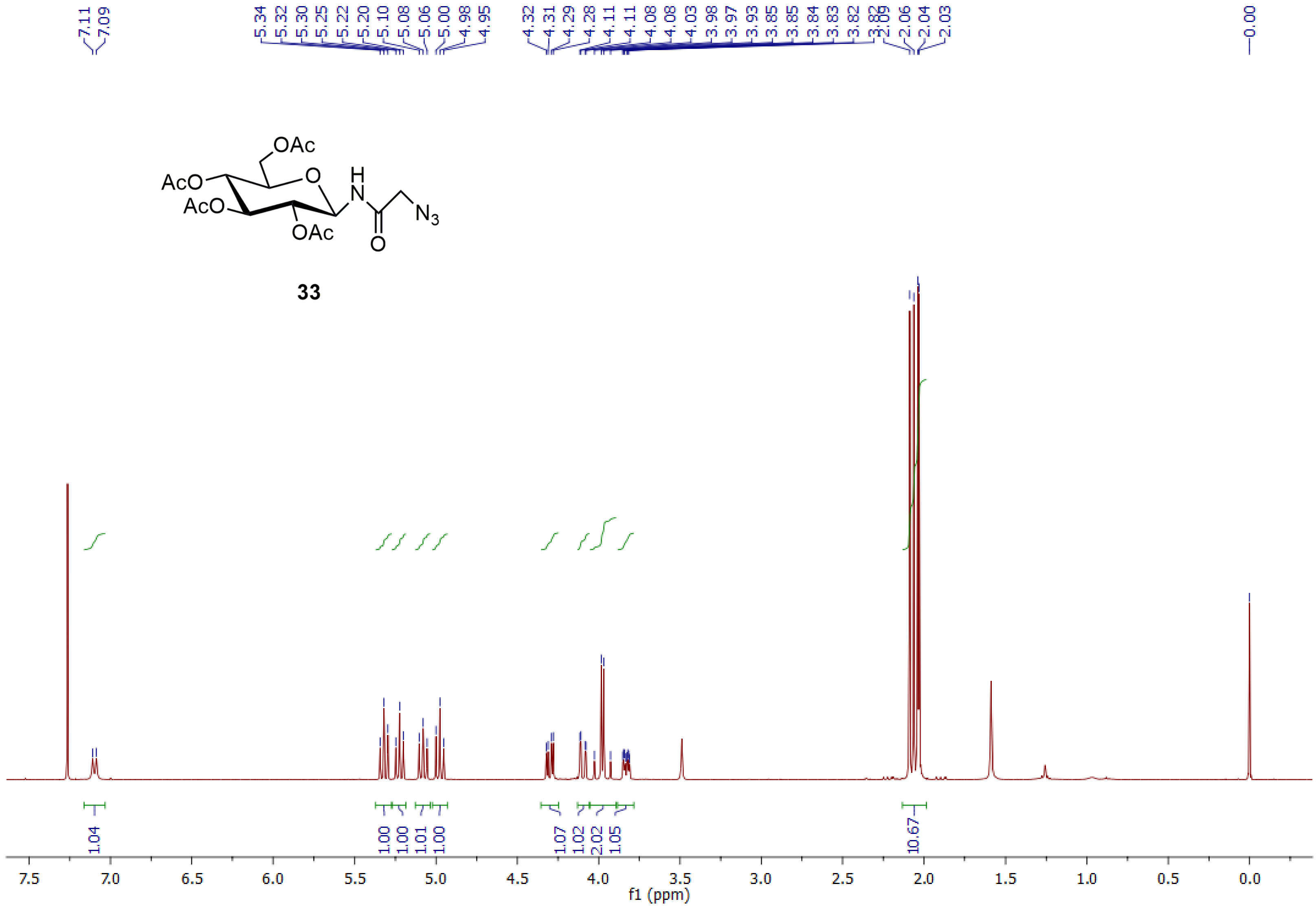

Fig. S57: ^{1}H NMR spectrum of compound **33**.

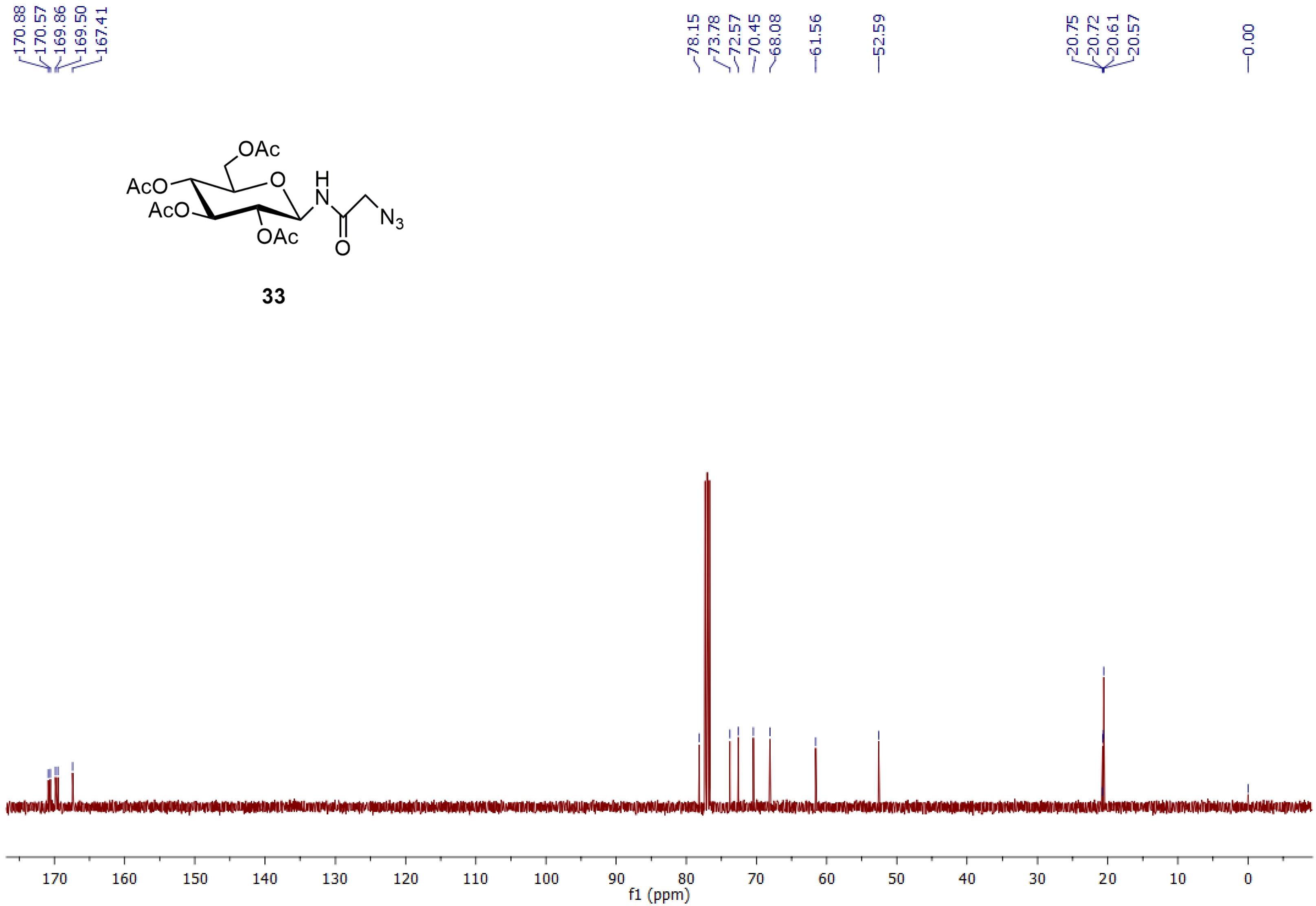

Fig. S58: ^{13}C NMR spectrum of compound **33**.

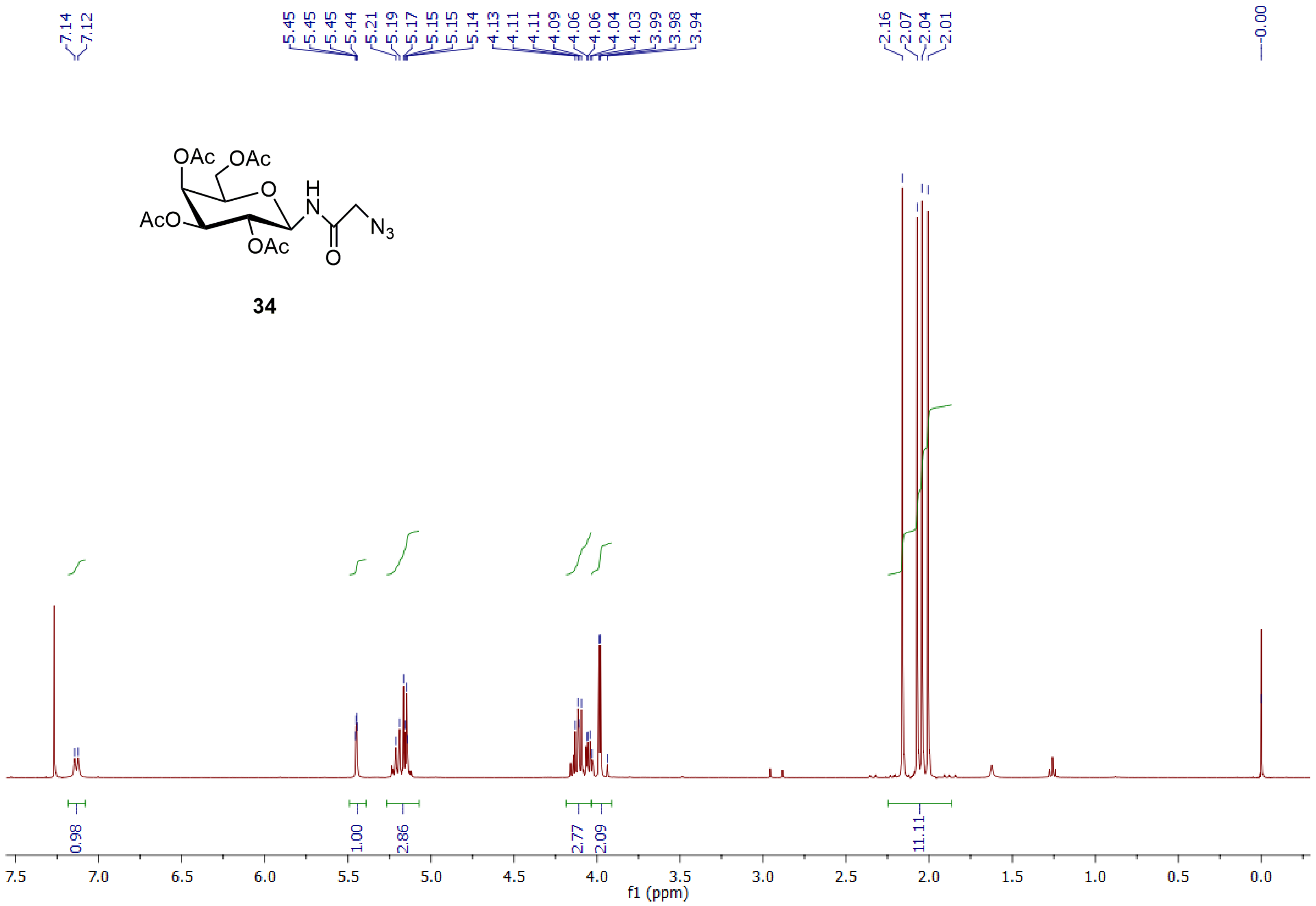

Fig. S59: ^{1}H NMR spectrum of compound **34**.

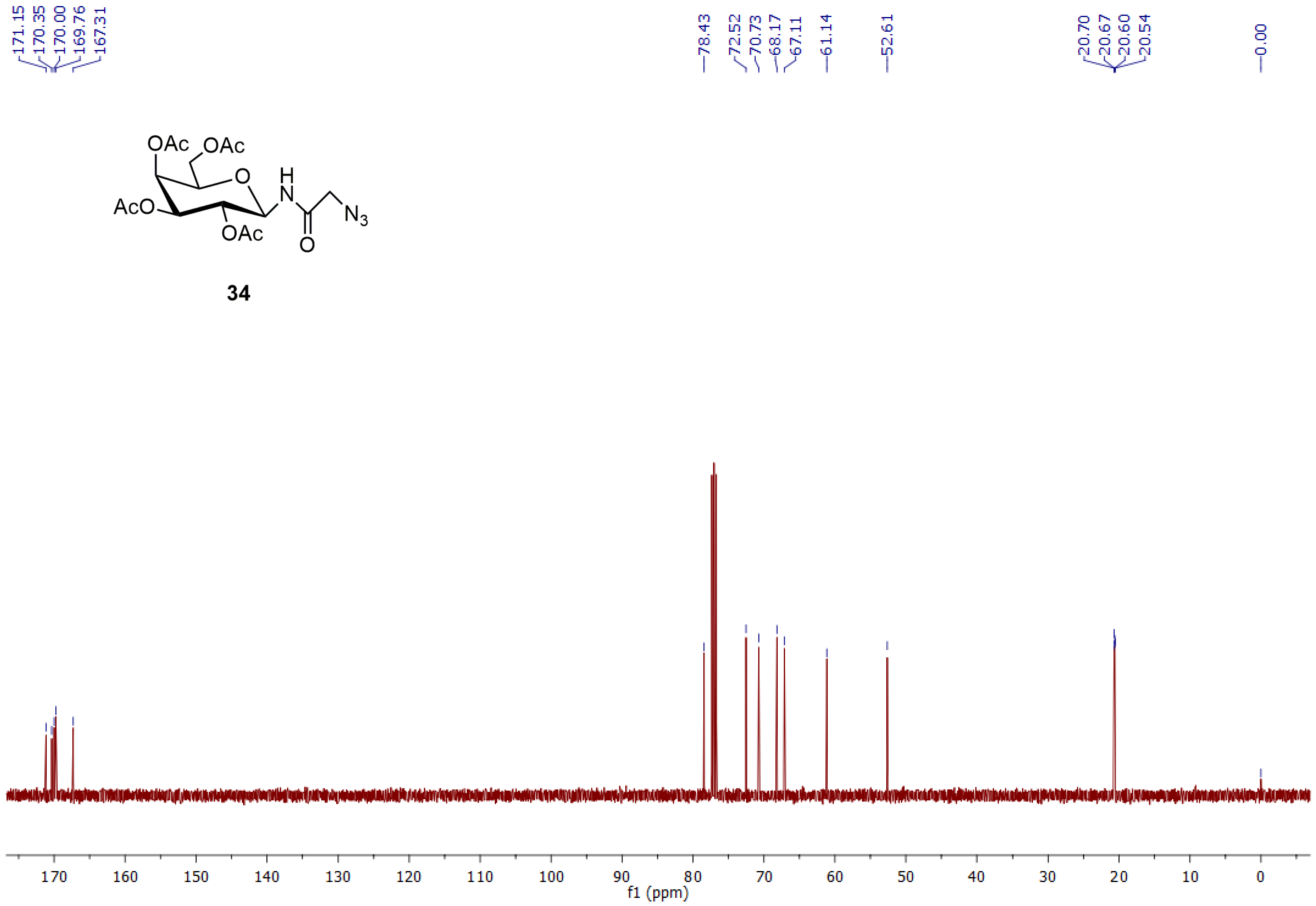

Fig. S60: ^{13}C NMR spectrum of compound **34**.

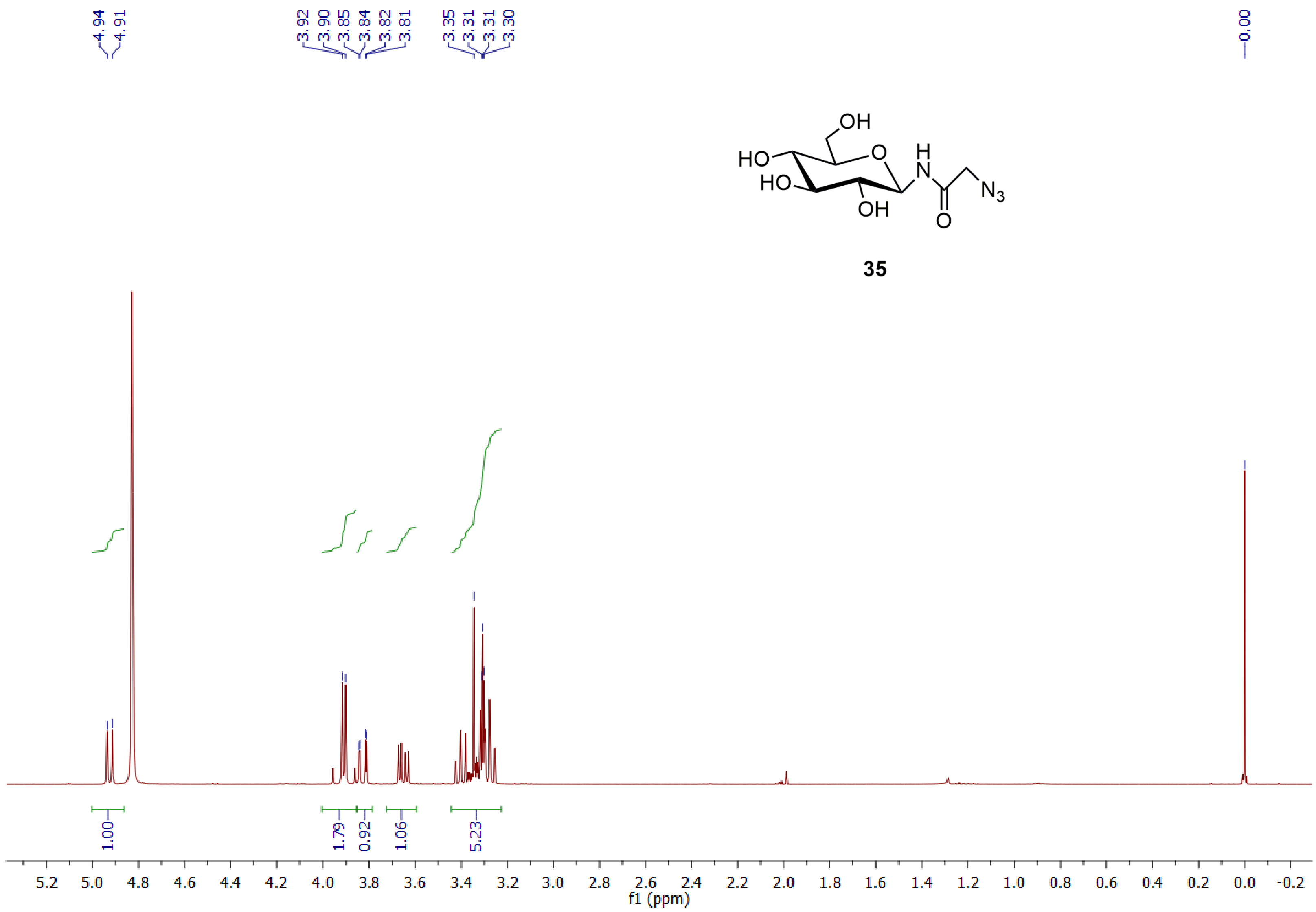

Fig. S61: ^{1}H NMR spectrum of compound **35**.

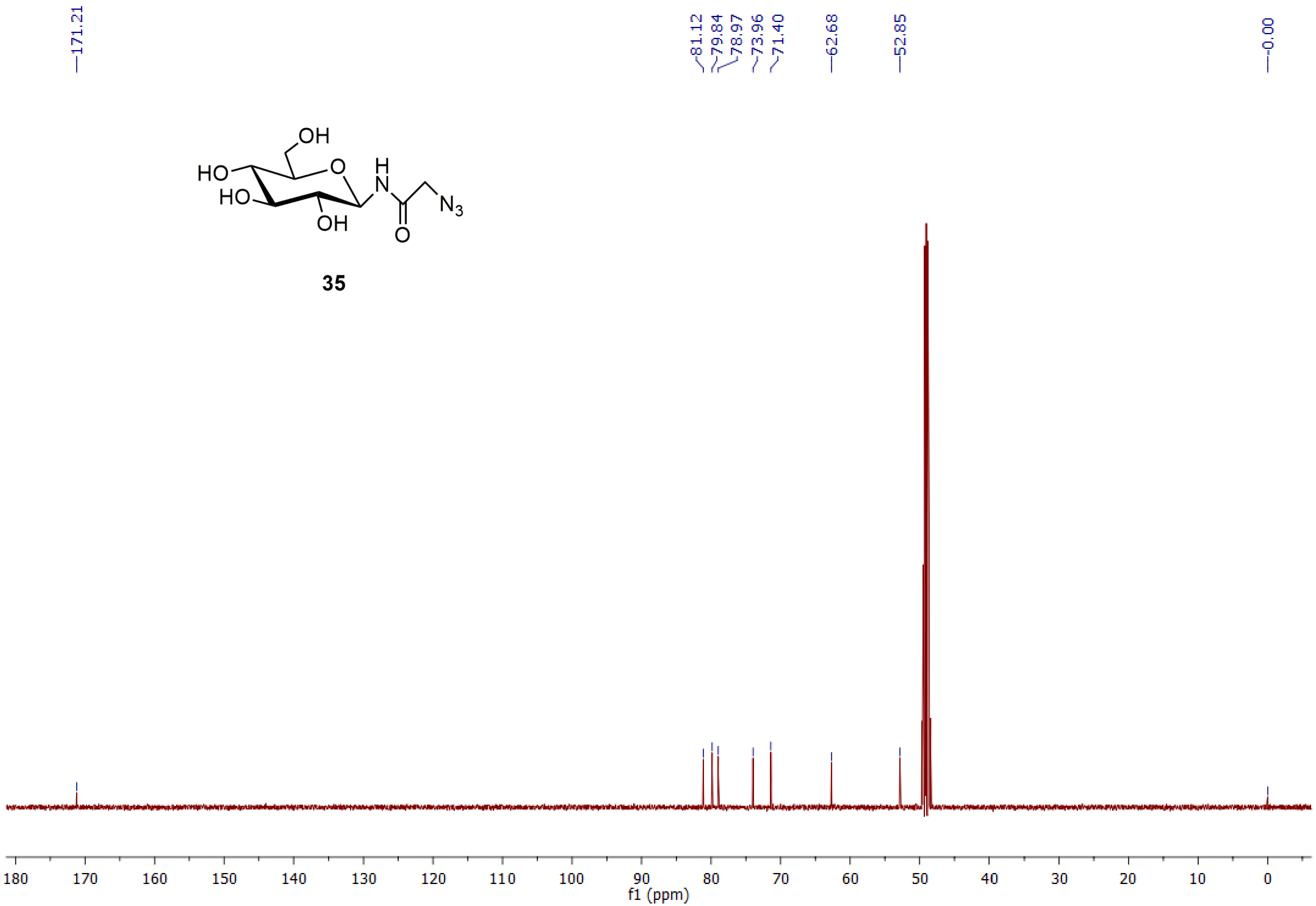

Fig. S62: ^{13}C NMR spectrum of compound **35**.

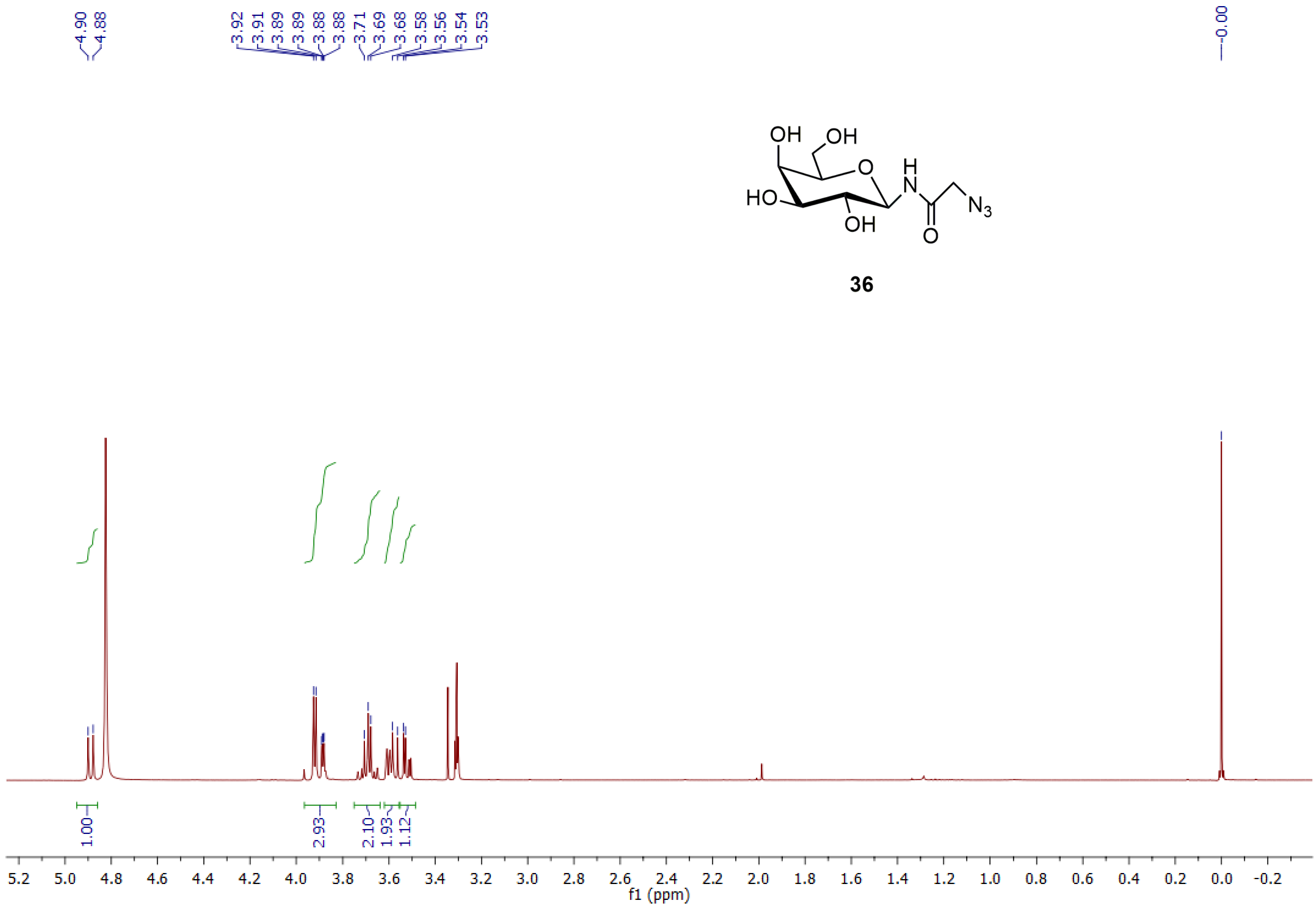

Fig. S63: ^{1}H NMR spectrum of compound **36**.

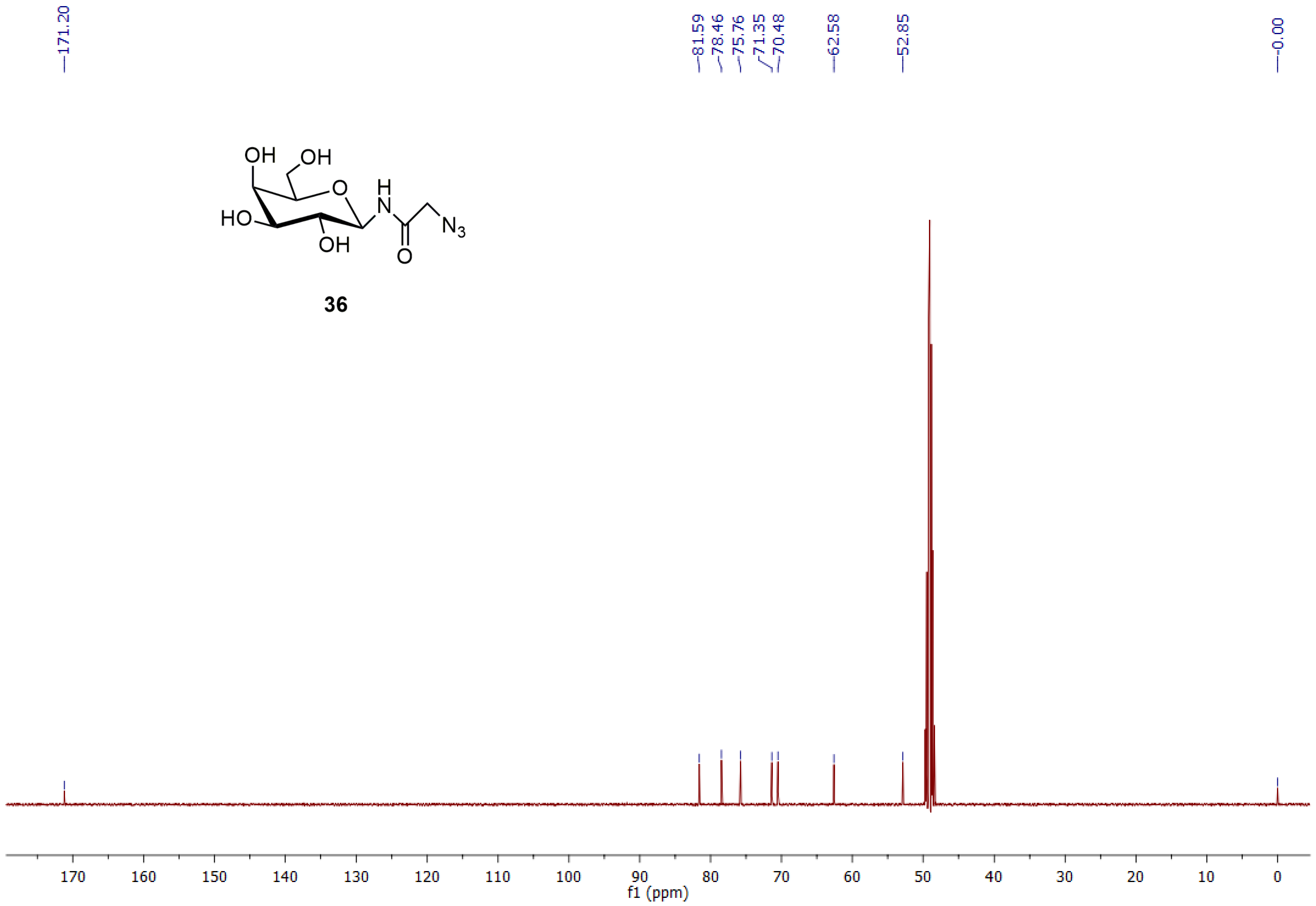

Fig. S64: ^{13}C NMR spectrum of compound **36**.

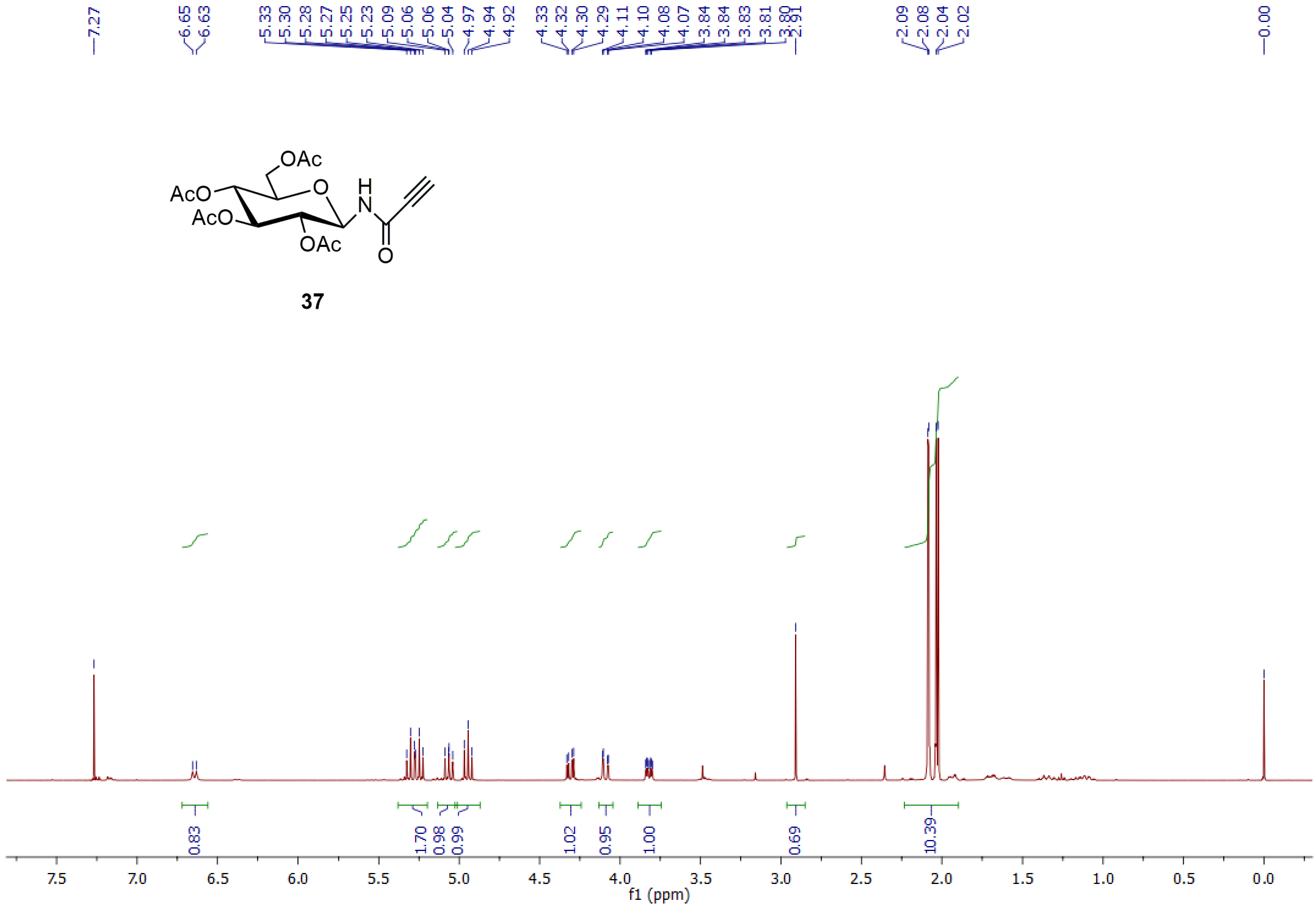

Fig. S65: ^{1}H NMR spectrum of compound **37**.

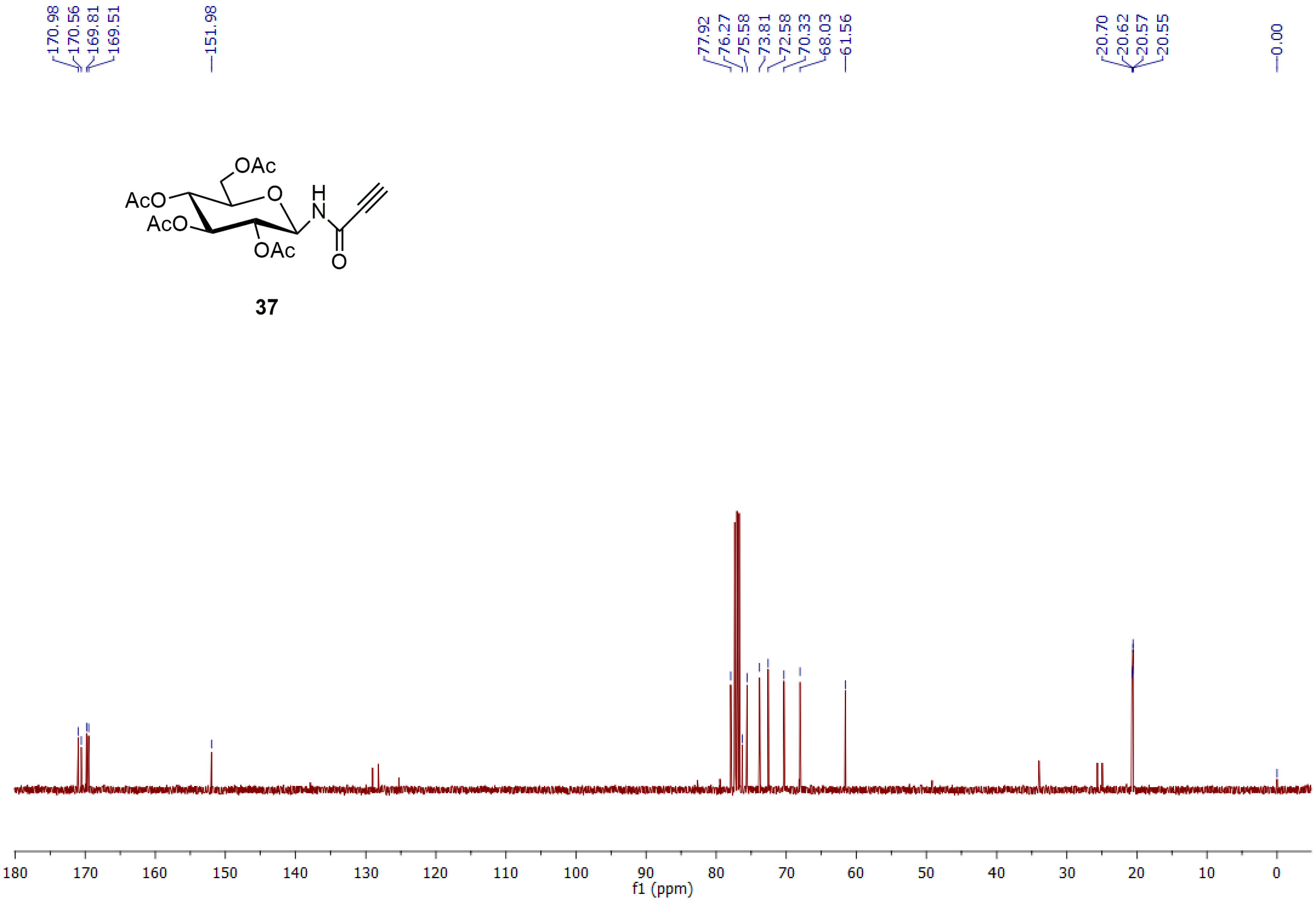

Fig. S66: ^{13}C NMR spectrum of compound **37**.

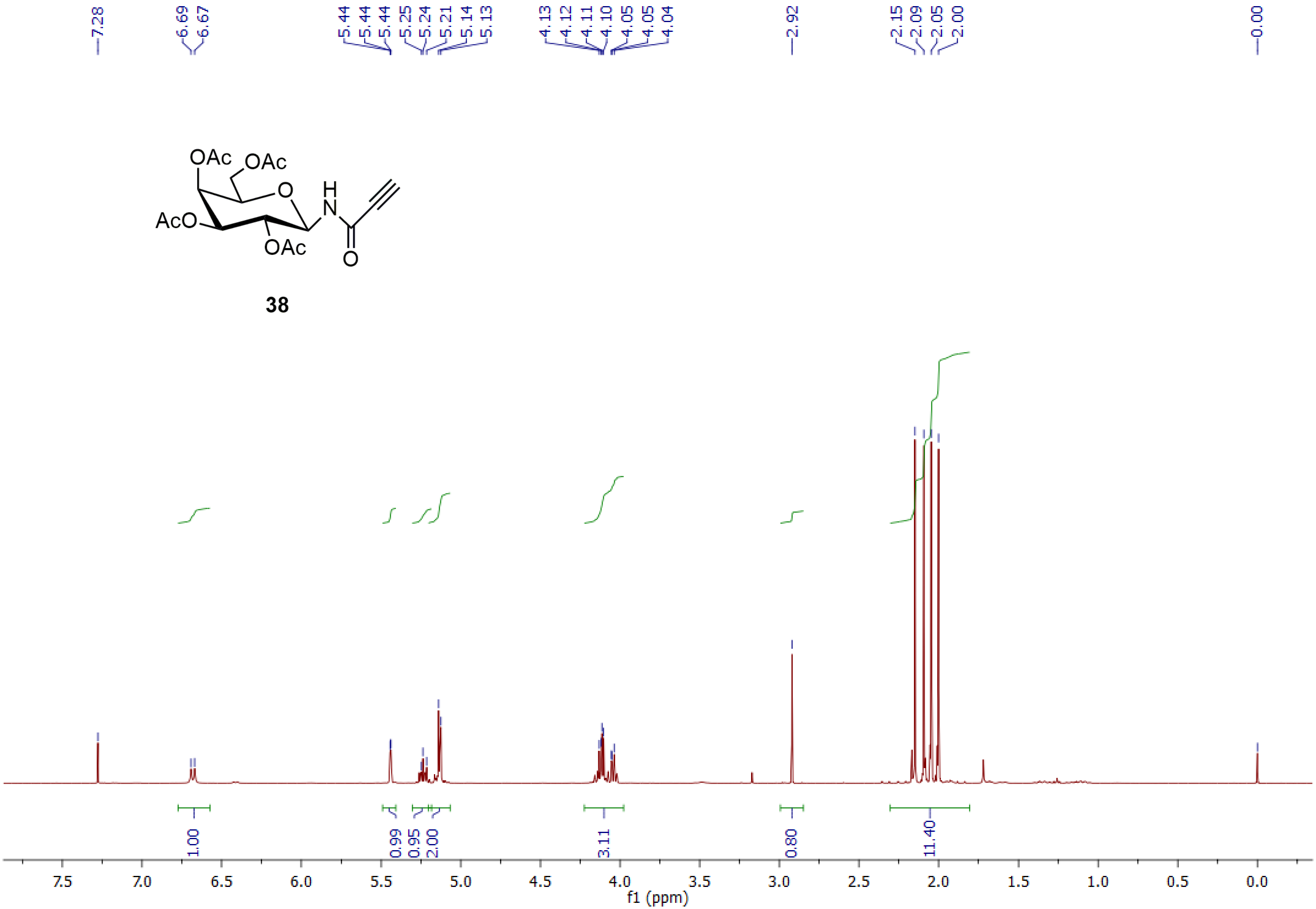

Fig. S67: ^{1}H NMR spectrum of compound **38**.

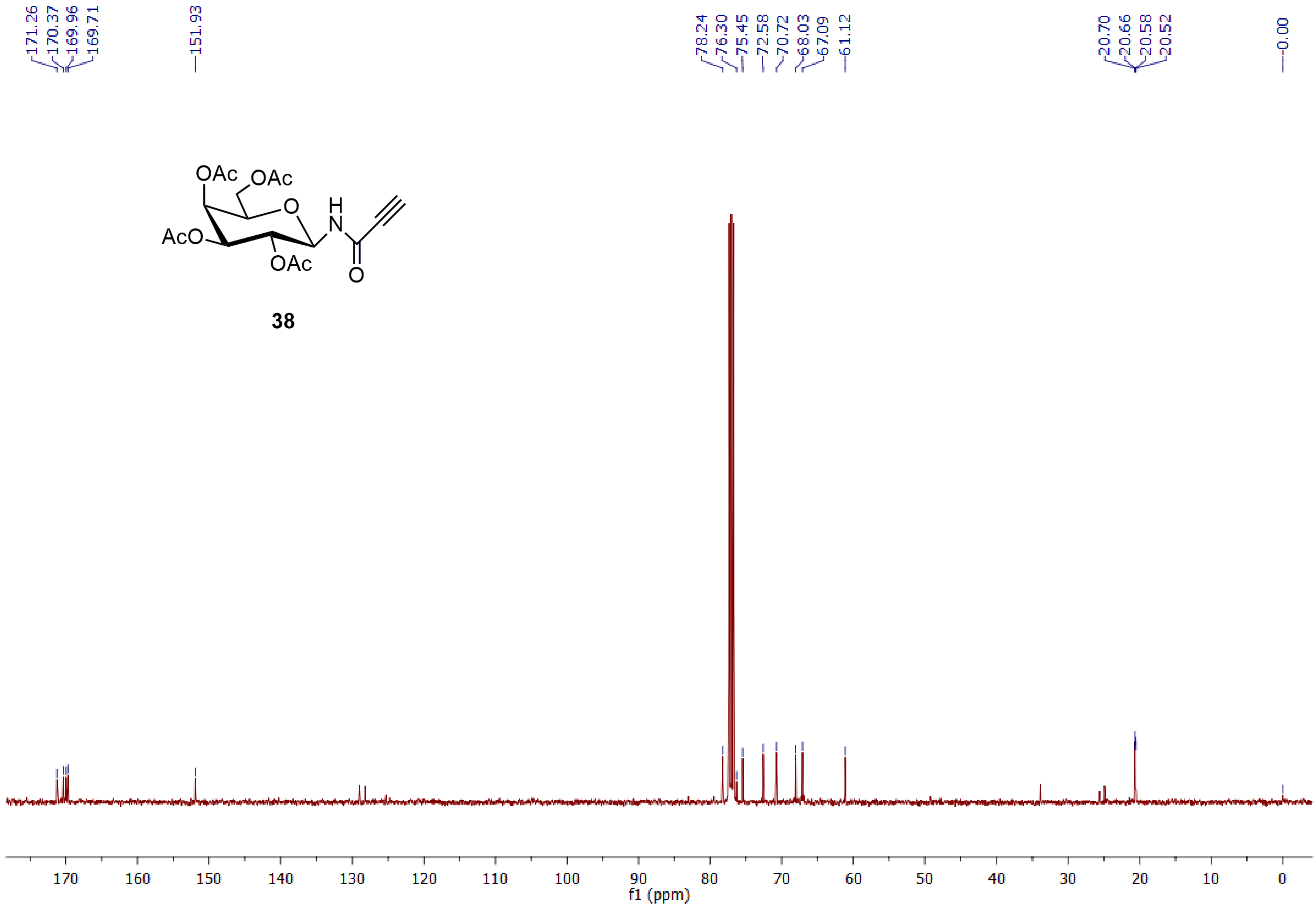

Fig. S68: ^{13}C NMR spectrum of compound **38**.

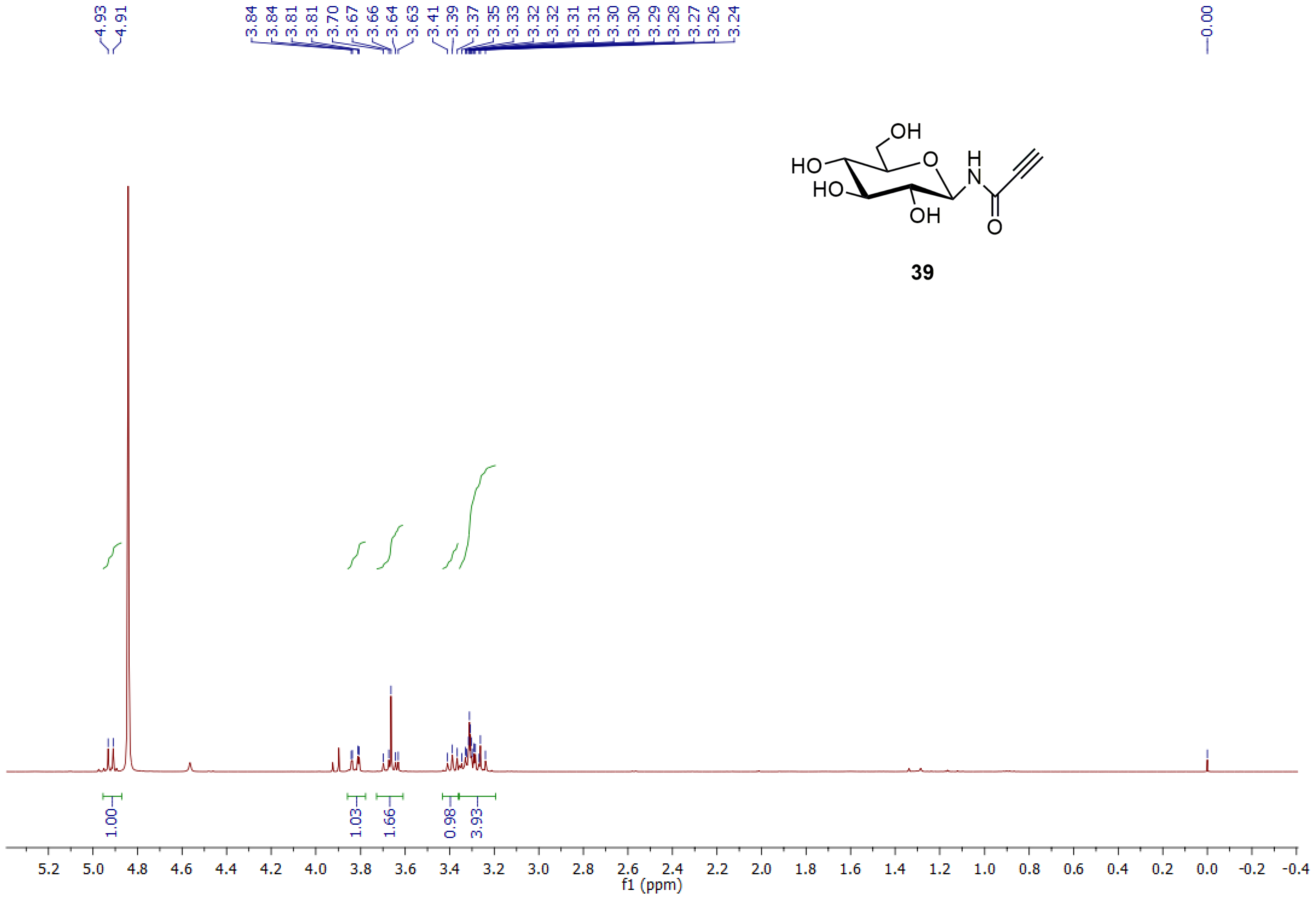

Fig. S69: ^{1}H NMR spectrum of compound **39**.

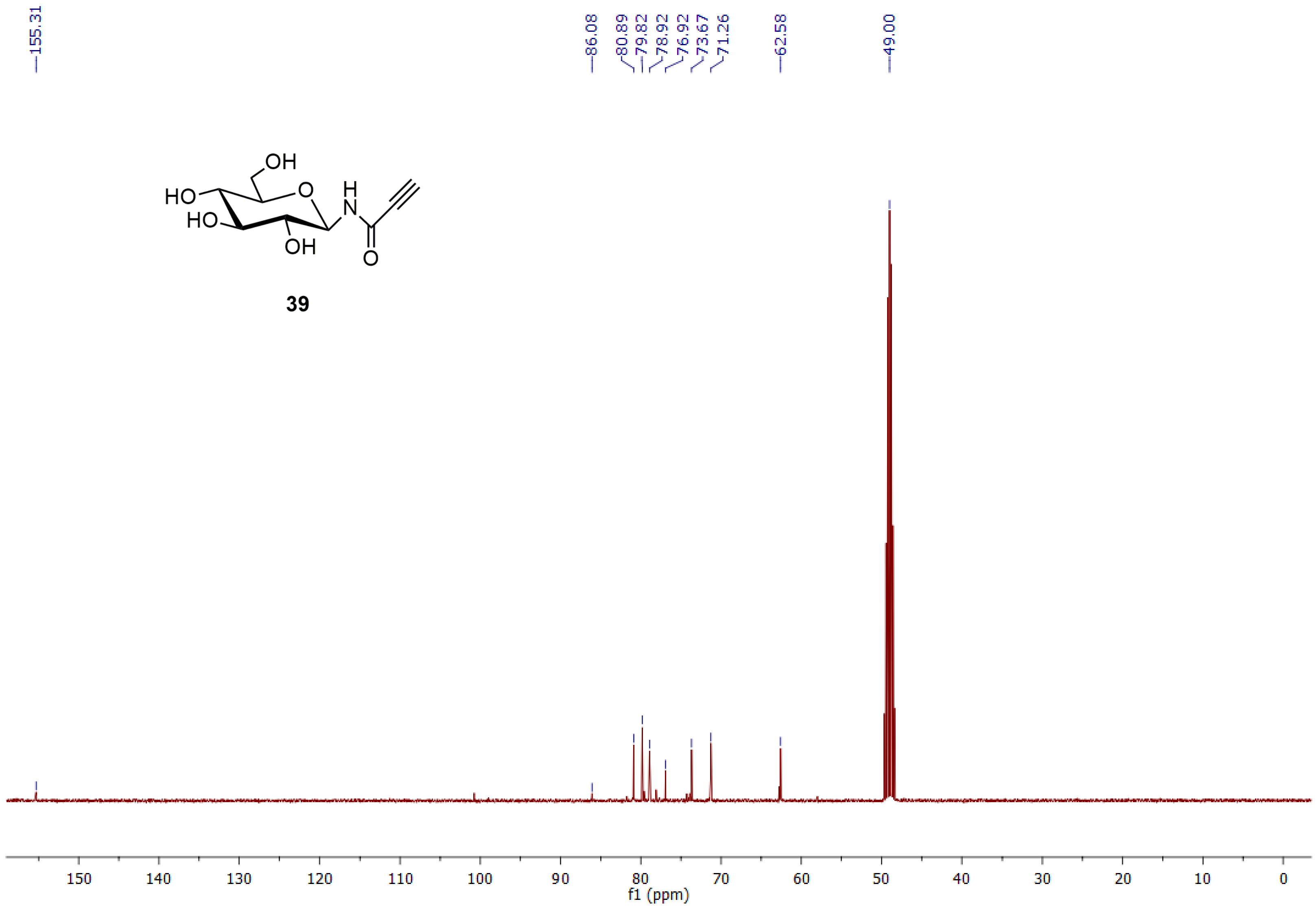

39

Fig. S70: ^{13}C NMR spectrum of compound **39**.

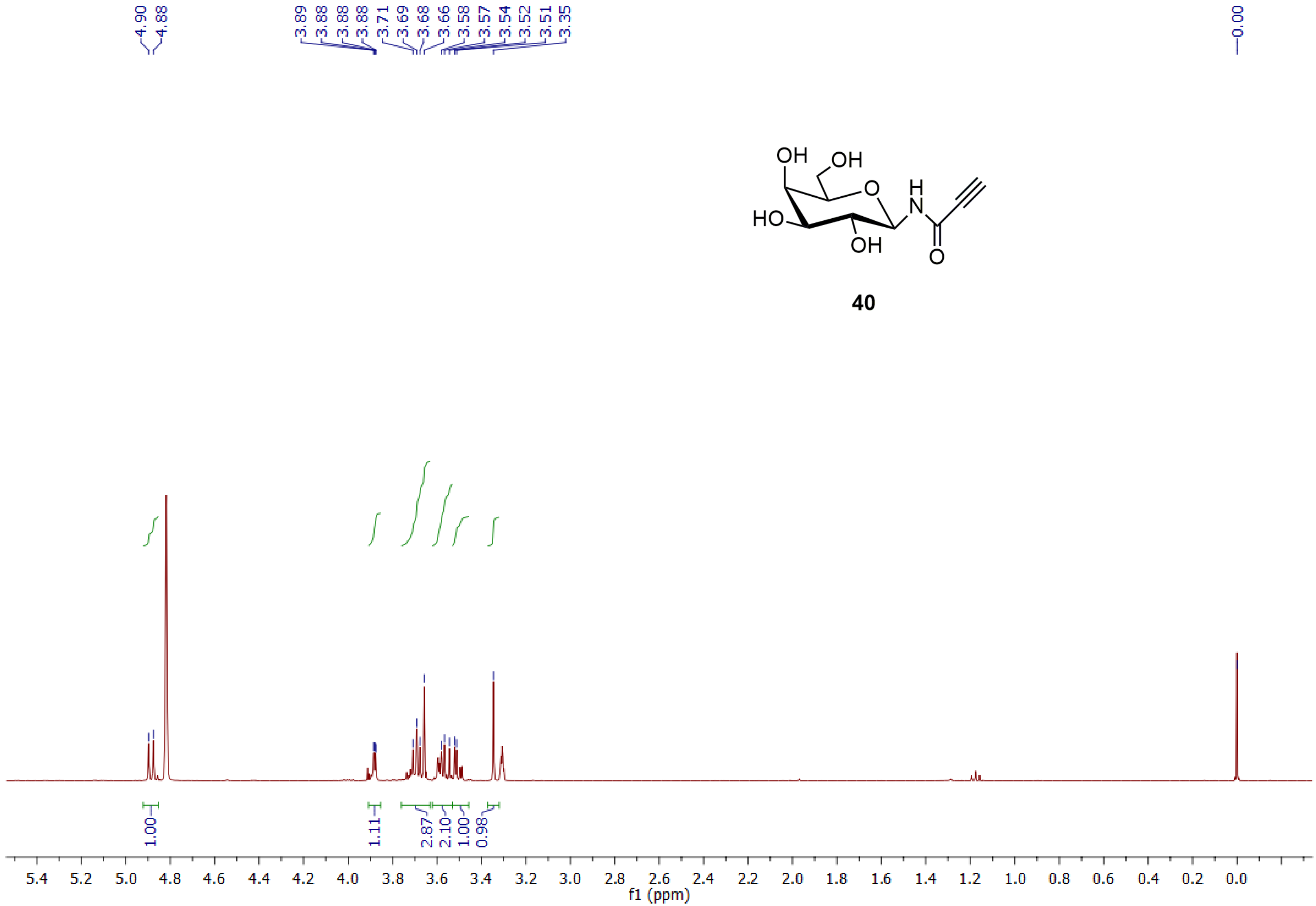

Fig. S71: ^{1}H NMR spectrum of compound **40**.

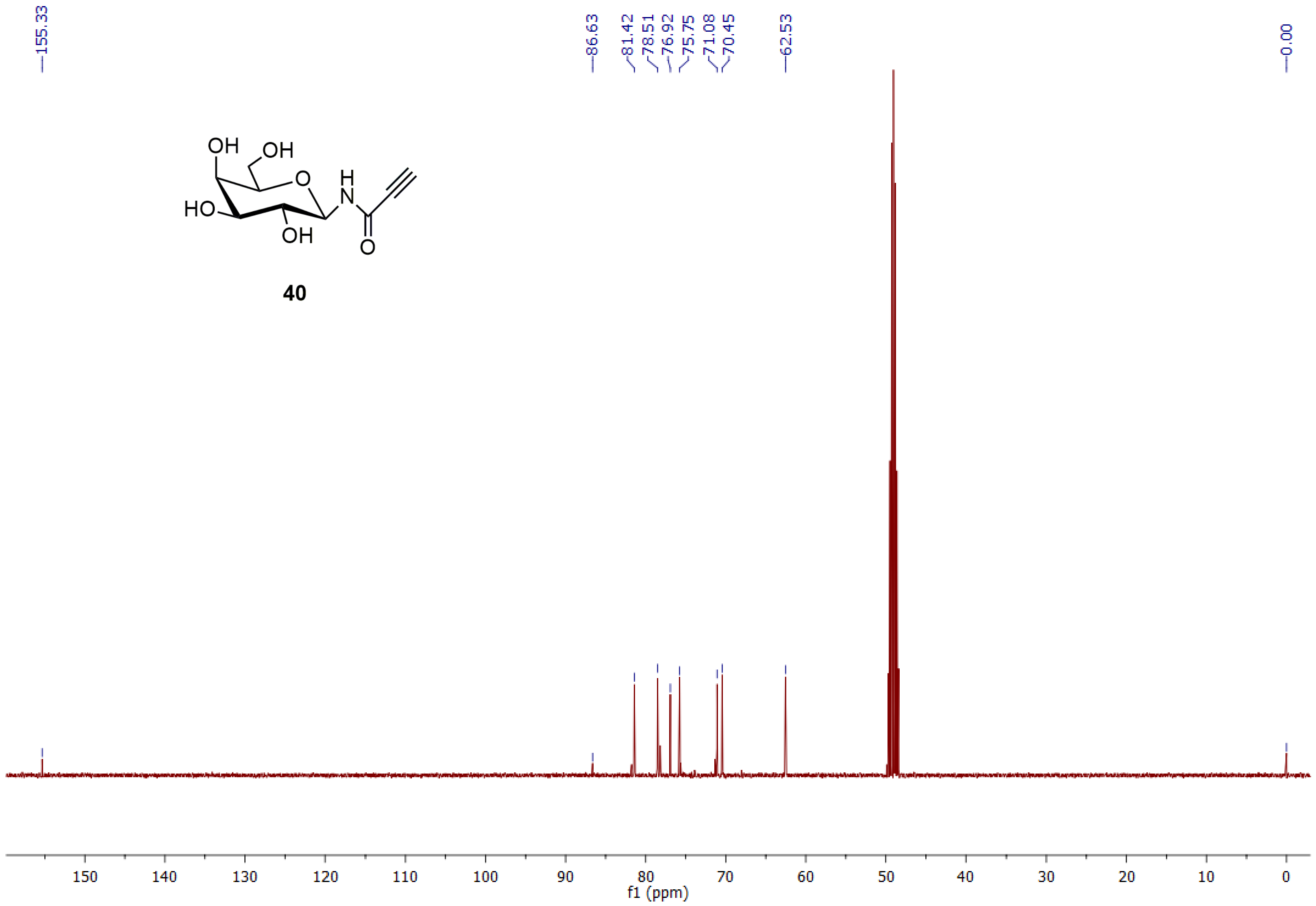

Fig. S72: ^{13}C NMR spectrum of compound **40**.

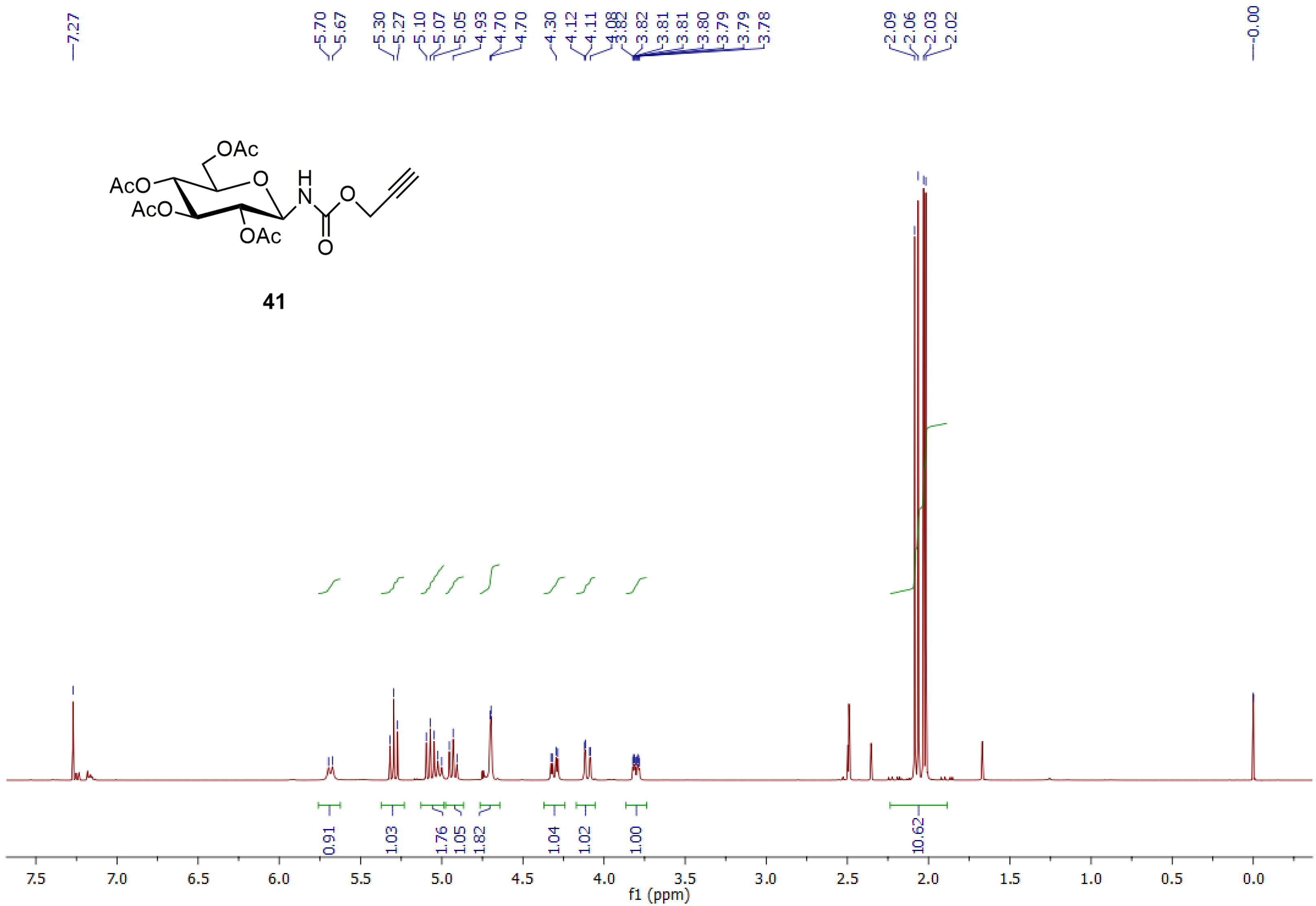

Fig. S73: ^{1}H NMR spectrum of compound **41**.

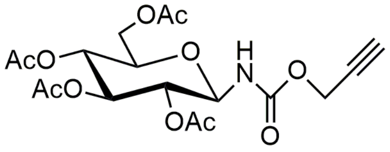

41

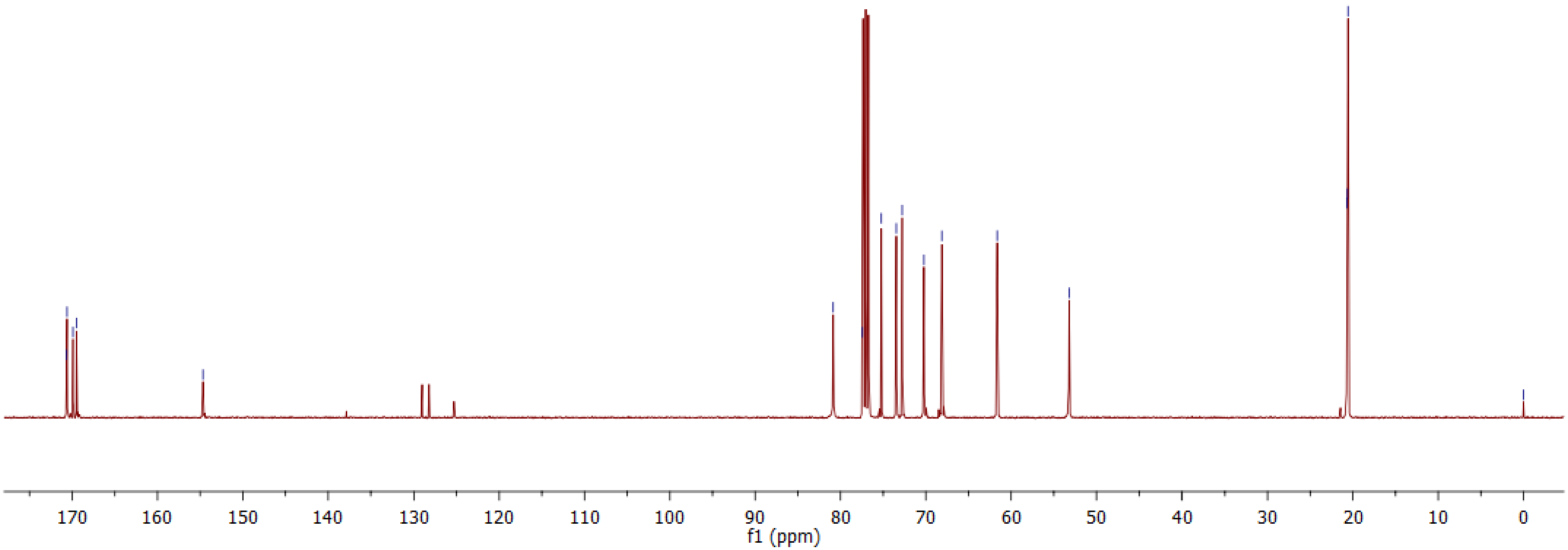

Fig. S74: ^{13}C NMR spectrum of compound **41**.

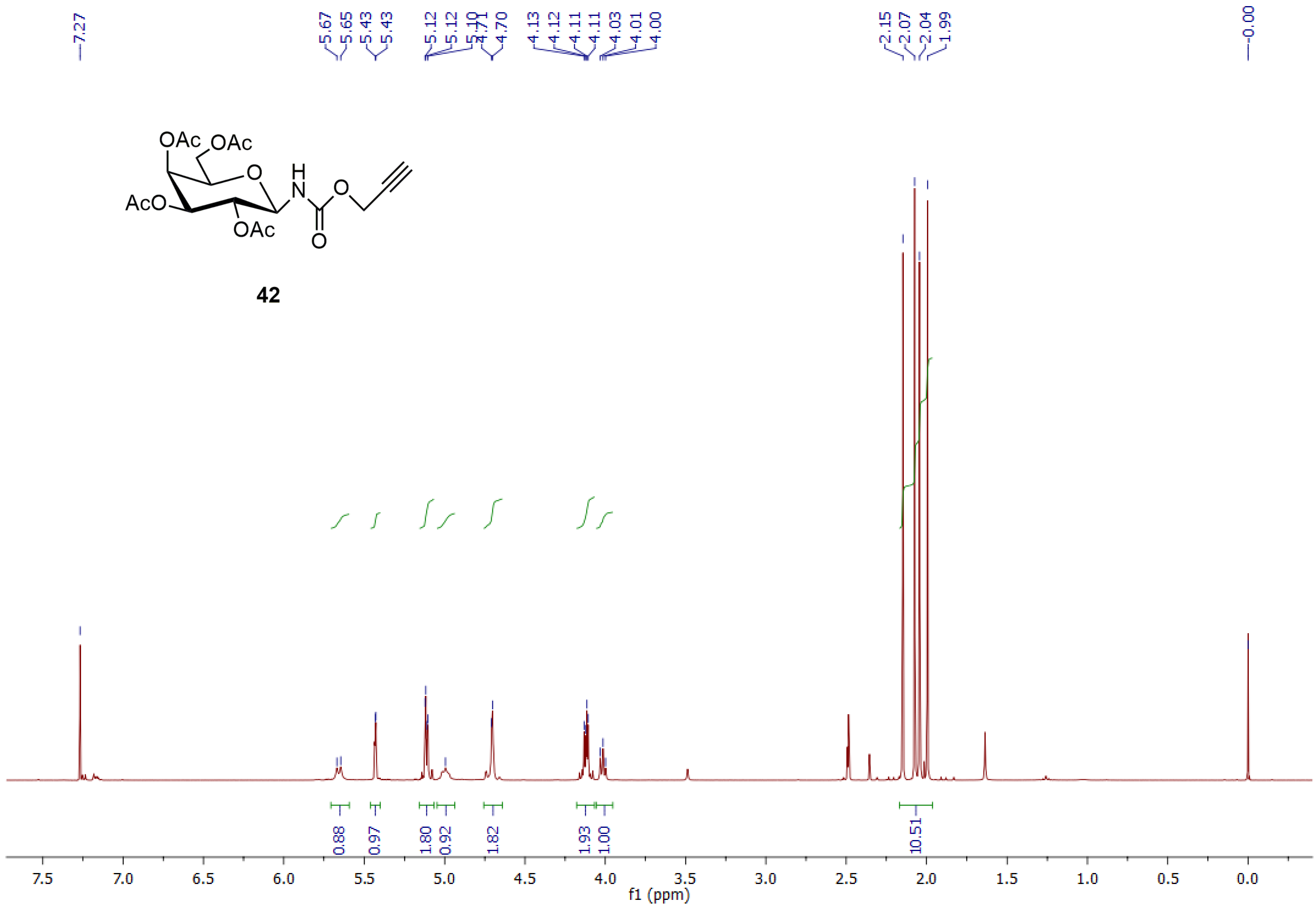

Fig. S75: ^{1}H NMR spectrum of compound **42**.

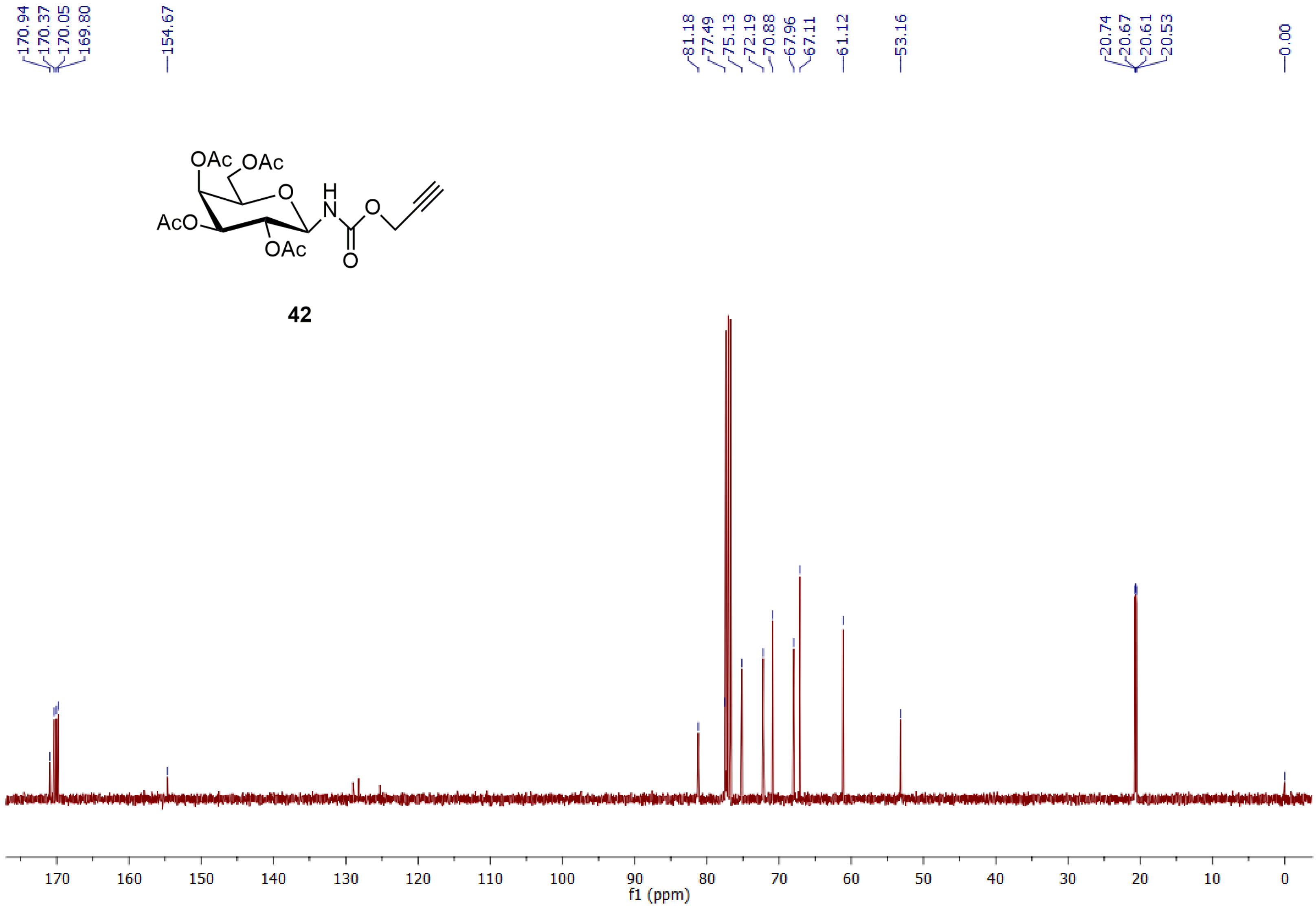

Fig. S76: ^{13}C NMR spectrum of compound **42**.

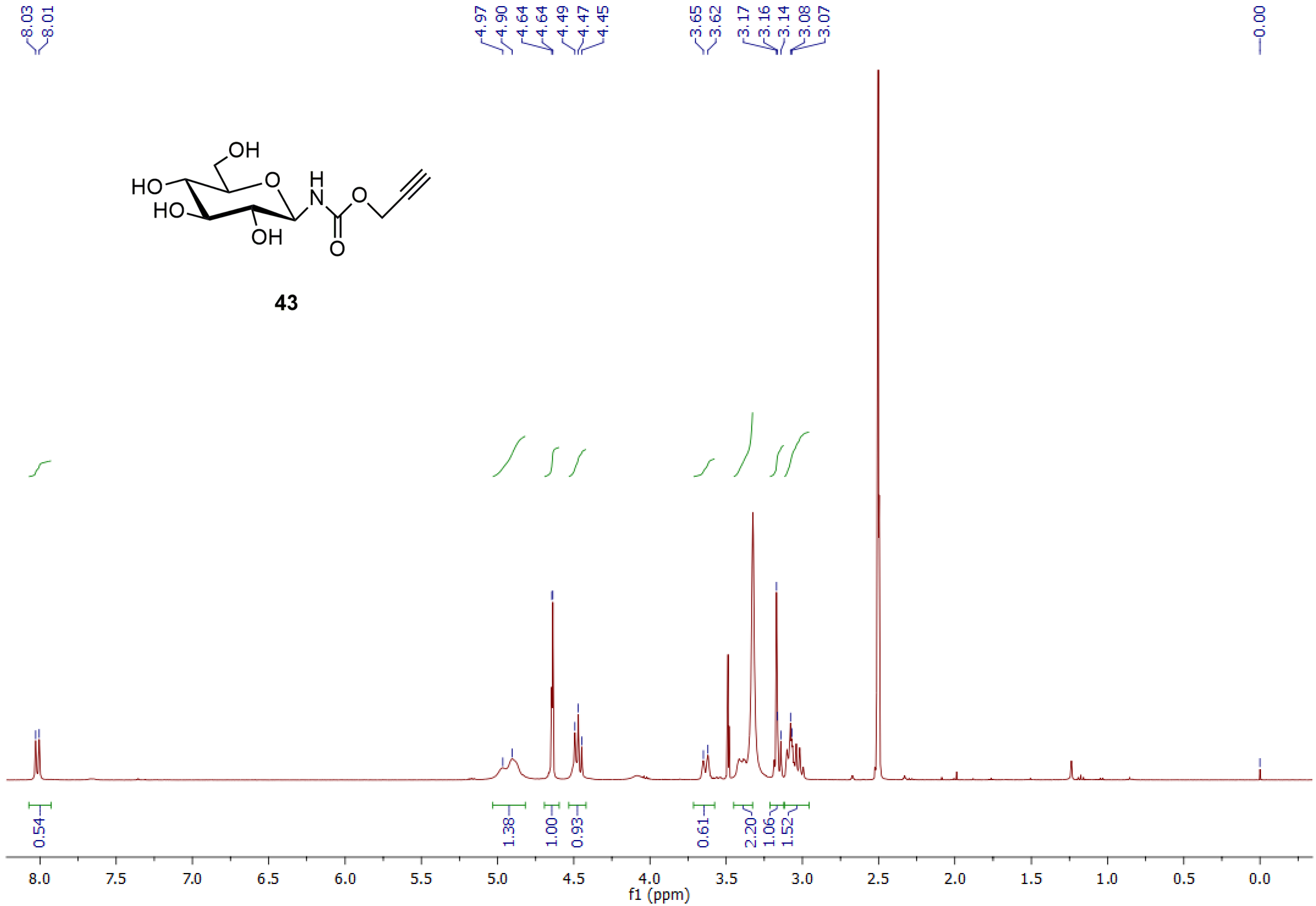

Fig. S77: ^{1}H NMR spectrum of compound **43**.

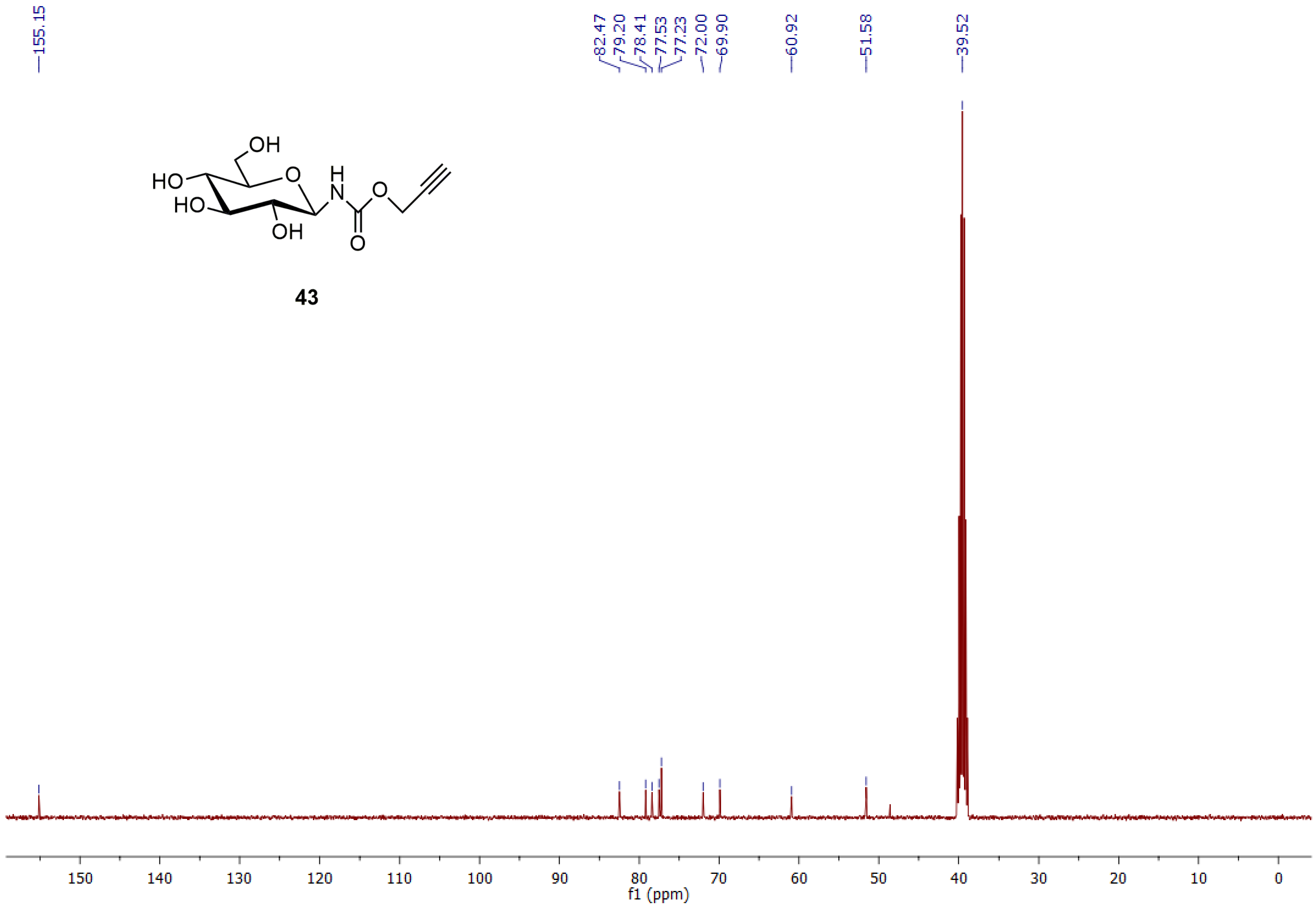

Fig. S78: ^{13}C NMR spectrum of compound **43**.

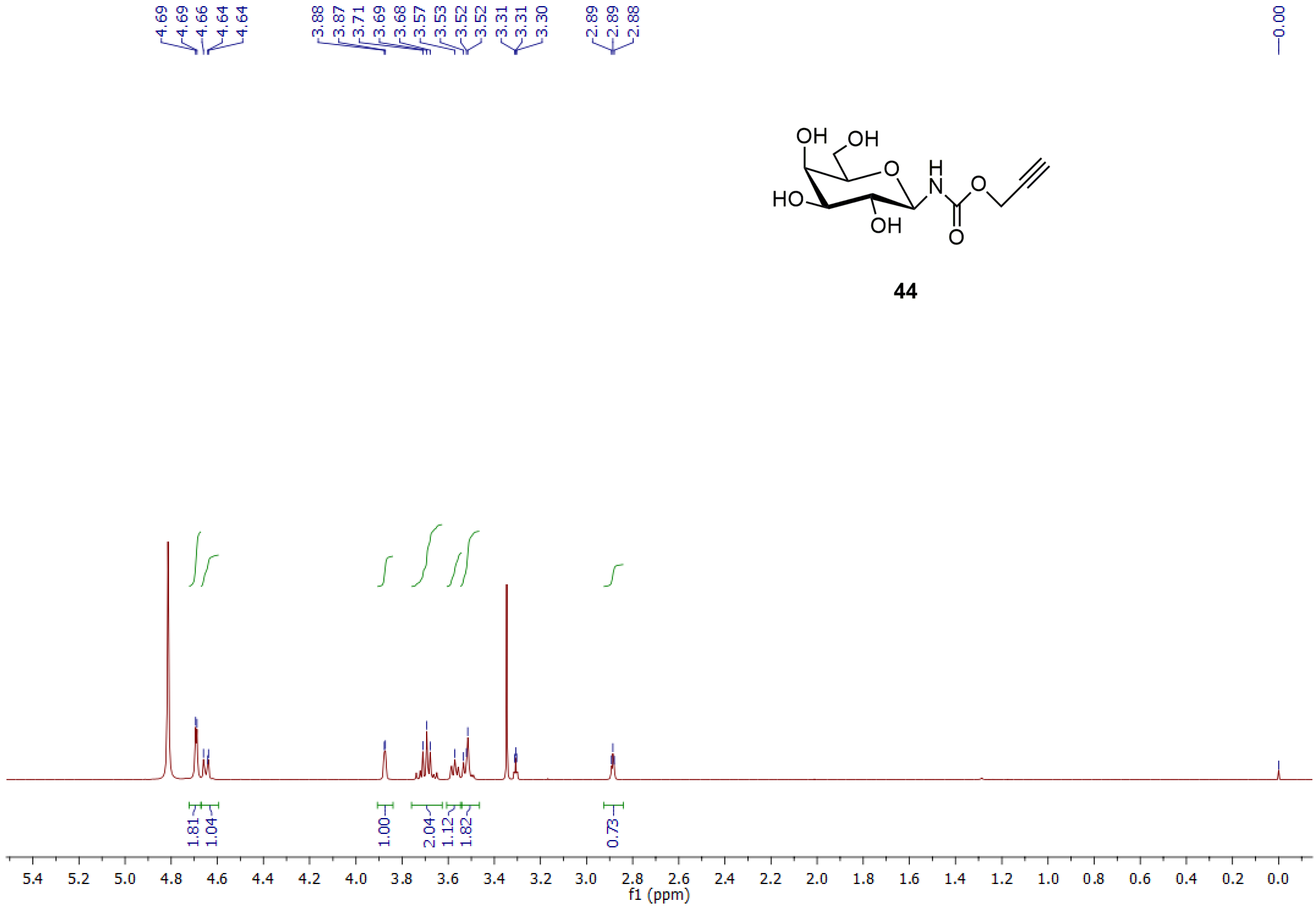

44

Fig. S79: ^{1}H NMR spectrum of compound **44**.

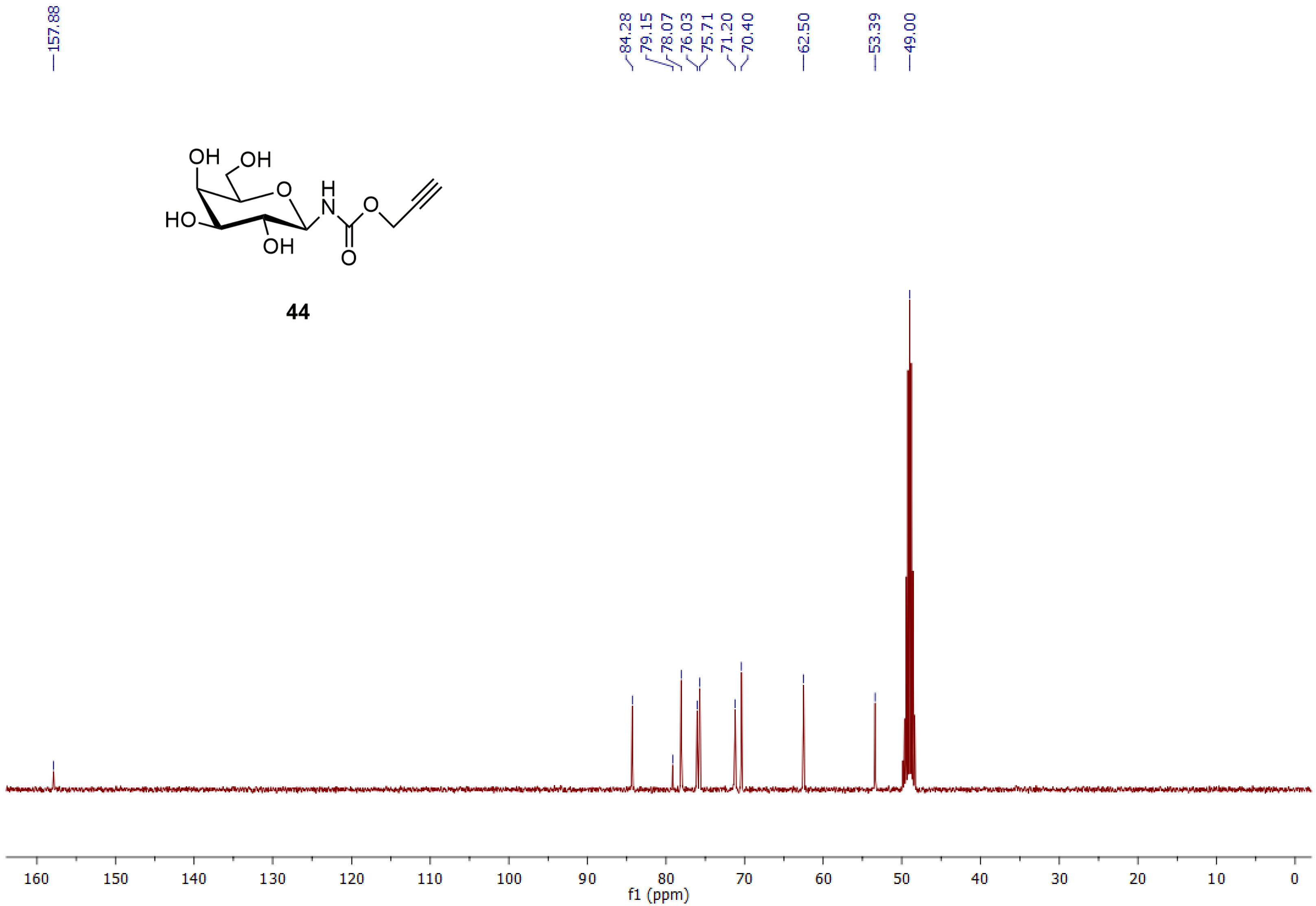

Fig. S80: ^{13}C NMR spectrum of compound **44**.

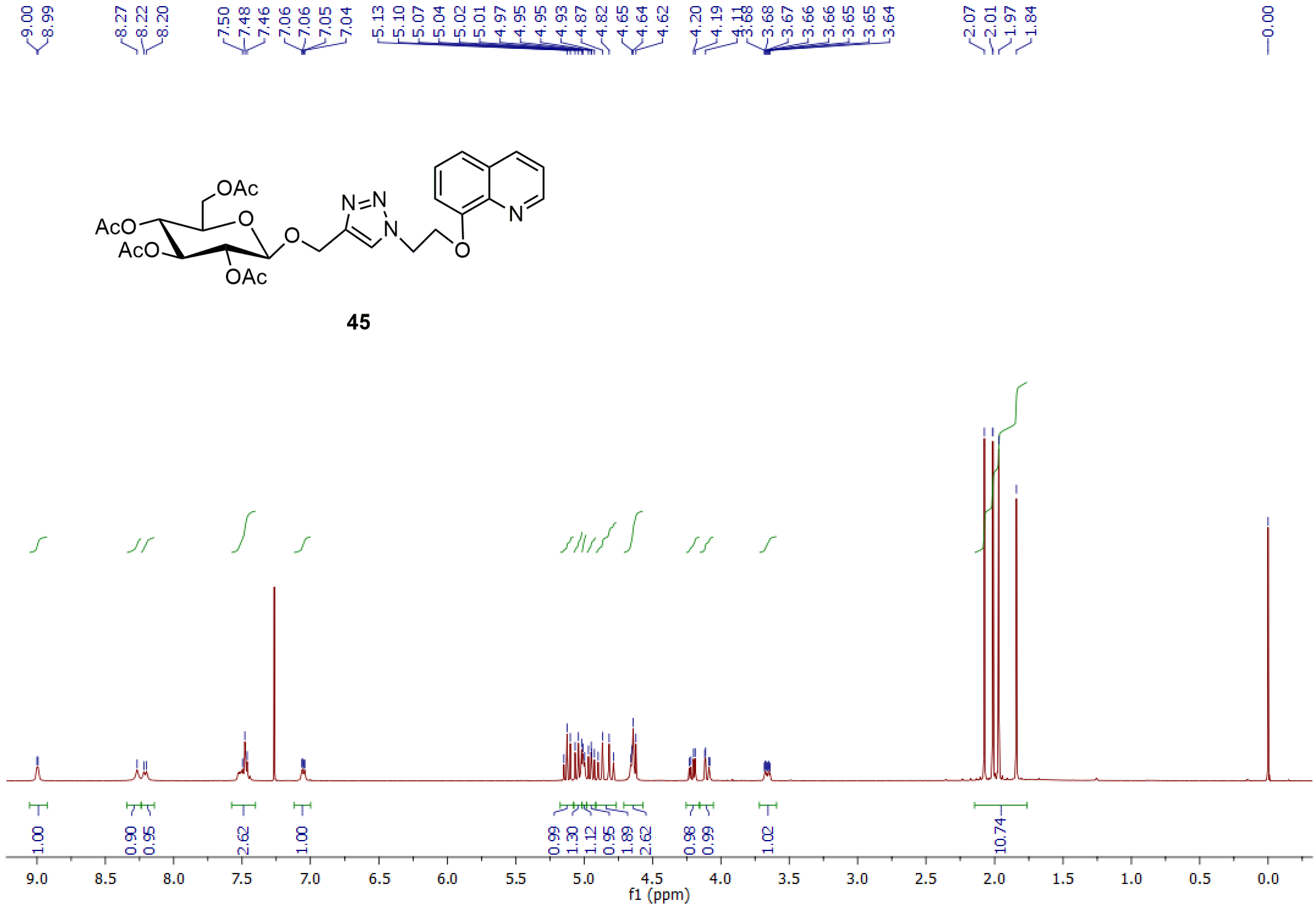

45

Fig. S81: ^{1}H NMR spectrum of glycoconjugate **45**.

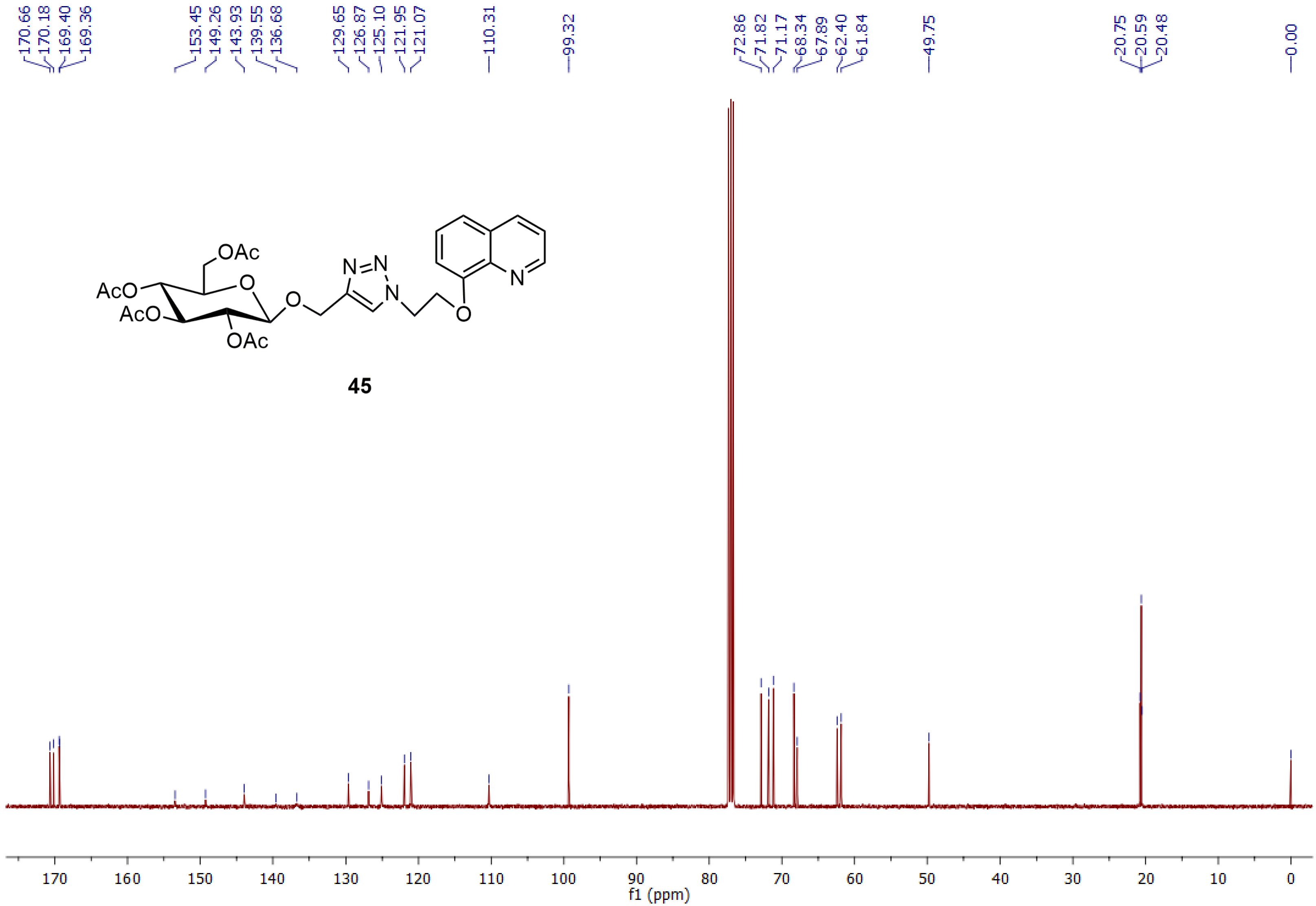

Fig. S82: ^{13}C NMR spectrum of glycoconjugate **45**.

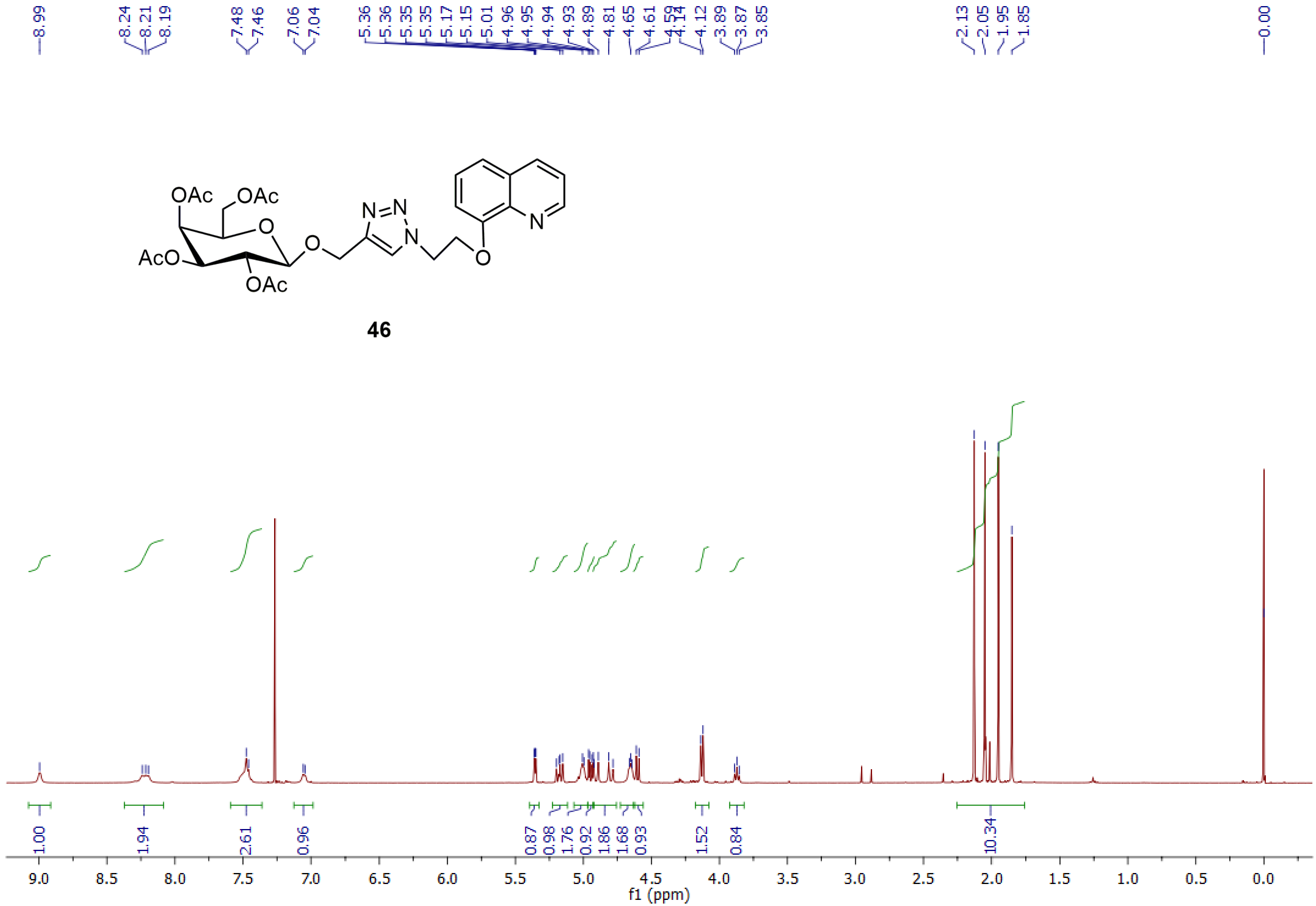

Fig. S83: ^{1}H NMR spectrum of glycoconjugate **46**.

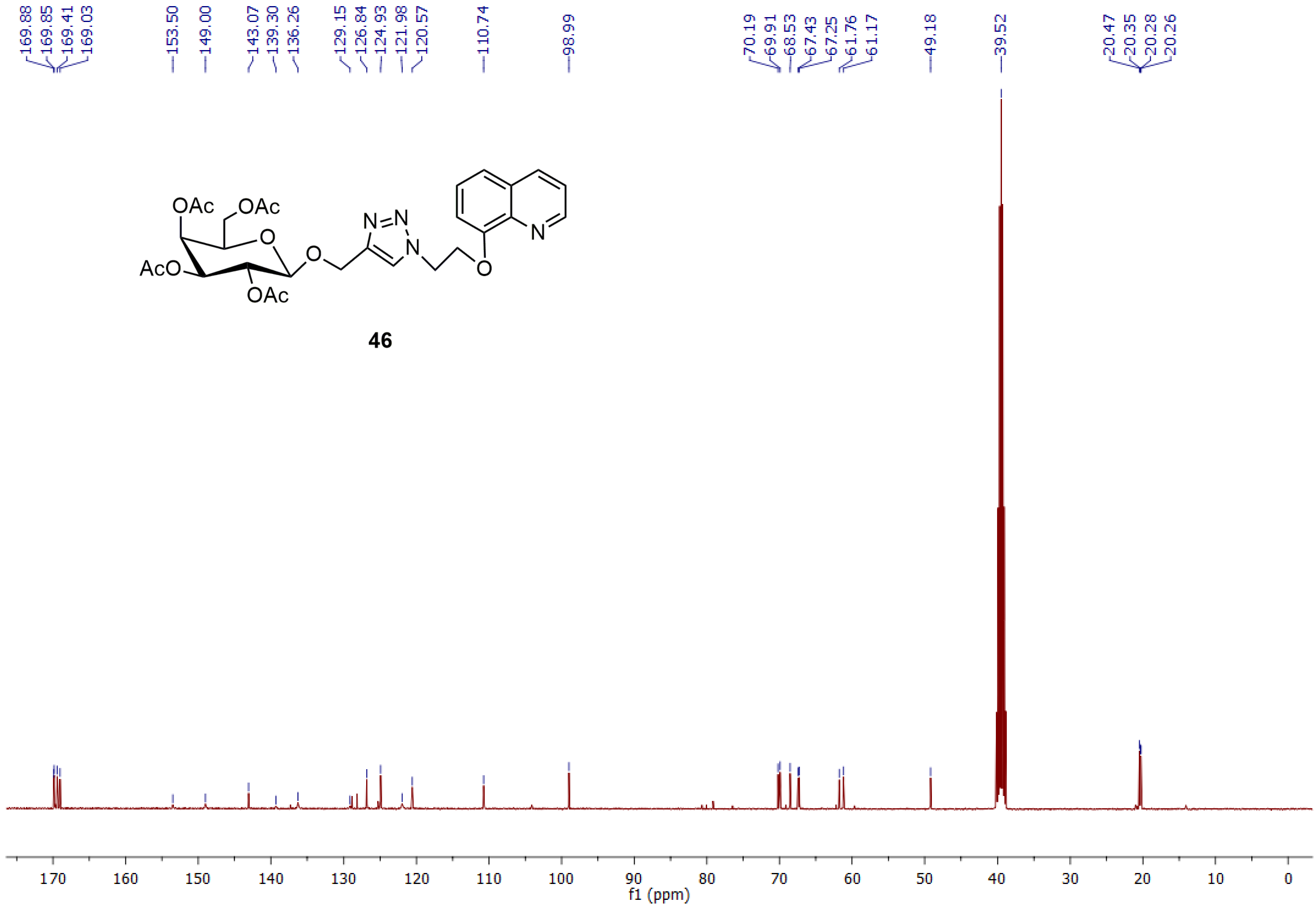

Fig. S84: ^{13}C NMR spectrum of glycoconjugate **46**.

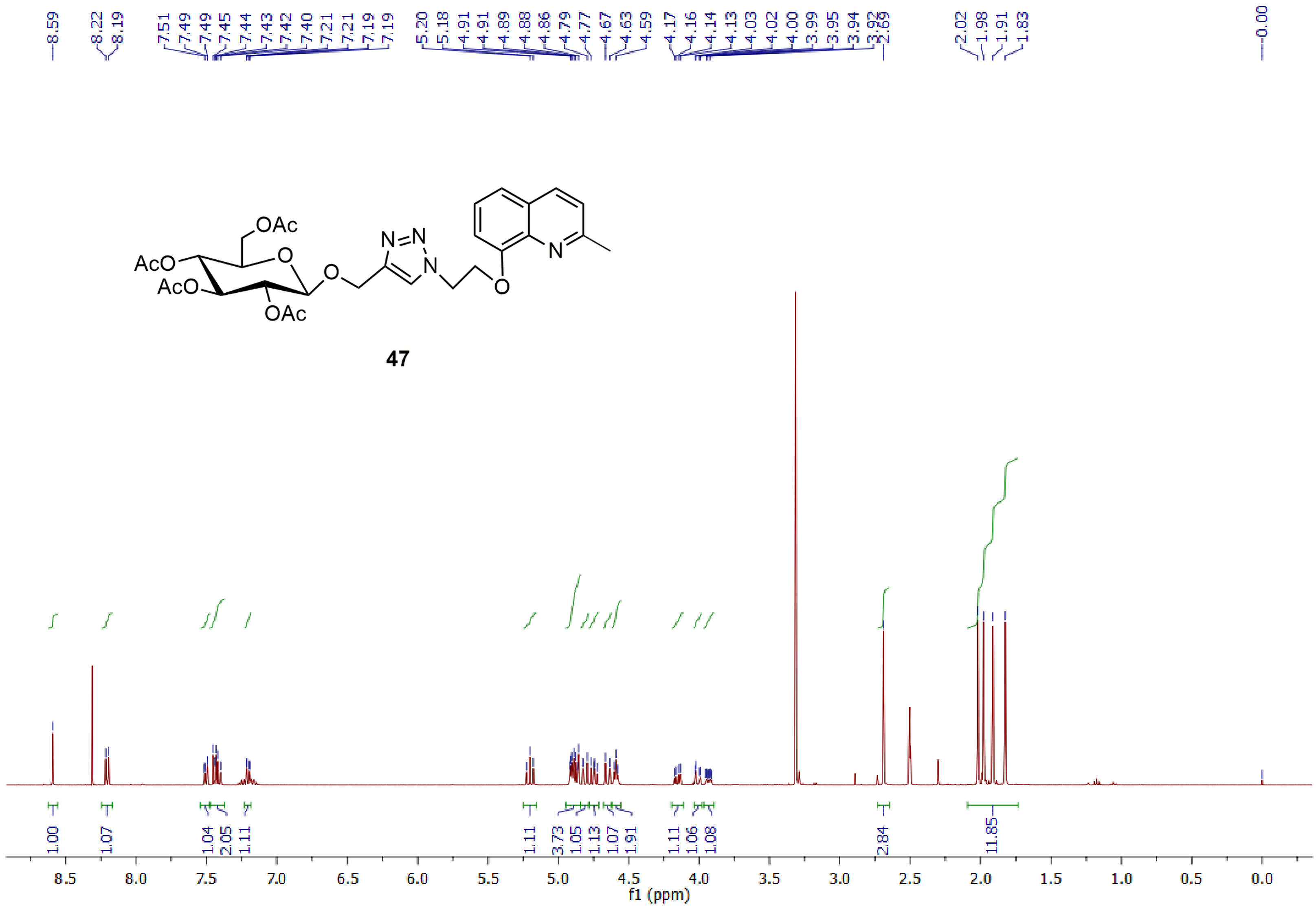

Fig. S85: ^{1}H NMR spectrum of glycoconjugate **47**.

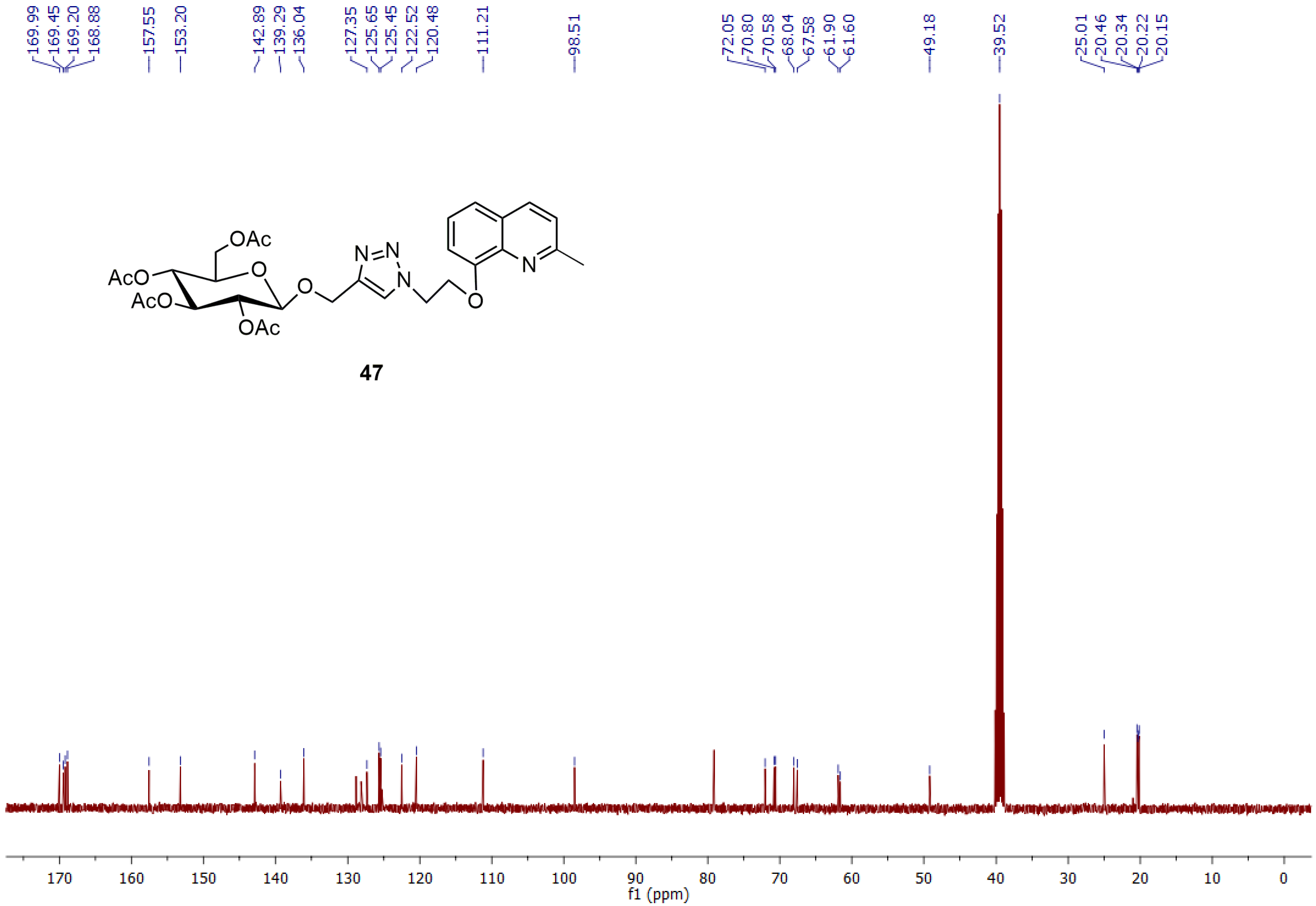

Fig. S86: ^{13}C NMR spectrum of glycoconjugate **47**.

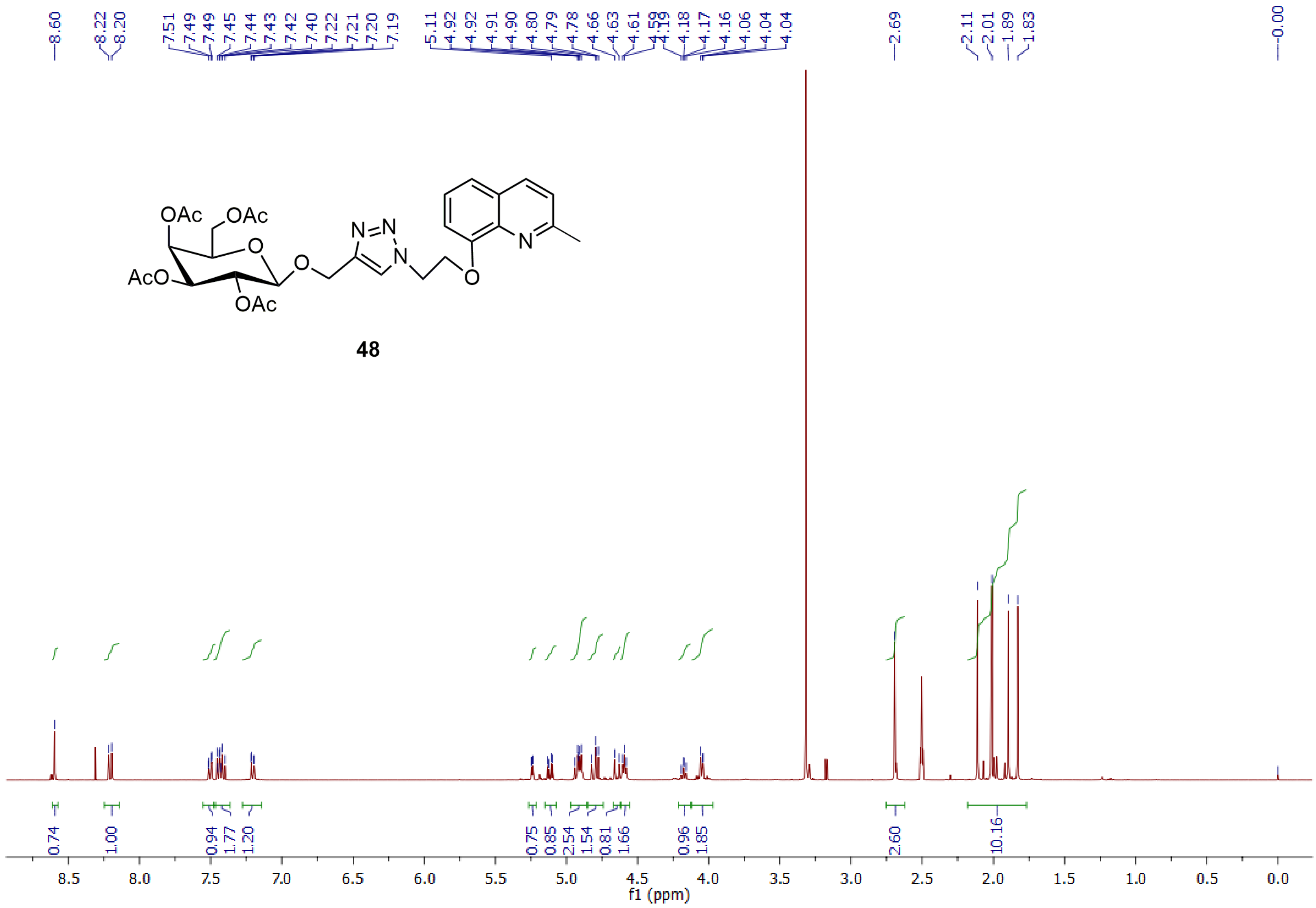

Fig. S87: ^{1}H NMR spectrum of glycoconjugate **48**.

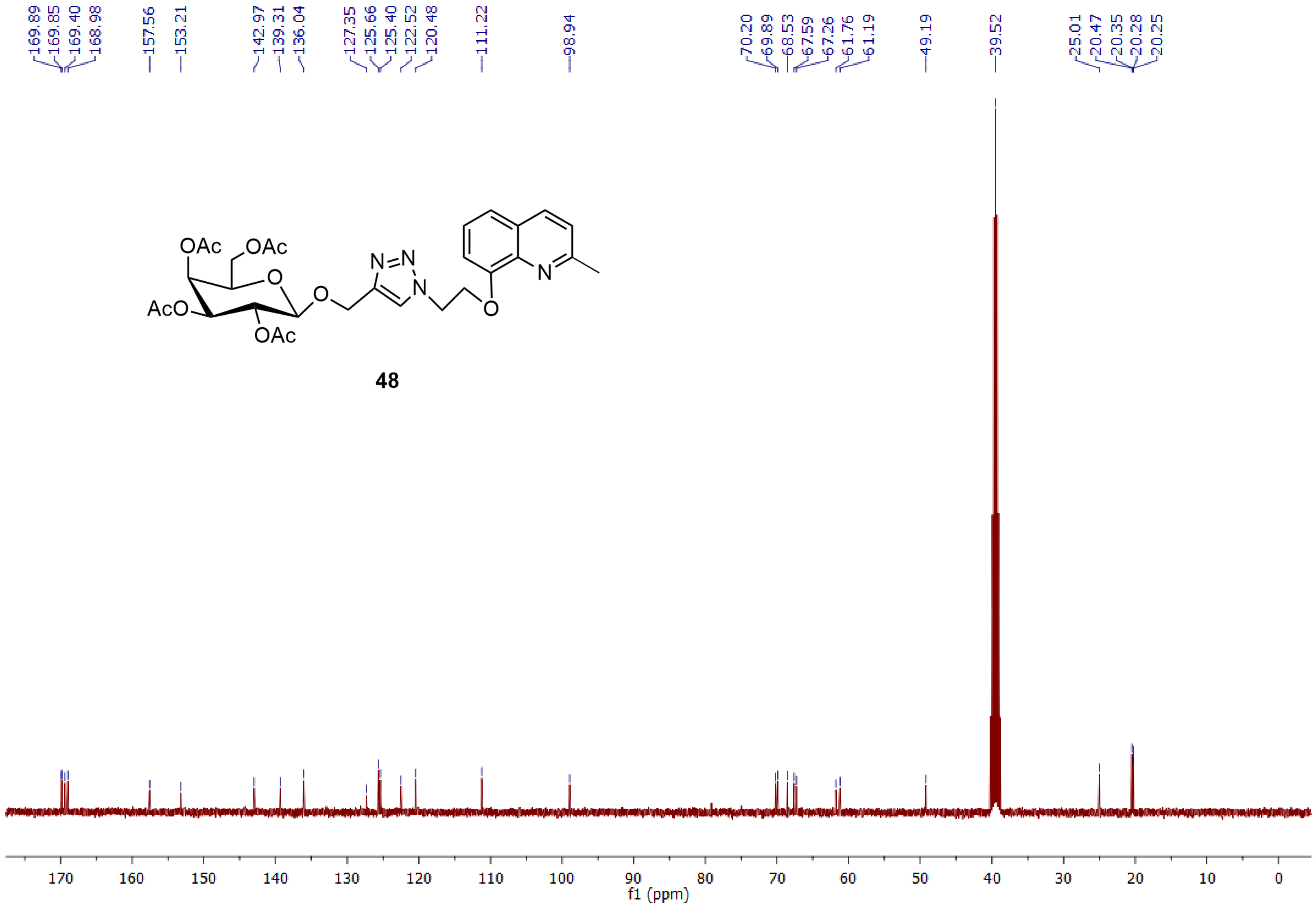

Fig. S88: ^{13}C NMR spectrum of glycoconjugate **48**.

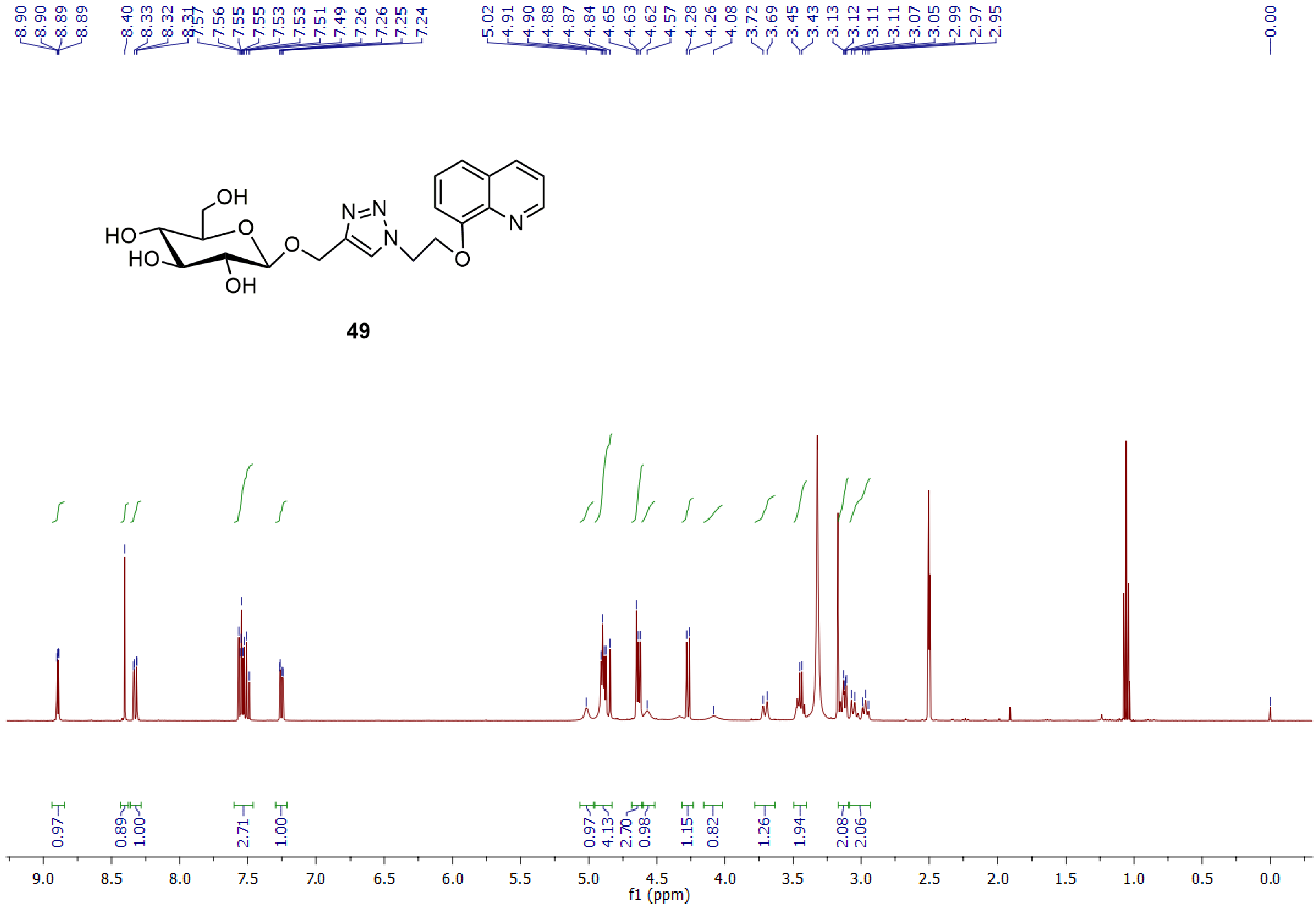

Fig. S89: ^{1}H NMR spectrum of glycoconjugate **49**.

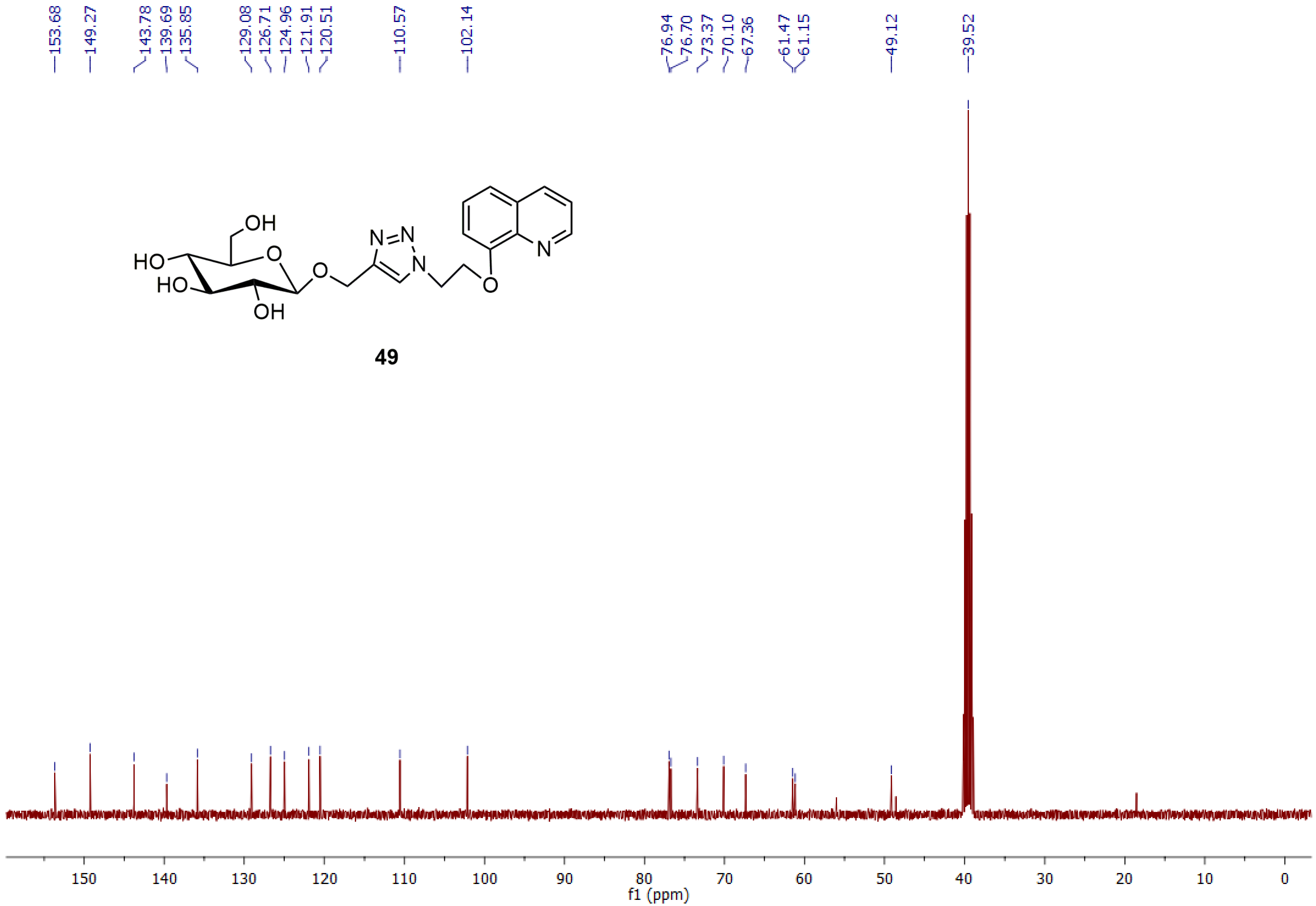

Fig. S90: ^{13}C NMR spectrum of glycoconjugate **49**.

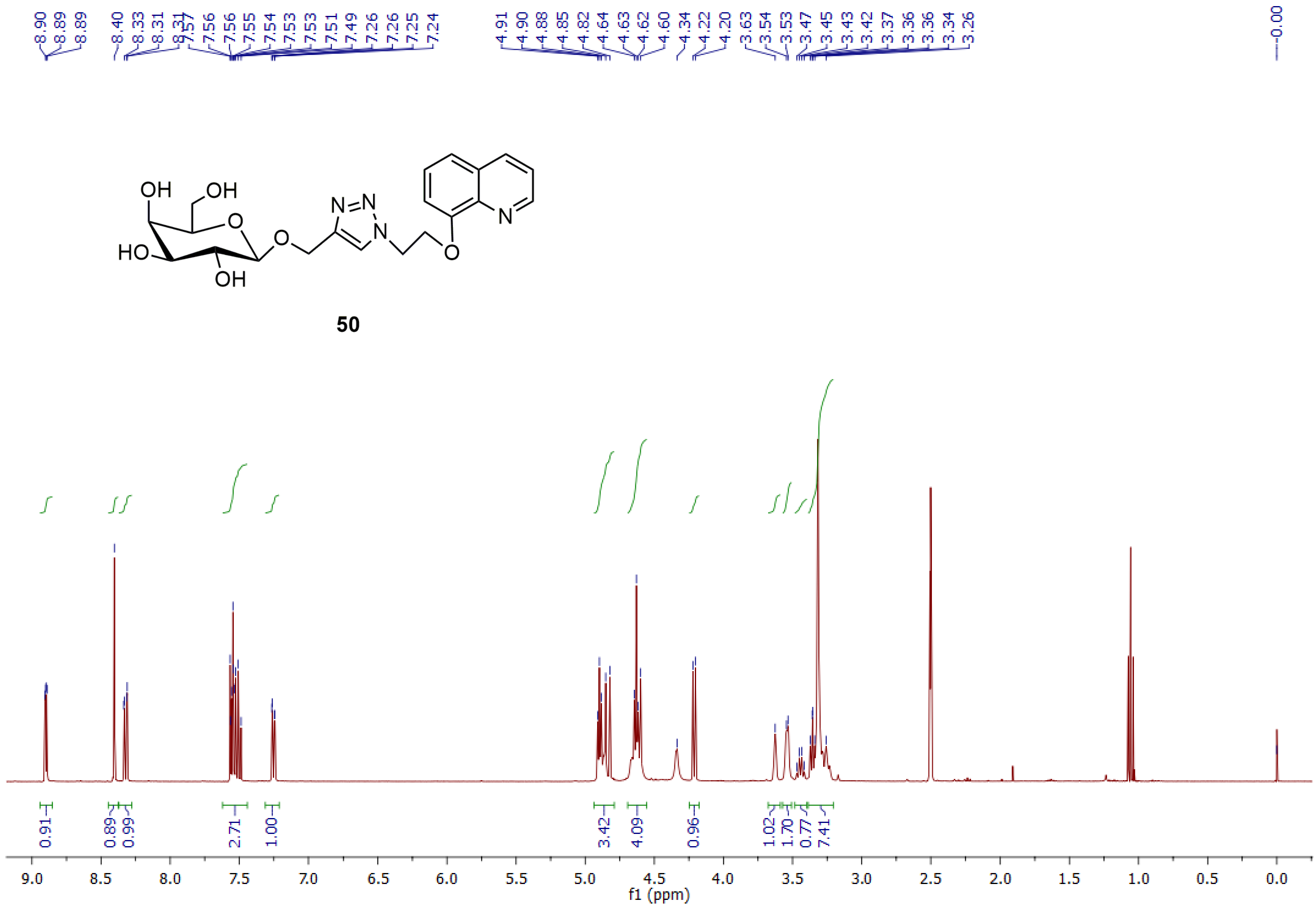

Fig. S91: ^{1}H NMR spectrum of glycoconjugate **50**.

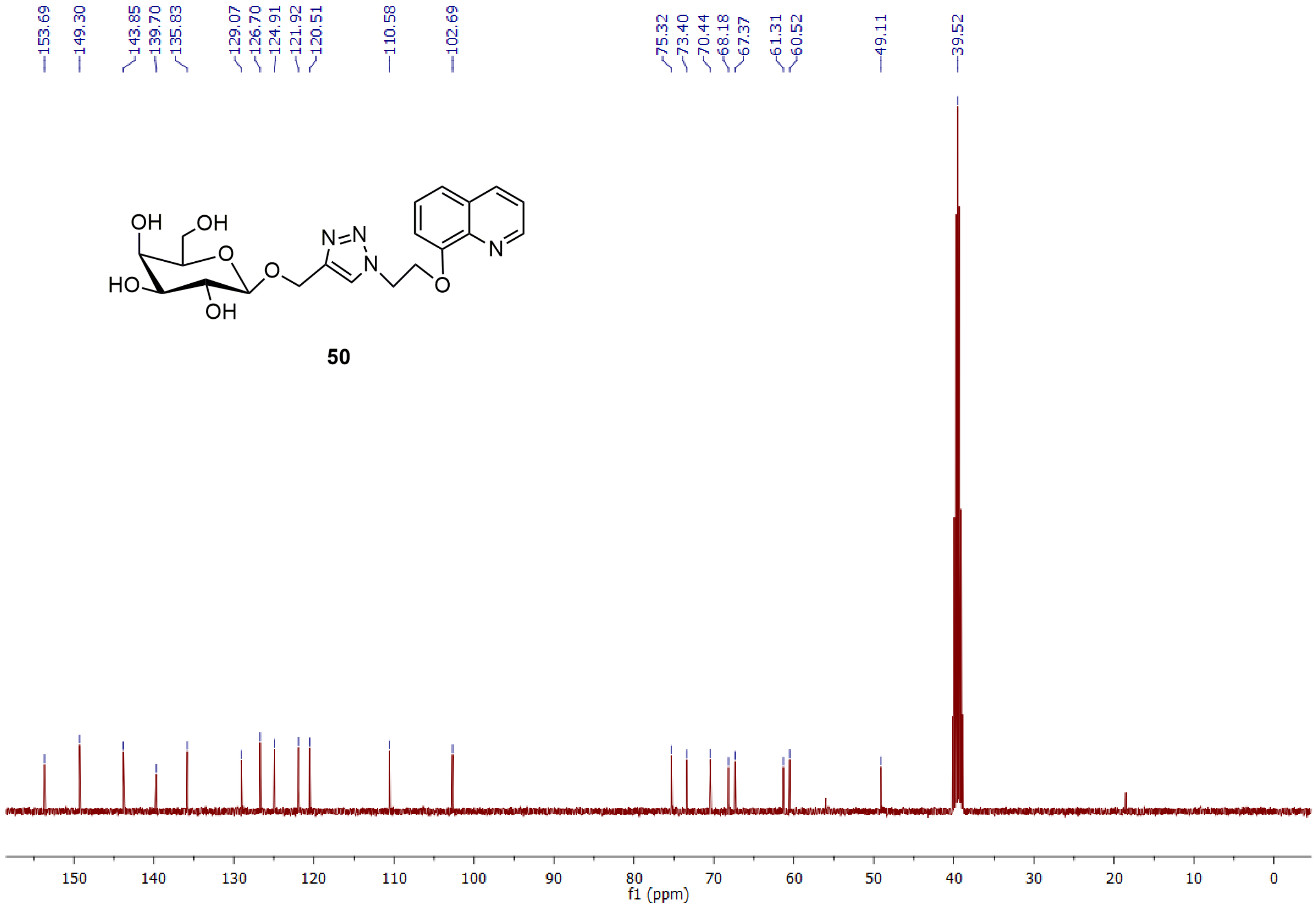

Fig. S92: ^{13}C NMR spectrum of glycoconjugate **50**.

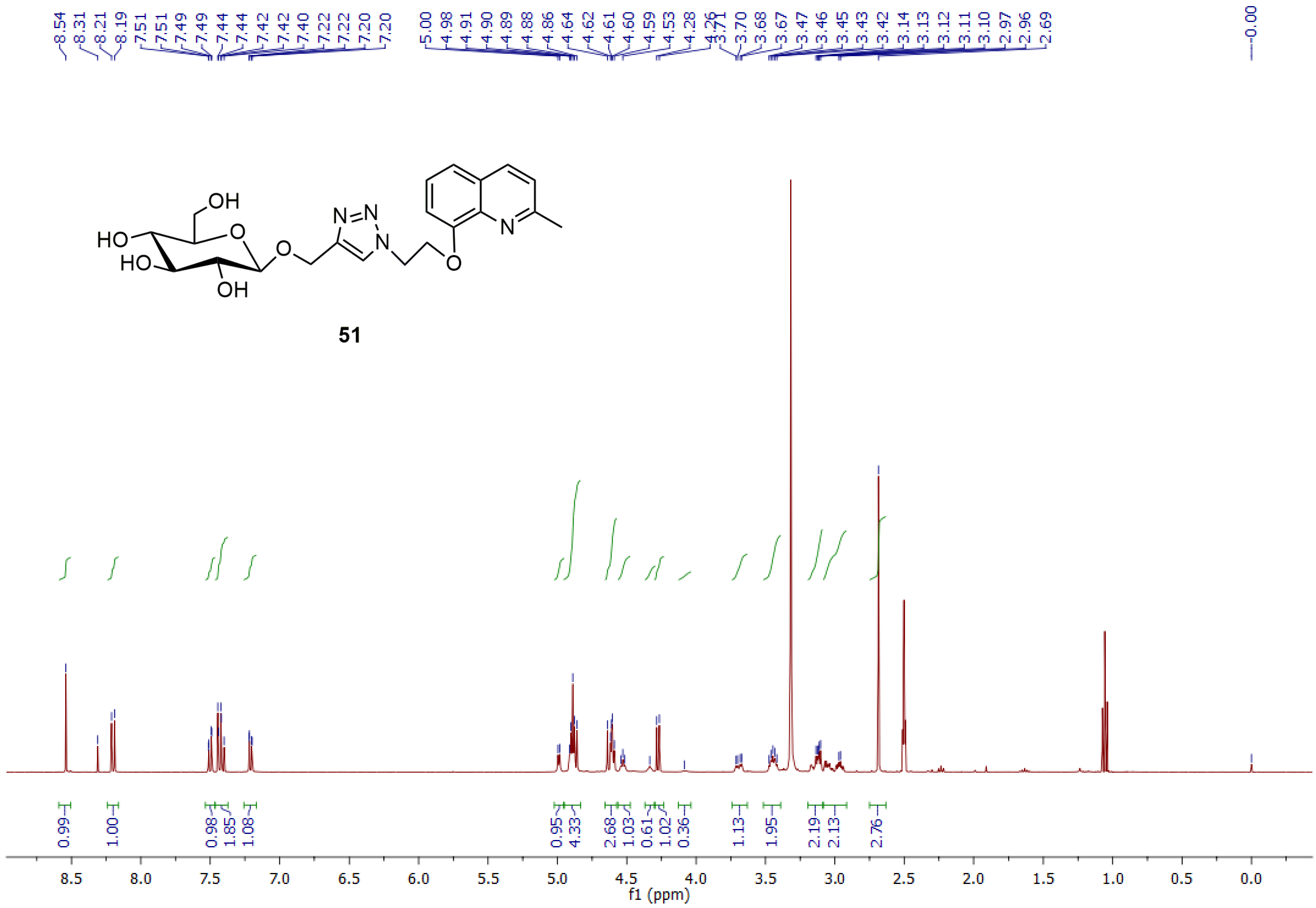

Fig. S93: ^{1}H NMR spectrum of glycoconjugate **51**.

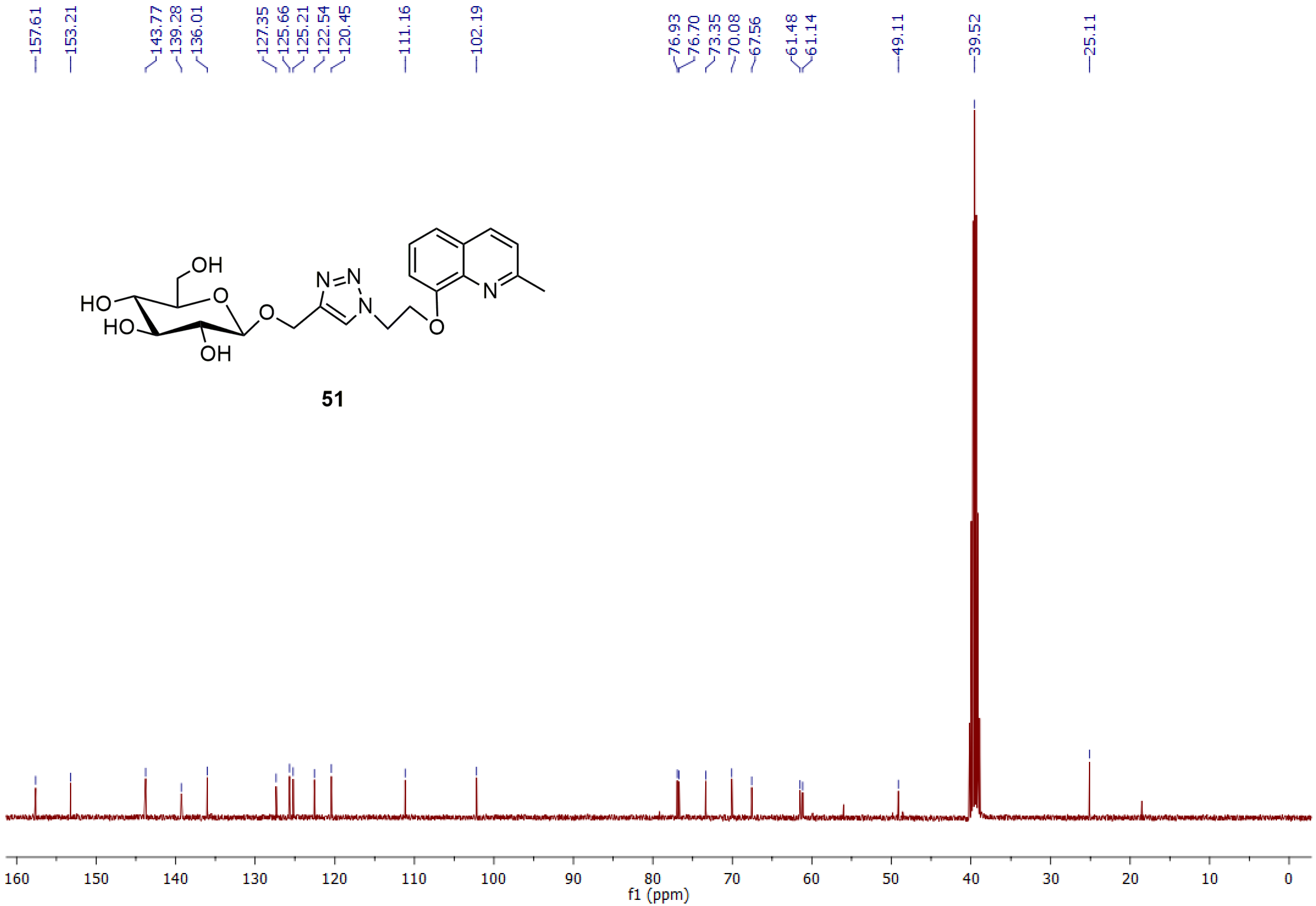

Fig. S94: ^{13}C NMR spectrum of glycoconjugate **51**.

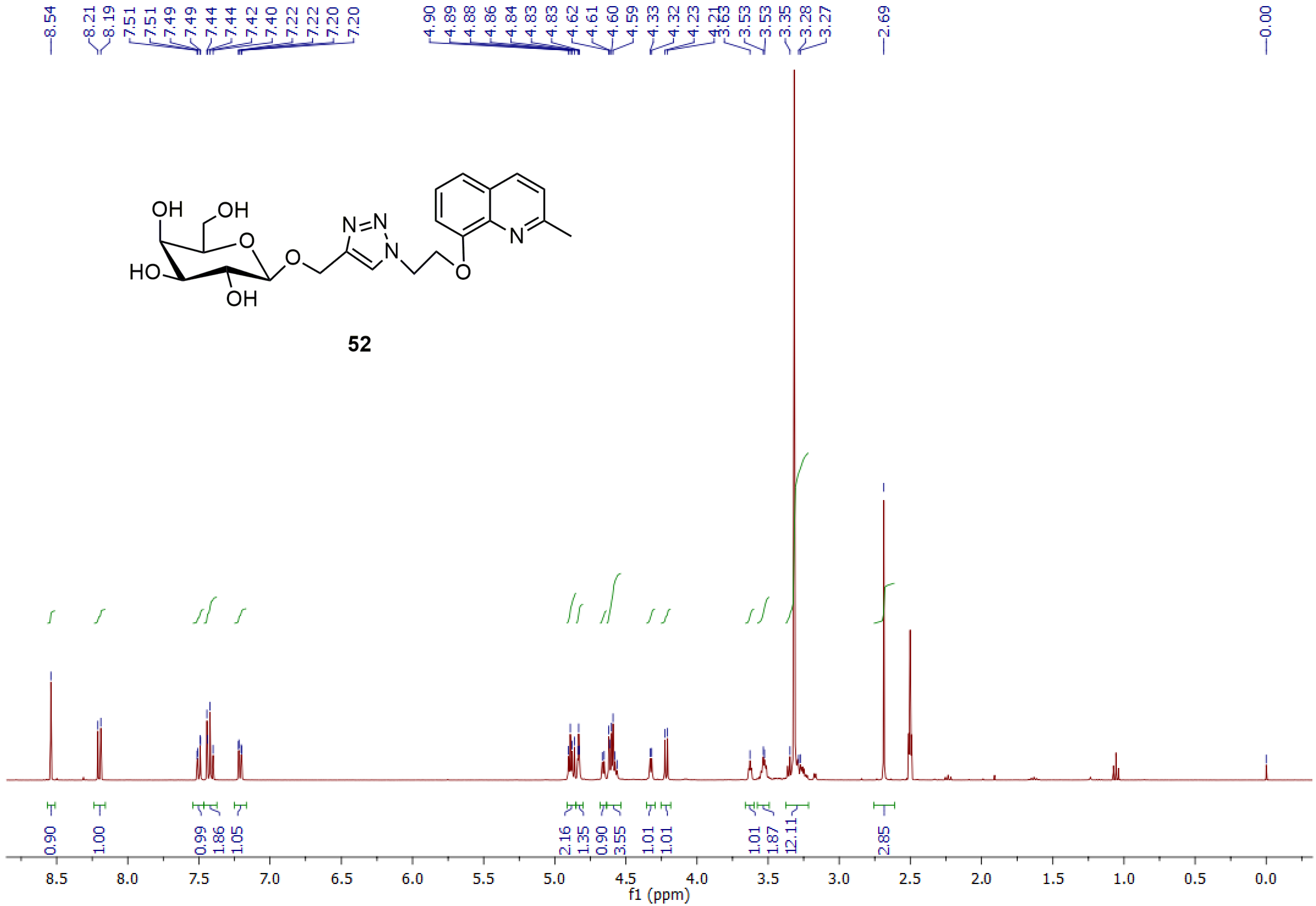

Fig. S95: ^{1}H NMR spectrum of glycoconjugate **52**.

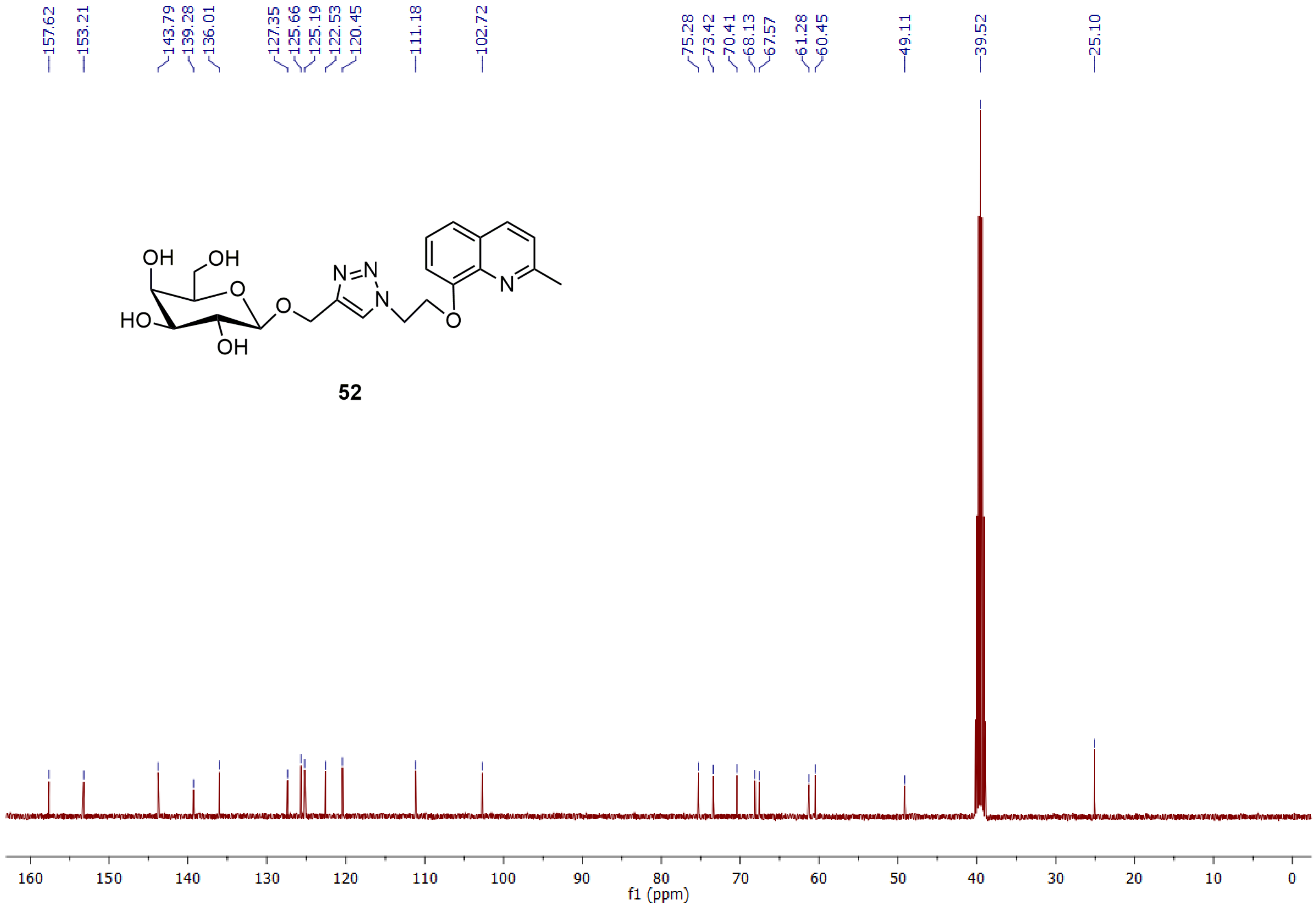

Fig. S96: ^{13}C NMR spectrum of glycoconjugate **52**.

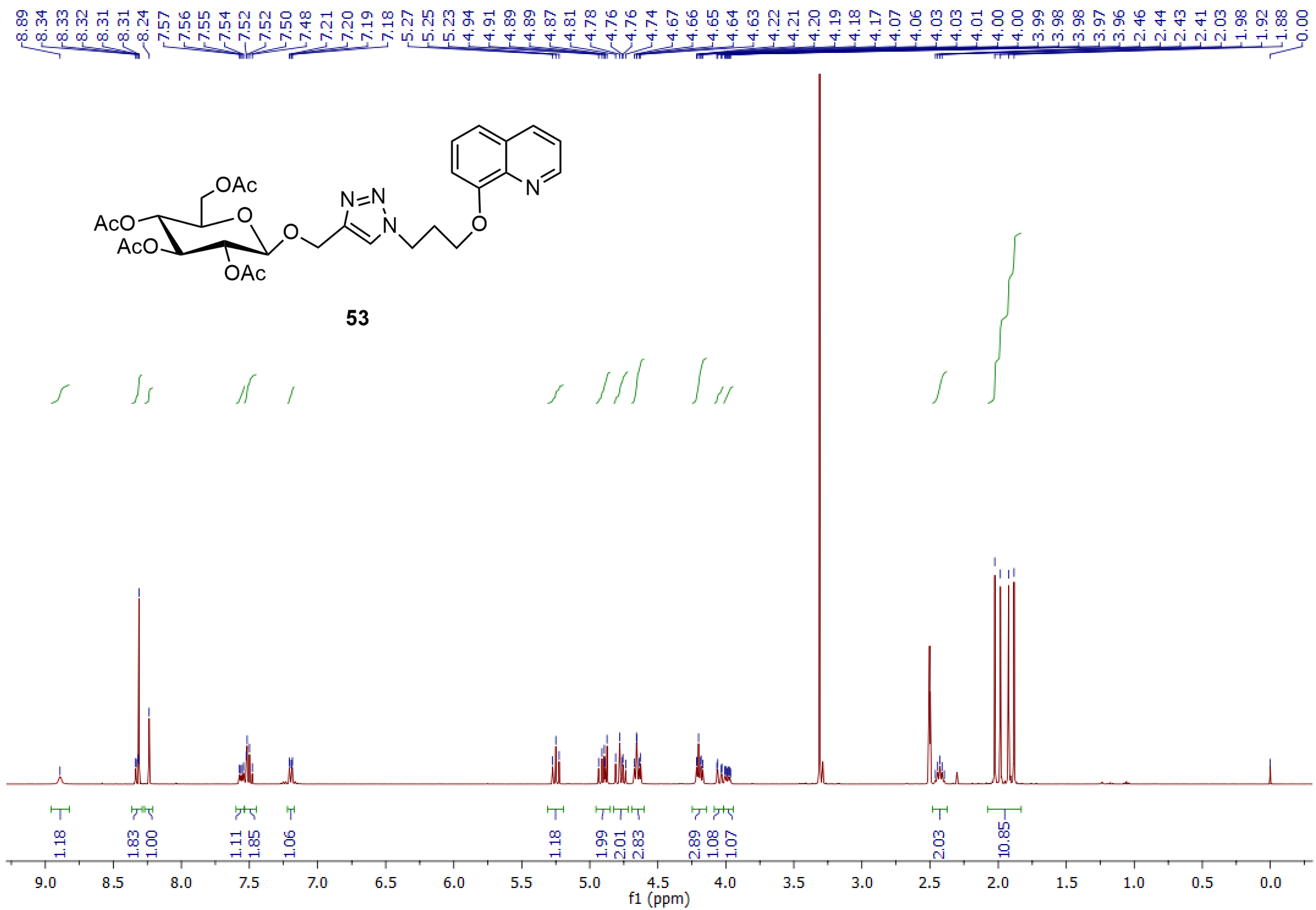

Fig. S97: ^{1}H NMR spectrum of glycoconjugate **53**.

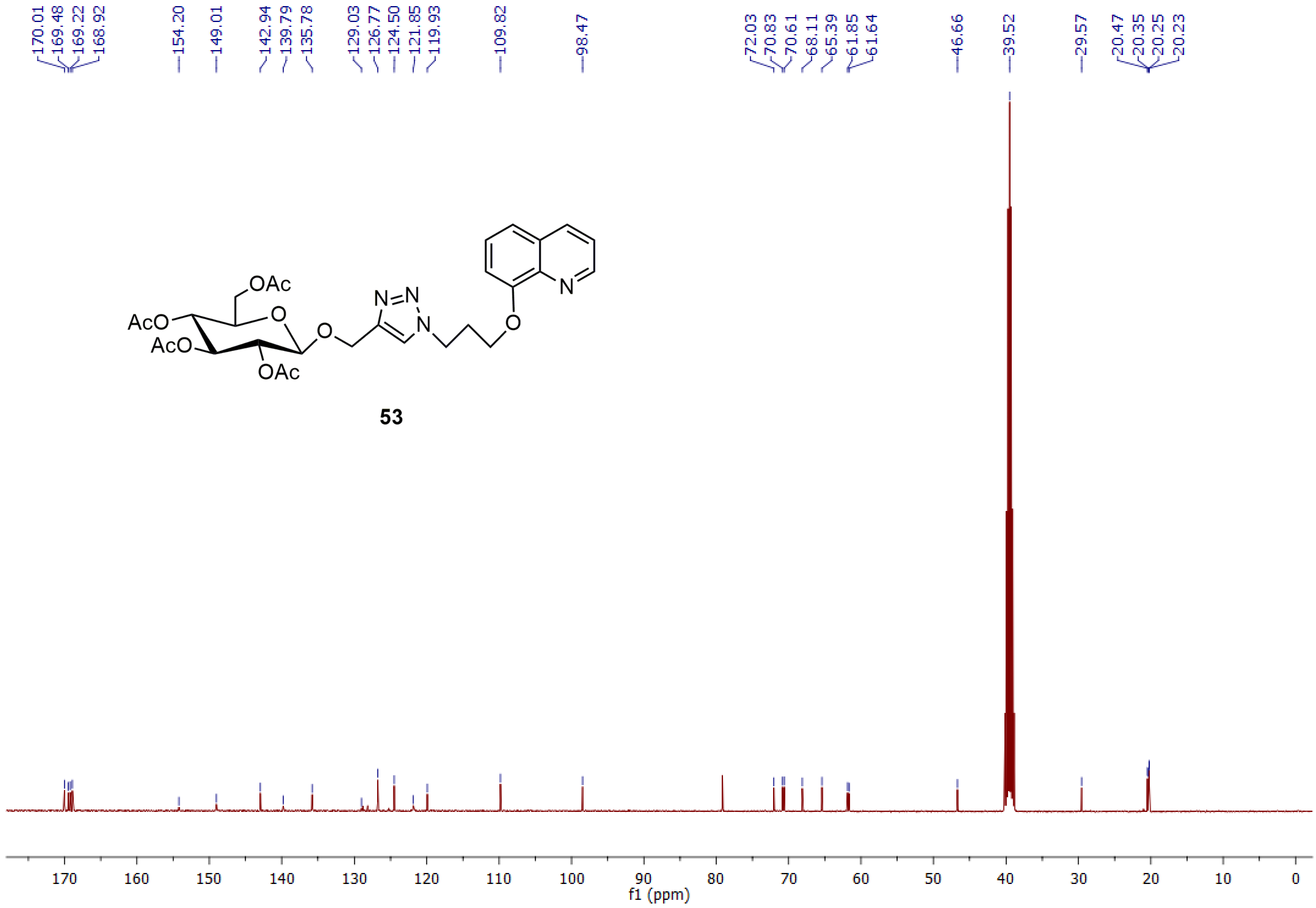

Fig. S98: ^{13}C NMR spectrum of glycoconjugate **53**.

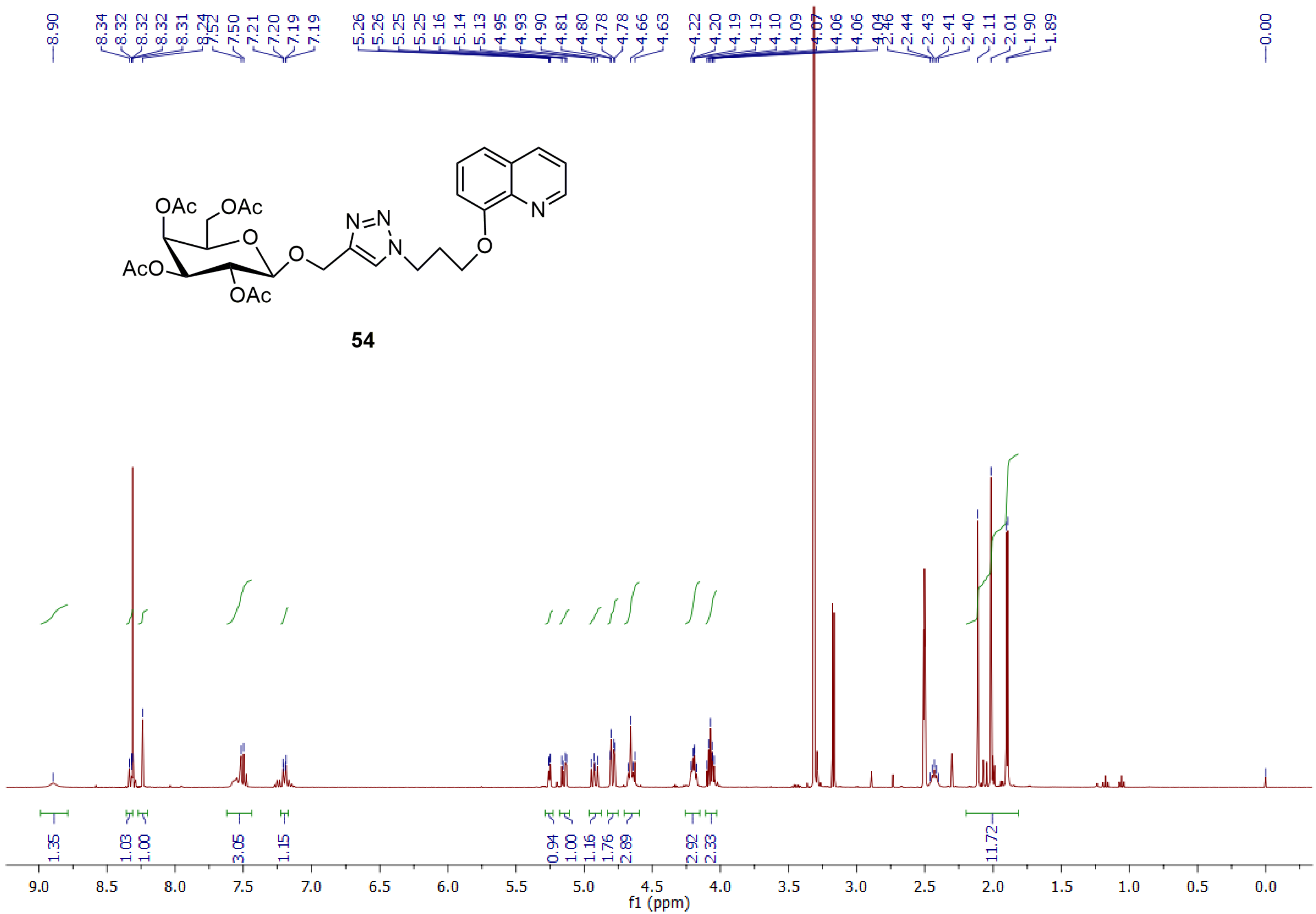

Fig. S99: ^{1}H NMR spectrum of glycoconjugate **54**.

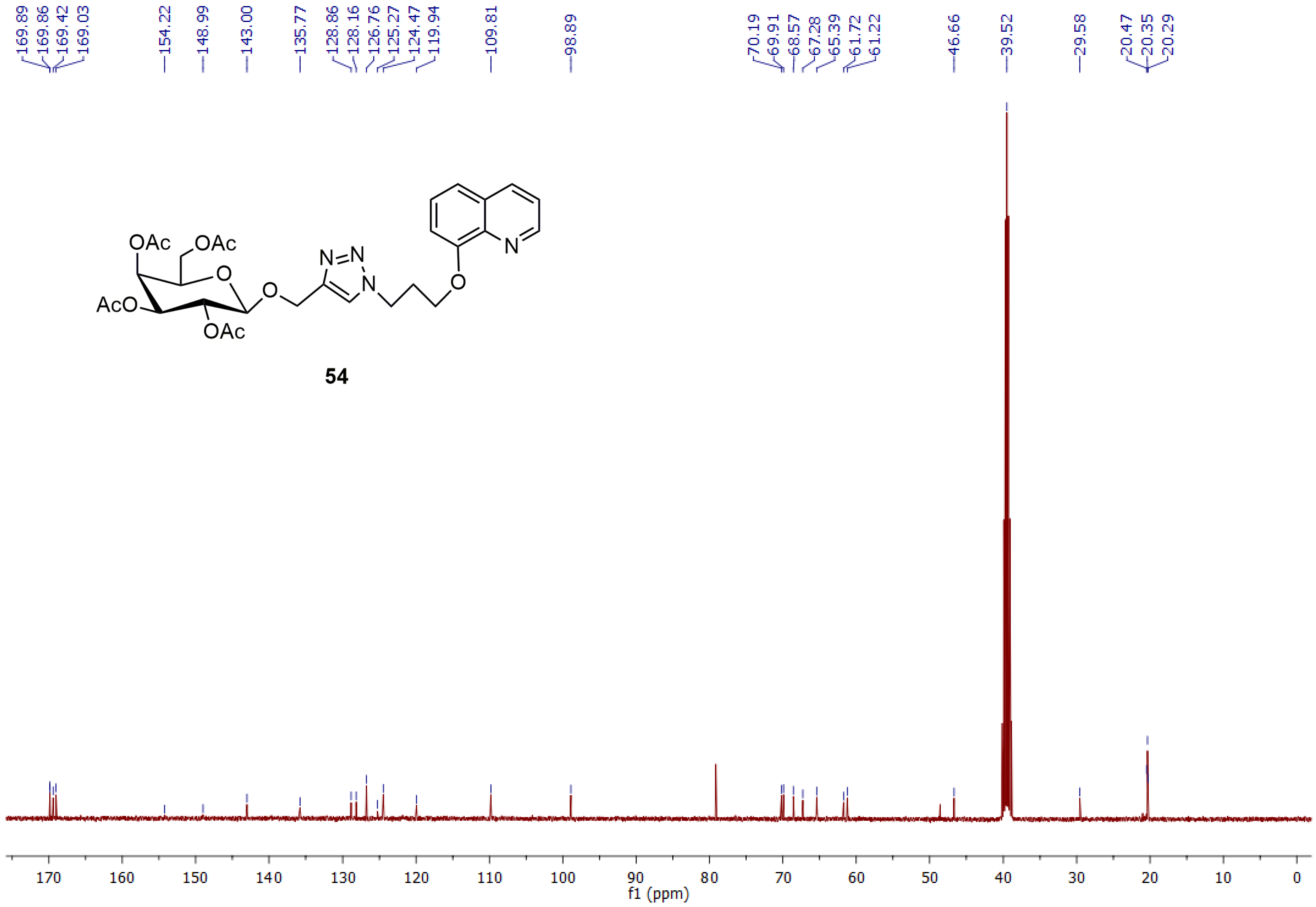

Fig. S100: ^{13}C NMR spectrum of glycoconjugate **54**.

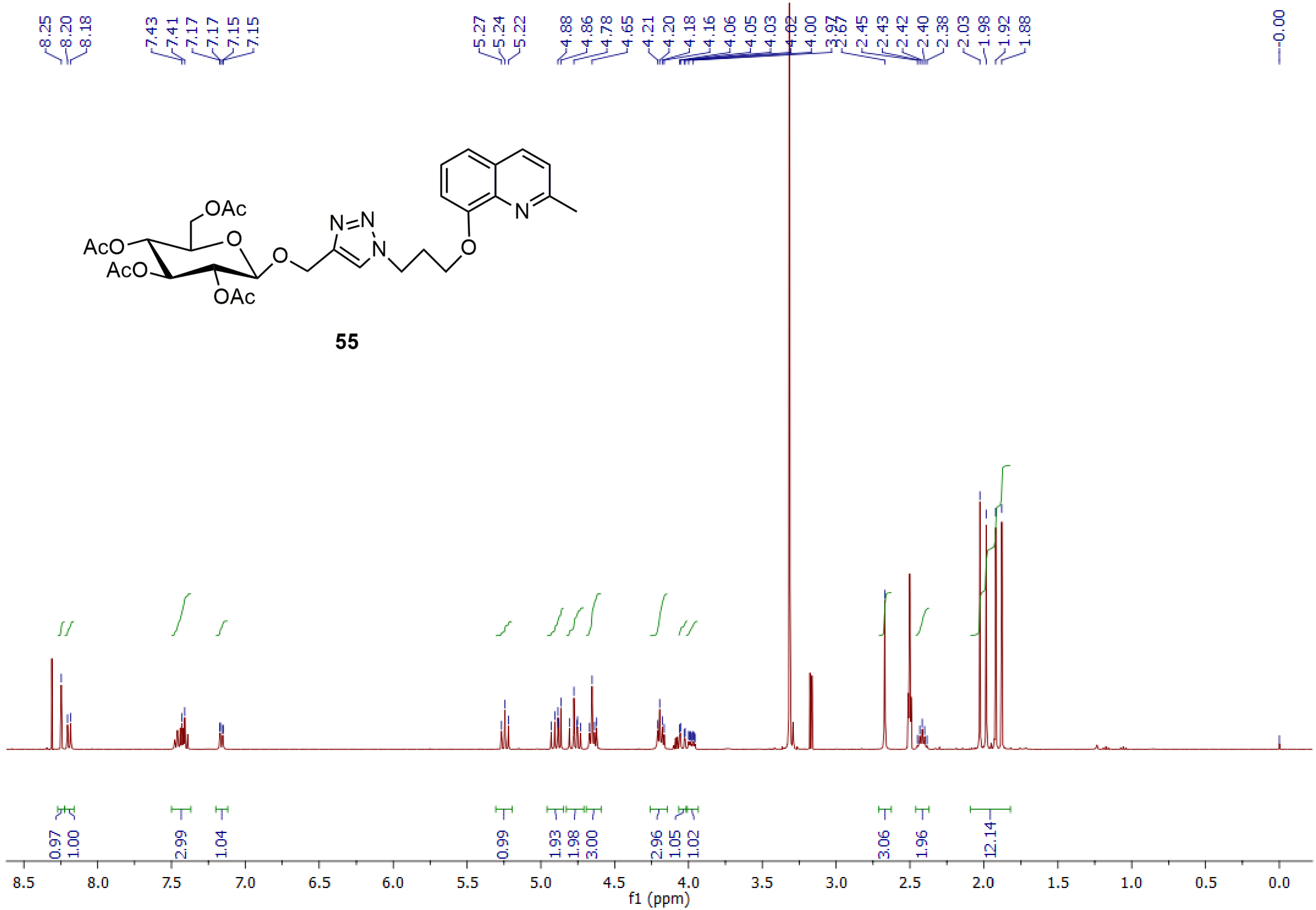

Fig. S101: ^{1}H NMR spectrum of glycoconjugate **55**.

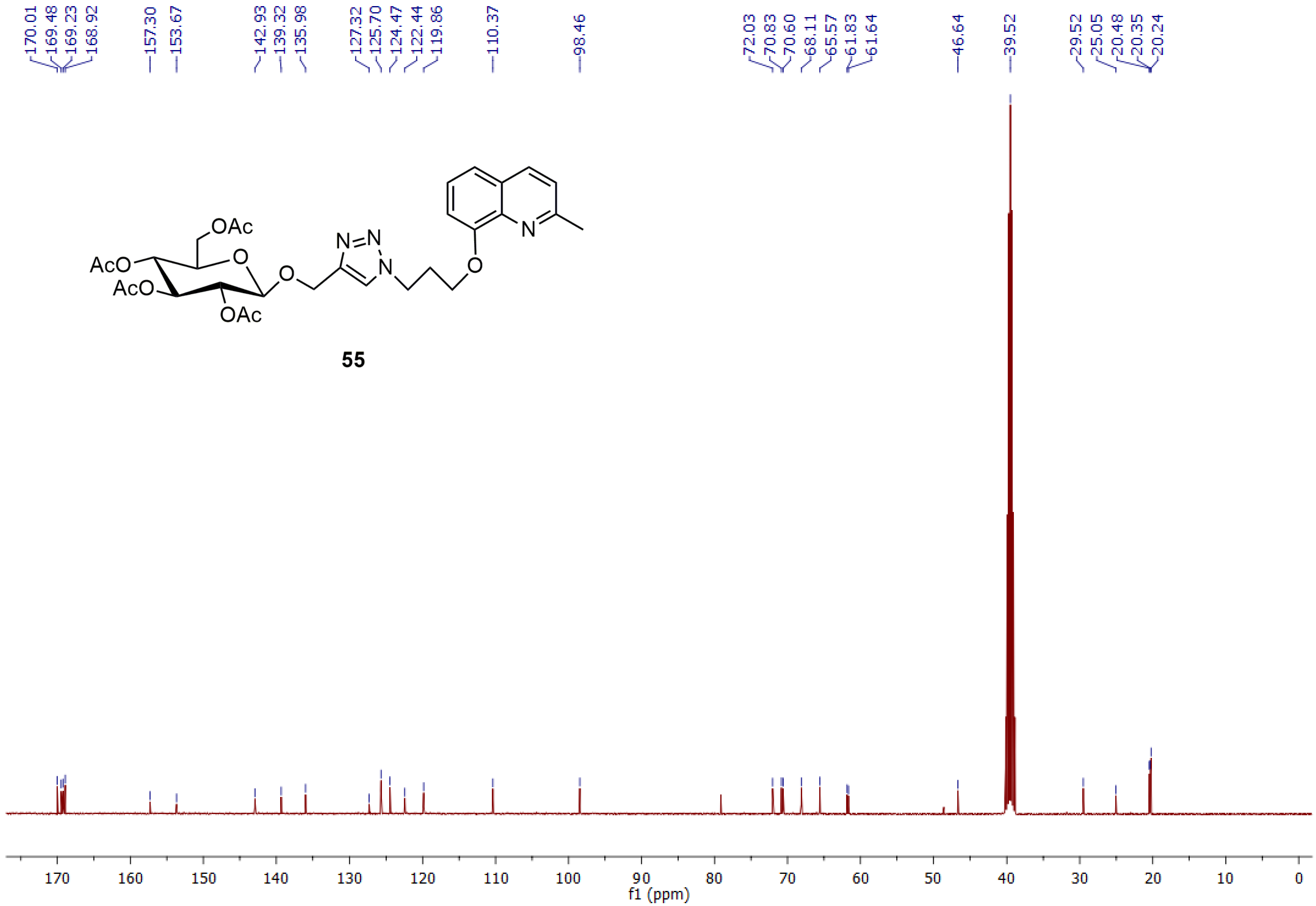

Fig. S102: ^{13}C NMR spectrum of glycoconjugate **55**.

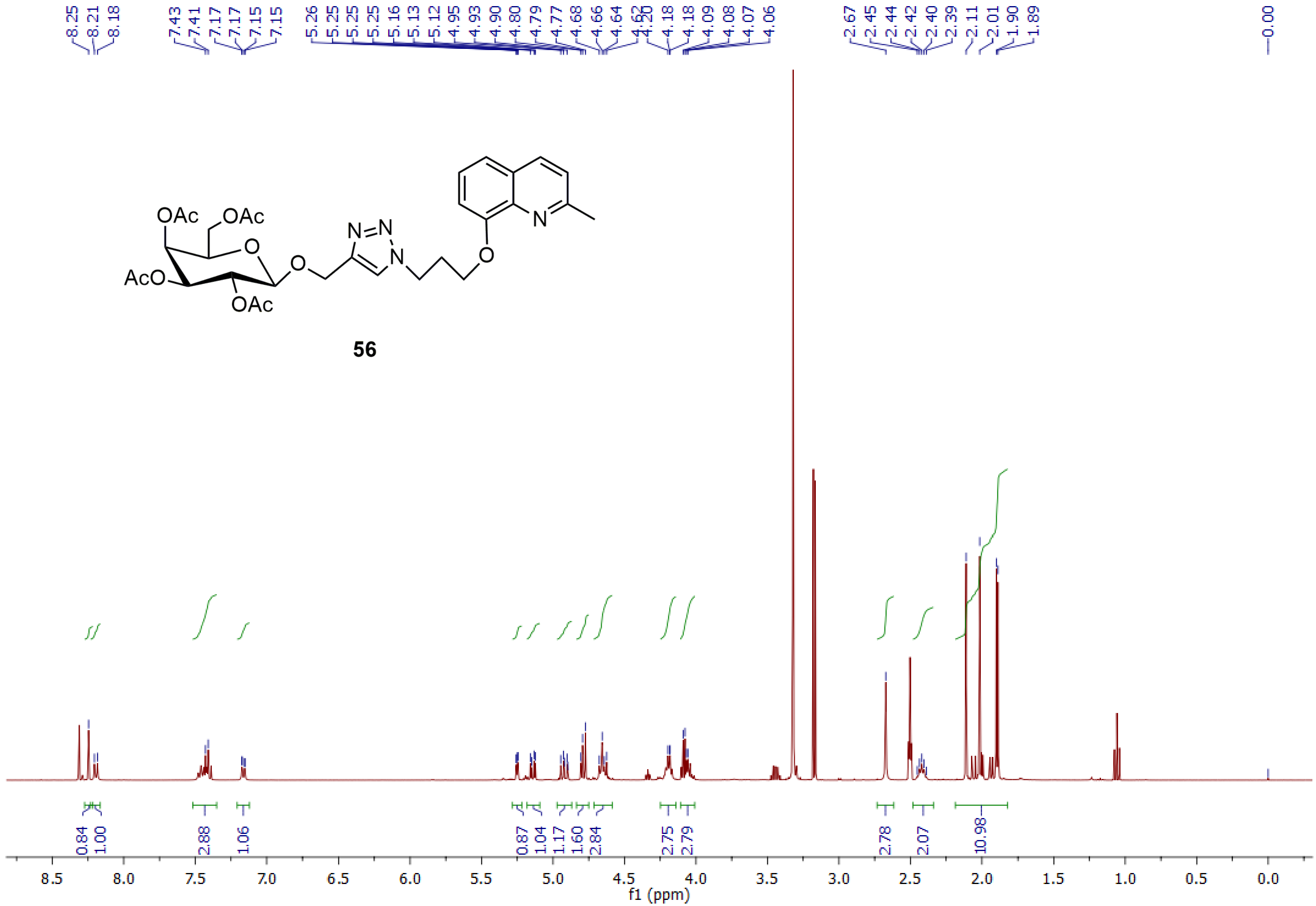

Fig. S103: ^{1}H NMR spectrum of glycoconjugate **56**.

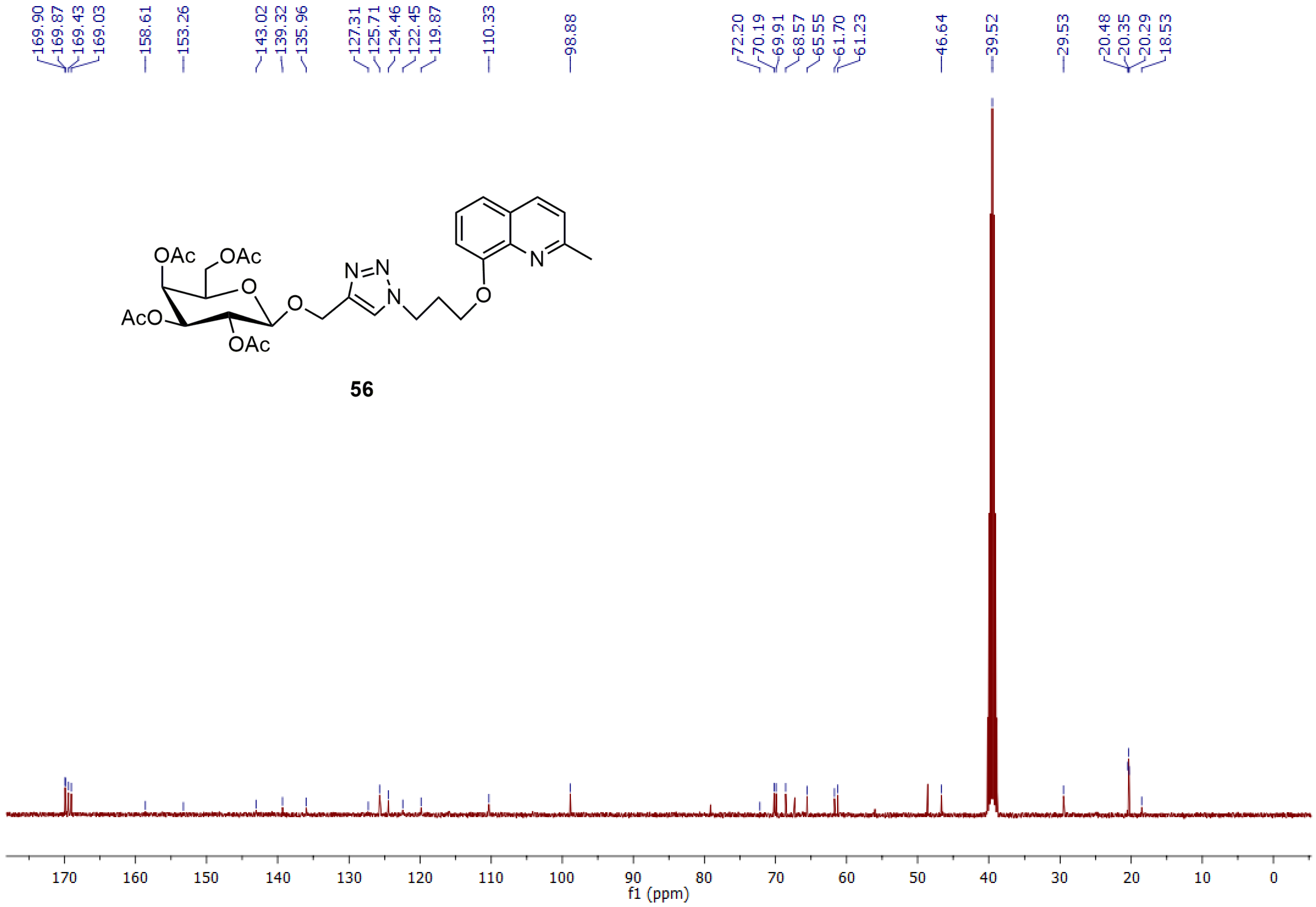

Fig. S104: ^{13}C NMR spectrum of glycoconjugate **56**.

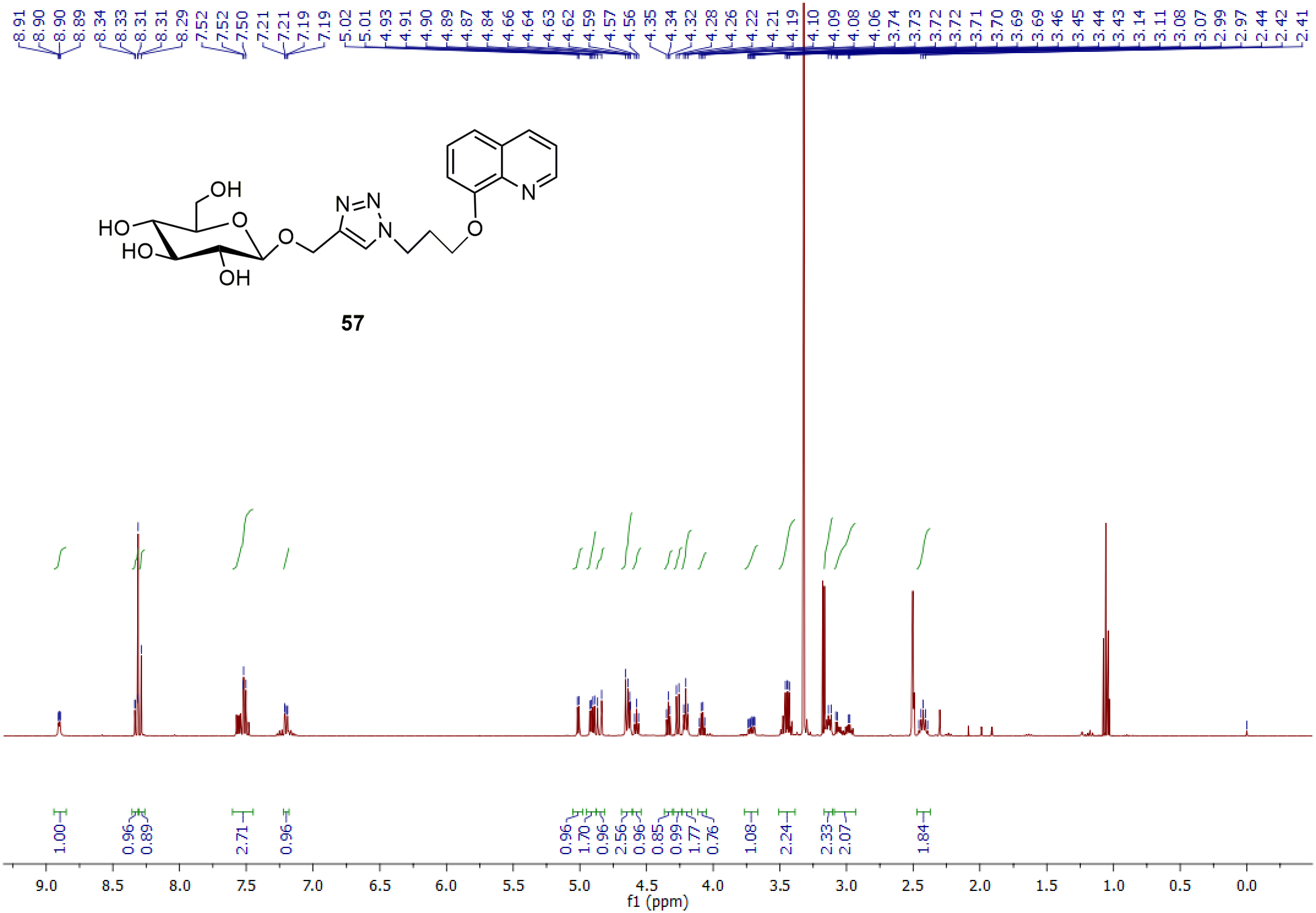

Fig. S105: ^{1}H NMR spectrum of glycoconjugate **57**.

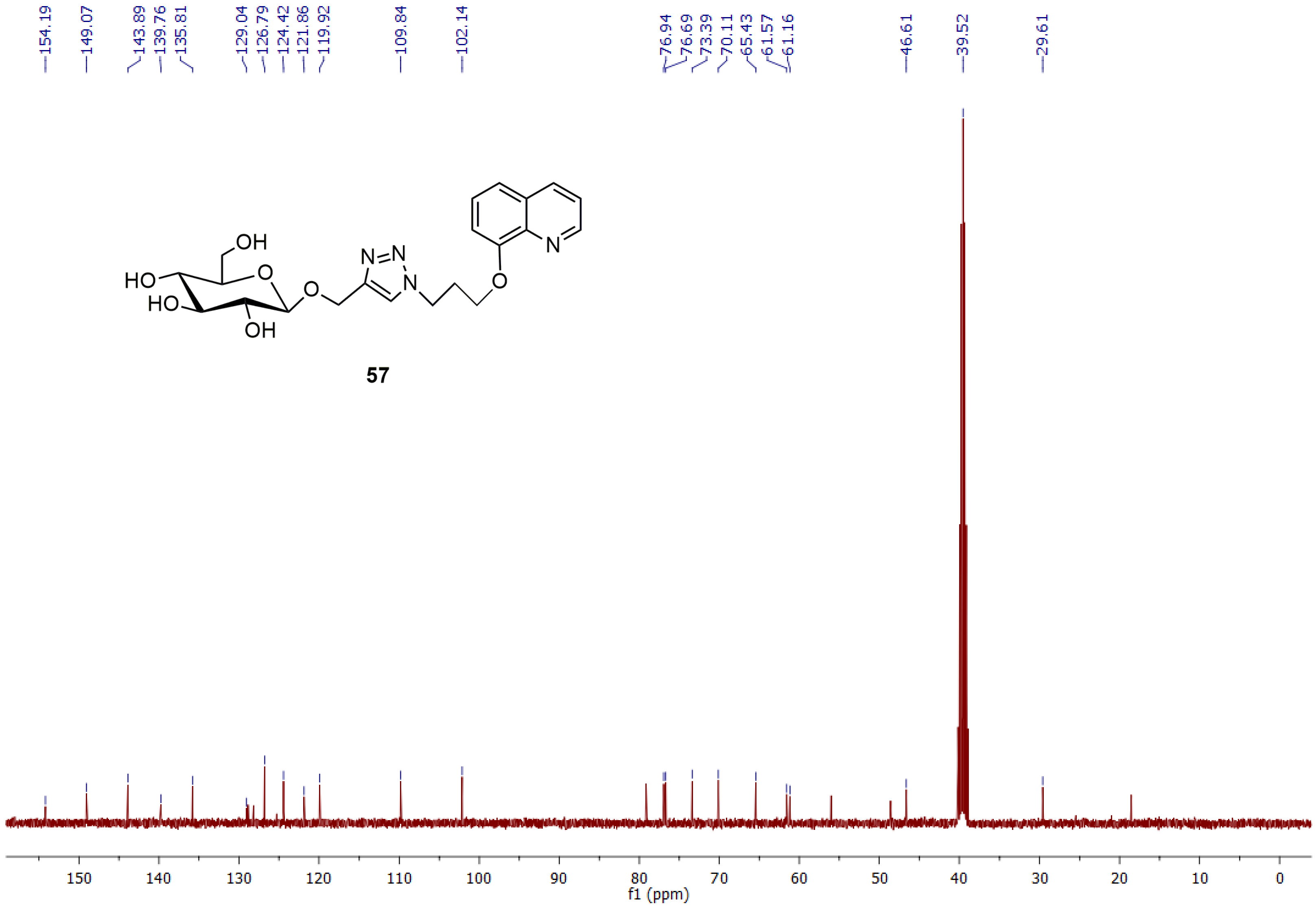

Fig. S106: ^{13}C NMR spectrum of glycoconjugate **57**.

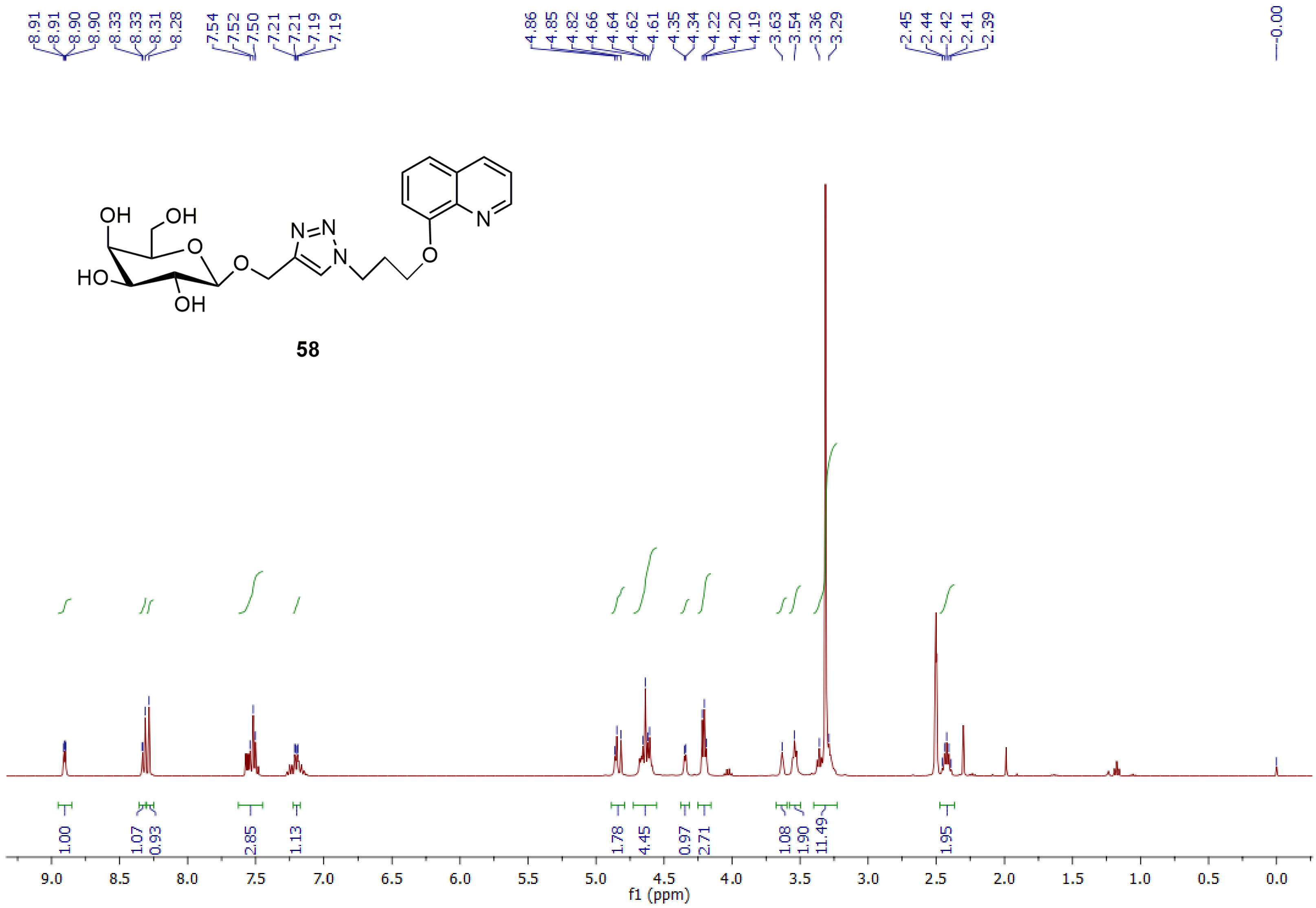

Fig. S107: ^{1}H NMR spectrum of glycoconjugate **58**.

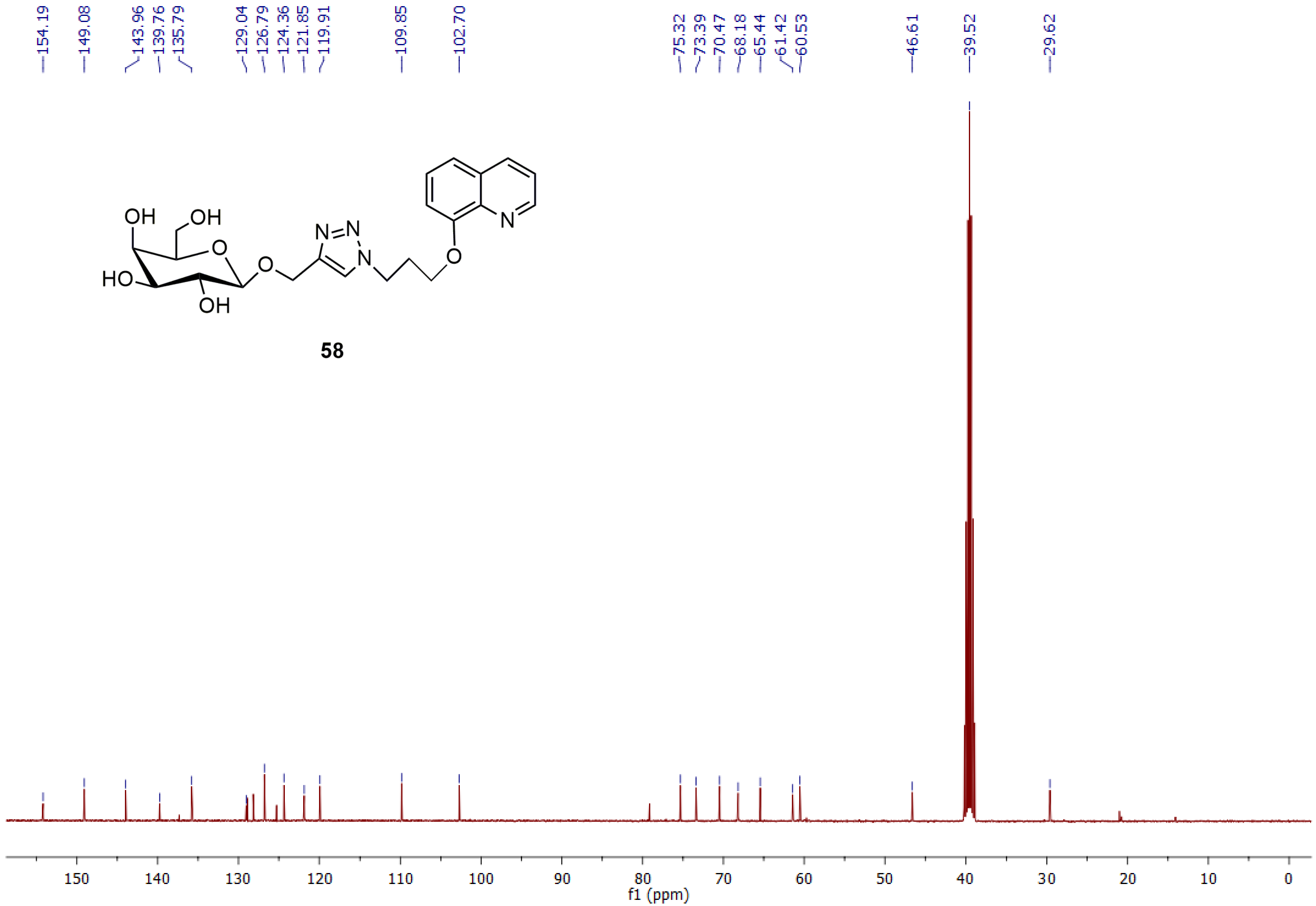

Fig. S108: ^{13}C NMR spectrum of glycoconjugate **58**.

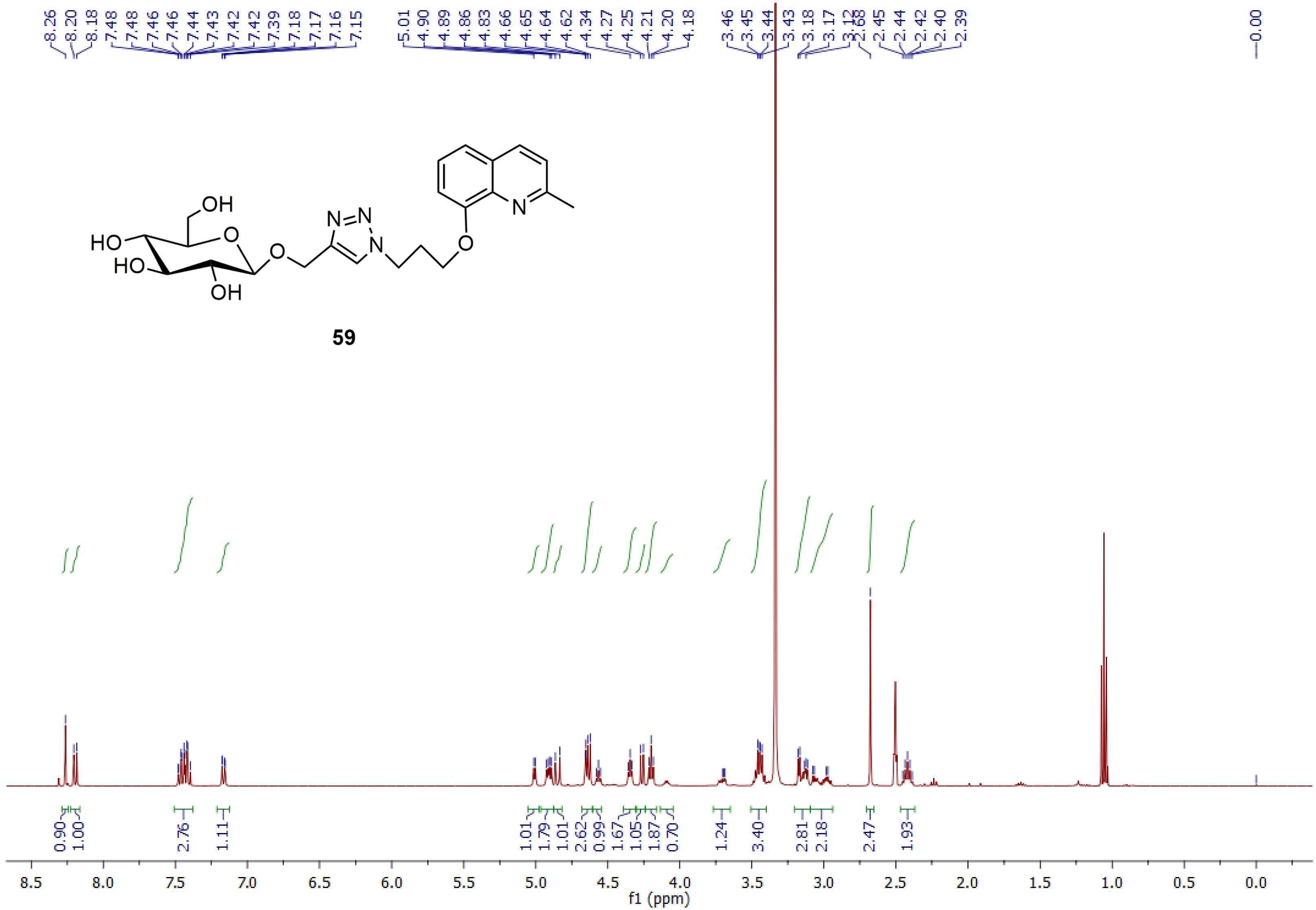

Fig. S109: ^{1}H NMR spectrum of glycoconjugate **59**.

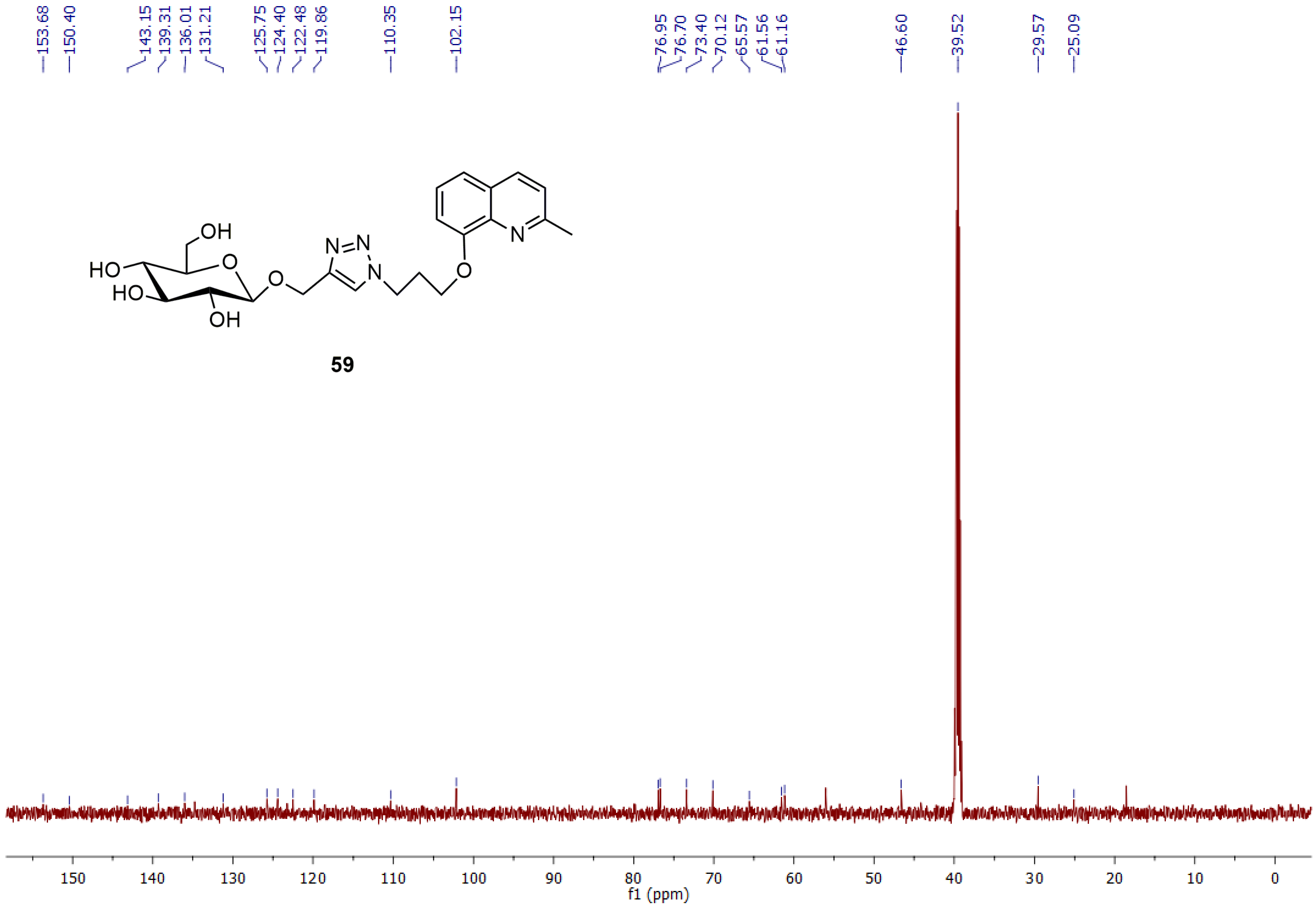

Fig. S110: ^{13}C NMR spectrum of glycoconjugate **59**.

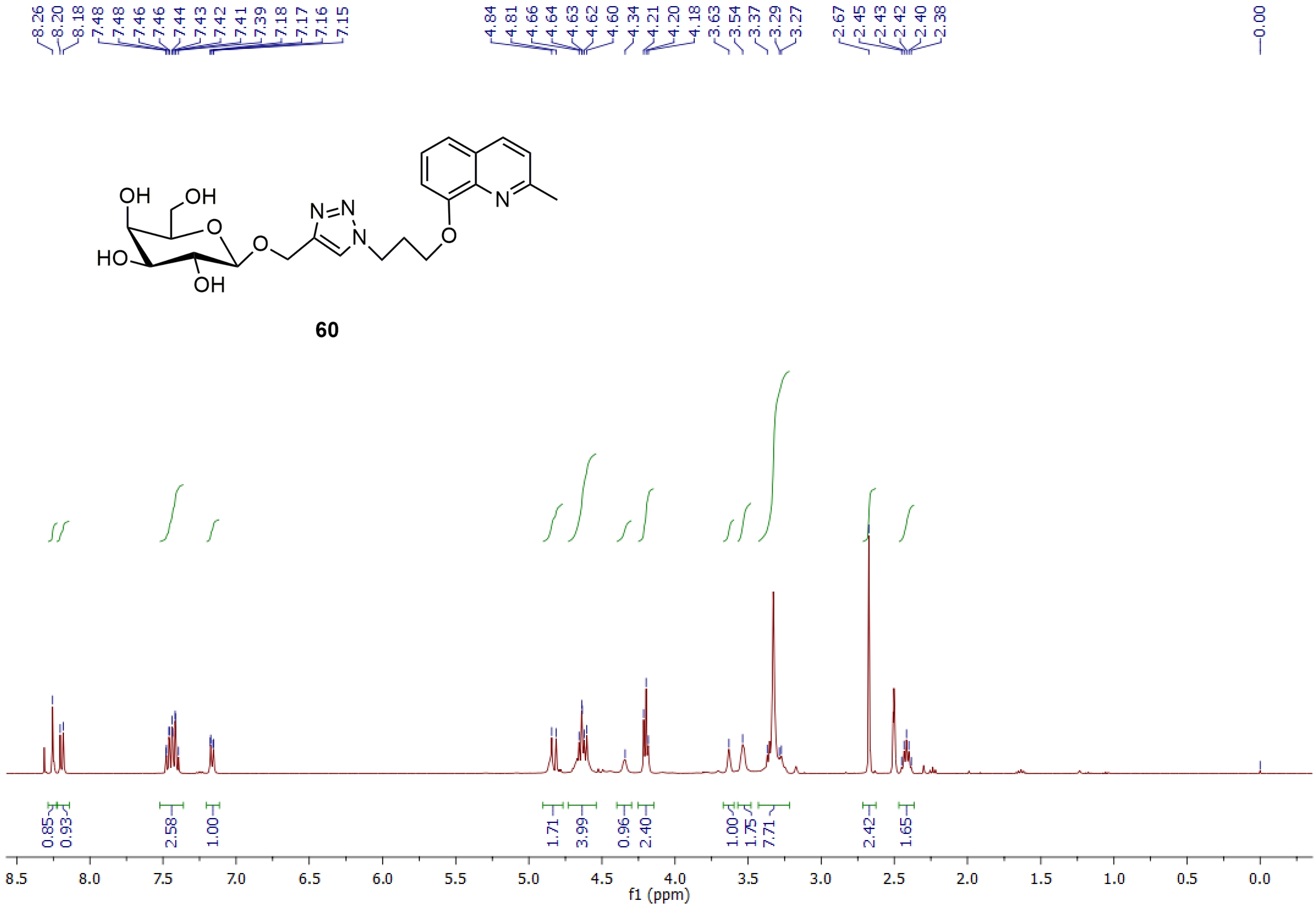

Fig. S111: ^{1}H NMR spectrum of glycoconjugate **60**.

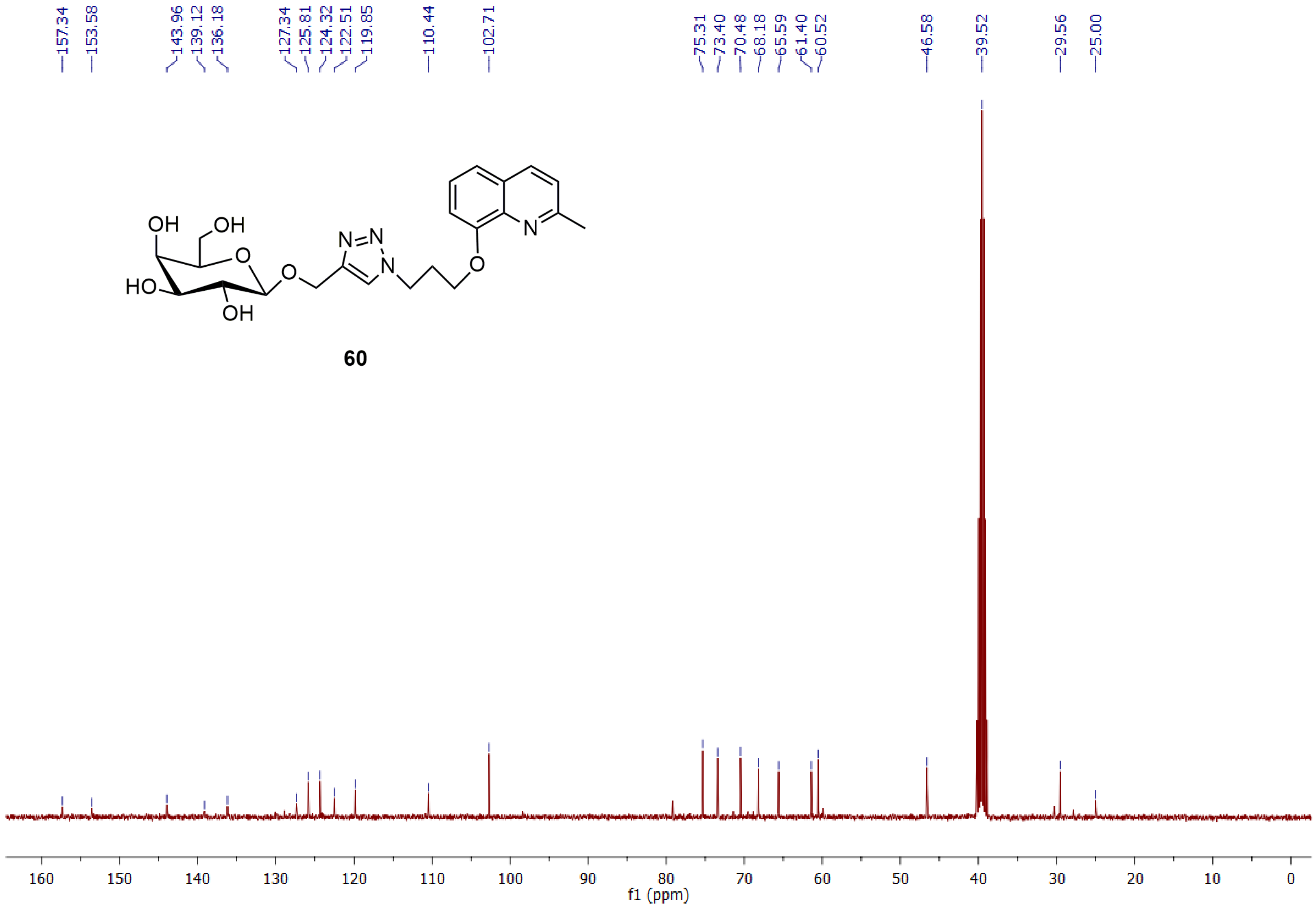

Fig. S112: ^{13}C NMR spectrum of glycoconjugate **60**.

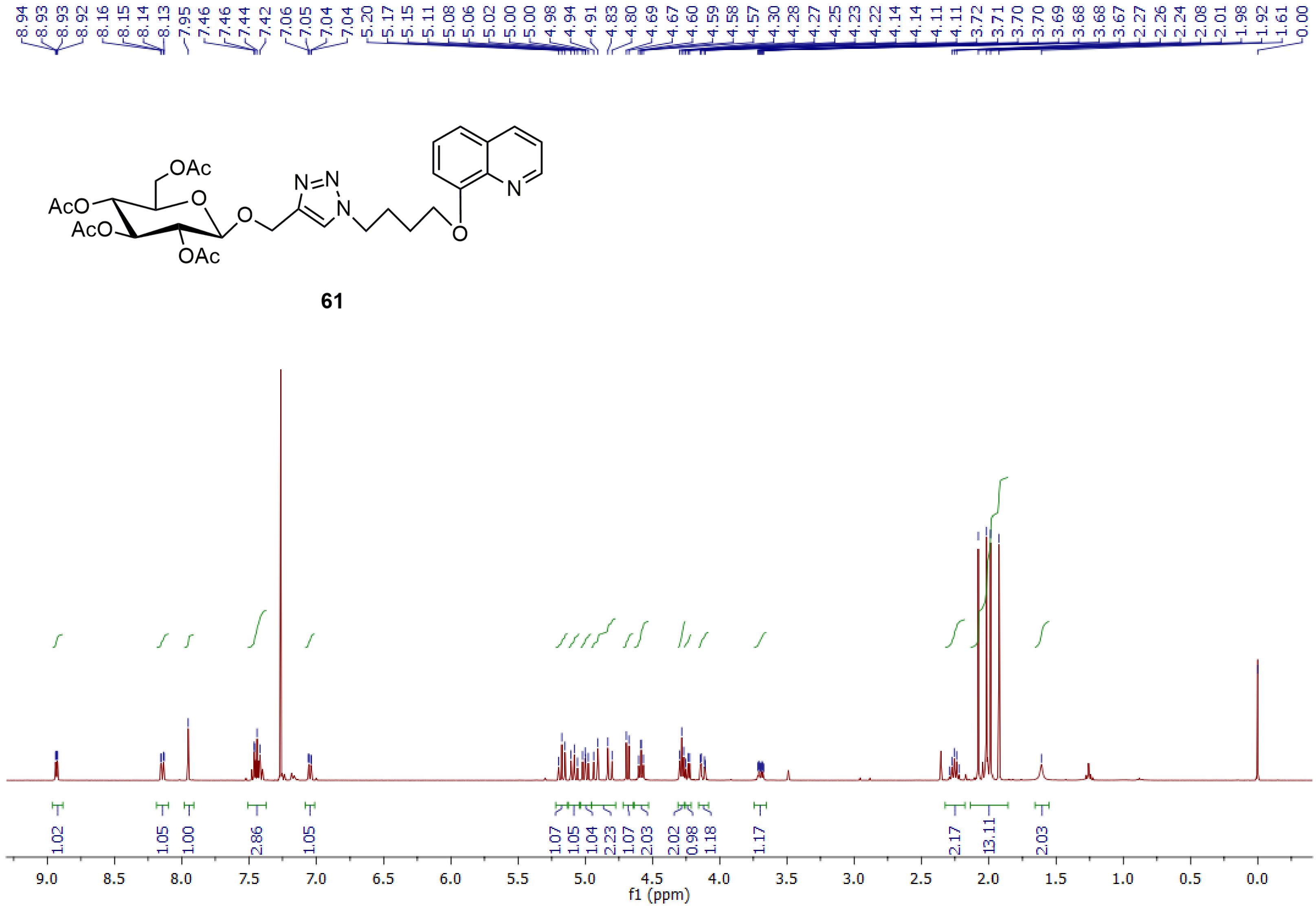

Fig. S113: ^{1}H NMR spectrum of glycoconjugate **61**.

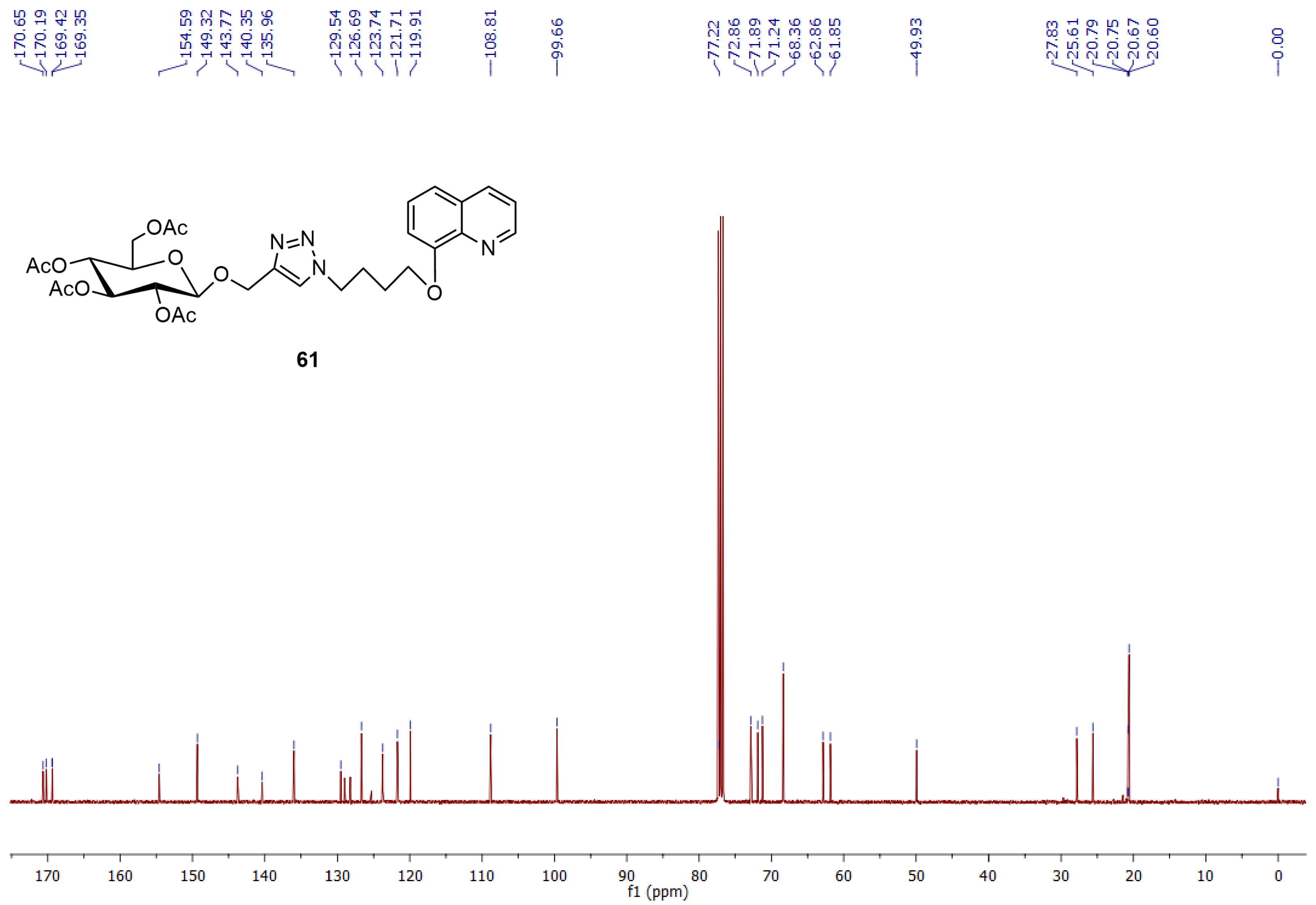

Fig. S114: ^{13}C NMR spectrum of glycoconjugate **61**.

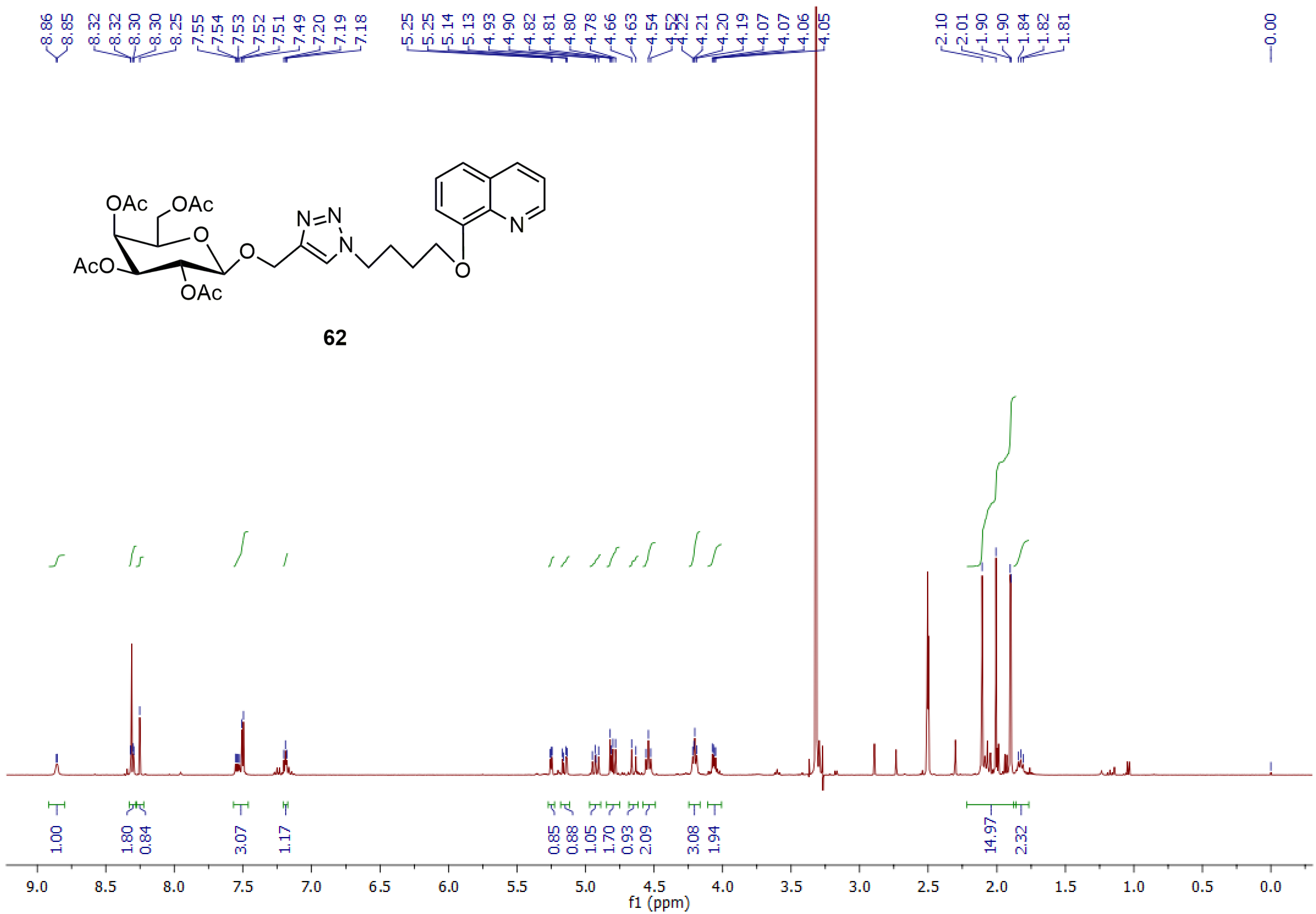

Fig. S115: ^{1}H NMR spectrum of glycoconjugate **62**.

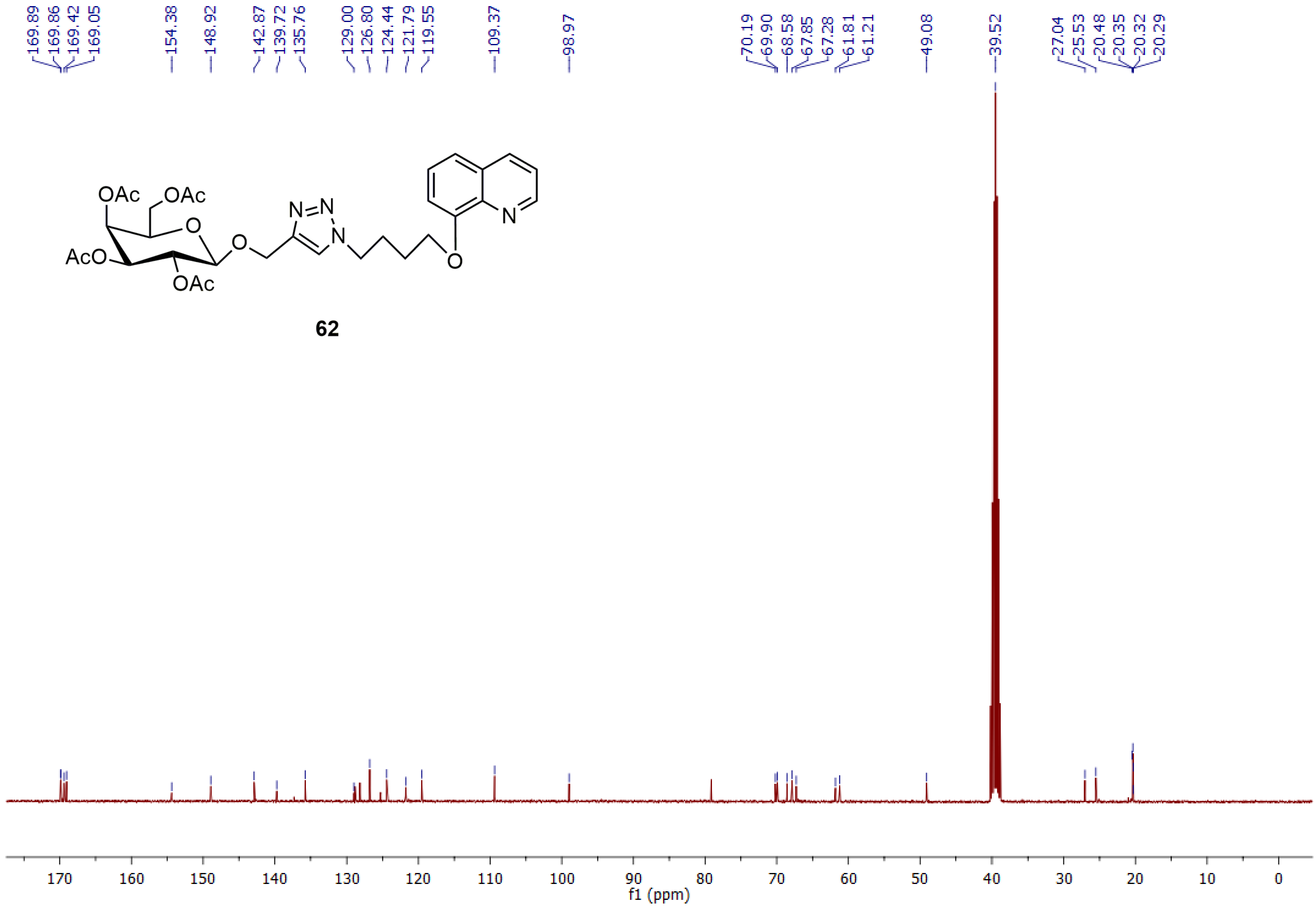

Fig. S116: ^{13}C NMR spectrum of glycoconjugate **62**.

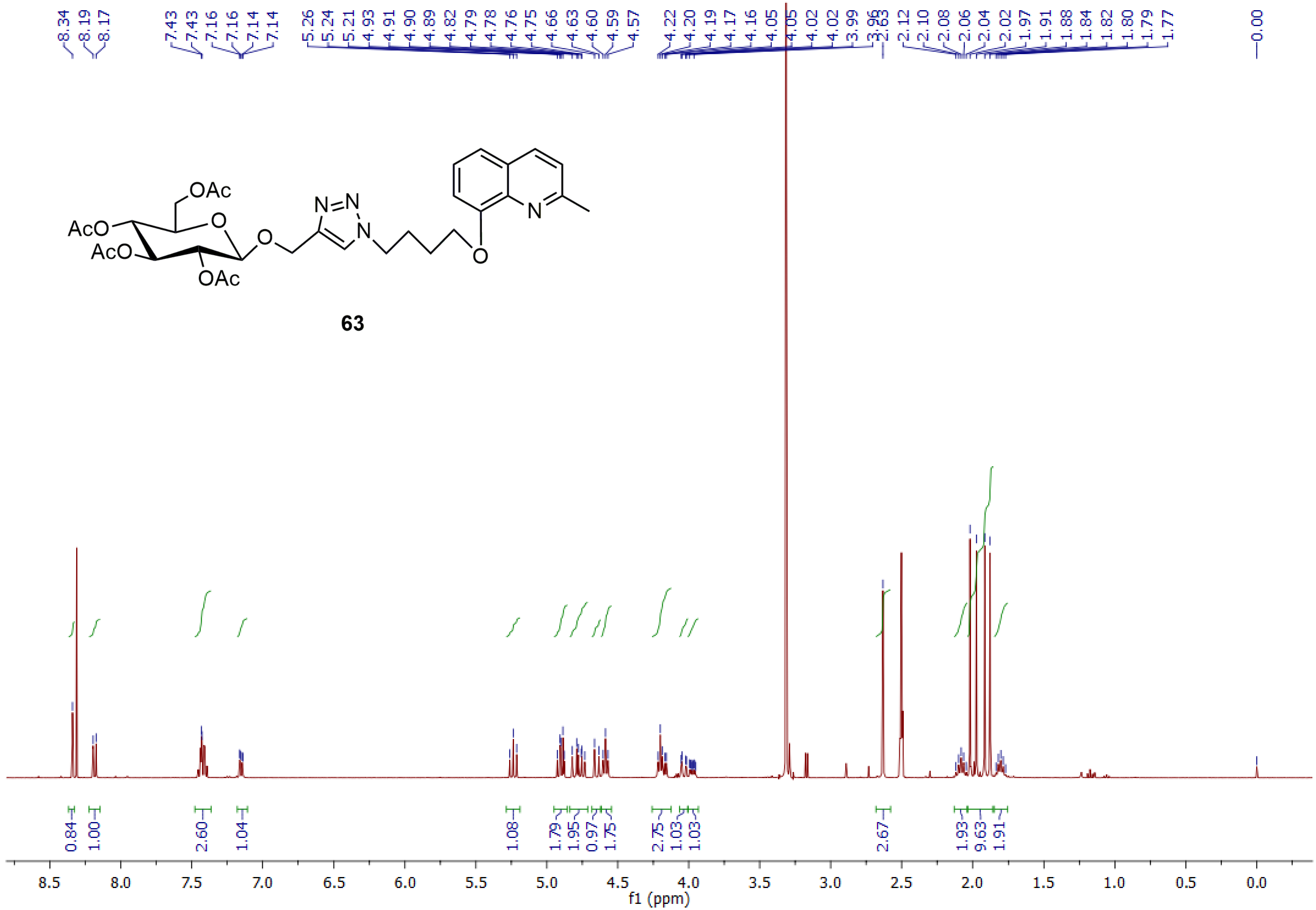

Fig. S117: ^{1}H NMR spectrum of glycoconjugate **63**.

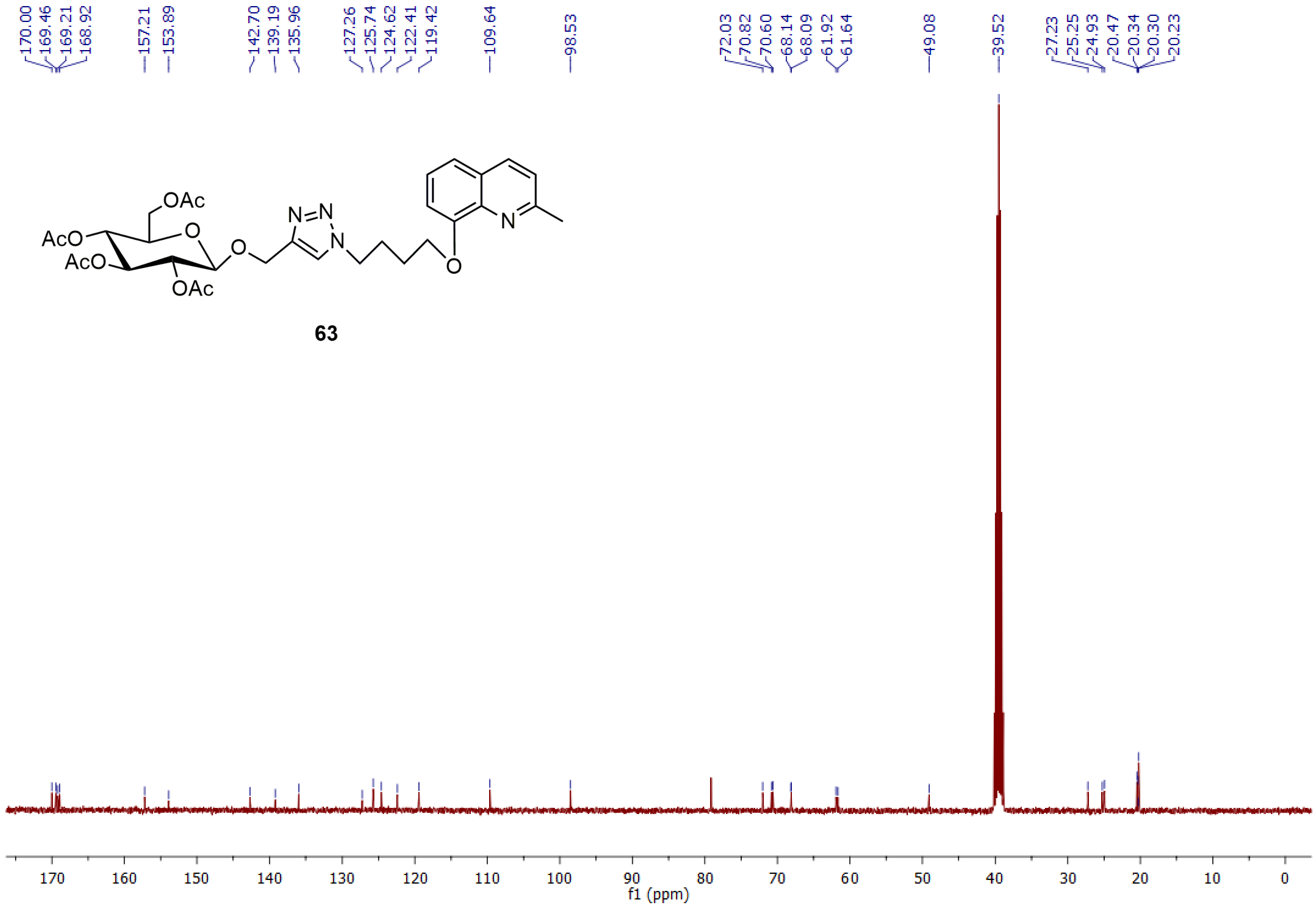

Fig. S118: ^{13}C NMR spectrum of glycoconjugate **63**.

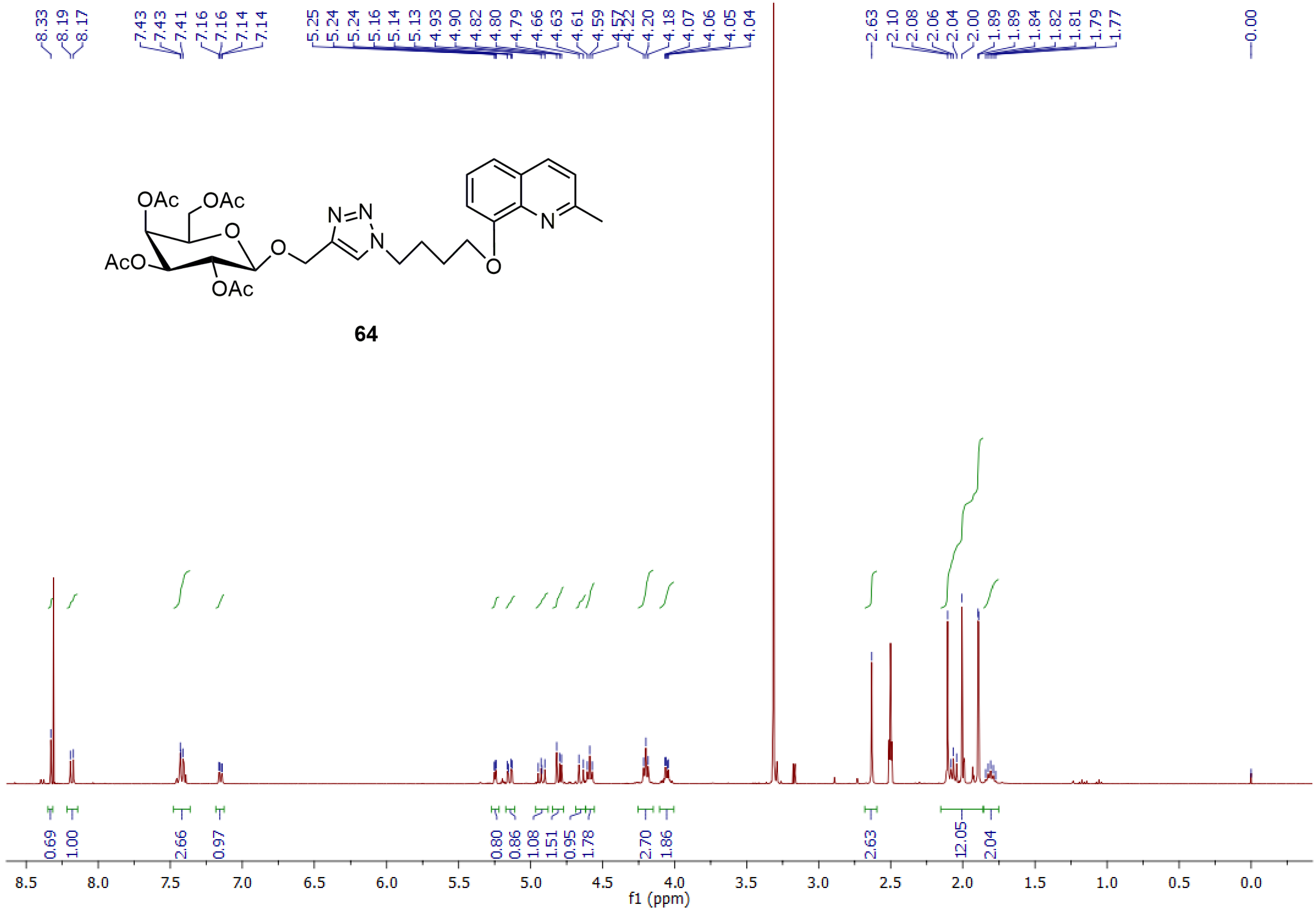

Fig. S119: ^{1}H NMR spectrum of glycoconjugate **64**.

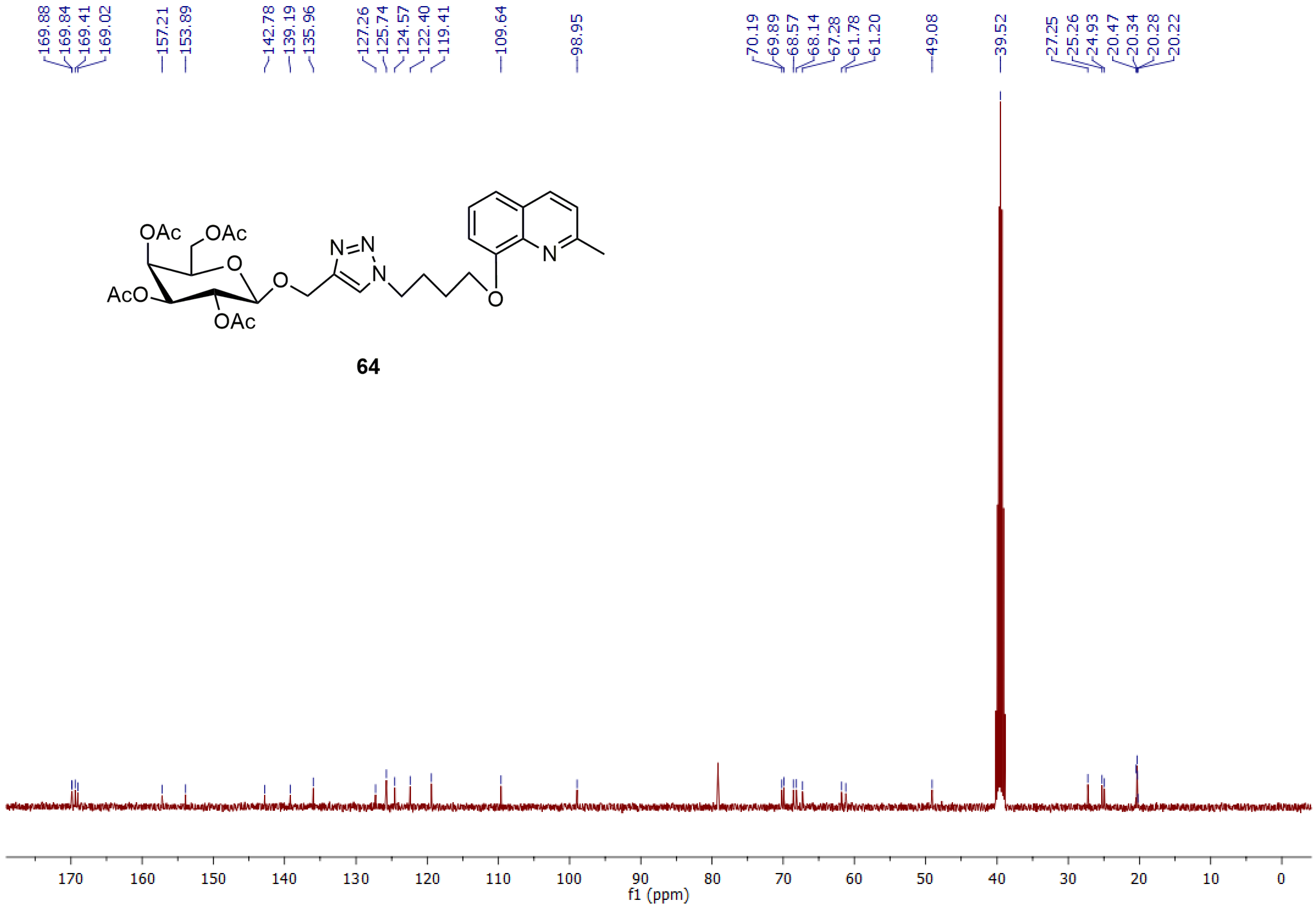

Fig. S120: ^{13}C NMR spectrum of glycoconjugate **64**.

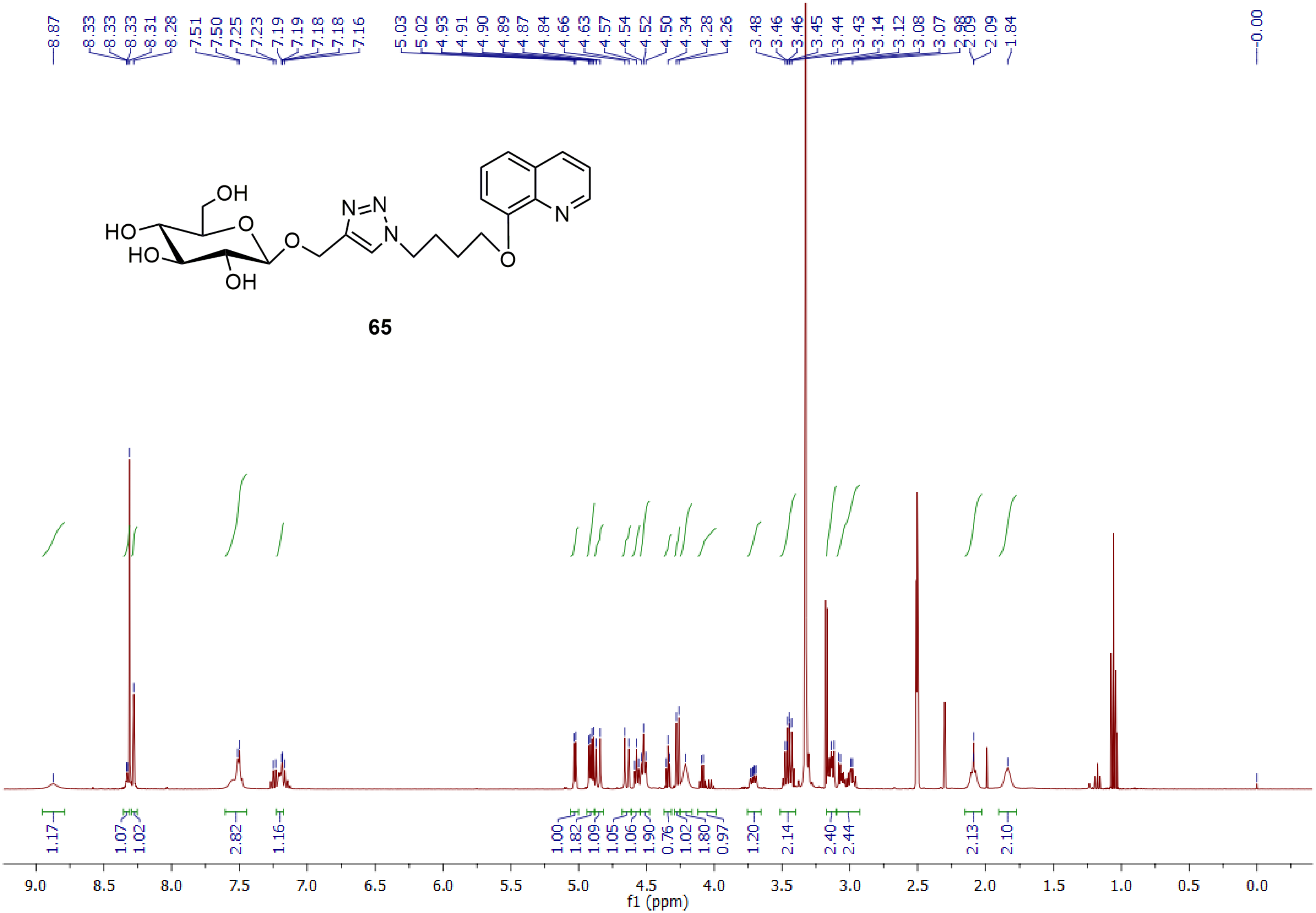

Fig. S121: ^{1}H NMR spectrum of glycoconjugate **65**.

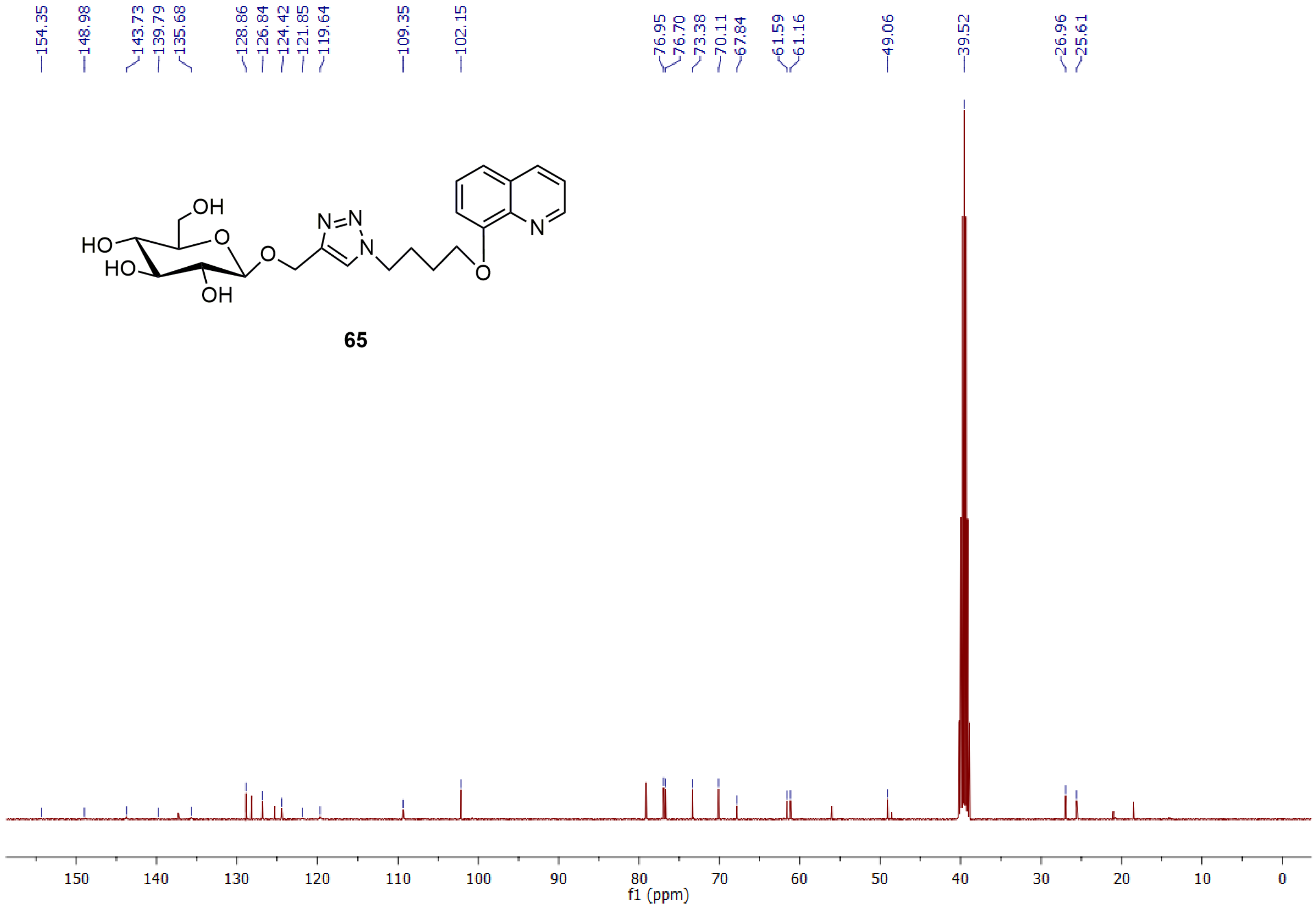

Fig. S122: ^{13}C NMR spectrum of glycoconjugate **65**.

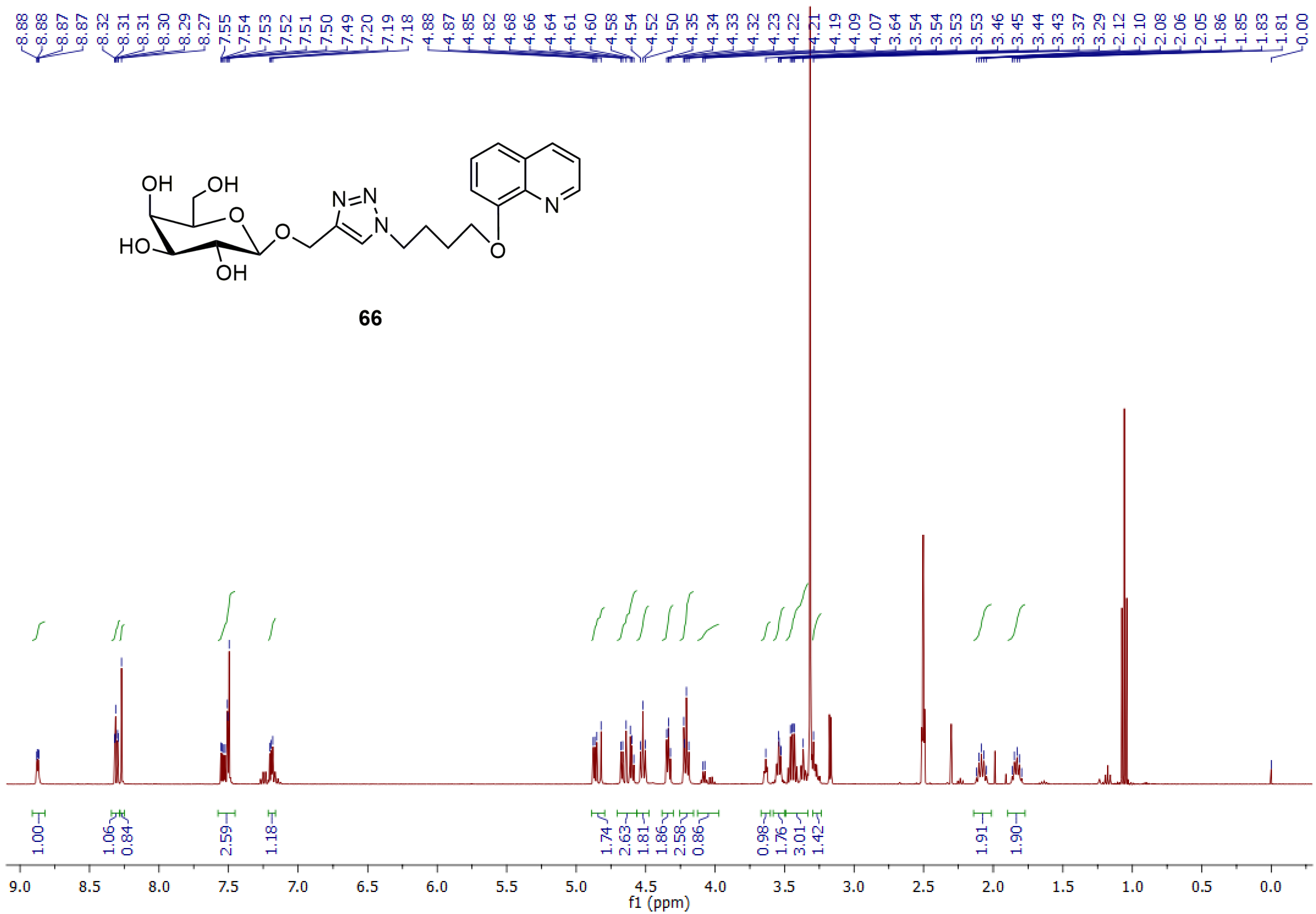

Fig. S123: ^{1}H NMR spectrum of glycoconjugate **66**.

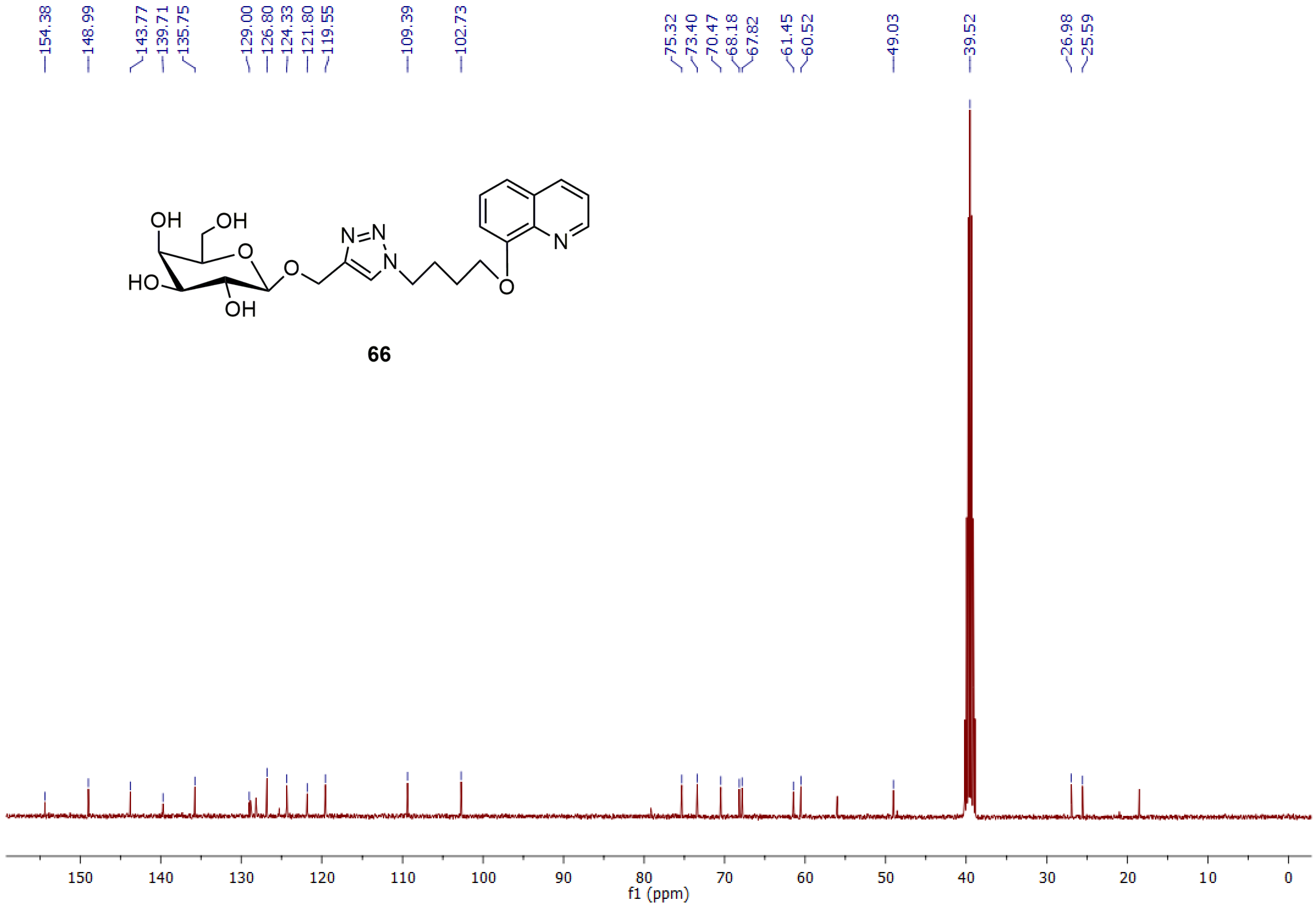

Fig. S124: ^{13}C NMR spectrum of glycoconjugate **66**.

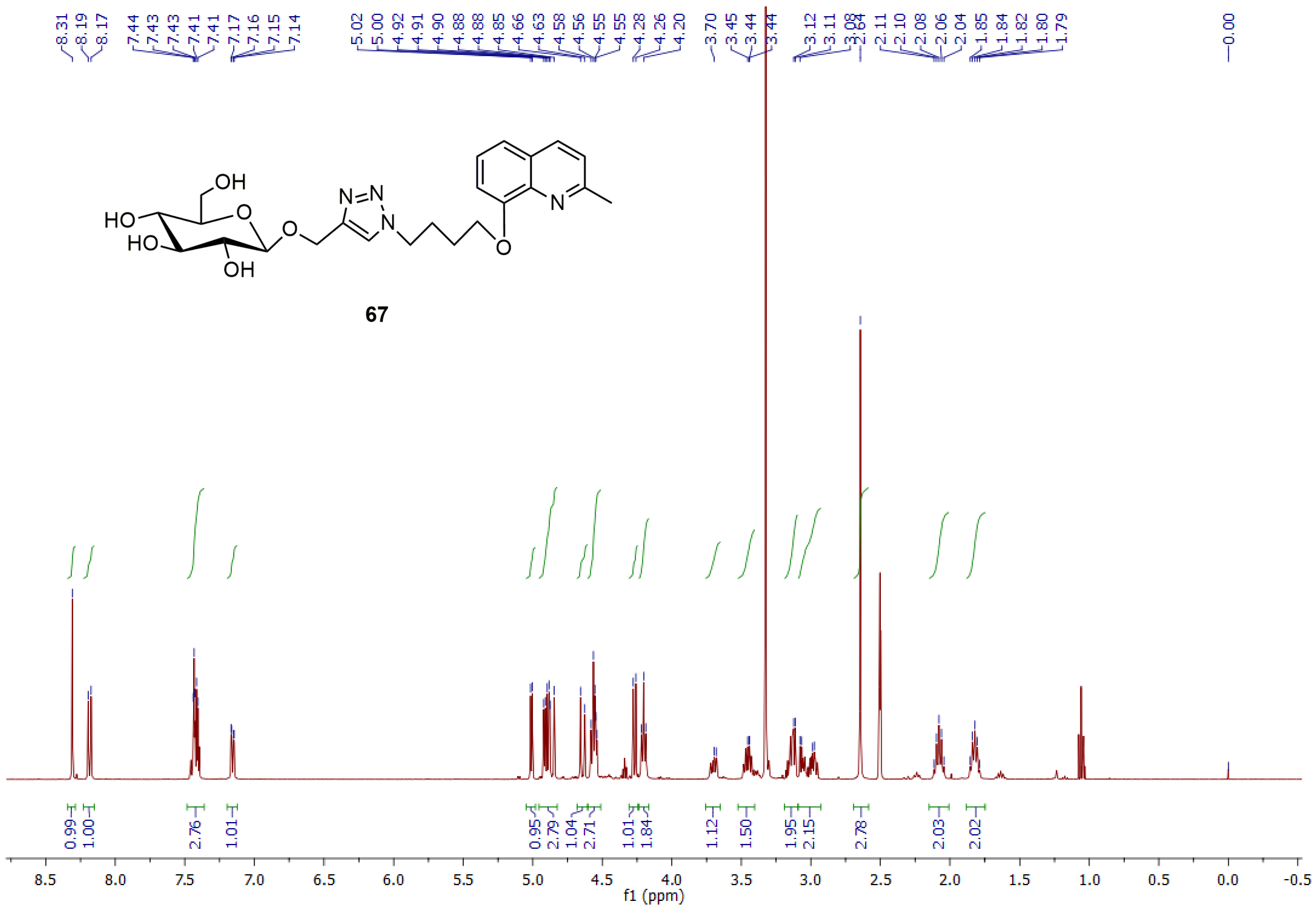

Fig. S125: ^{1}H NMR spectrum of glycoconjugate **67**.

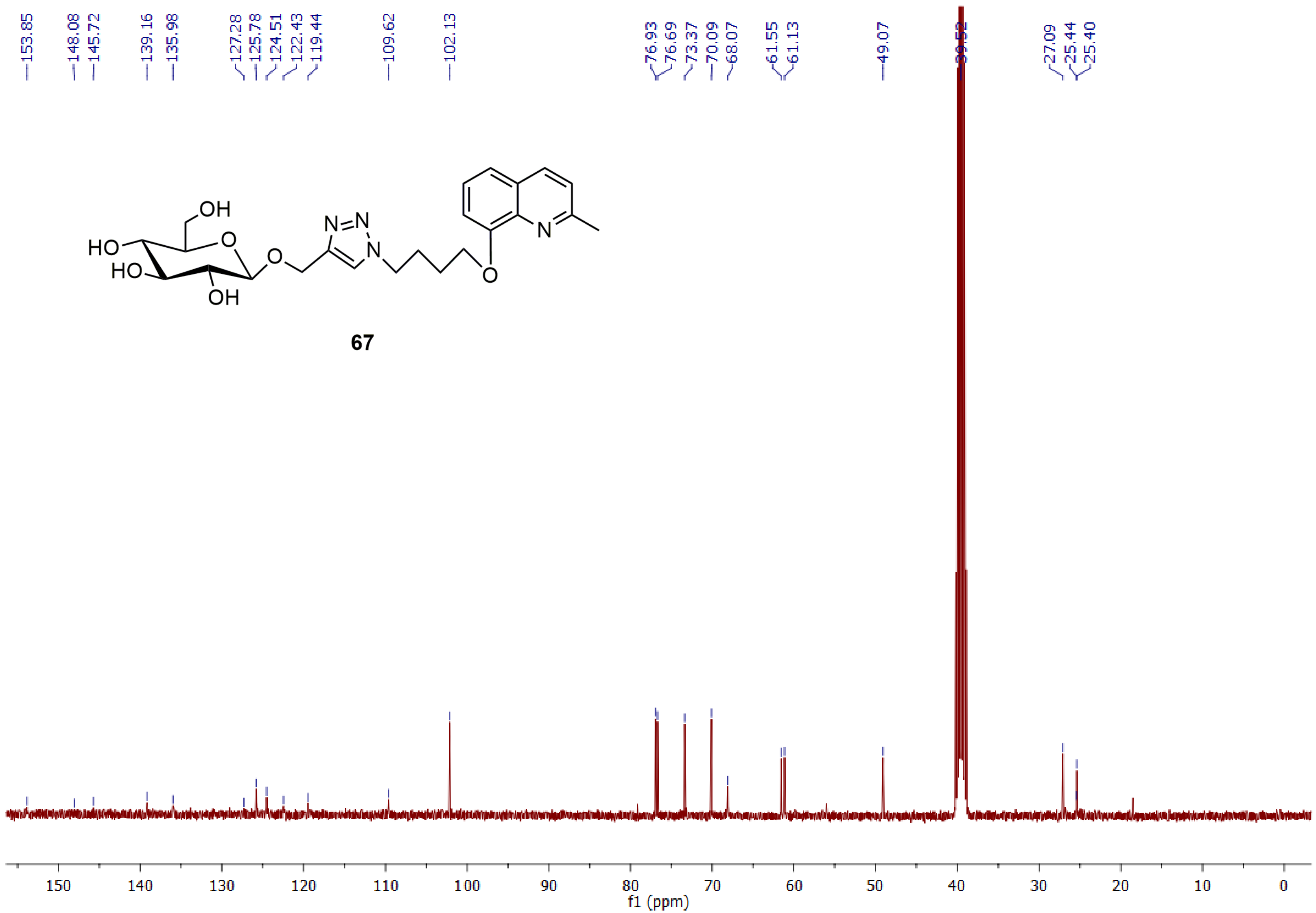

Fig. S126: ^{13}C NMR spectrum of glycoconjugate **67**.

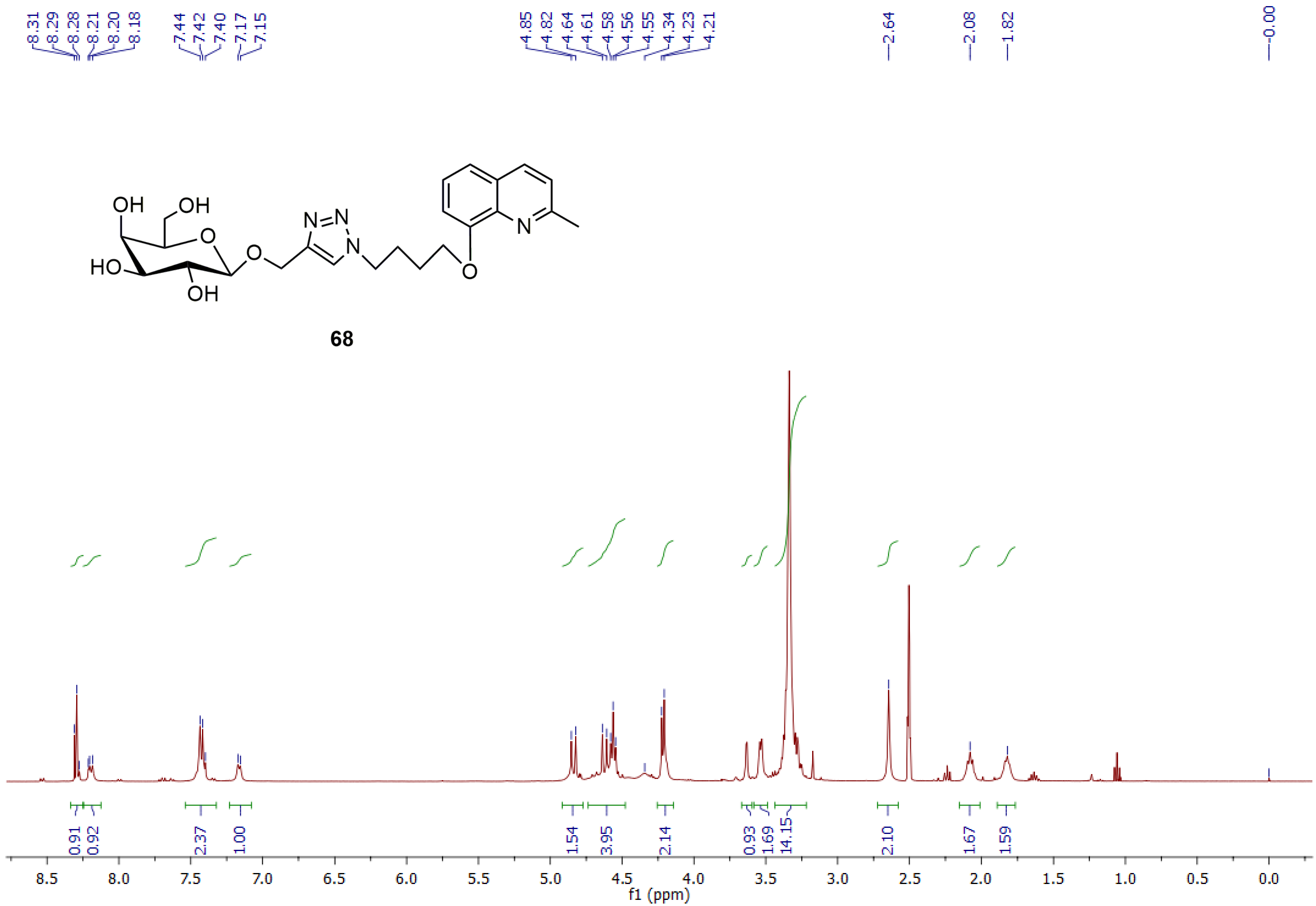

Fig. S127: ^{1}H NMR spectrum of glycoconjugate **68**.

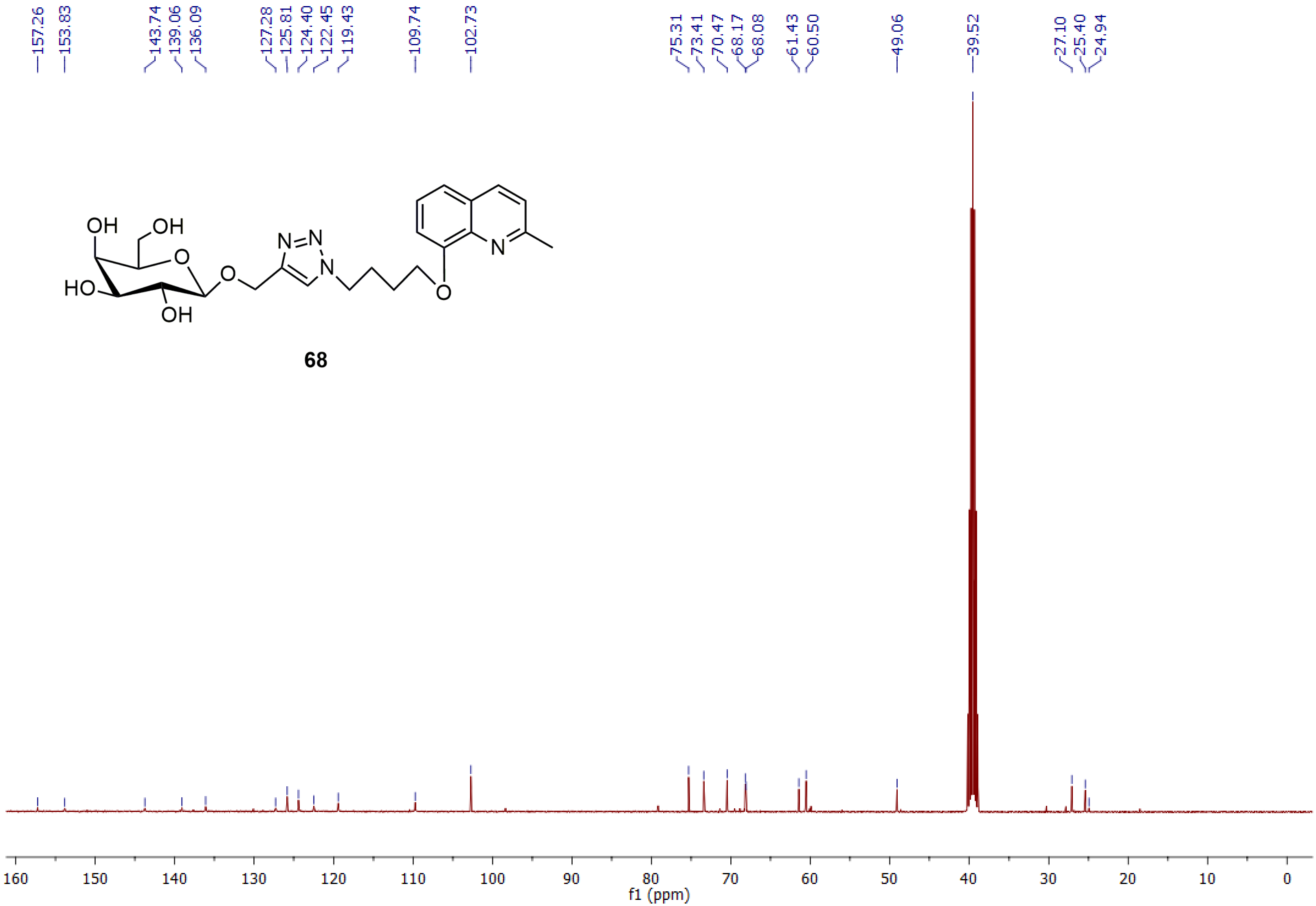

Fig. S128: ^{13}C NMR spectrum of glycoconjugate **68**.

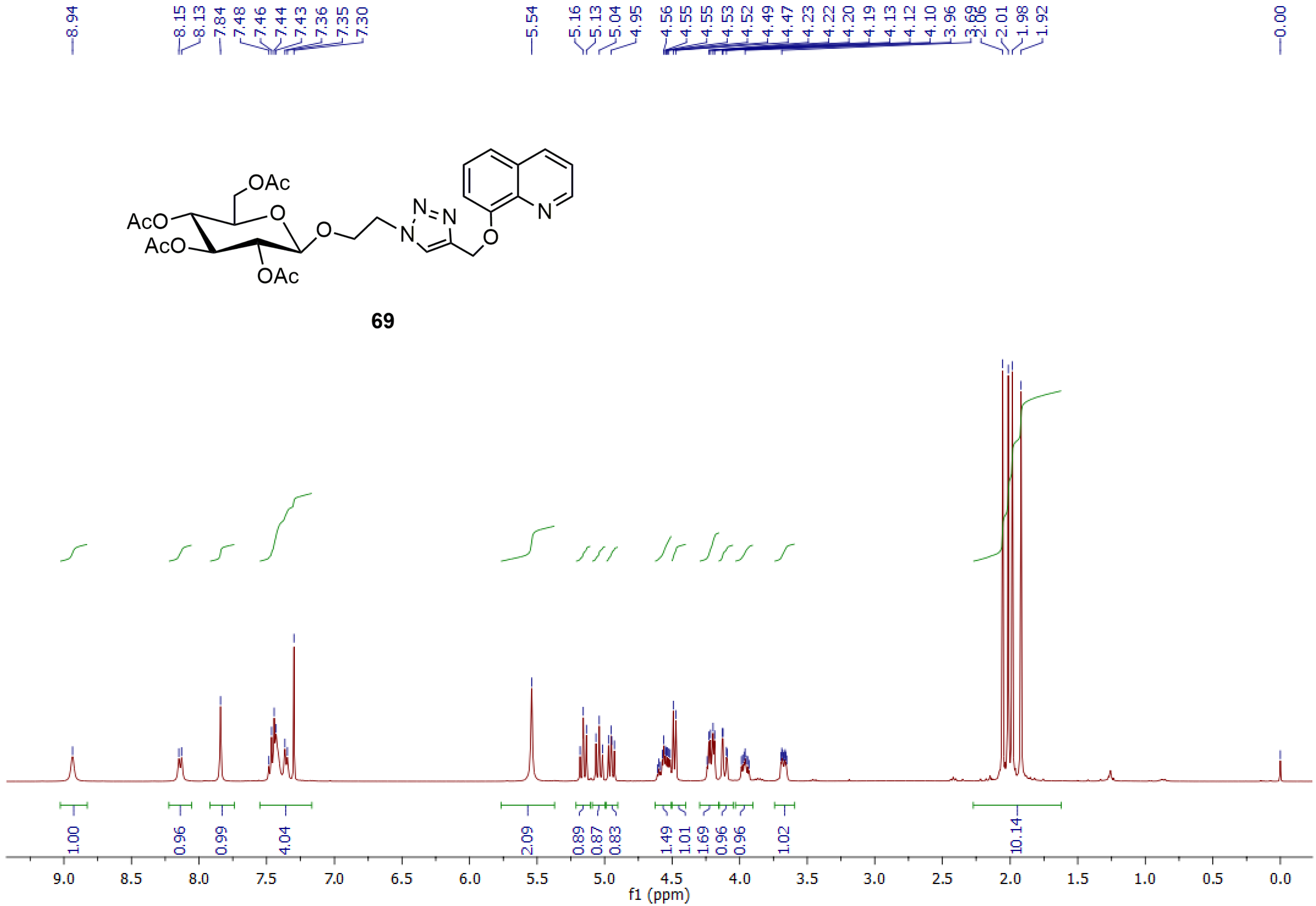

Fig. S129: ^{1}H NMR spectrum of glycoconjugate **69**.

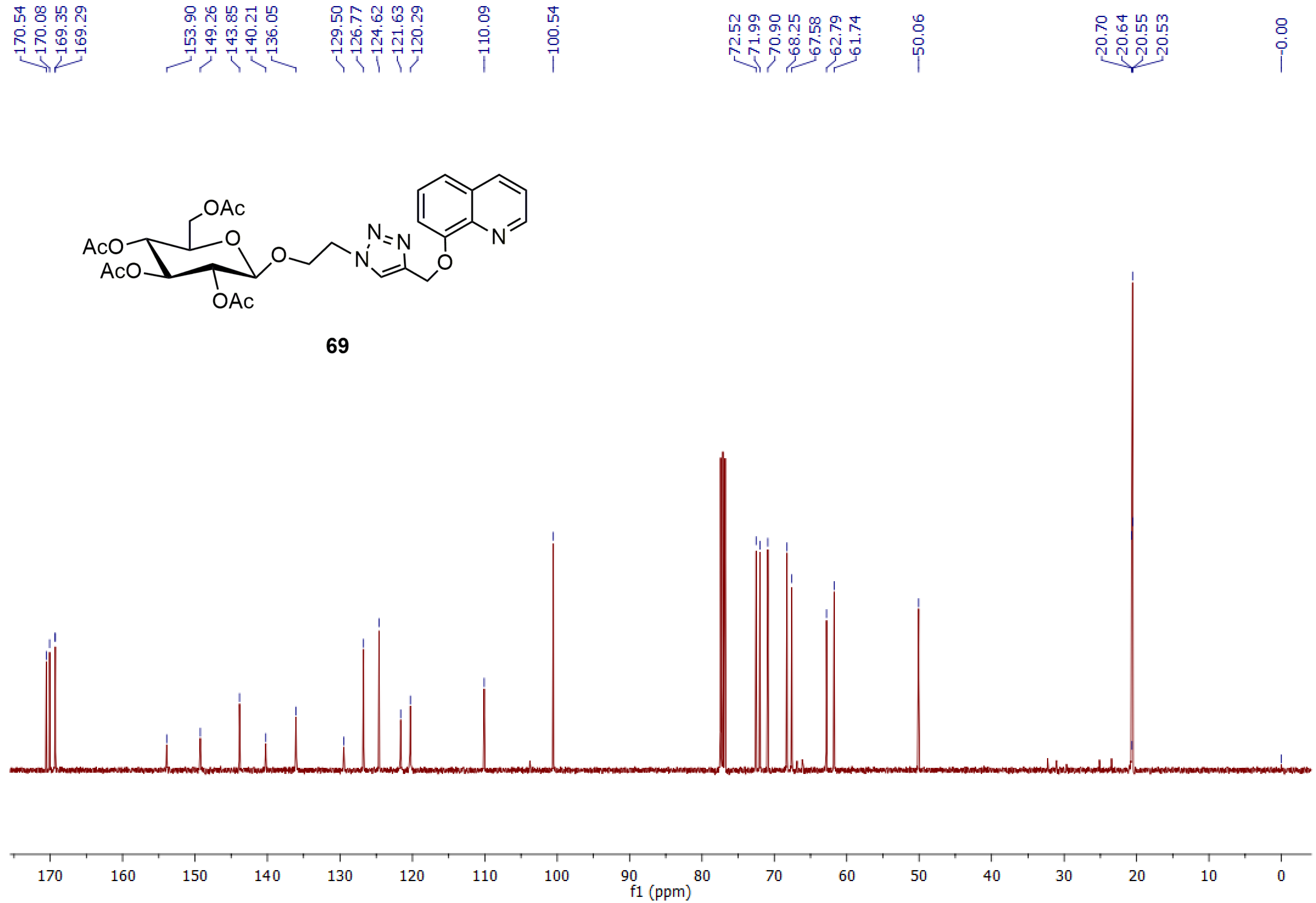

Fig. S130: ^{13}C NMR spectrum of glycoconjugate **69**.

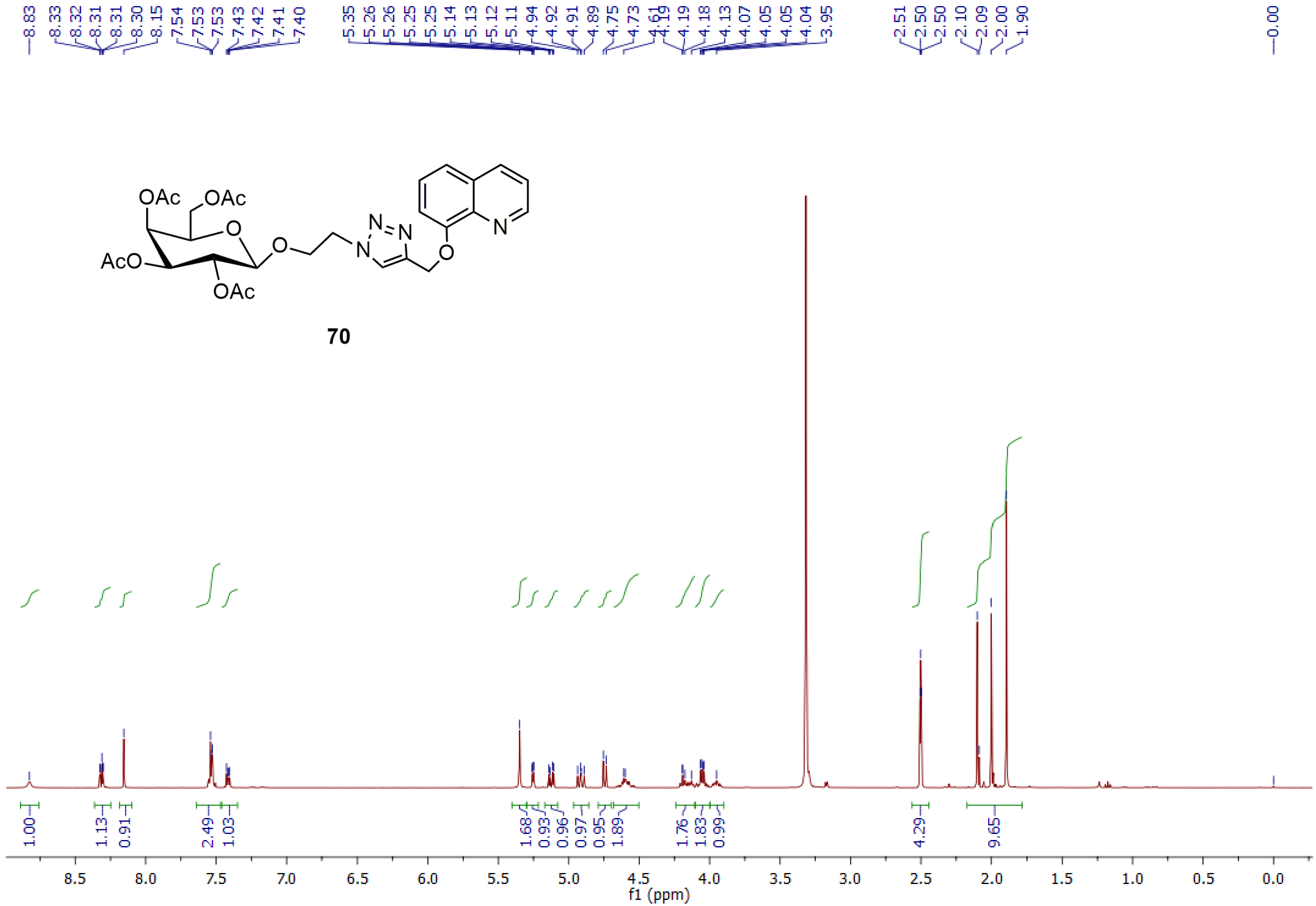

Fig. S131: ^{1}H NMR spectrum of glycoconjugate **70**.

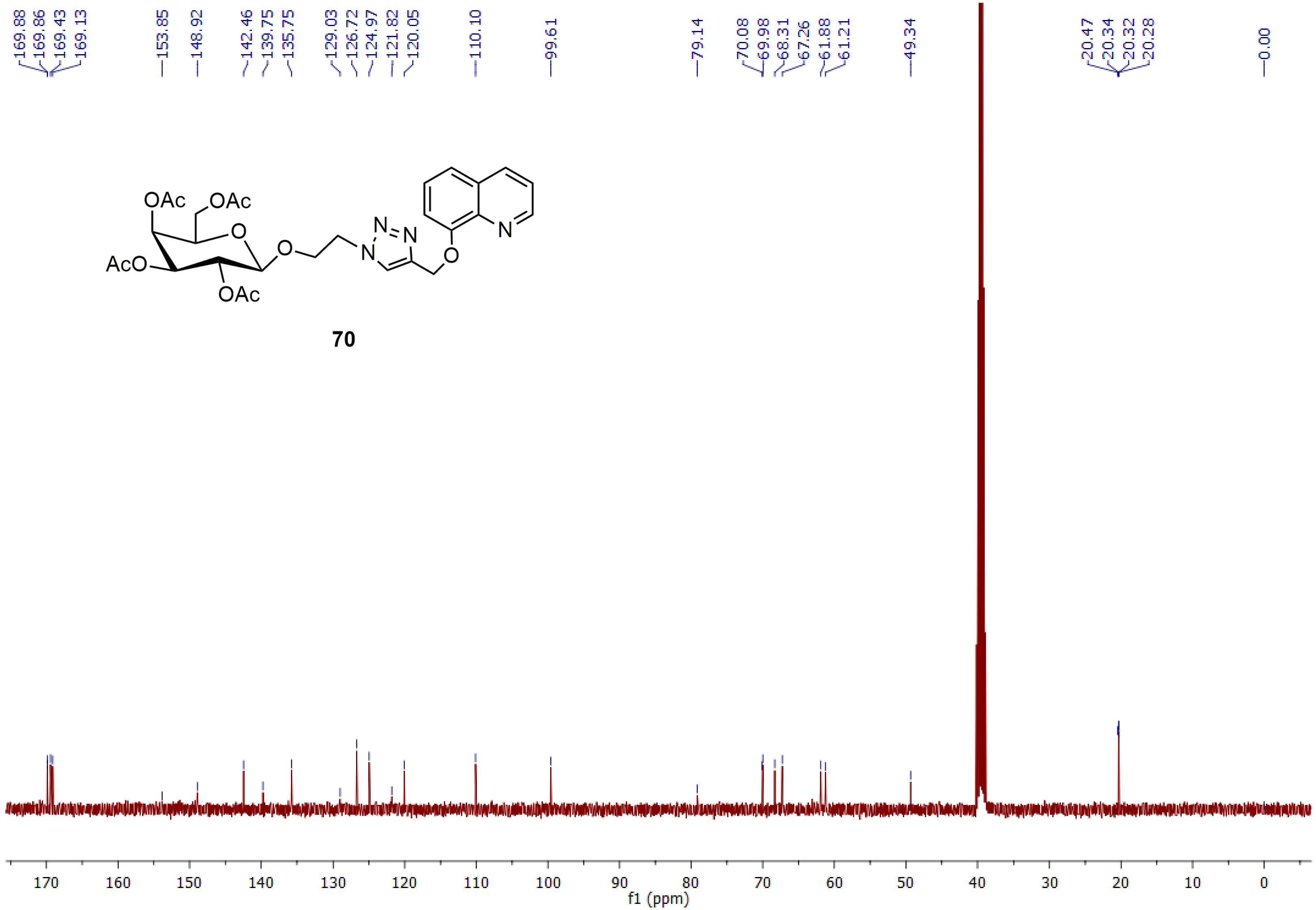

Fig. S132: ^{13}C NMR spectrum of glycoconjugate **70**.

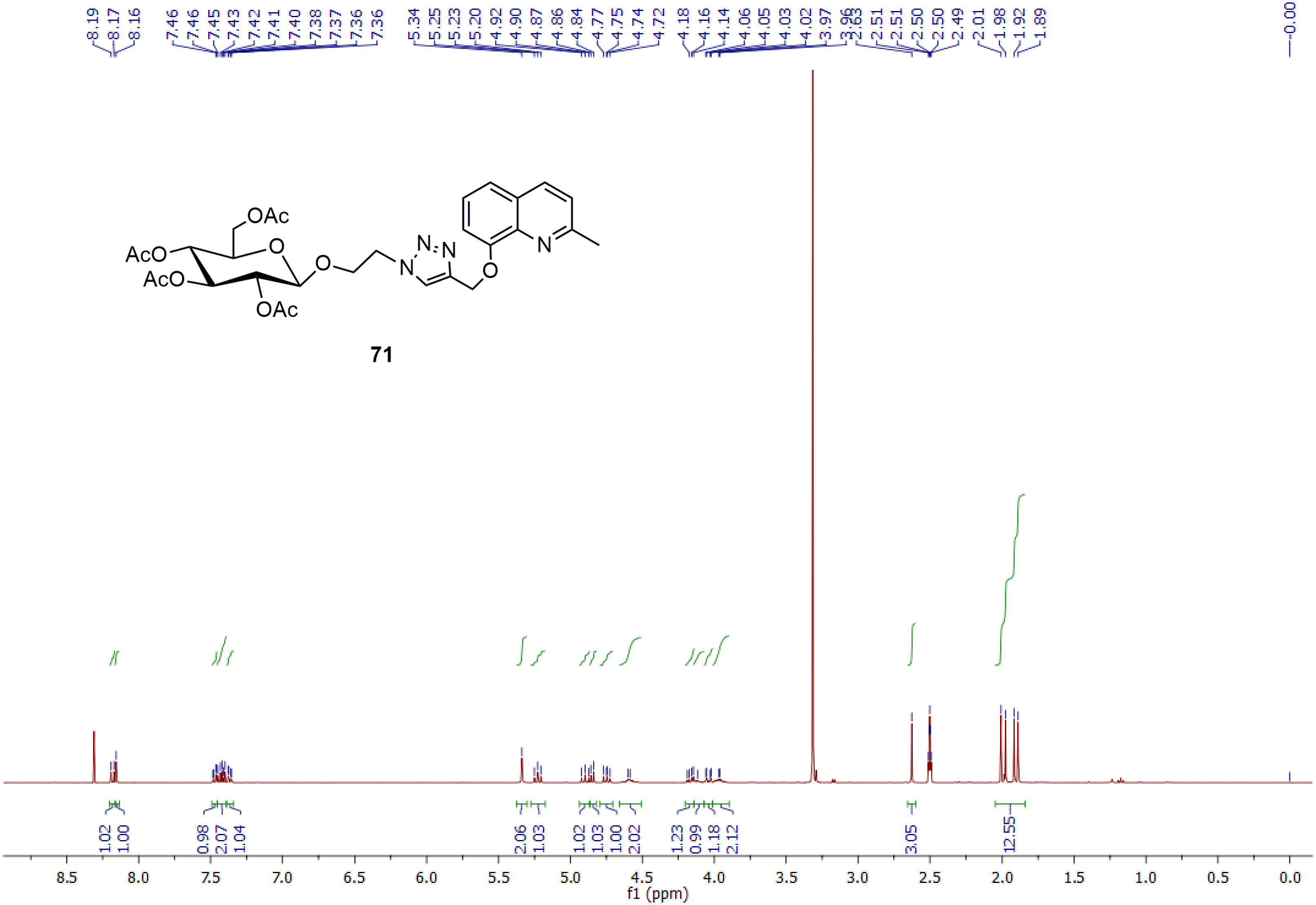

71

Fig. S133: ^{1}H NMR spectrum of glycoconjugate **71**.

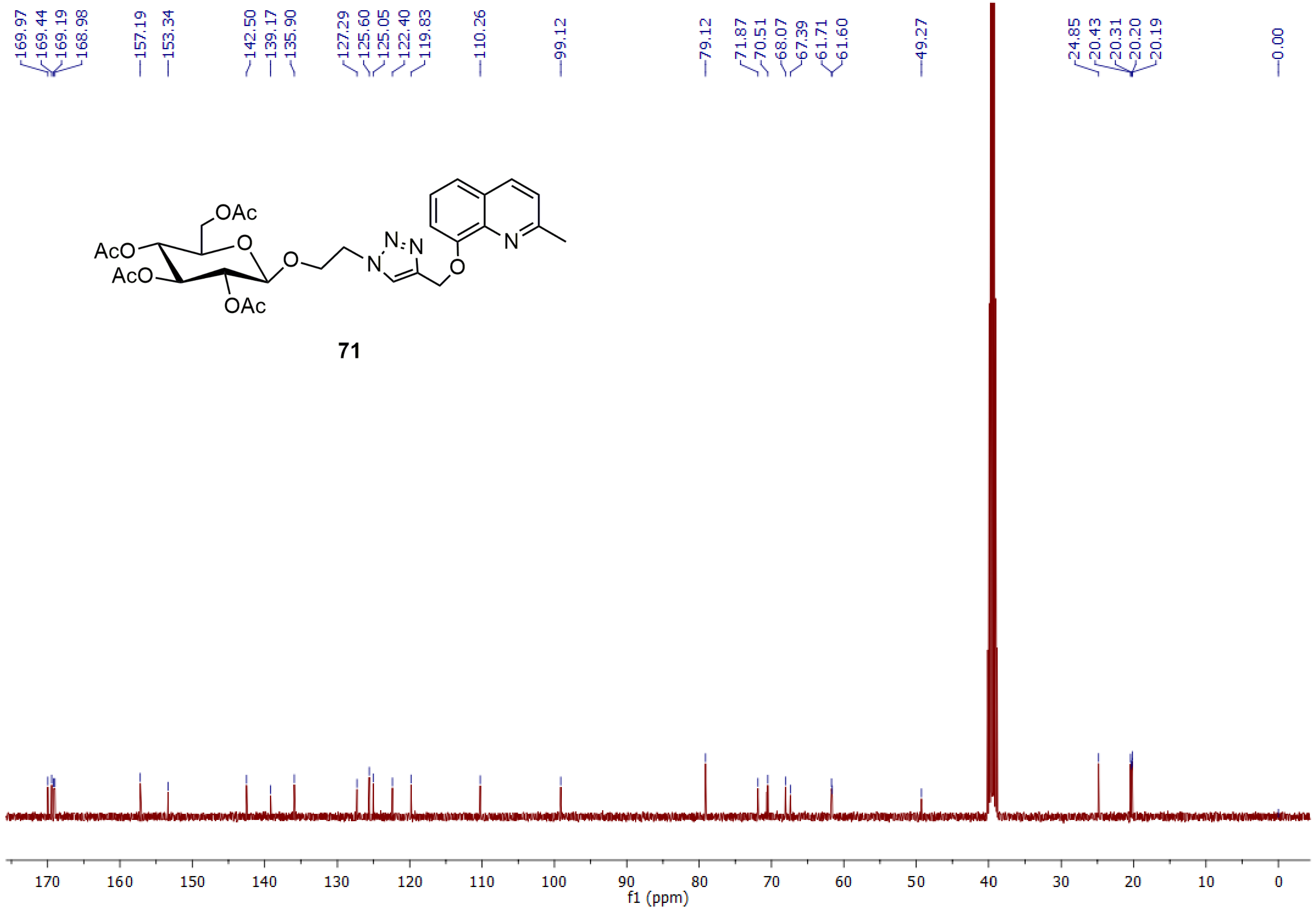

Fig. S134: ^{13}C NMR spectrum of glycoconjugate **71**.

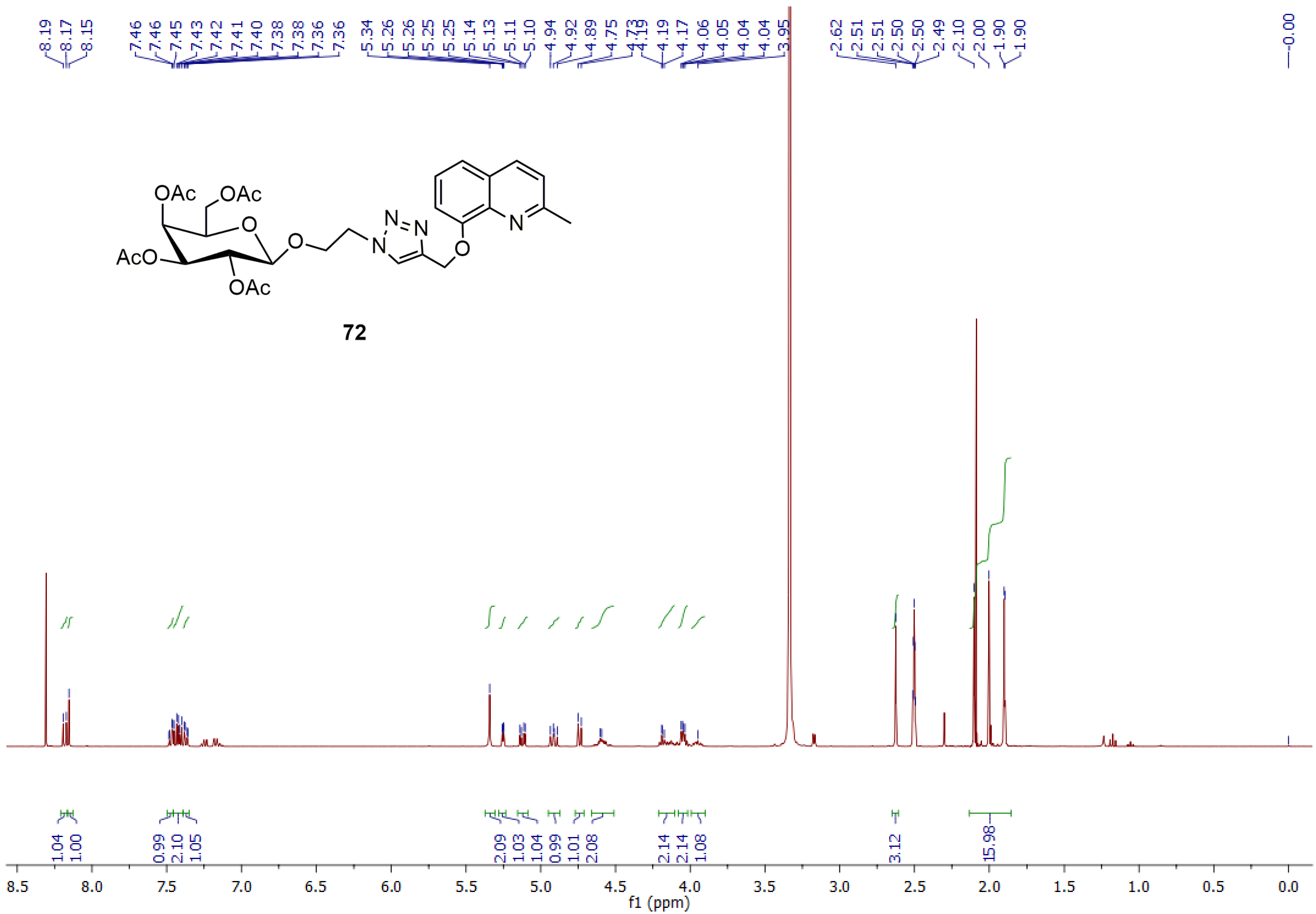

Fig. S135: ^{1}H NMR spectrum of glycoconjugate **72**.

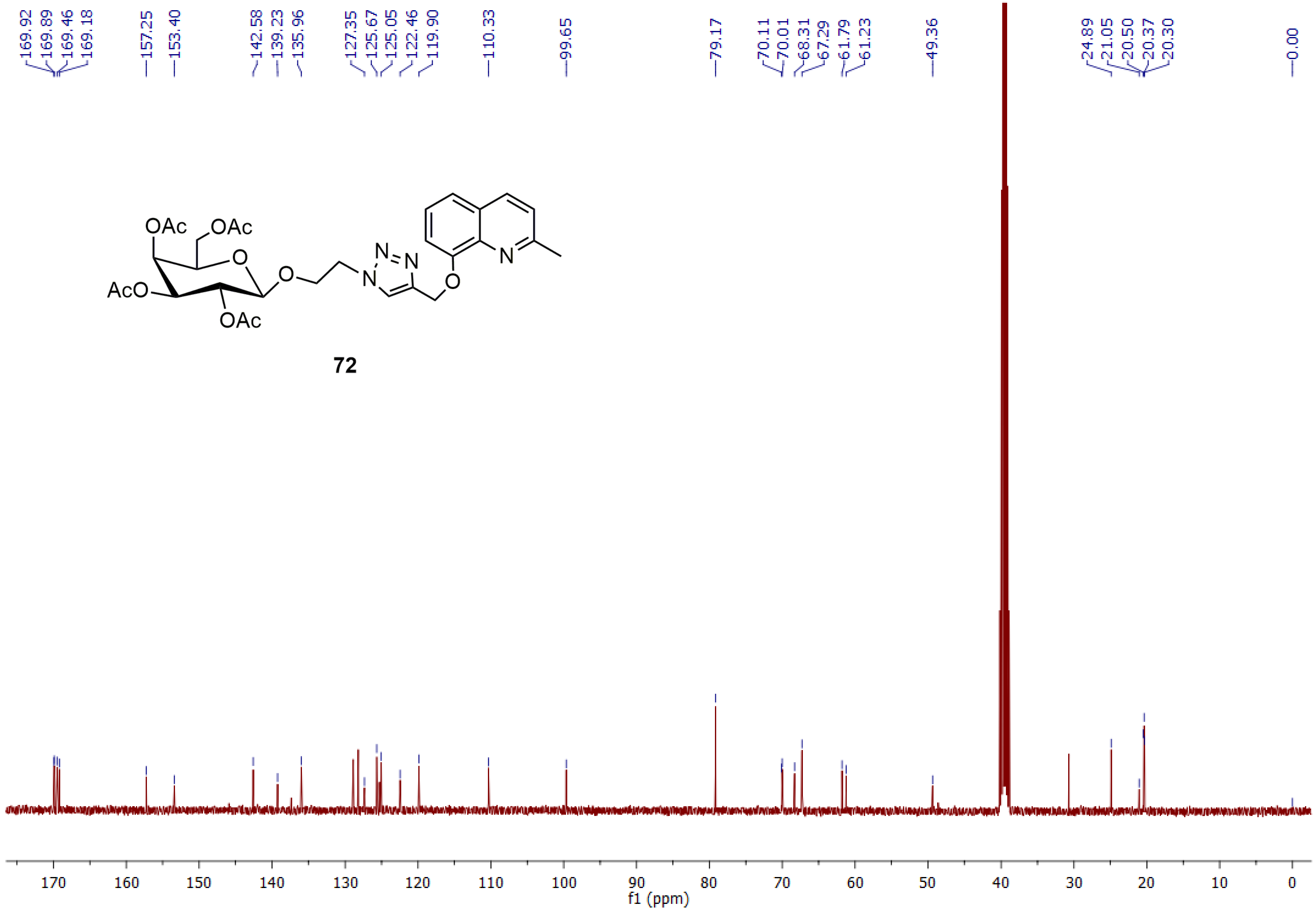

Fig. S136: ^{13}C NMR spectrum of glycoconjugate **72**.

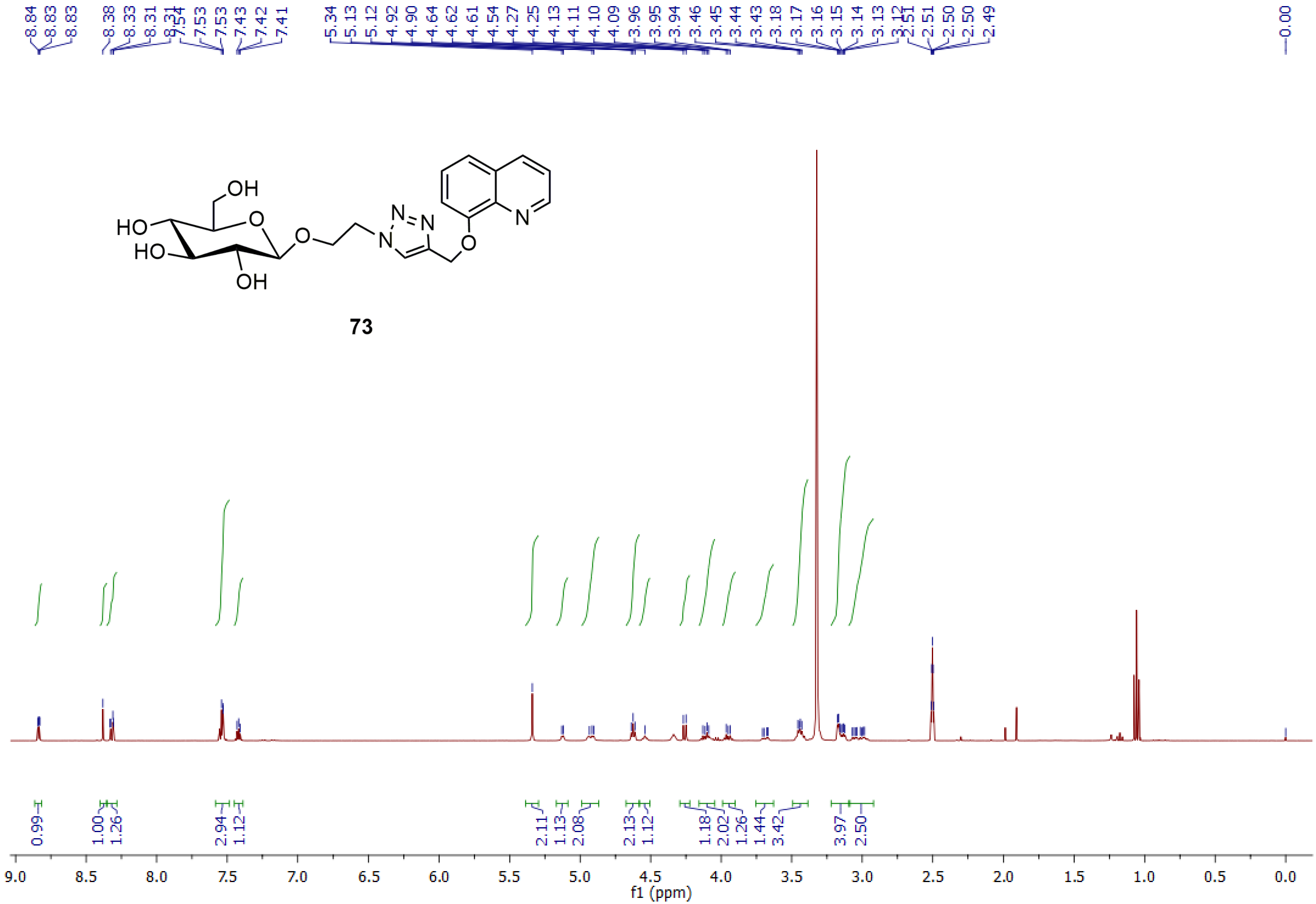

Fig. S137: ^{1}H NMR spectrum of glycoconjugate **73**.

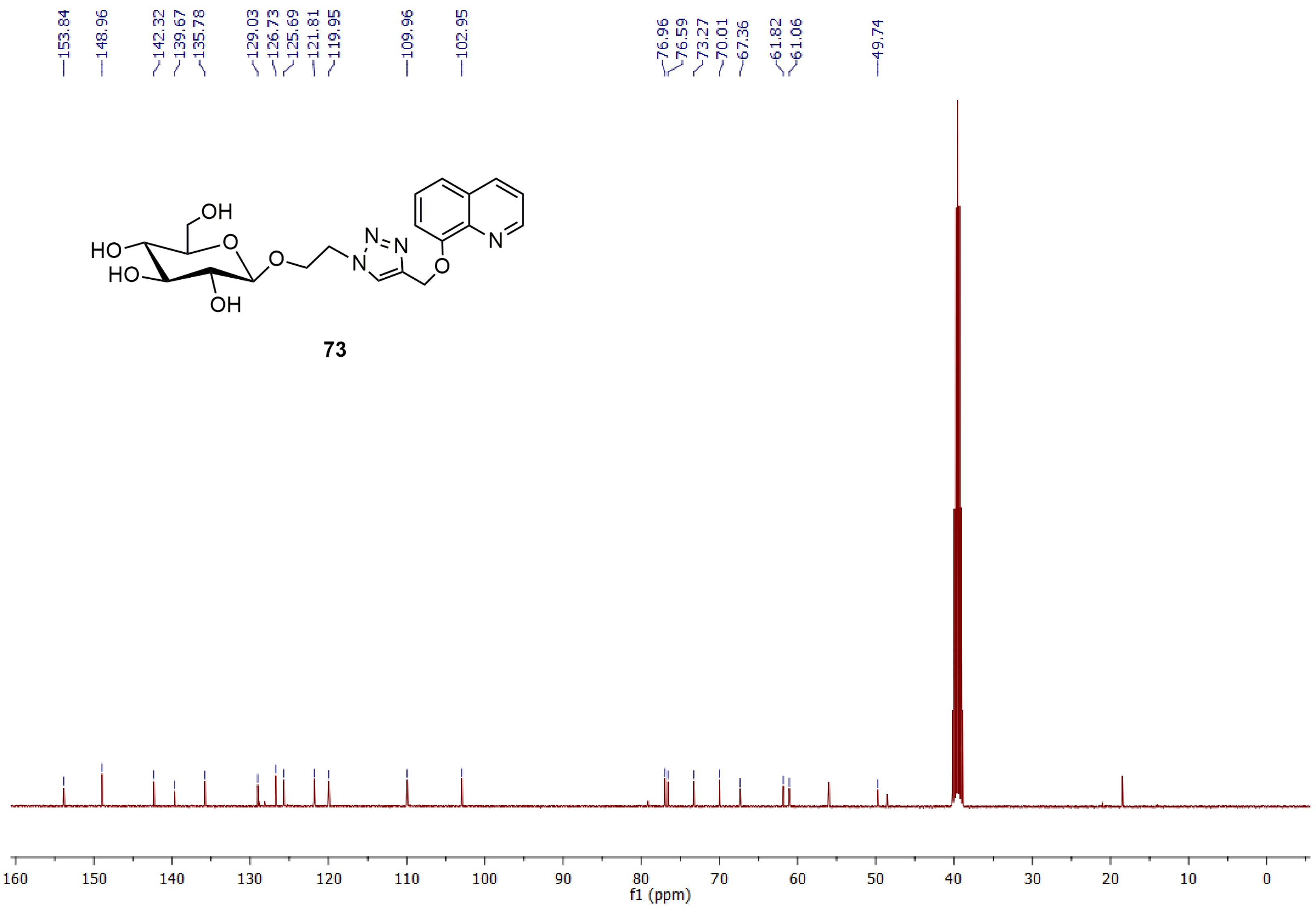

Fig. S138: ^{13}C NMR spectrum of glycoconjugate **73**.

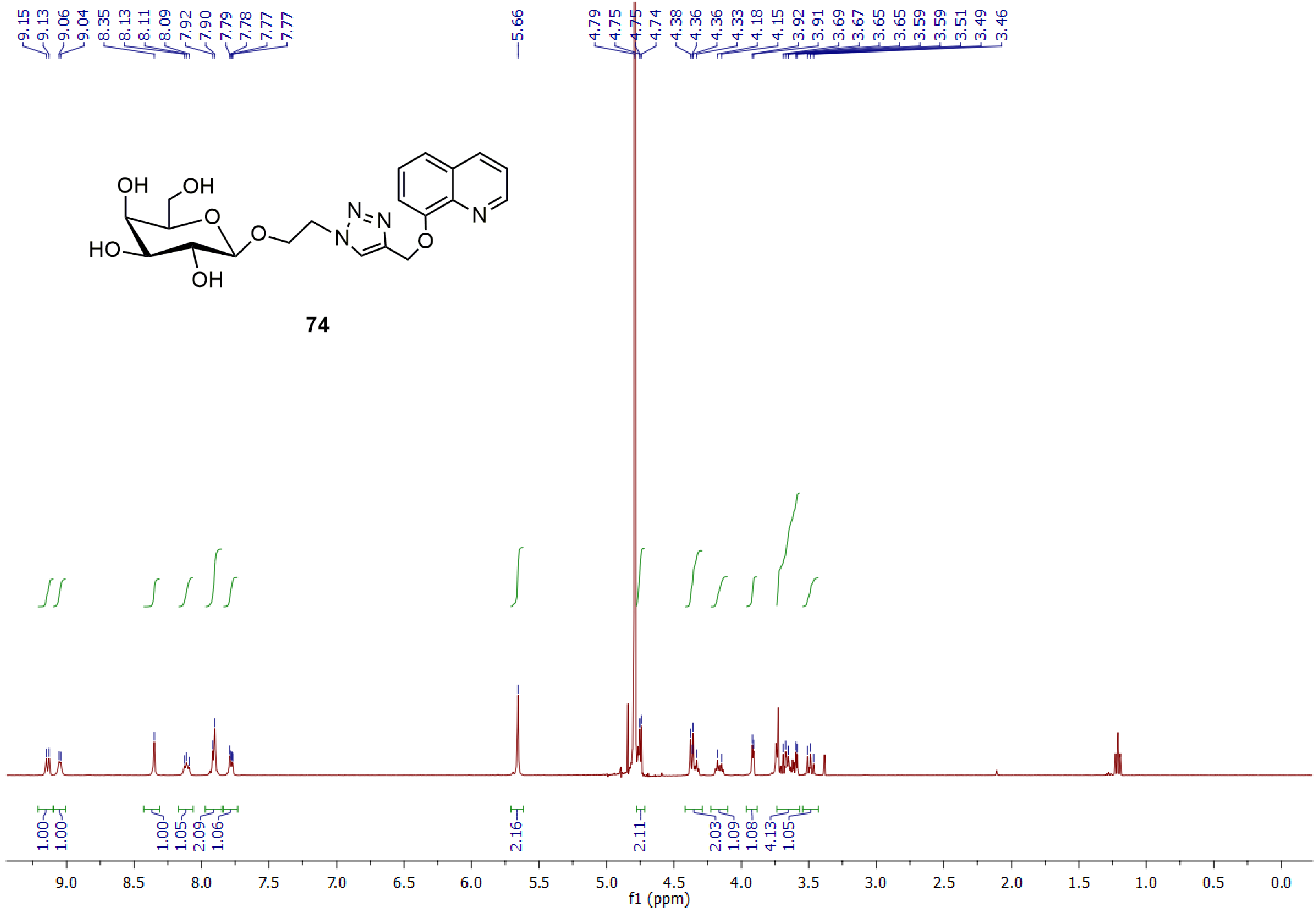

Fig. S139: ^{1}H NMR spectrum of glycoconjugate **74**.

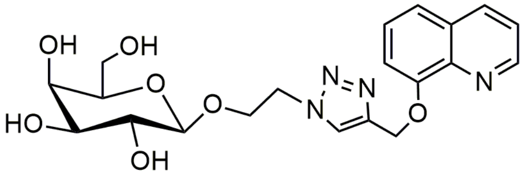

74

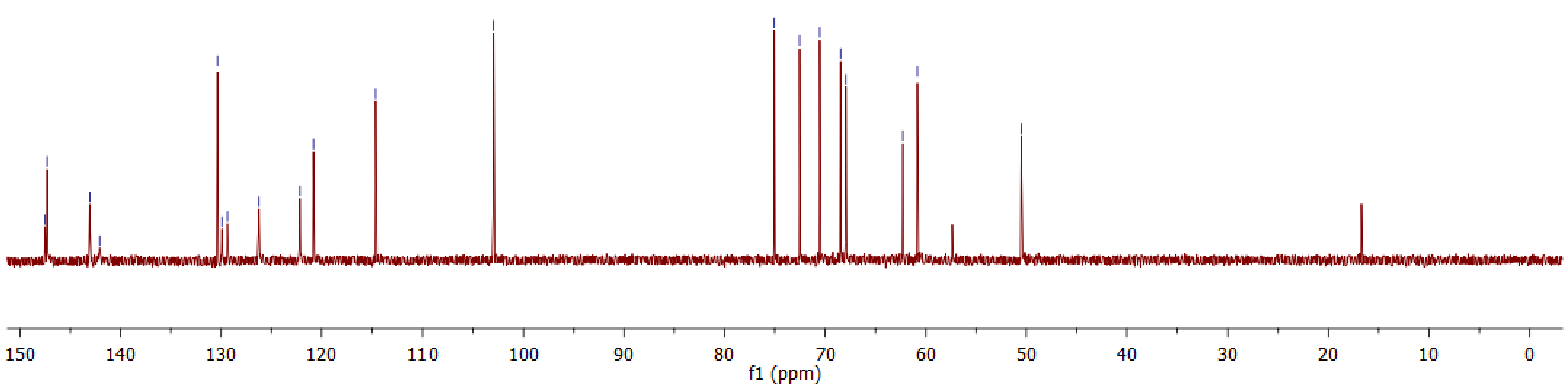

Fig. S140: ^{13}C NMR spectrum of glycoconjugate **74**.

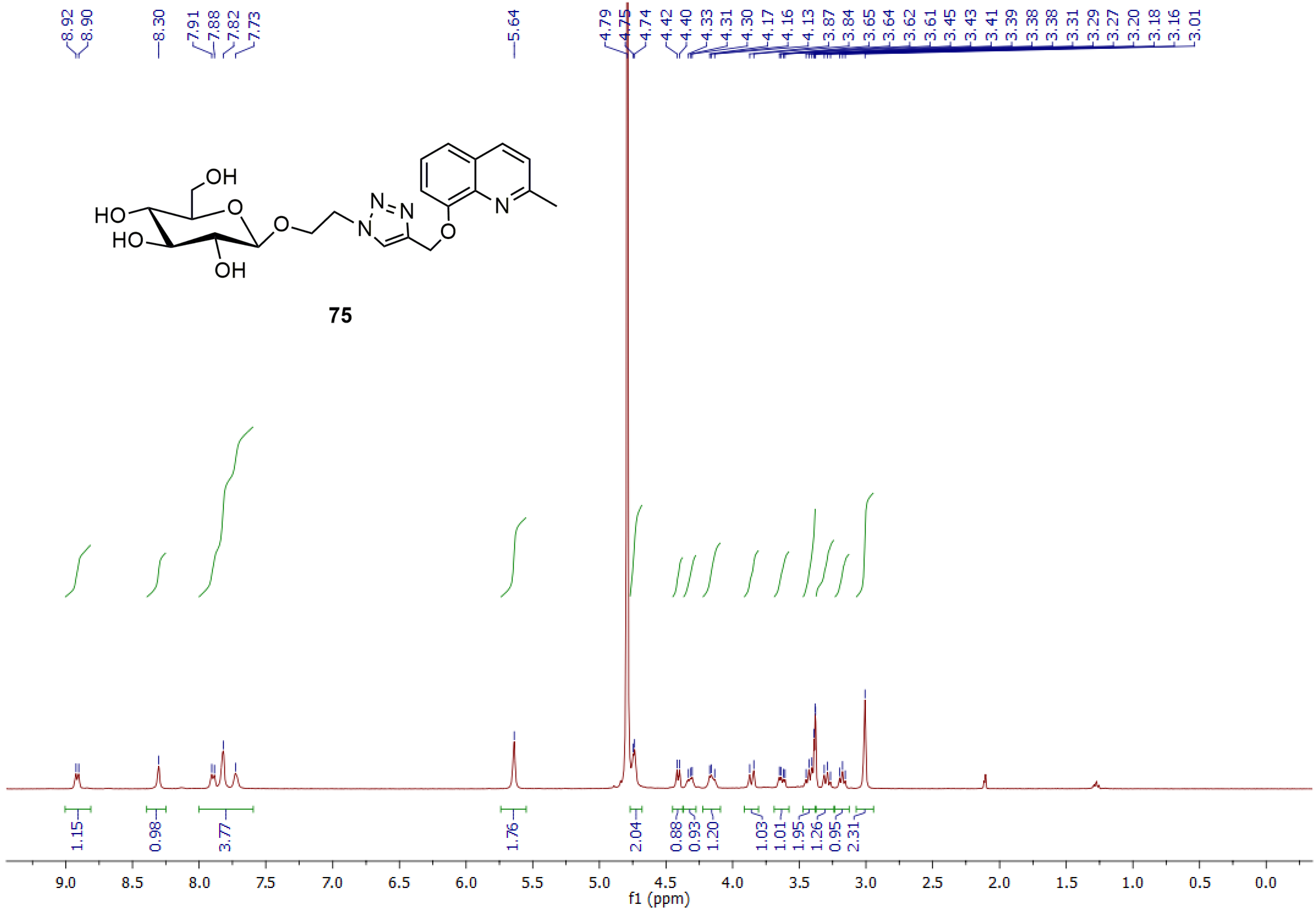

Fig. S141: ^{1}H NMR spectrum of glycoconjugate **75**.

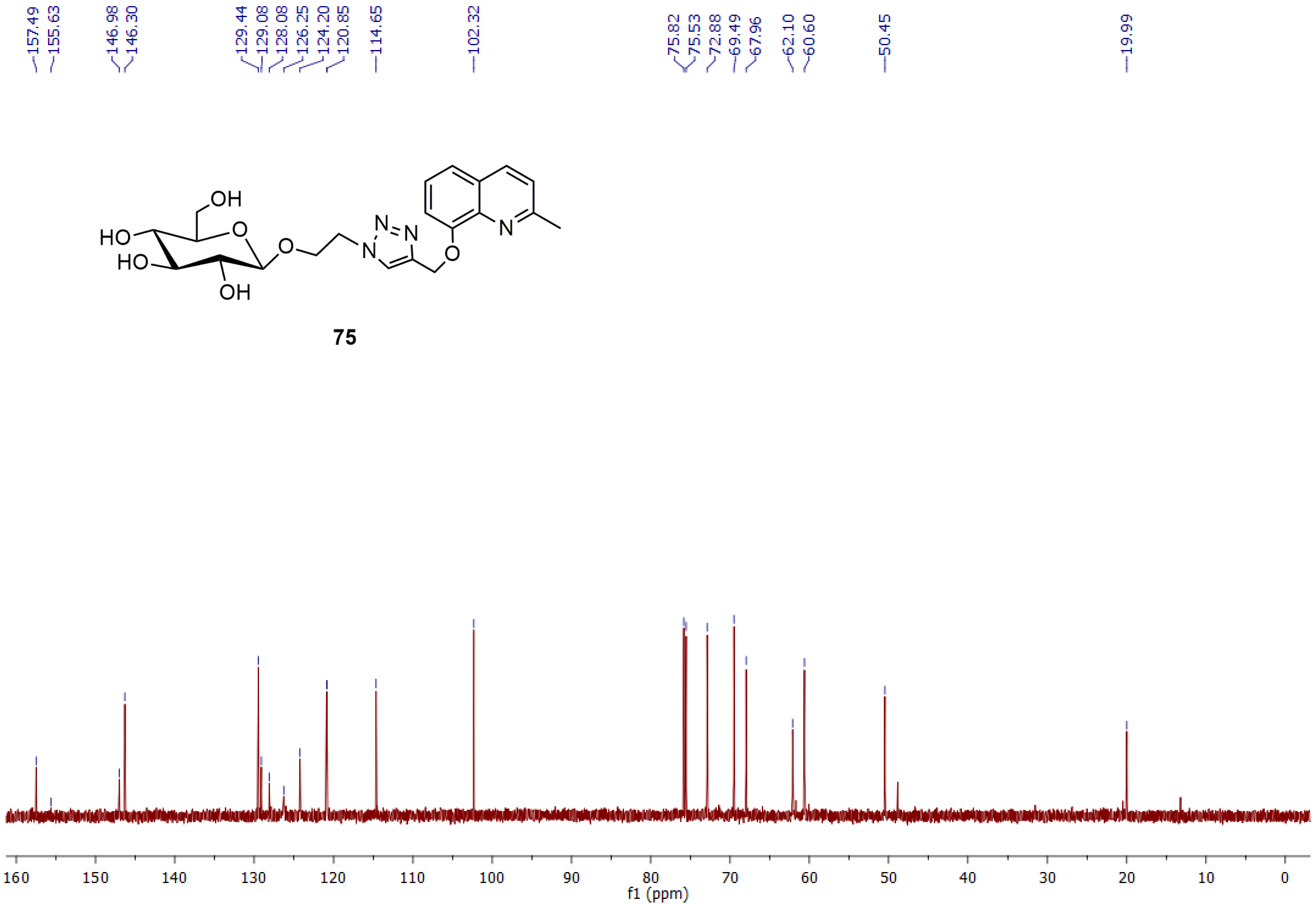

Fig. S142: ^{13}C NMR spectrum of glycoconjugate **75**.

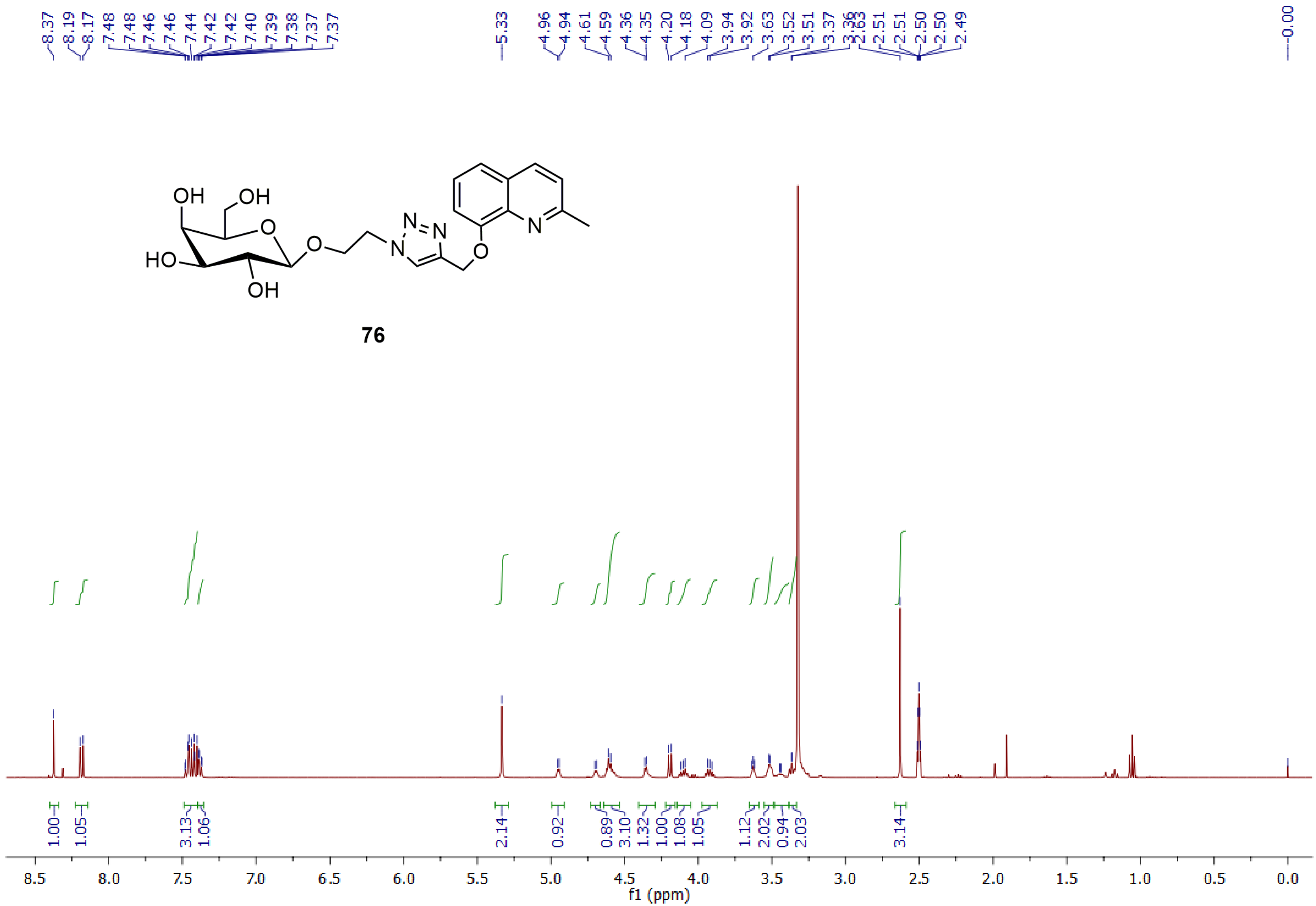

Fig. S143: ^{1}H NMR spectrum of glycoconjugate **76**.

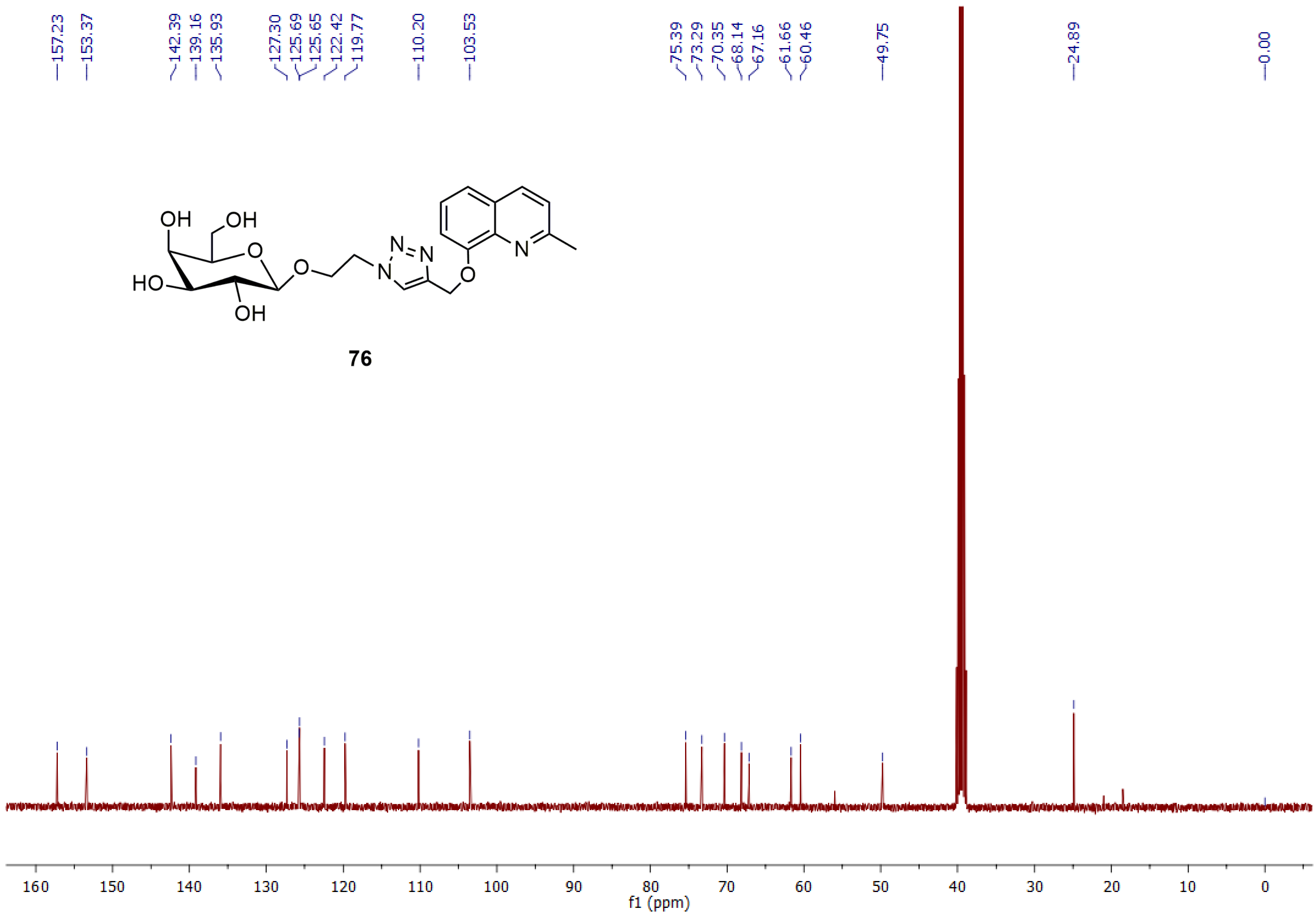

Fig. S144: ^{13}C NMR spectrum of glycoconjugate **76**.

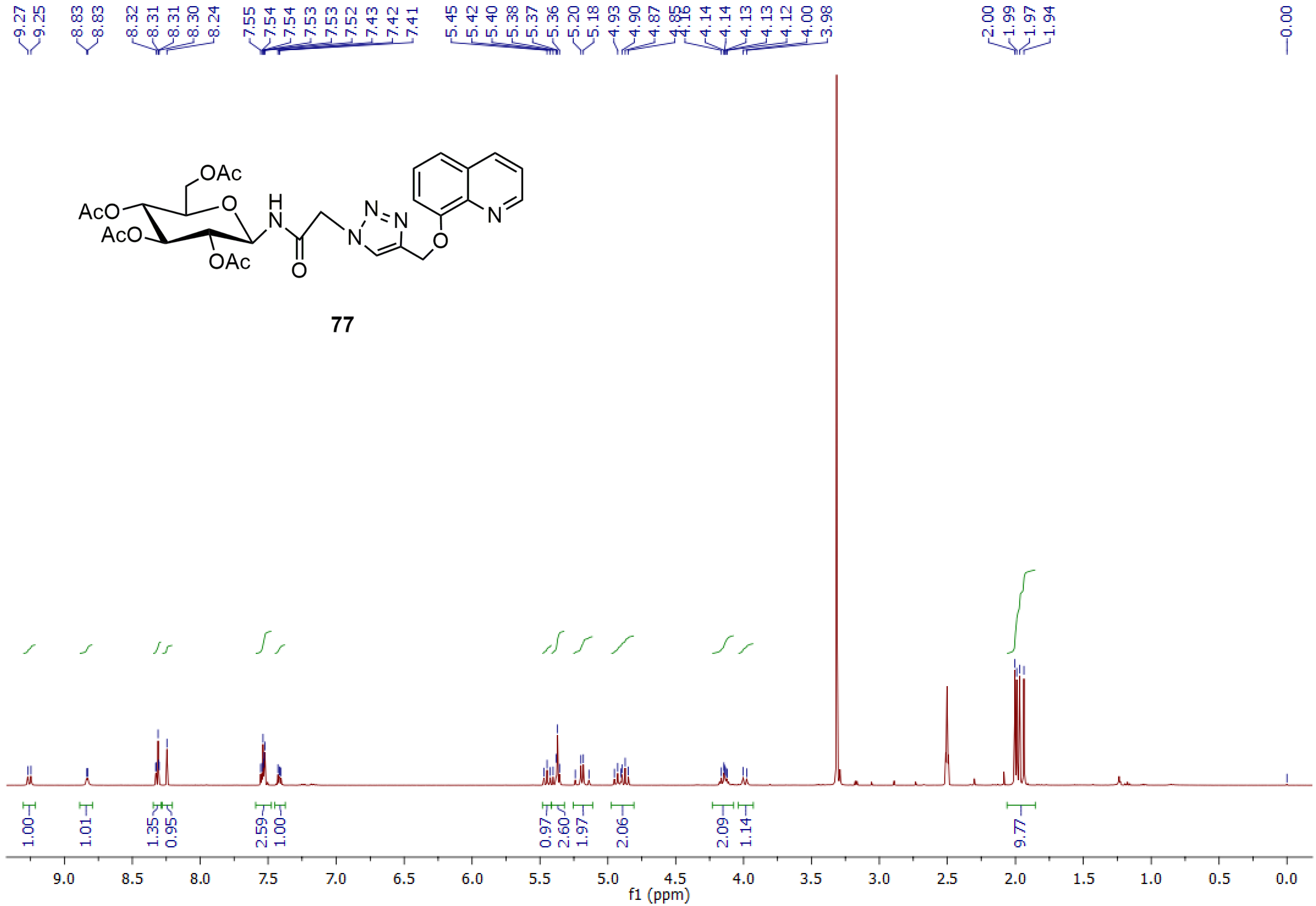

77

Fig. S145: ^{1}H NMR spectrum of glycoconjugate **77**.

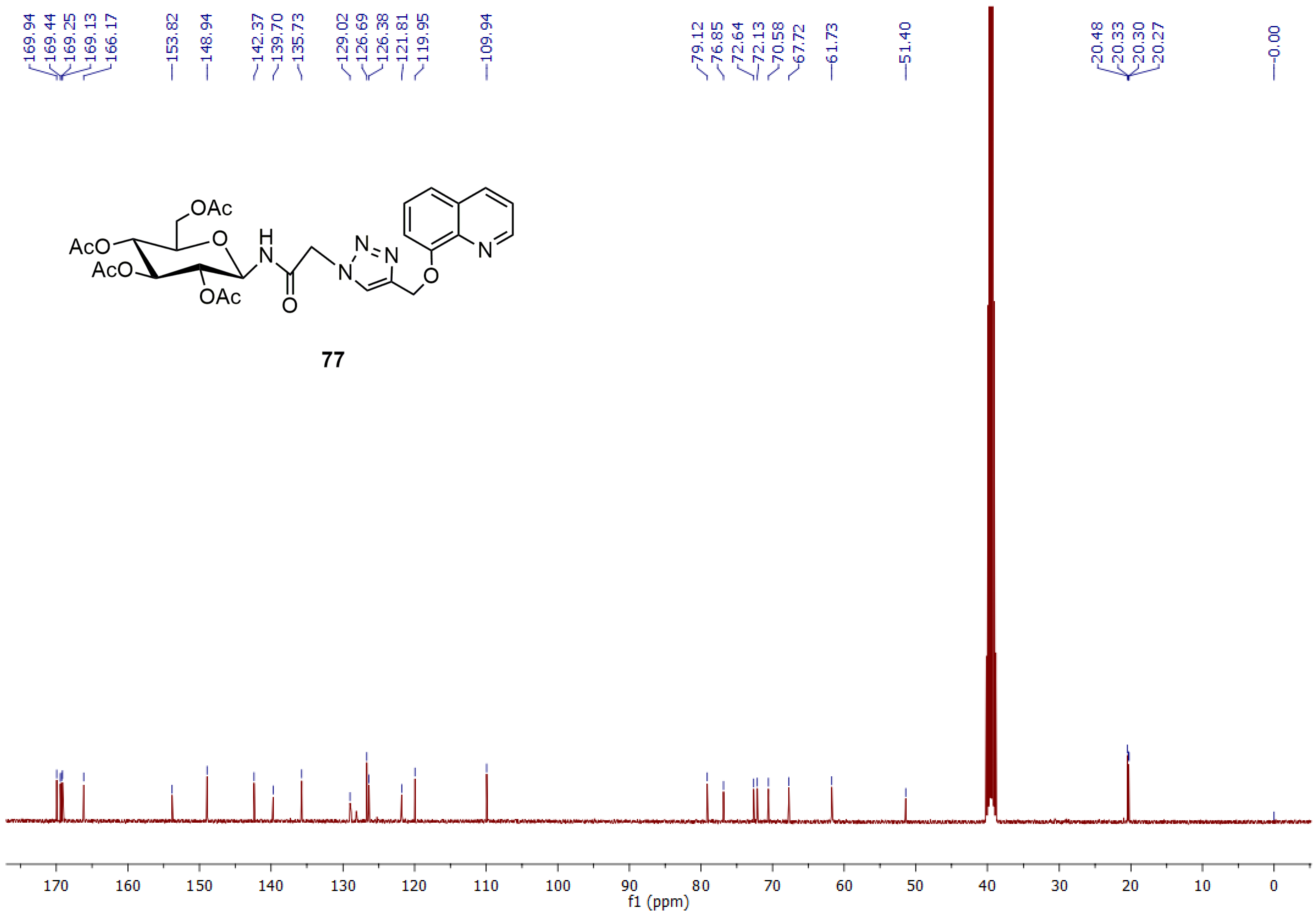

Fig. S146: ^{13}C NMR spectrum of glycoconjugate **77**.

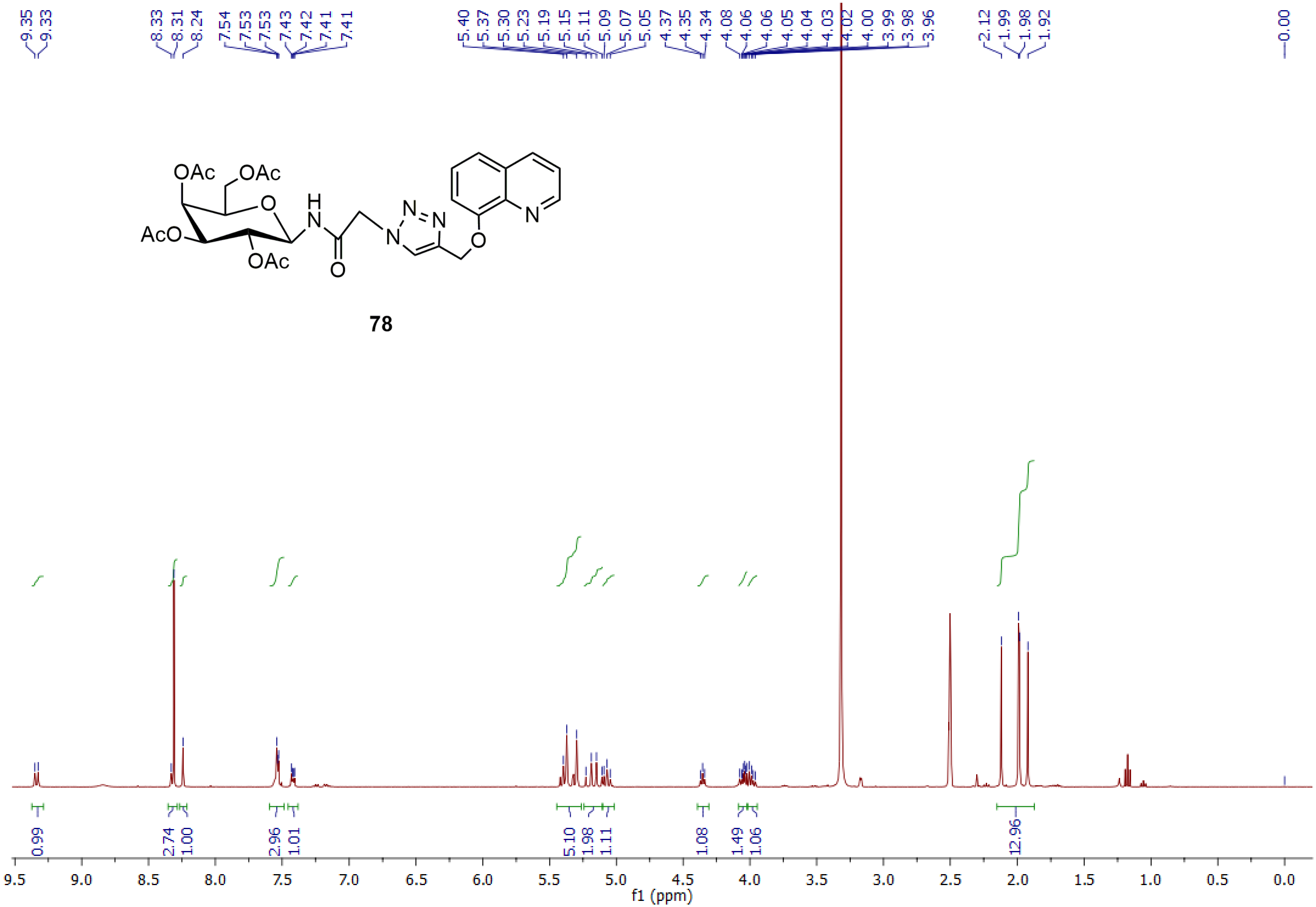

Fig. S147: ^{1}H NMR spectrum of glycoconjugate **78**.

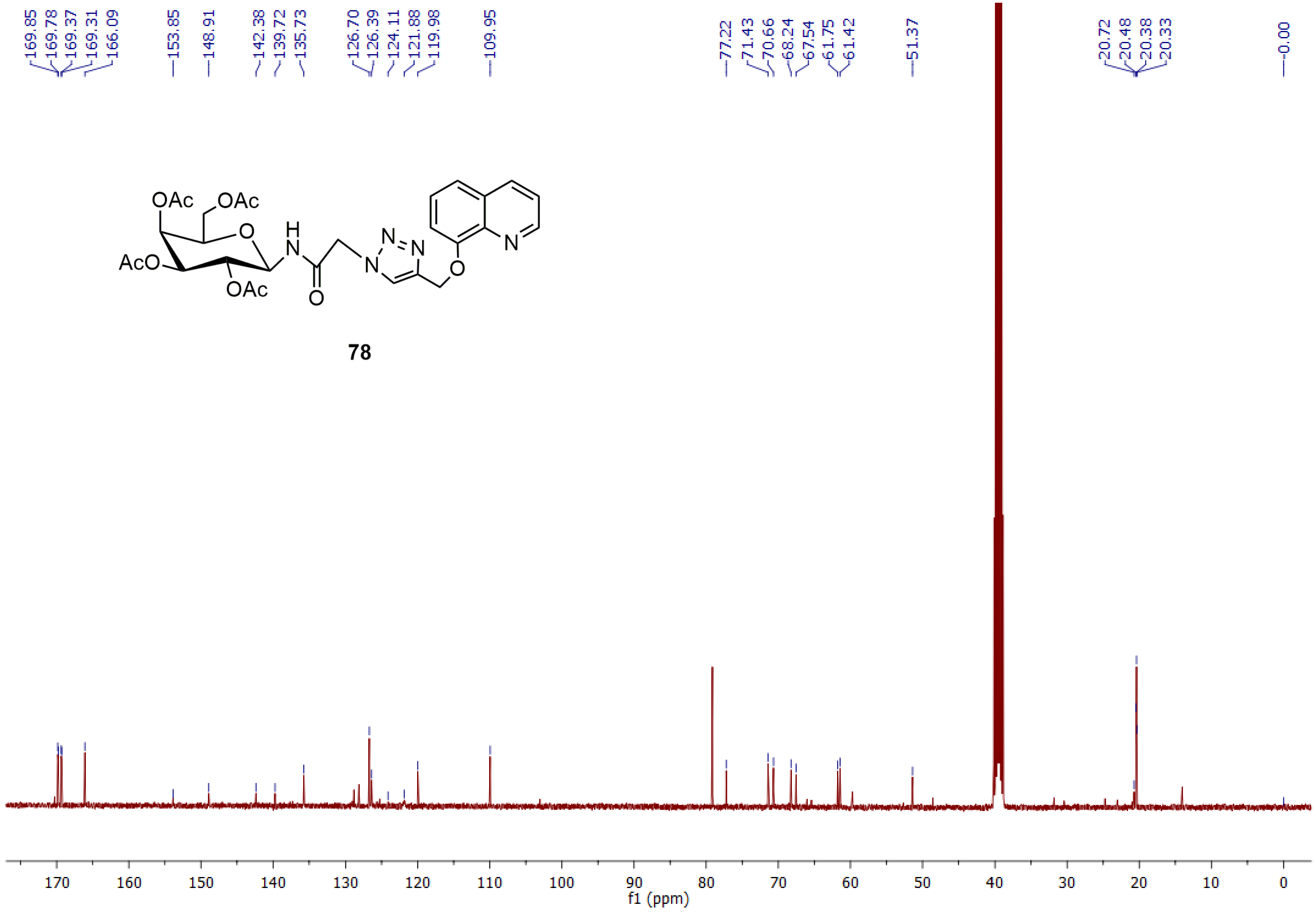

Fig. S148: ^{13}C NMR spectrum of glycoconjugate **78**.

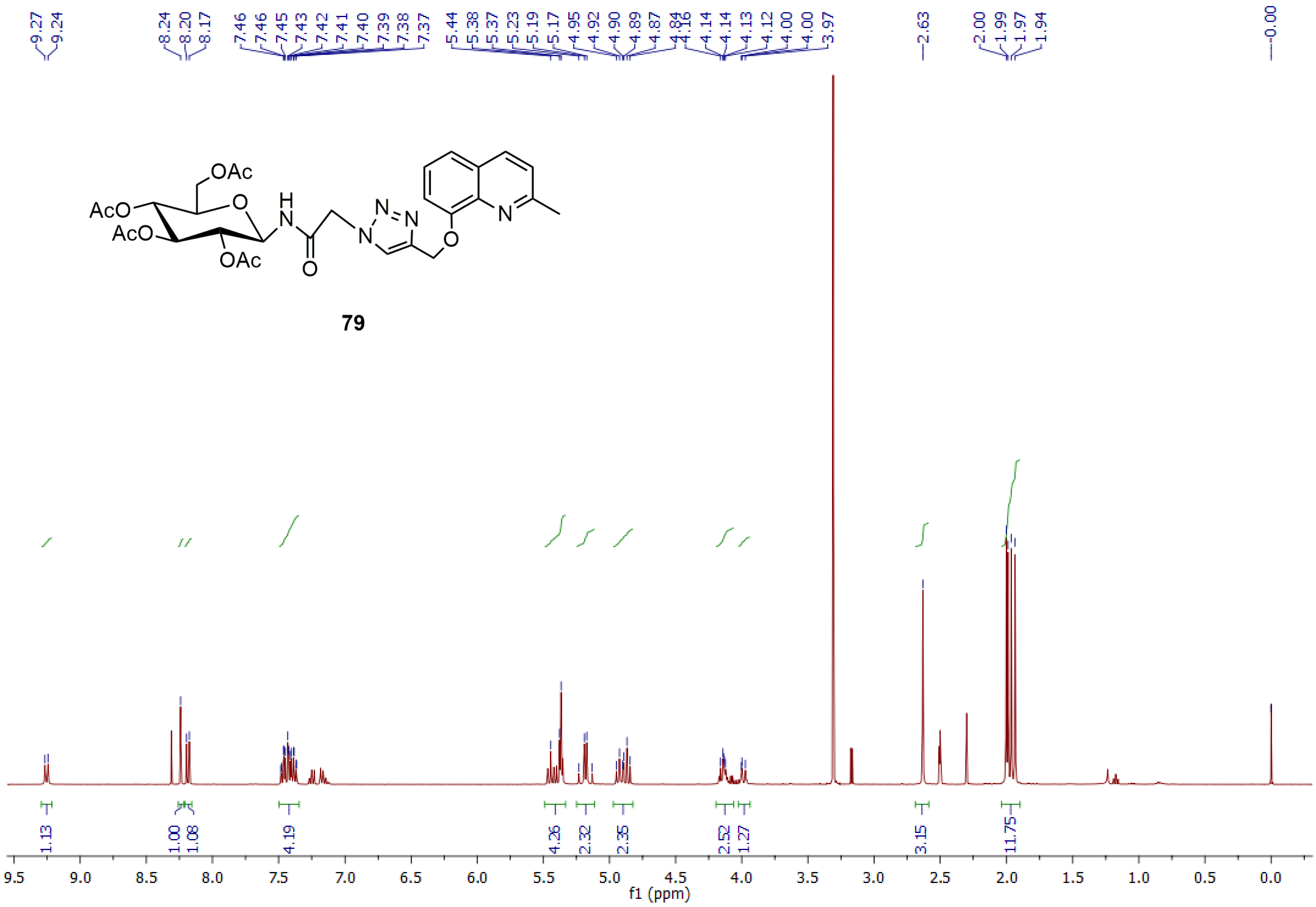

Fig. S149: ^{1}H NMR spectrum of glycoconjugate **79**.

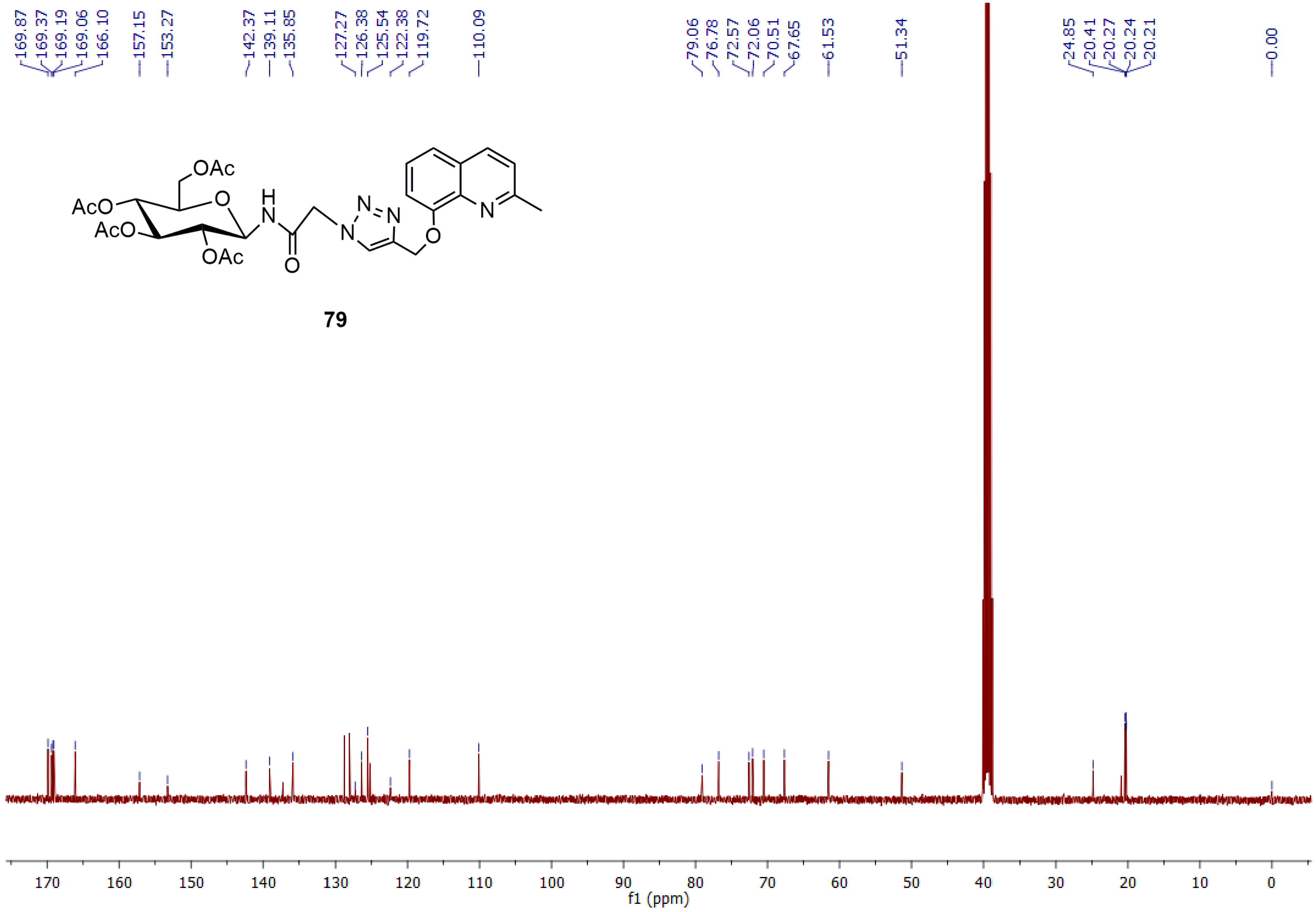

Fig. S150: ^{13}C NMR spectrum of glycoconjugate **79**.

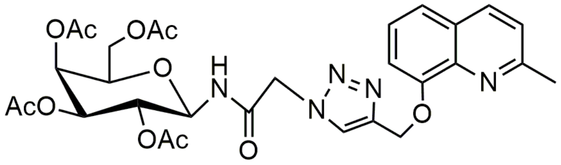

80

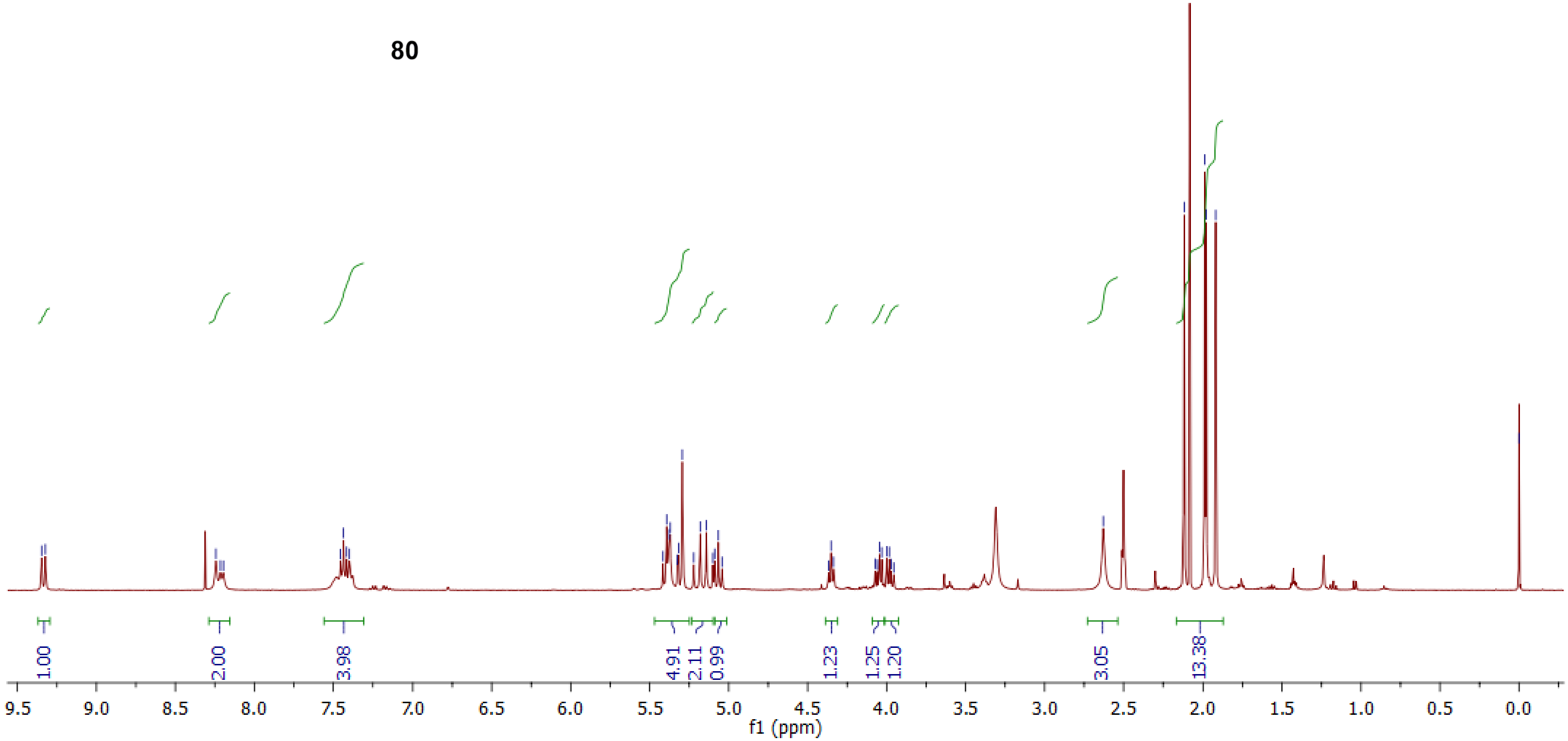

Fig. S151: ^{1}H NMR spectrum of glycoconjugate **80**.

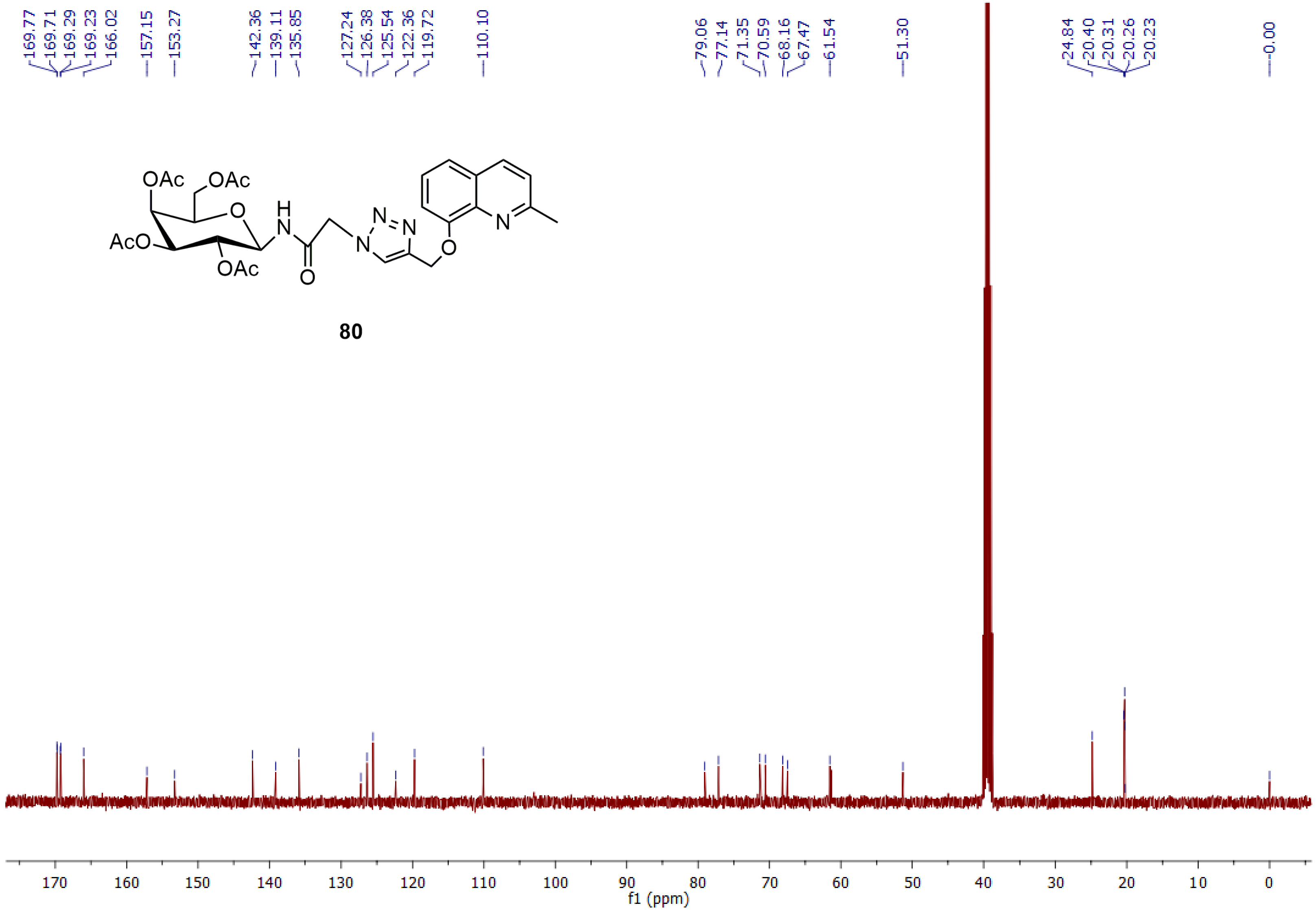

Fig. S152: ^{13}C NMR spectrum of glycoconjugate **80**.

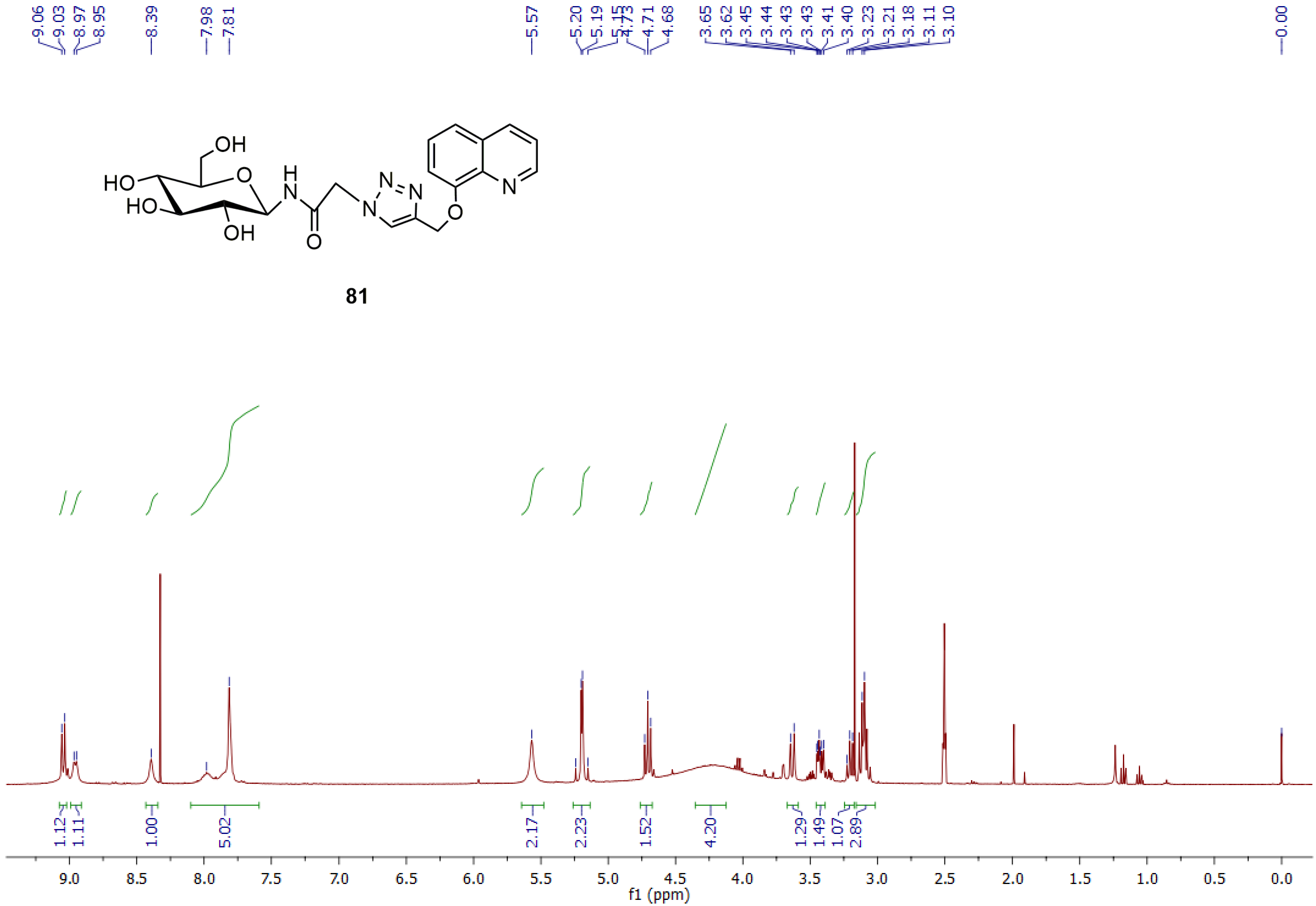

Fig. S153: ^{1}H NMR spectrum of glycoconjugate **81**.

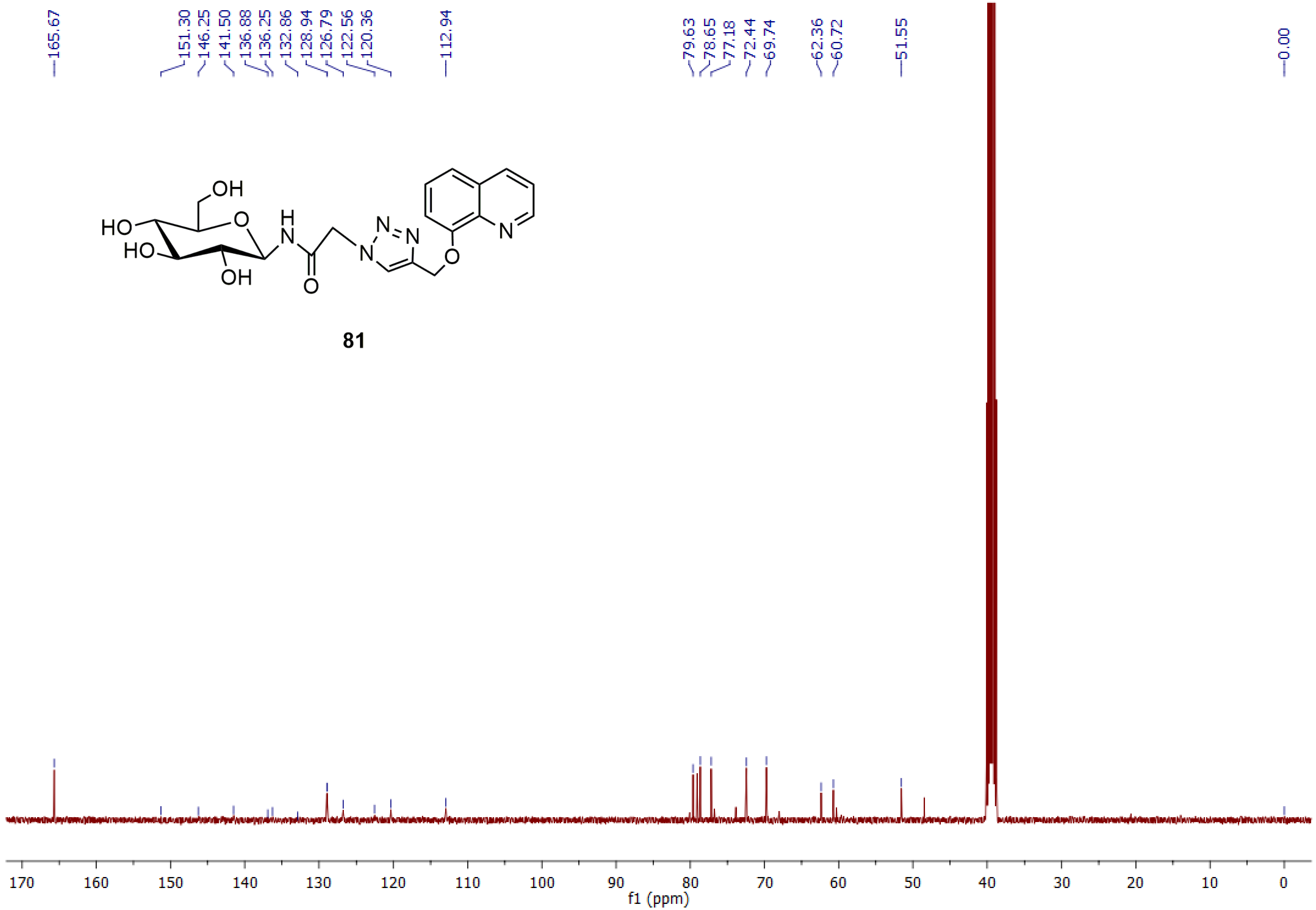

Fig. S154: ^{13}C NMR spectrum of glycoconjugate **81**.

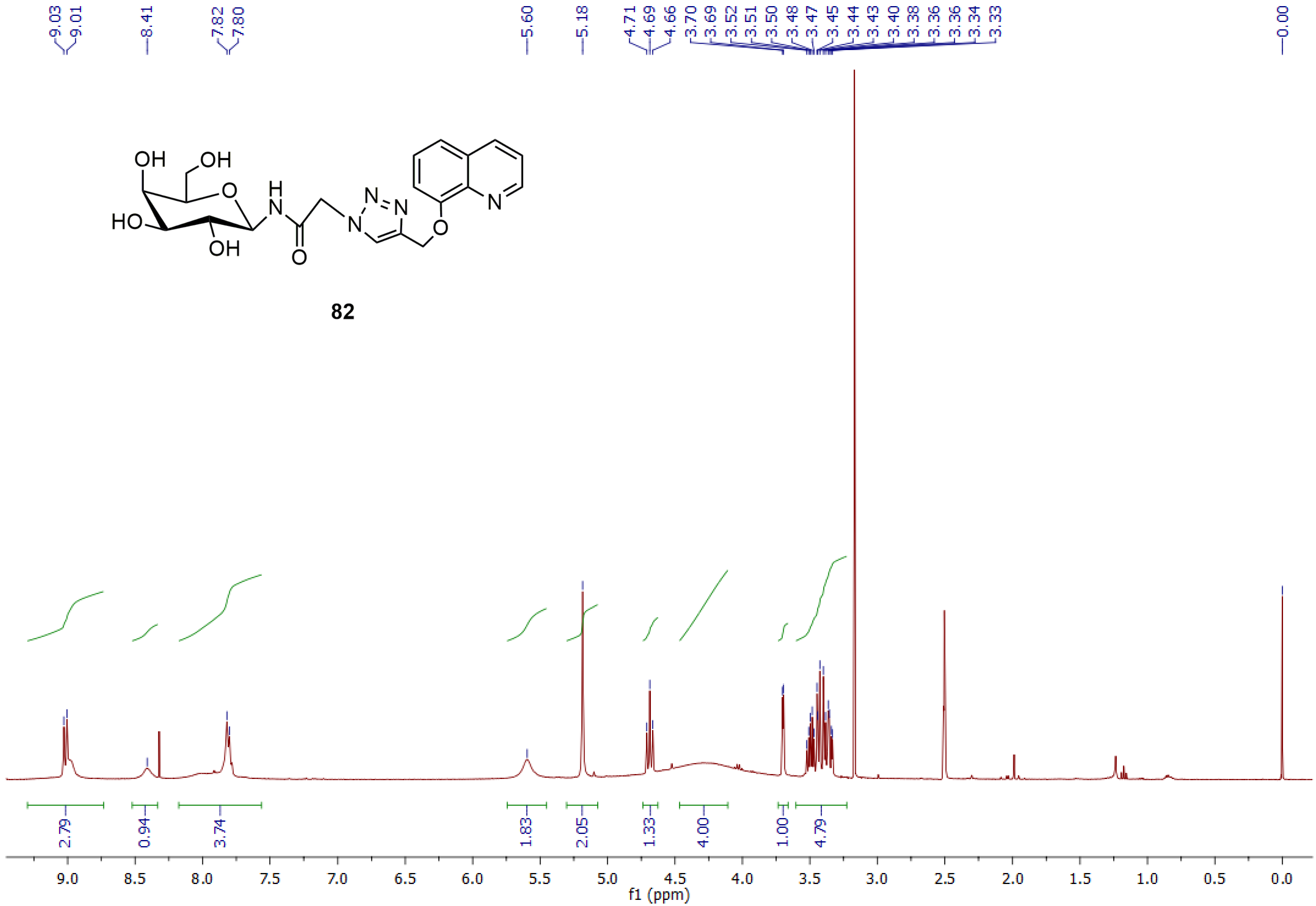

Fig. S155: ^{1}H NMR spectrum of glycoconjugate **82**.

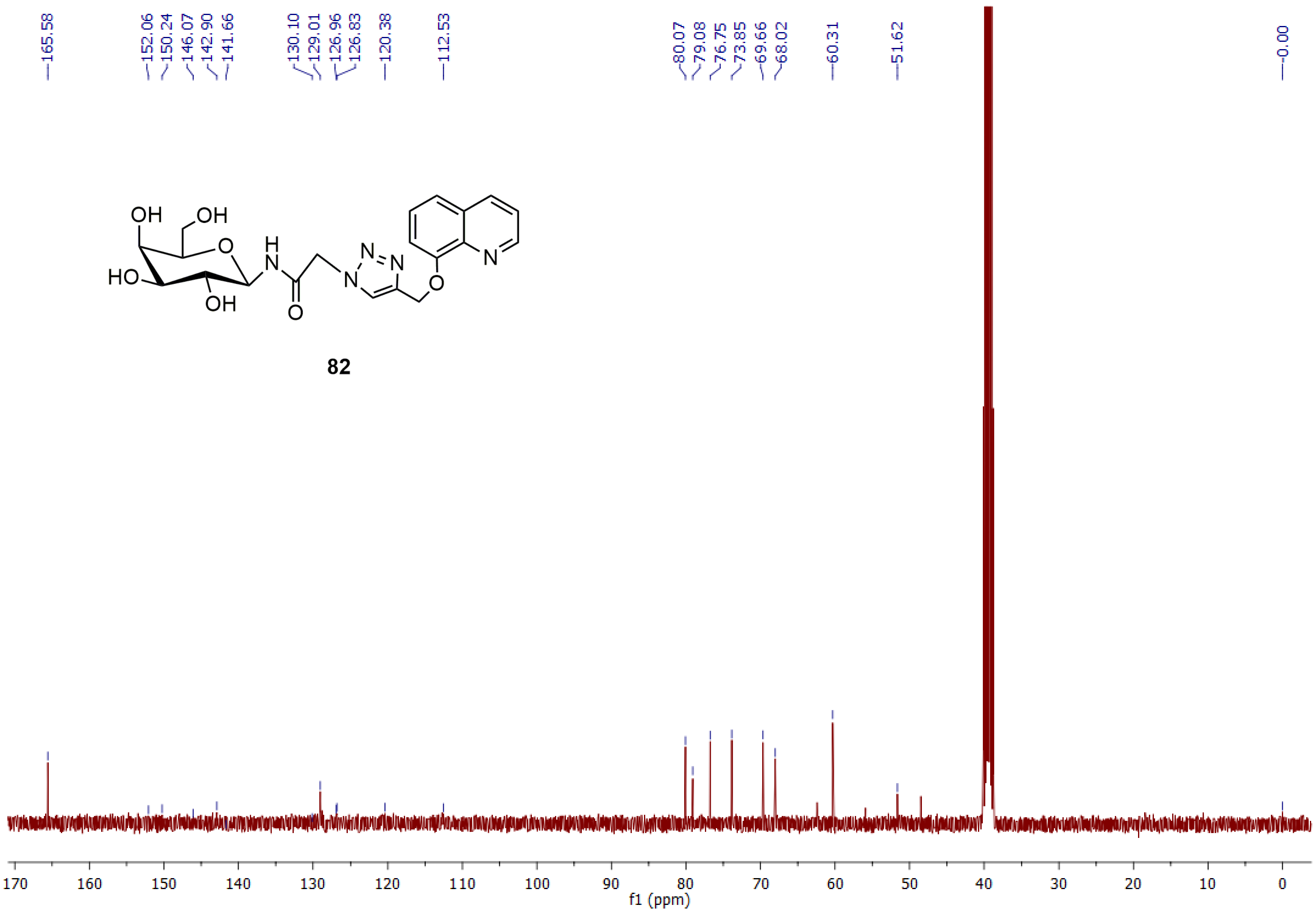

82

Fig. S156: ^{13}C NMR spectrum of glycoconjugate **82**.

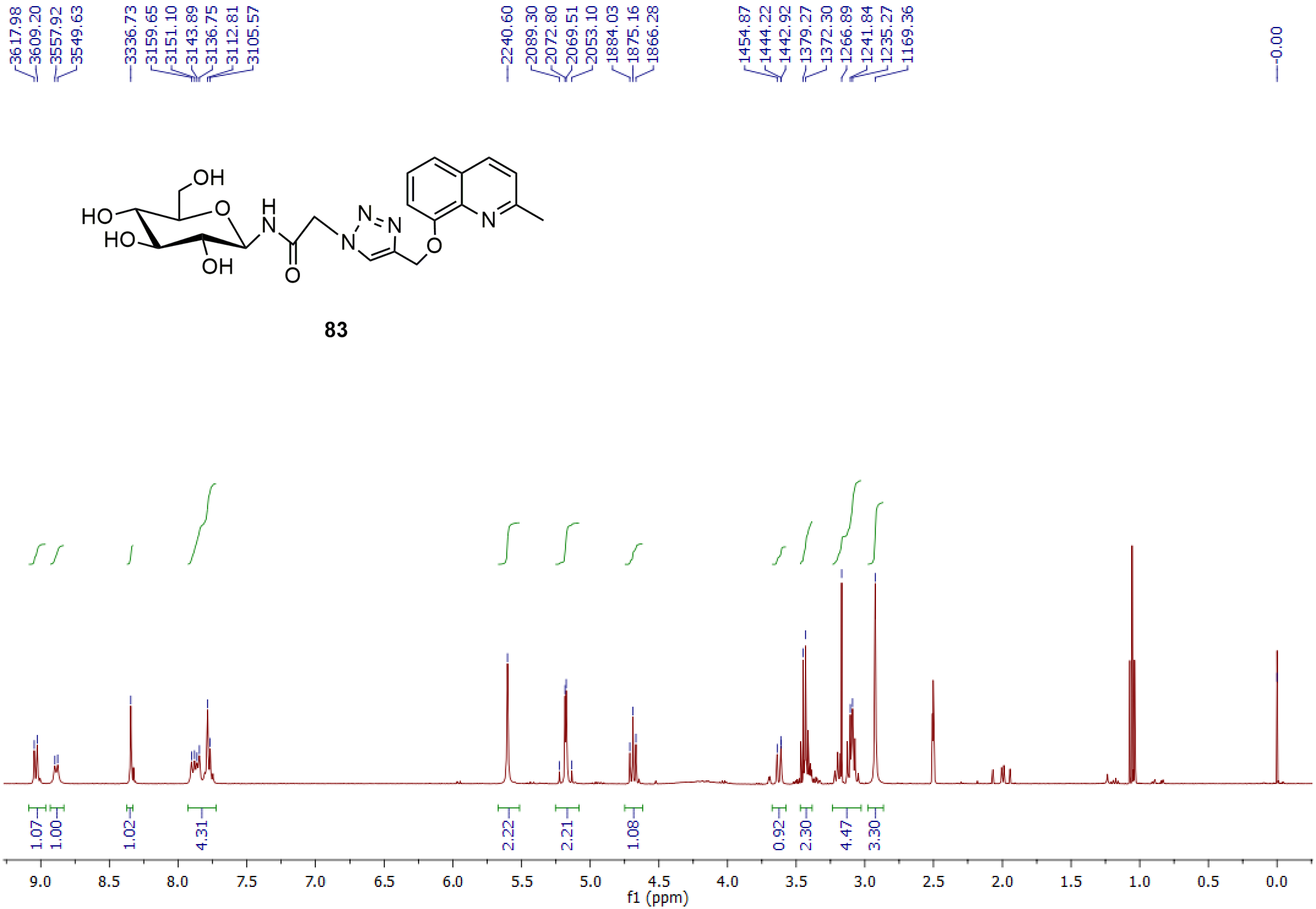

Fig. S157: ^{1}H NMR spectrum of glycoconjugate **83**.

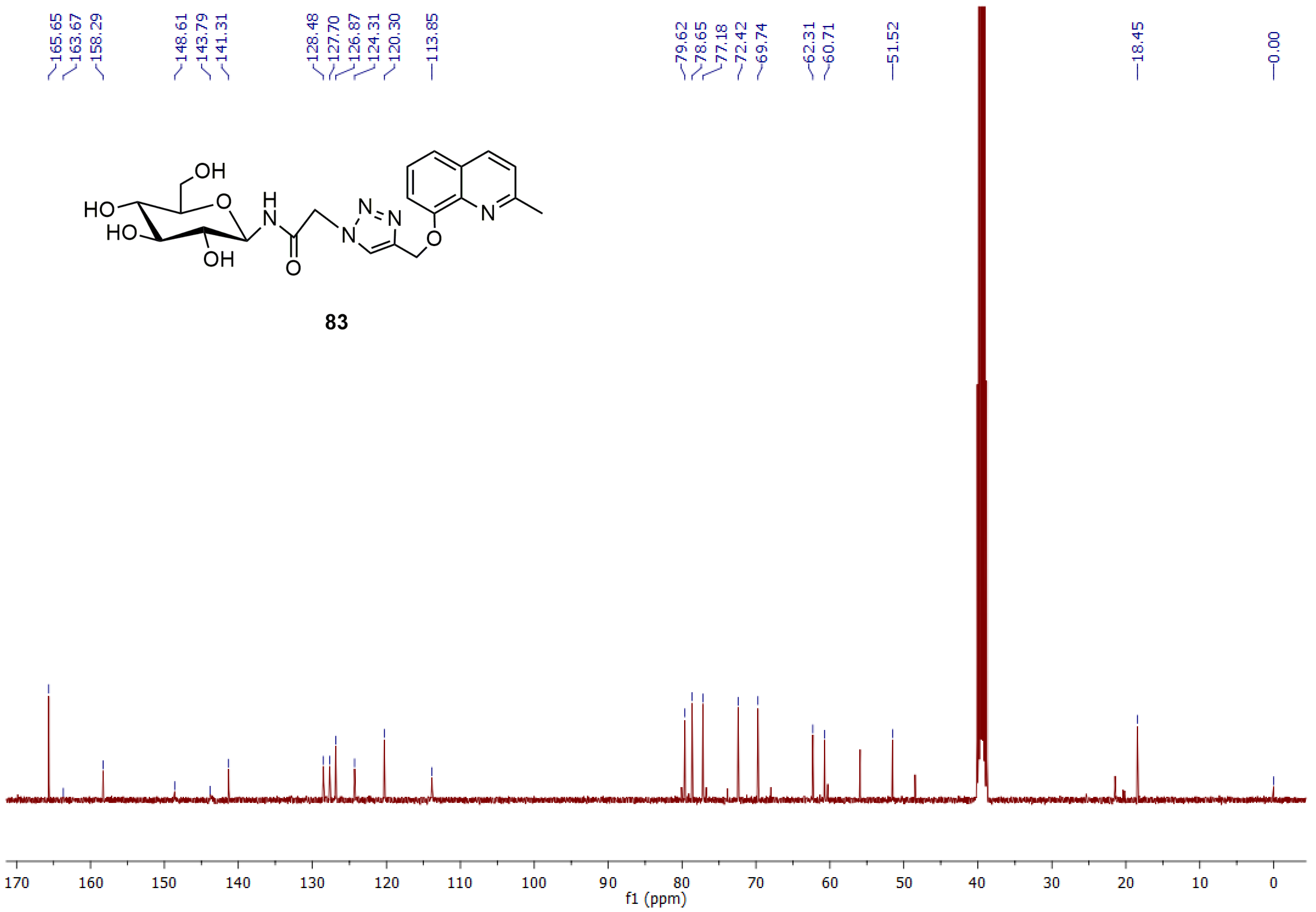

Fig. S158: ^{13}C NMR spectrum of glycoconjugate **83**.

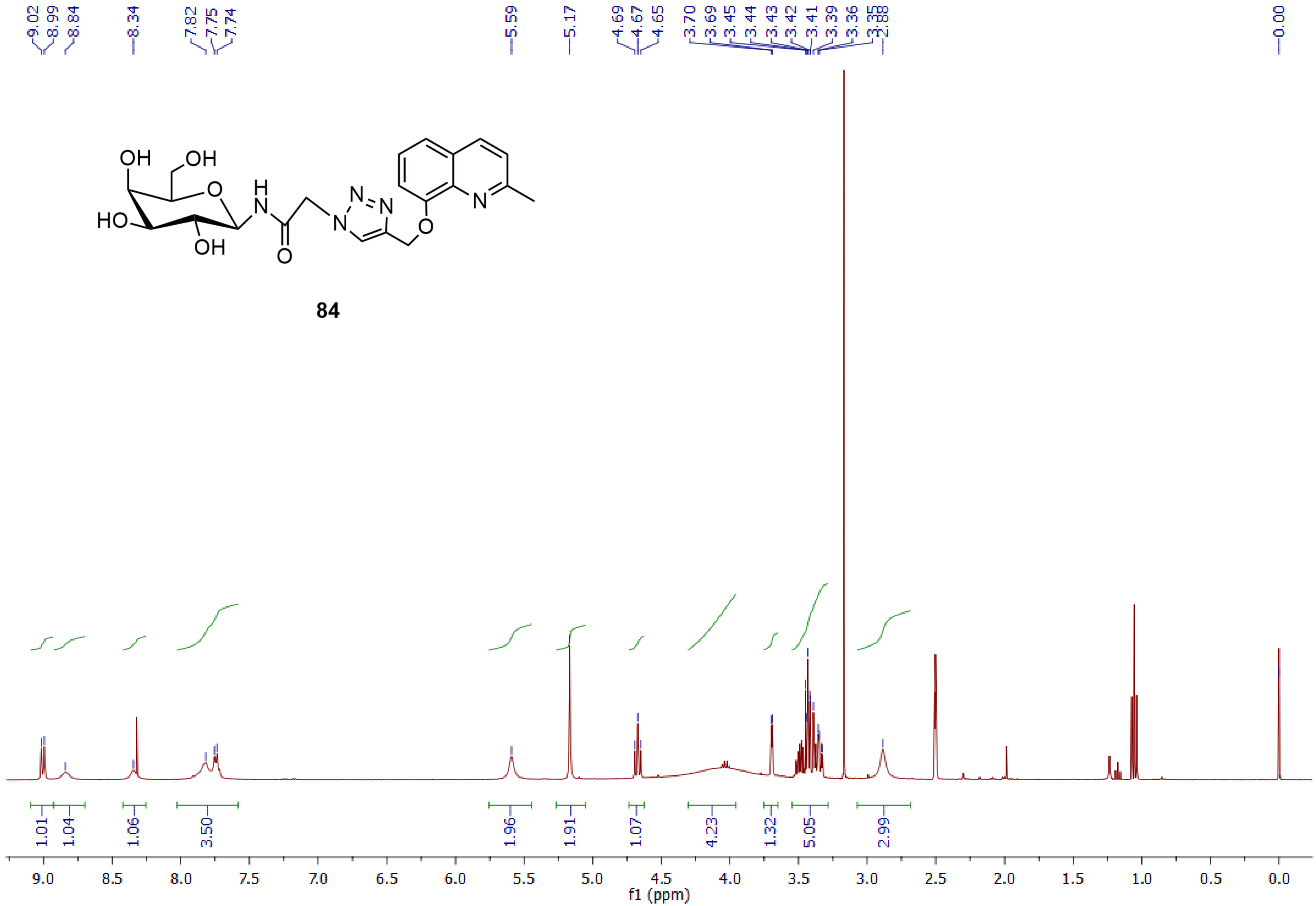

Fig. S159: ^{1}H NMR spectrum of glycoconjugate **84**.

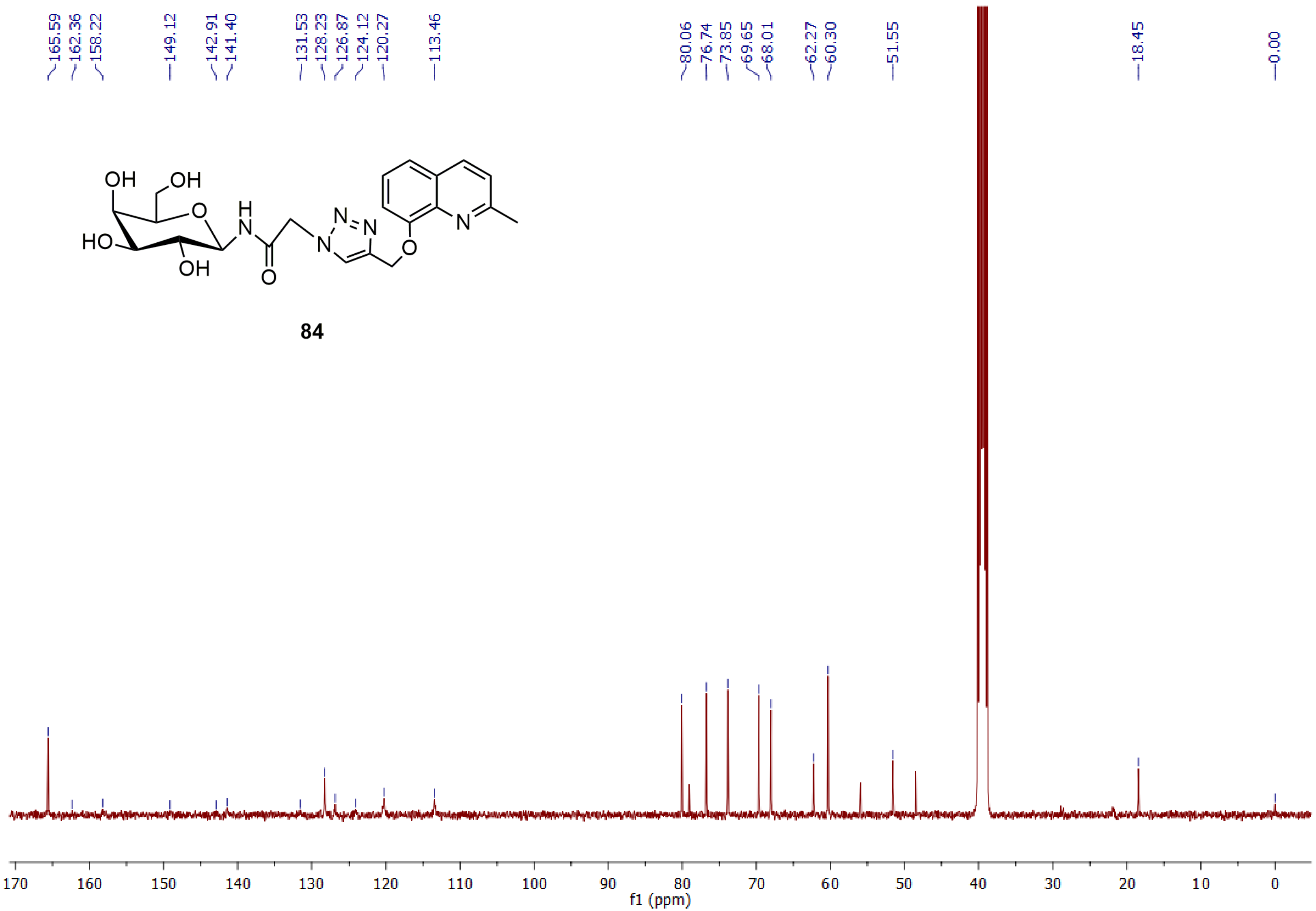

Fig. S160: ^{13}C NMR spectrum of glycoconjugate **84**.

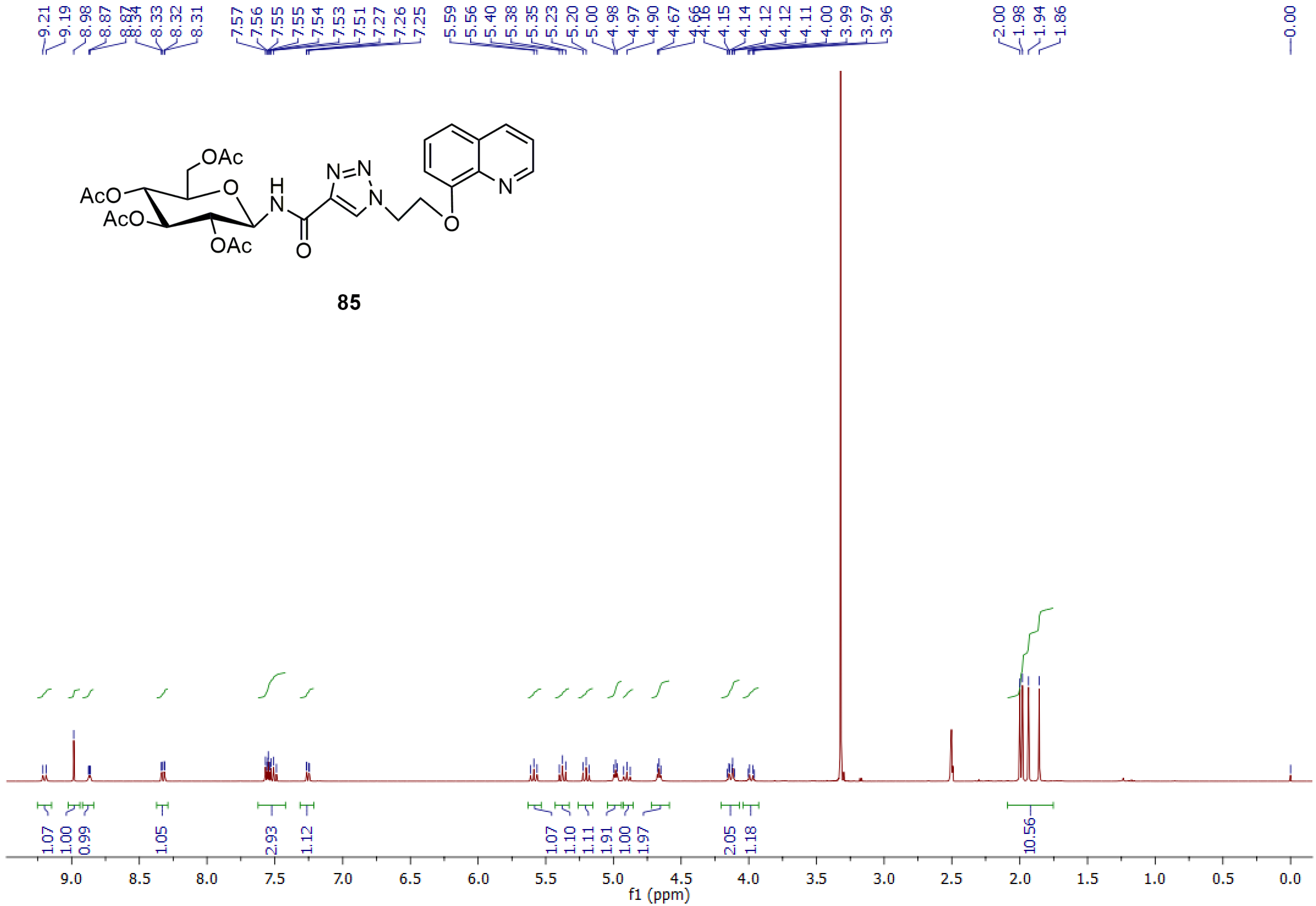

Fig. S161: ^{1}H NMR spectrum of glycoconjugate **85**.

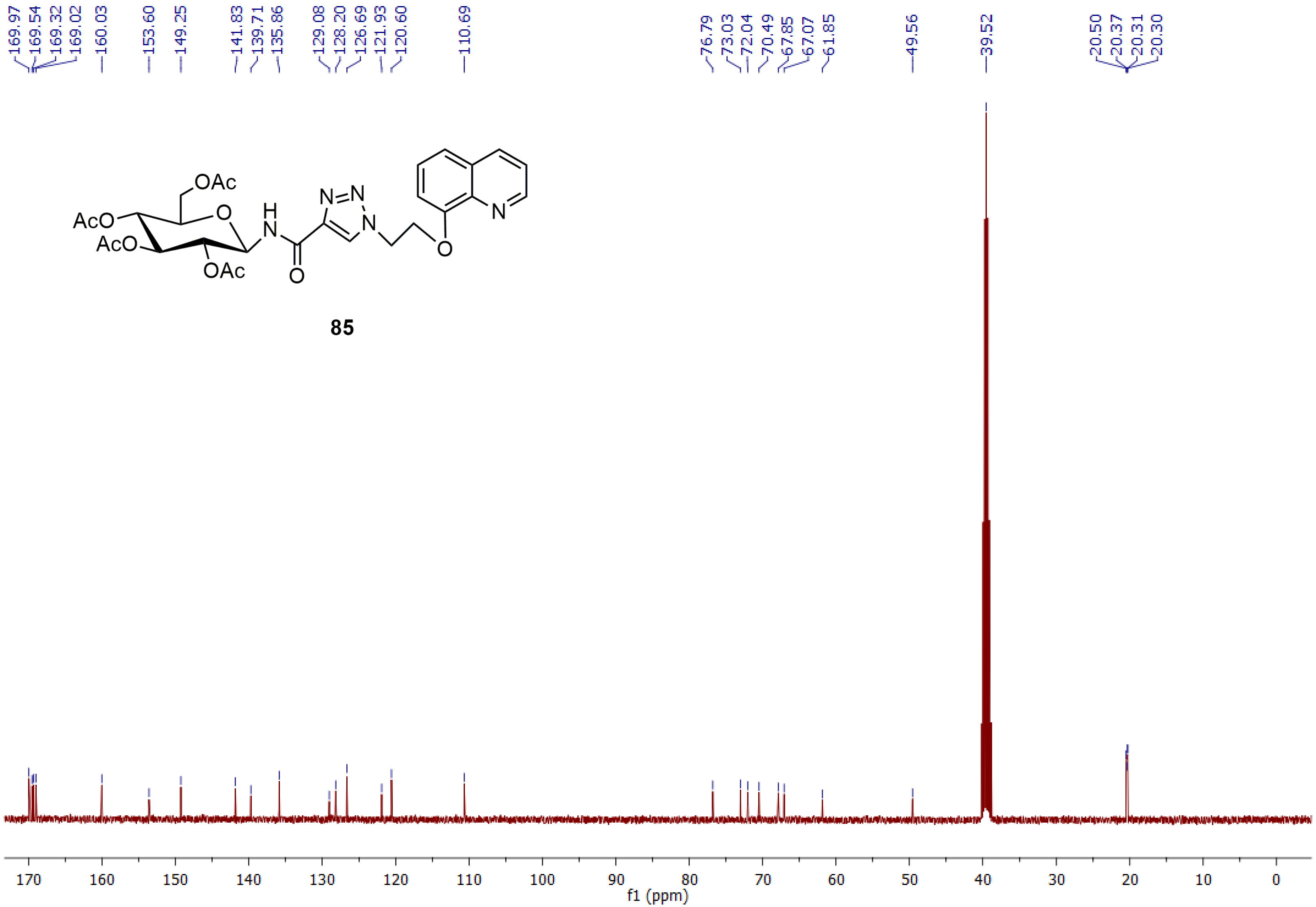

Fig. S162: ^{13}C NMR spectrum of glycoconjugate **85**.

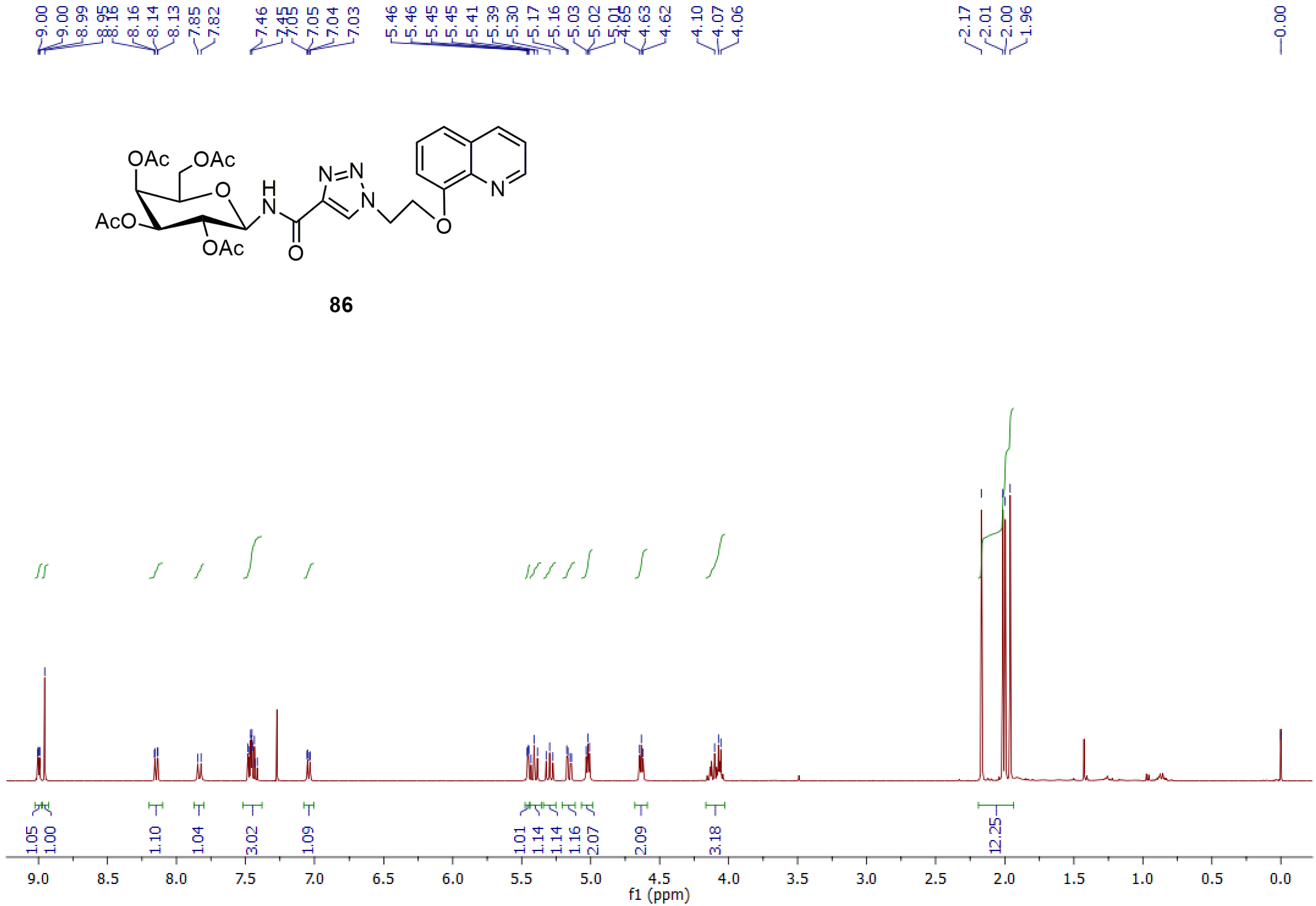

86

Fig. S163: ^{1}H NMR spectrum of glycoconjugate **86**.

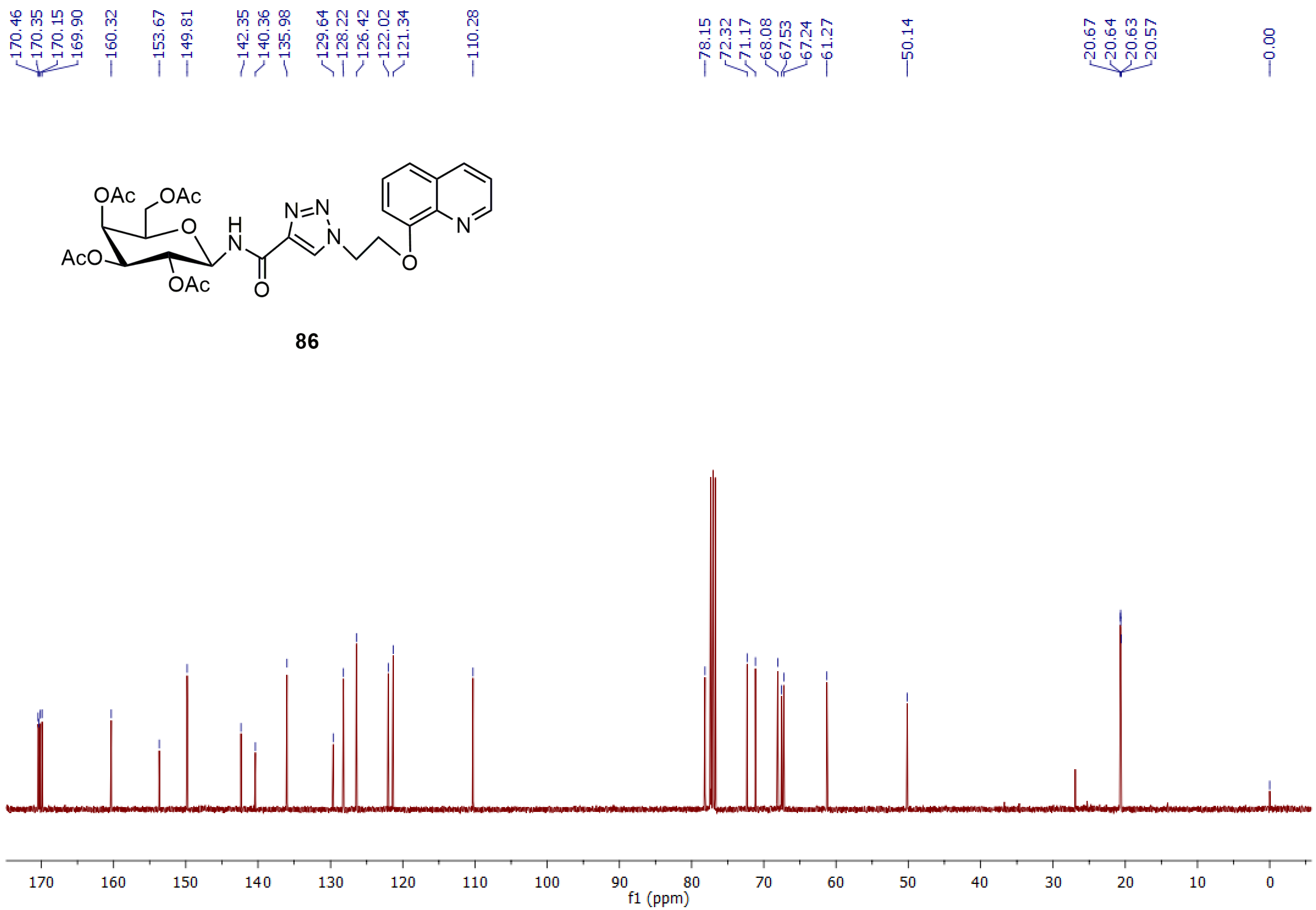

Fig. S164: ^{13}C NMR spectrum of glycoconjugate **86**.

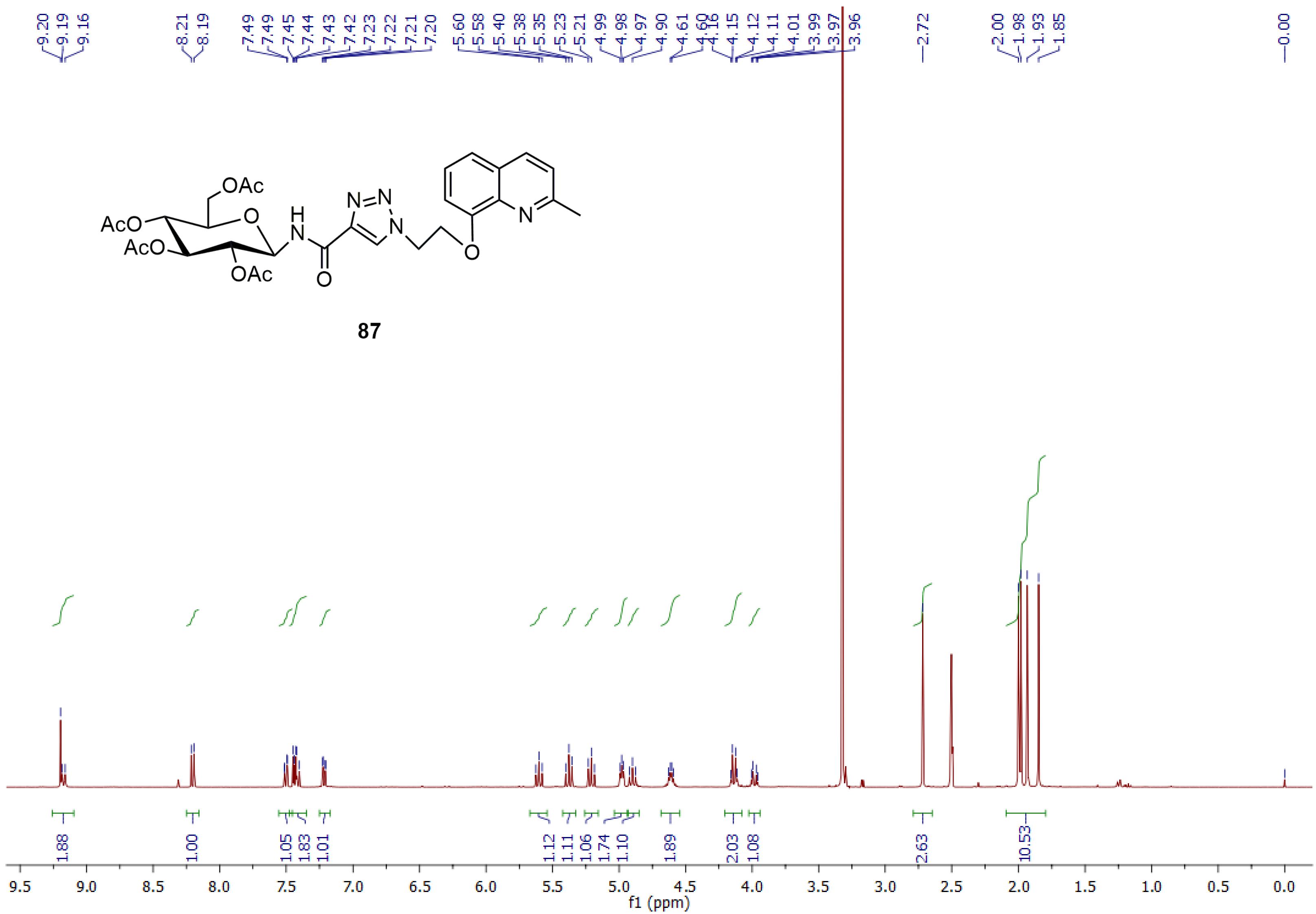

Fig. S165: ^{1}H NMR spectrum of glycoconjugate **87**.

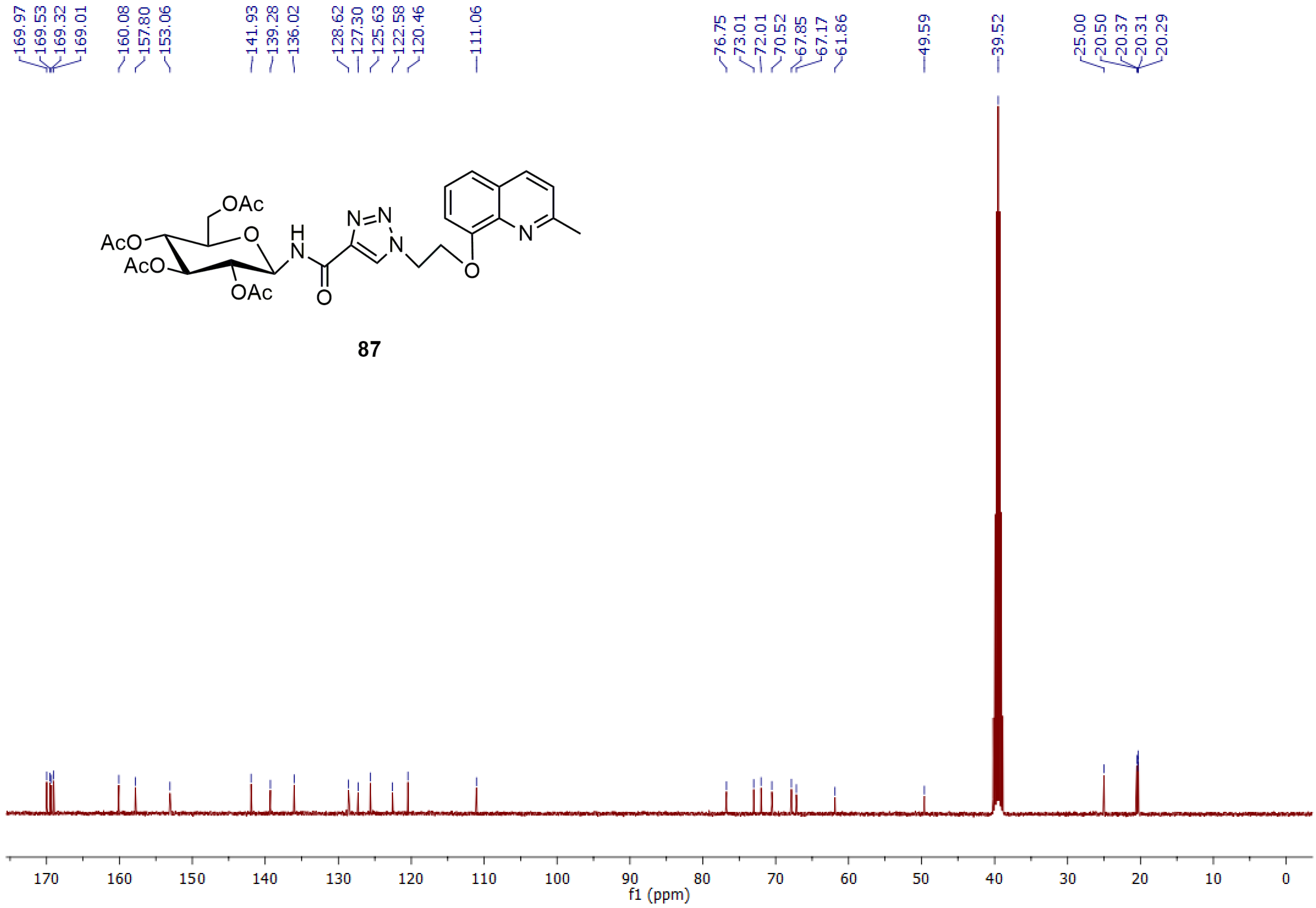

Fig. S166: ^{13}C NMR spectrum of glycoconjugate **87**.

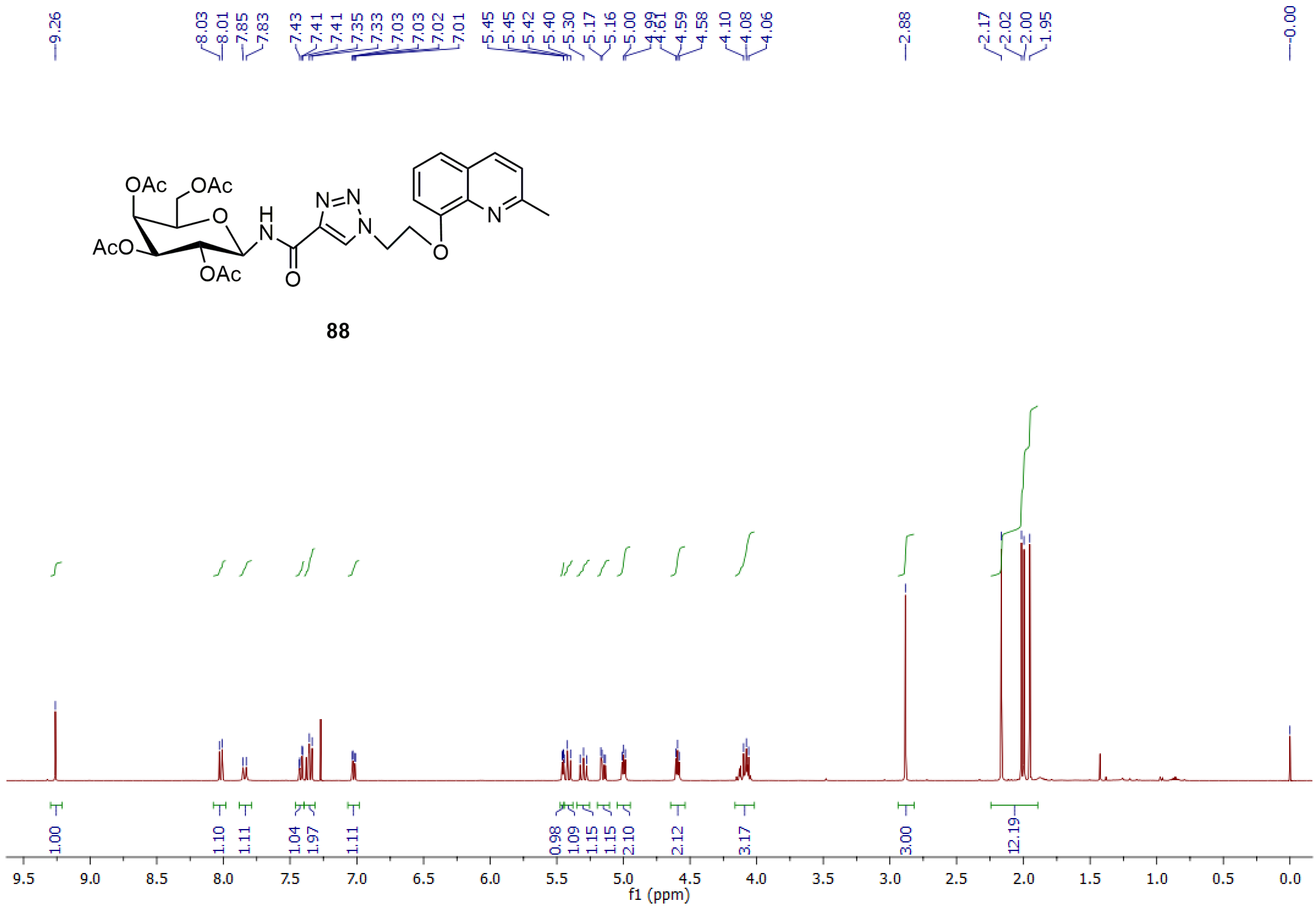

Fig. S167: ^{1}H NMR spectrum of glycoconjugate **88**.

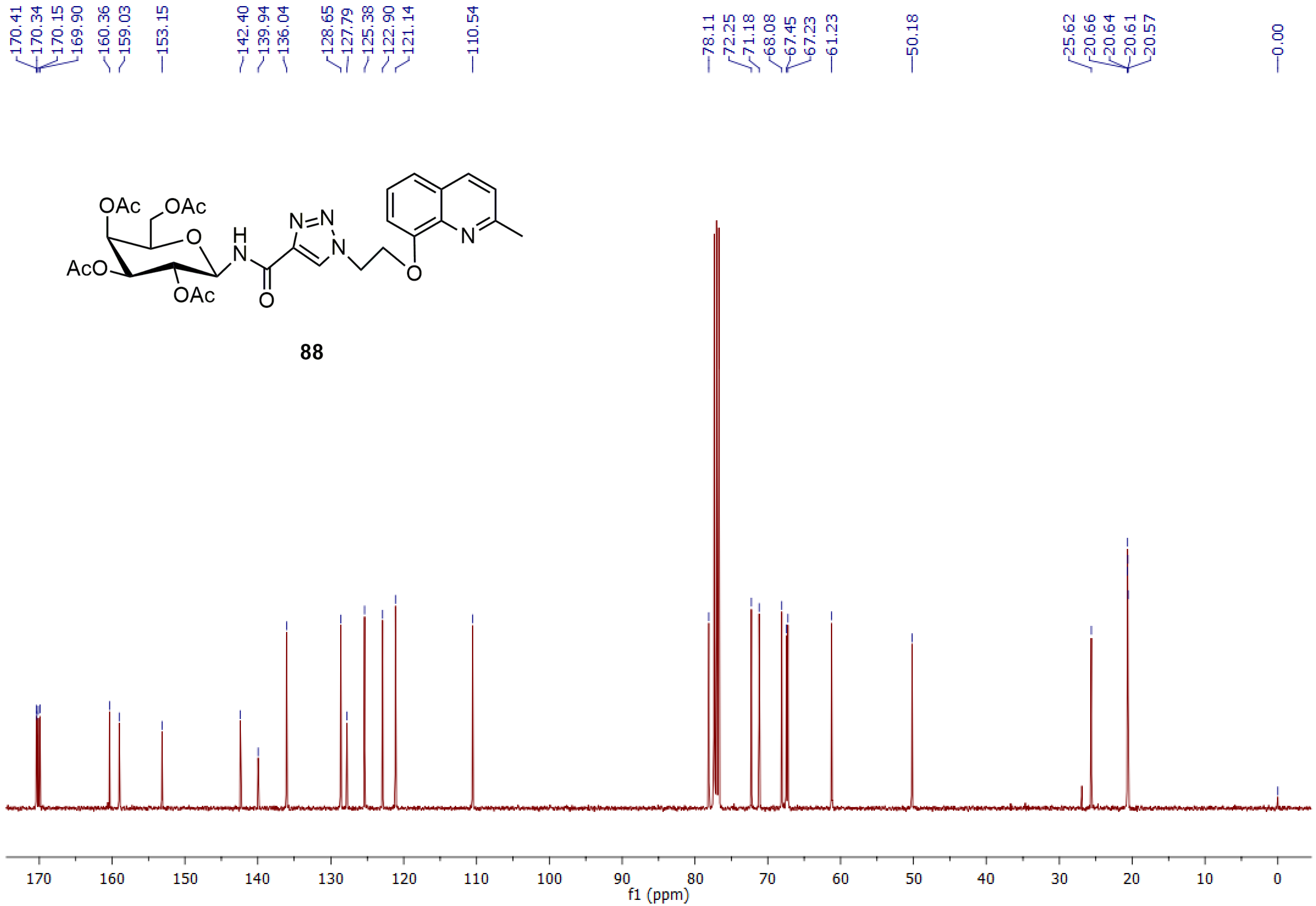

Fig. S168: ^{13}C NMR spectrum of glycoconjugate **88**.

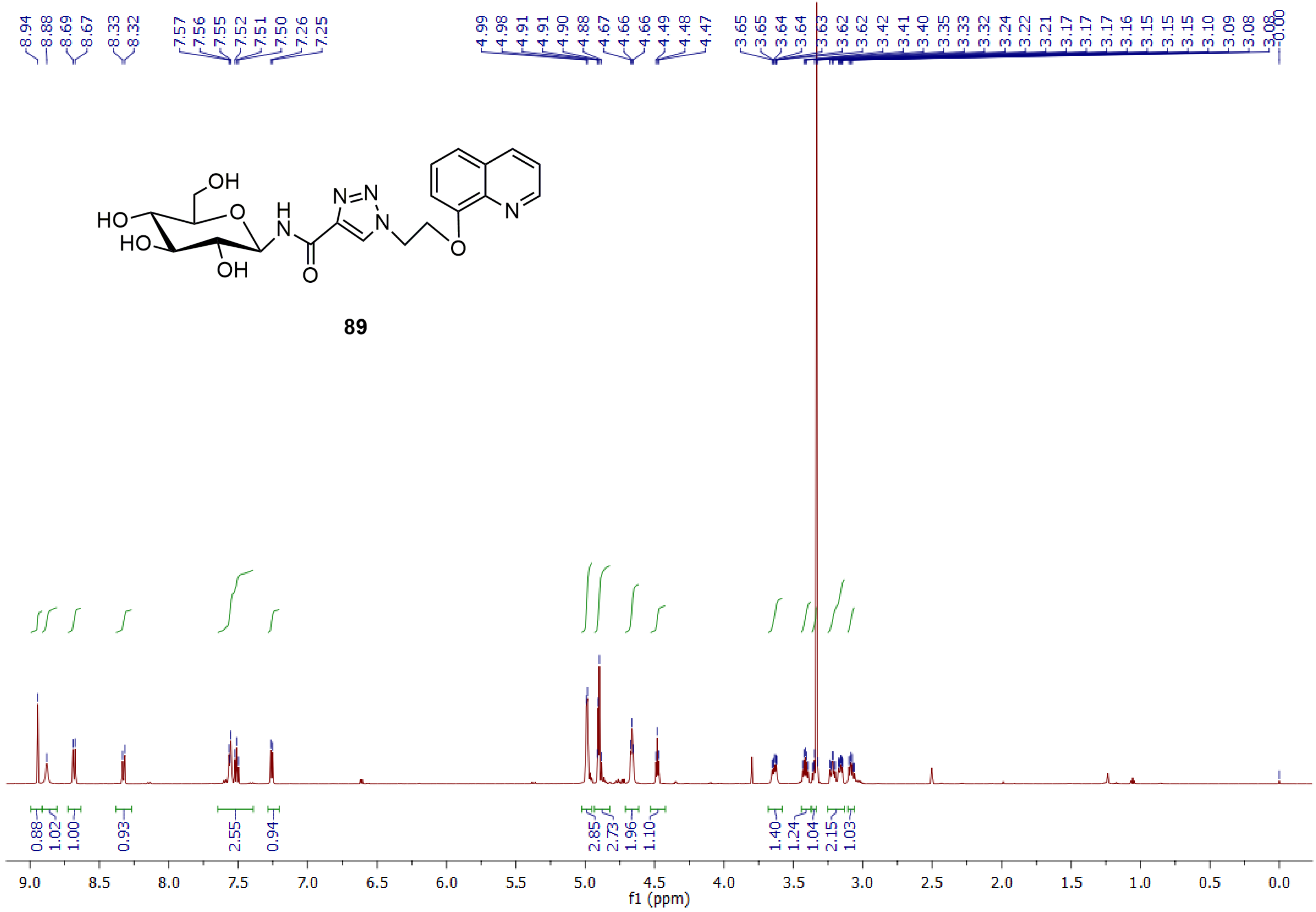

Fig. S169: ^{1}H NMR spectrum of glycoconjugate **89**.

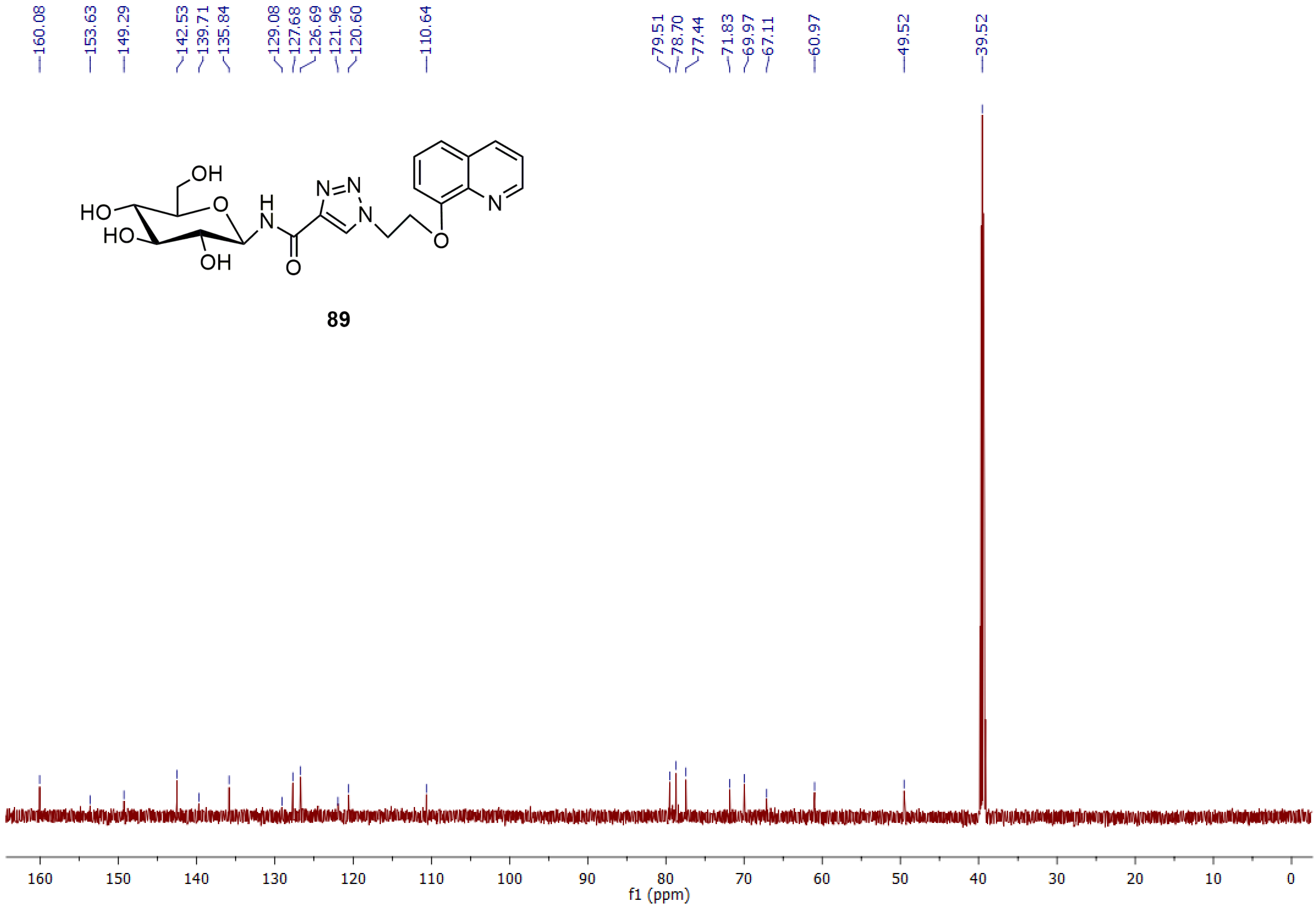

Fig. S170: ^{13}C NMR spectrum of glycoconjugate **89**.

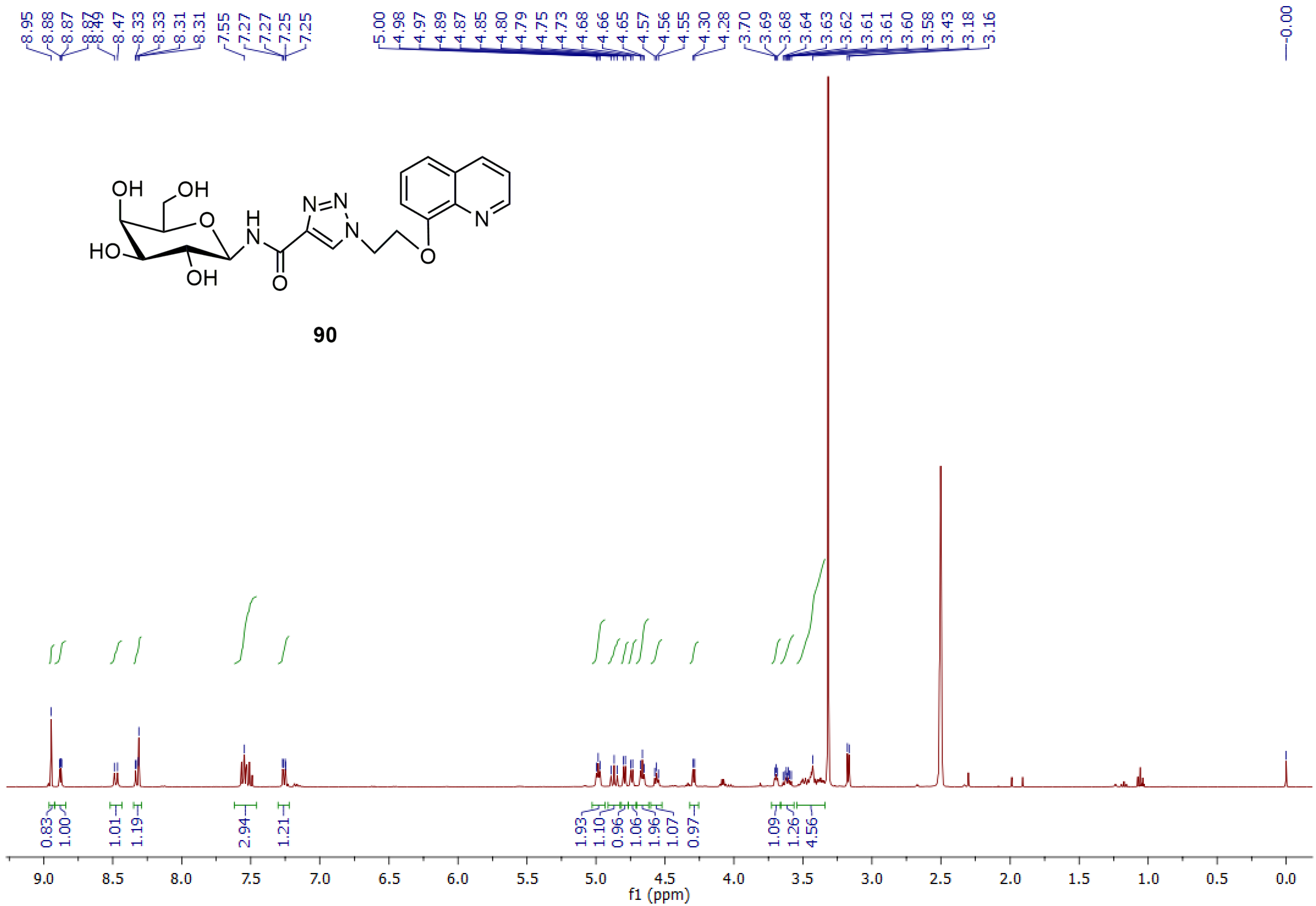

Fig. S171: ^{1}H NMR spectrum of glycoconjugate **90**.

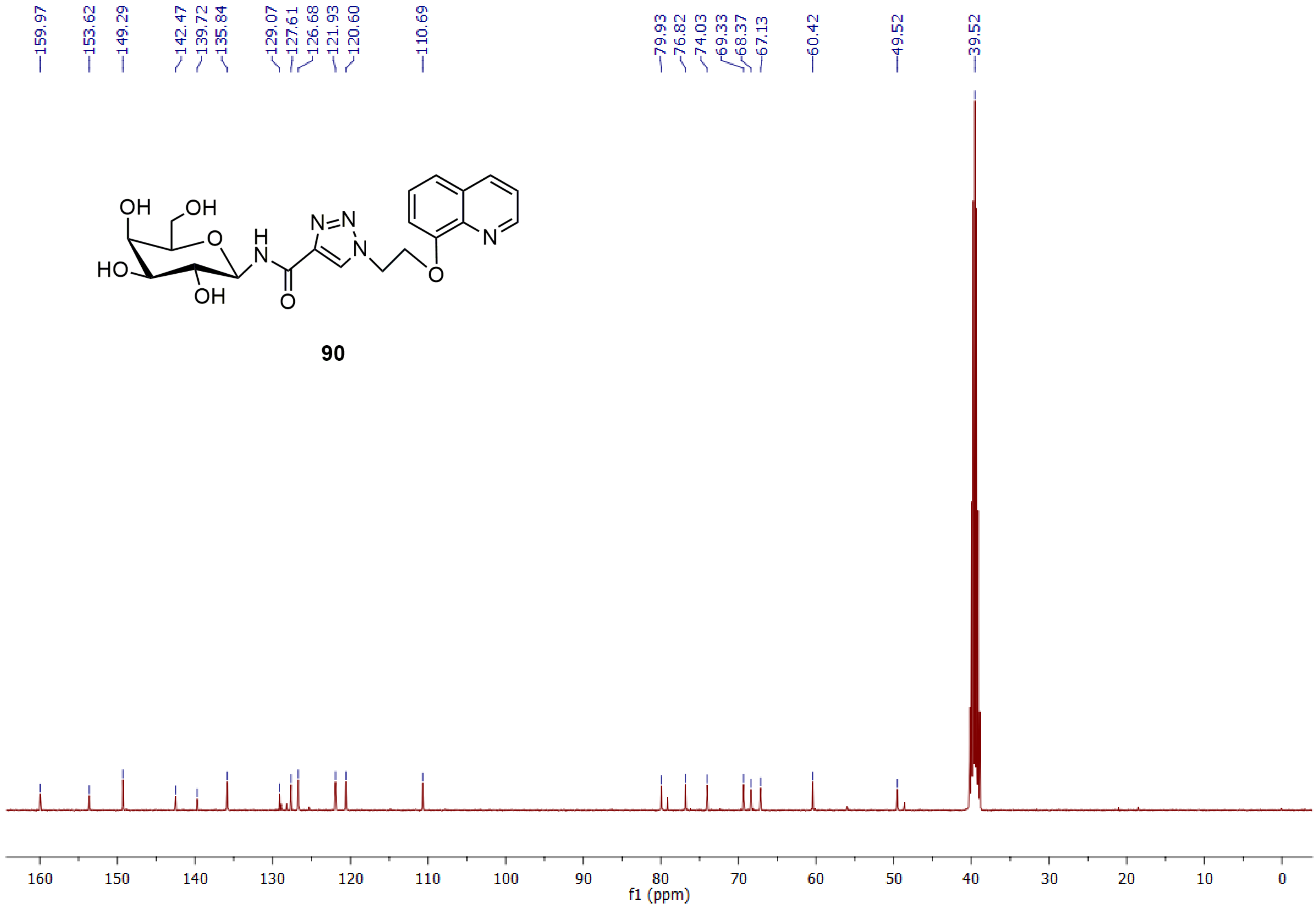

Fig. S172: ^{13}C NMR spectrum of glycoconjugate **90**.

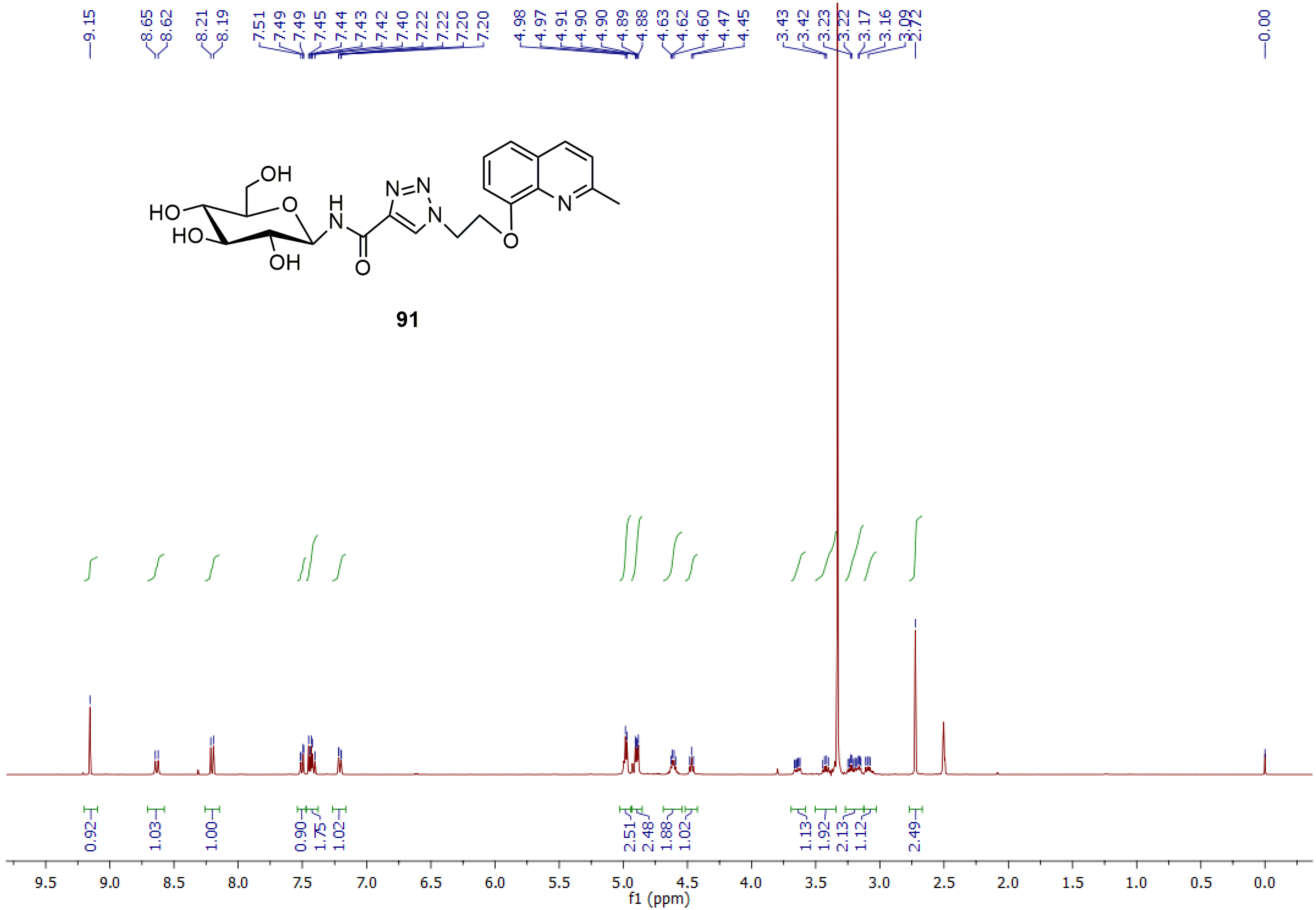

Fig. S173: ^{1}H NMR spectrum of glycoconjugate **91**.

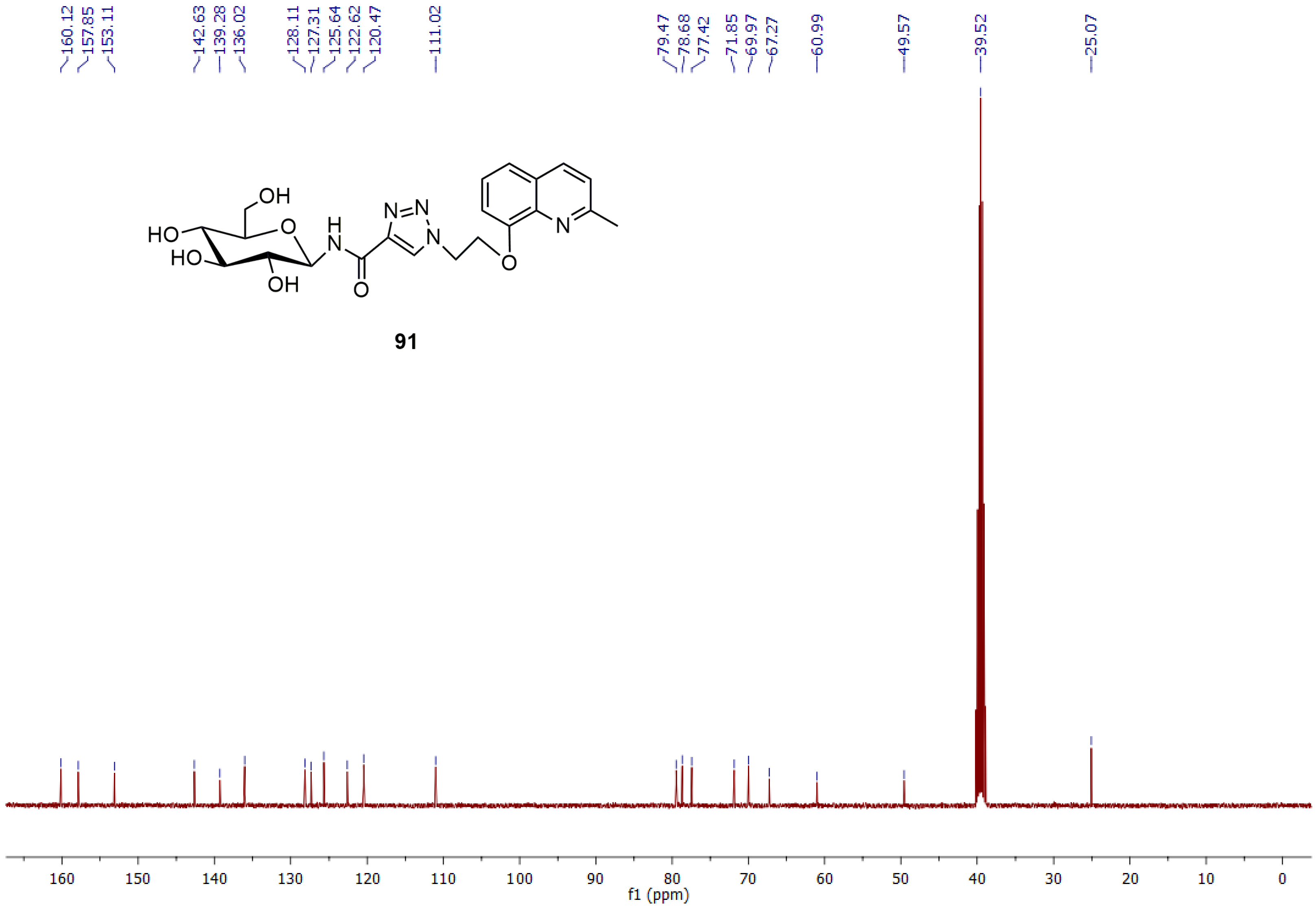

Fig. S174: ^{13}C NMR spectrum of glycoconjugate **91**.

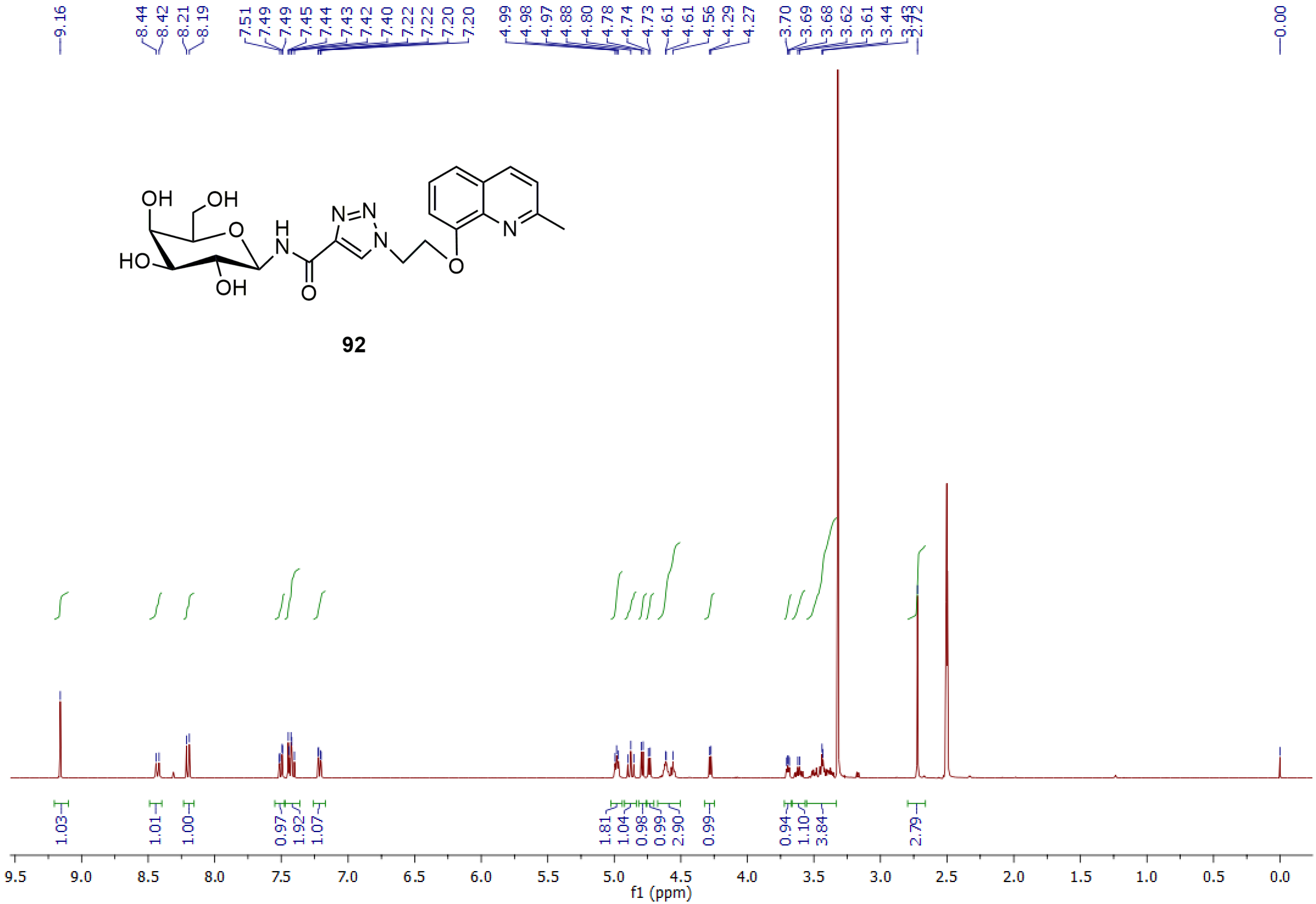

Fig. S175: ^{1}H NMR spectrum of glycoconjugate **92**.

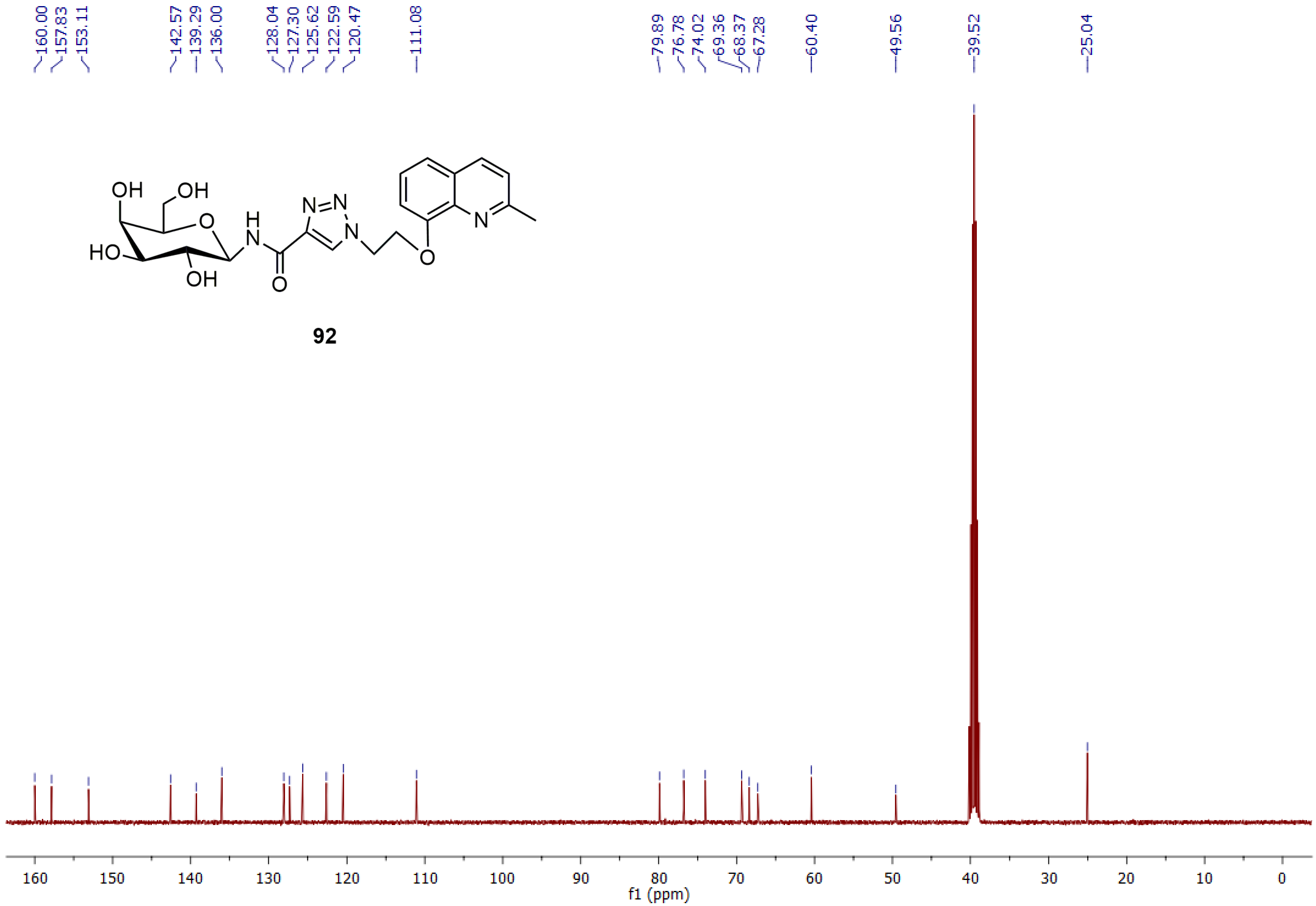

Fig. S176: ^{13}C NMR spectrum of glycoconjugate **92**.

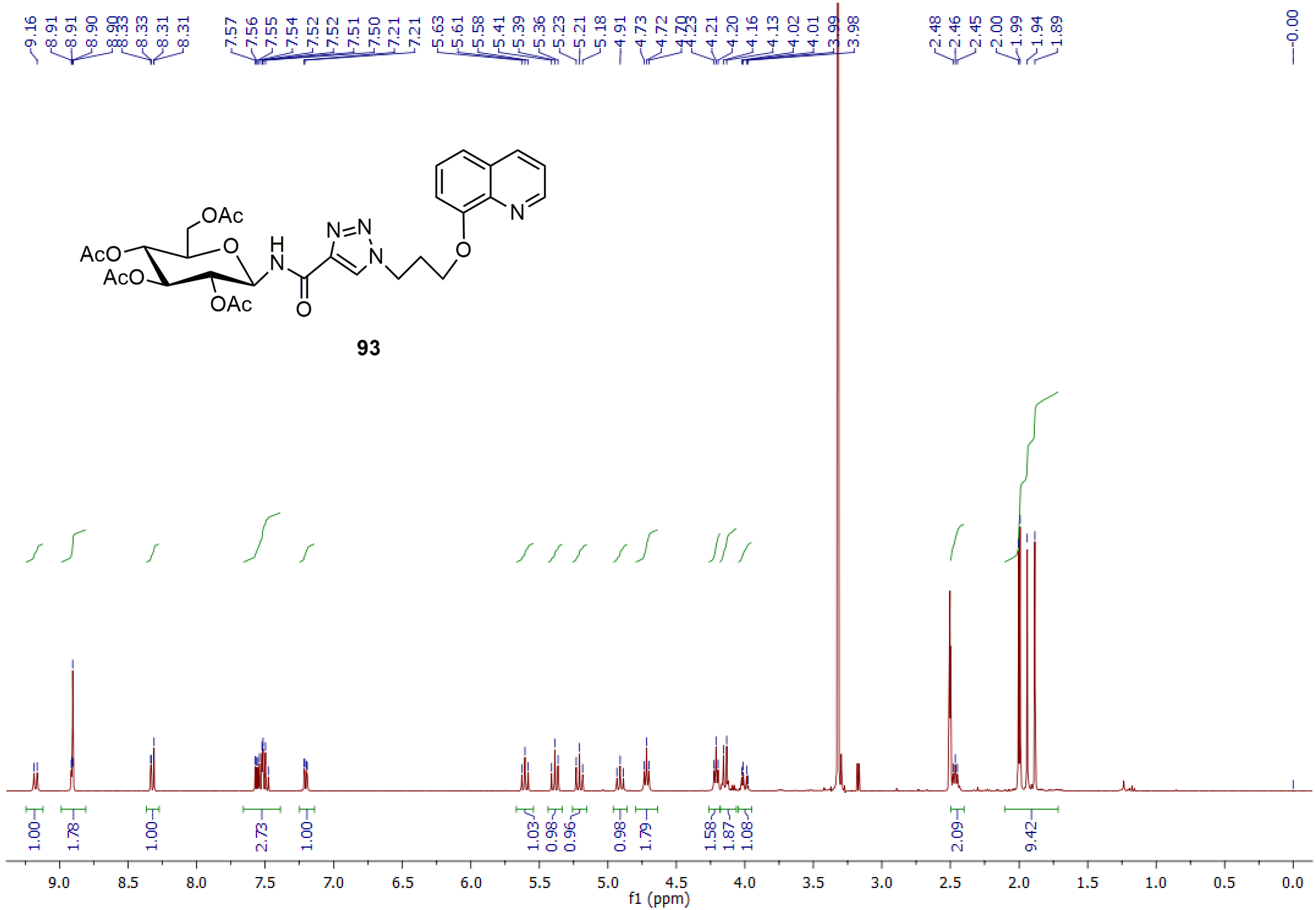

Fig. S177: ^{1}H NMR spectrum of glycoconjugate **93**.

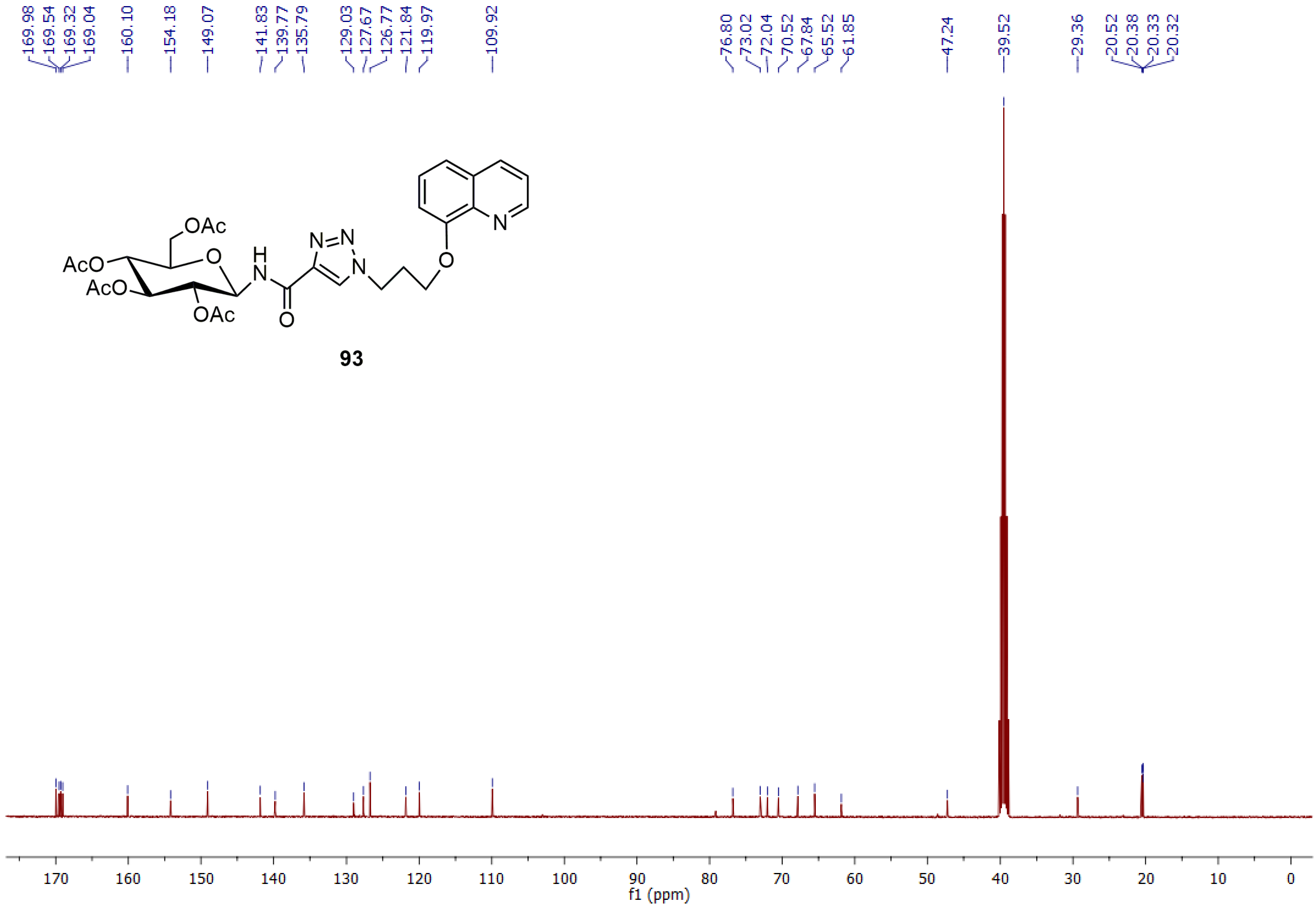

Fig. S178: ^{13}C NMR spectrum of glycoconjugate **93**.

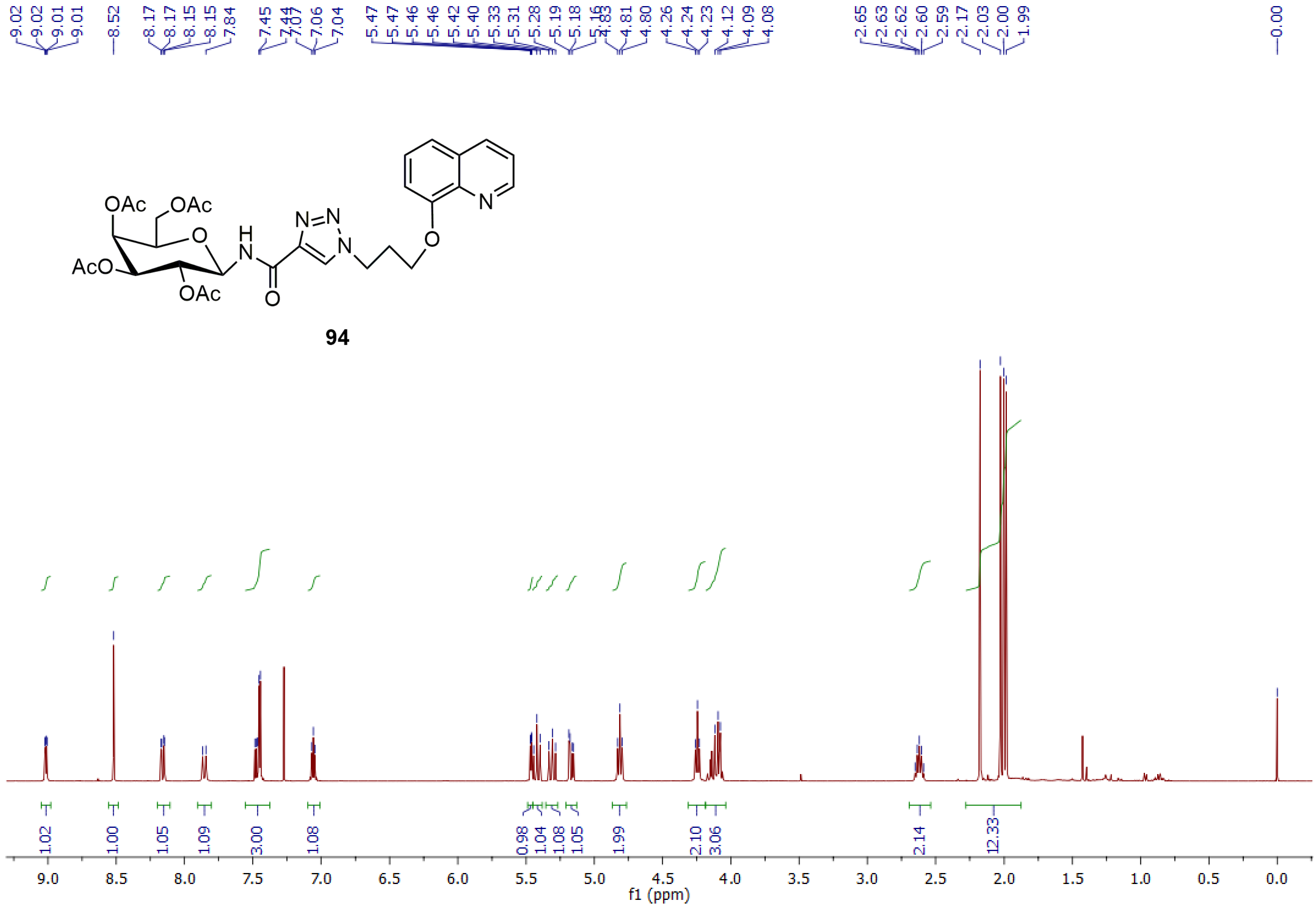

Fig. S179: ^{1}H NMR spectrum of glycoconjugate **94**.

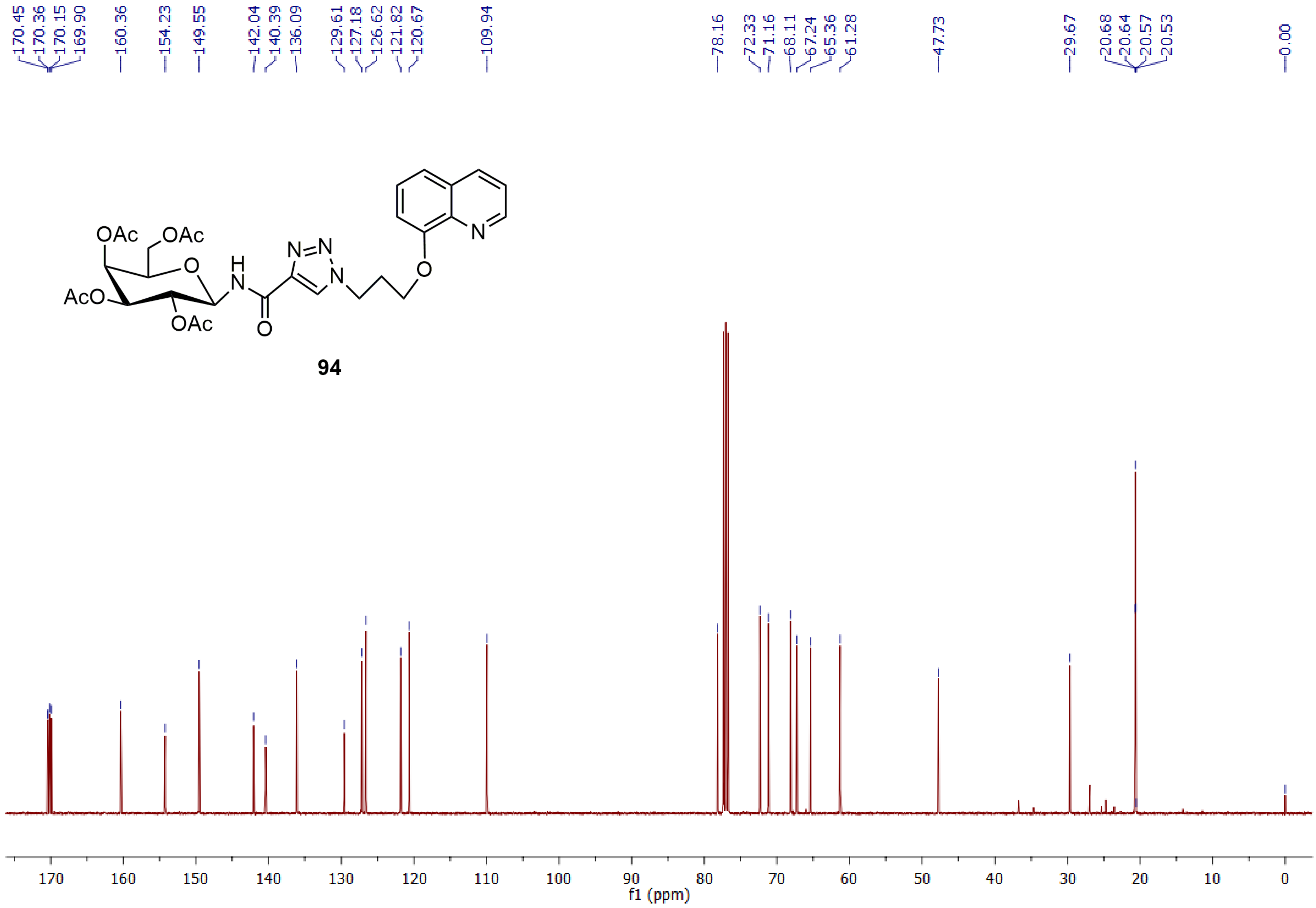

Fig. S180: ^{13}C NMR spectrum of glycoconjugate **94**.

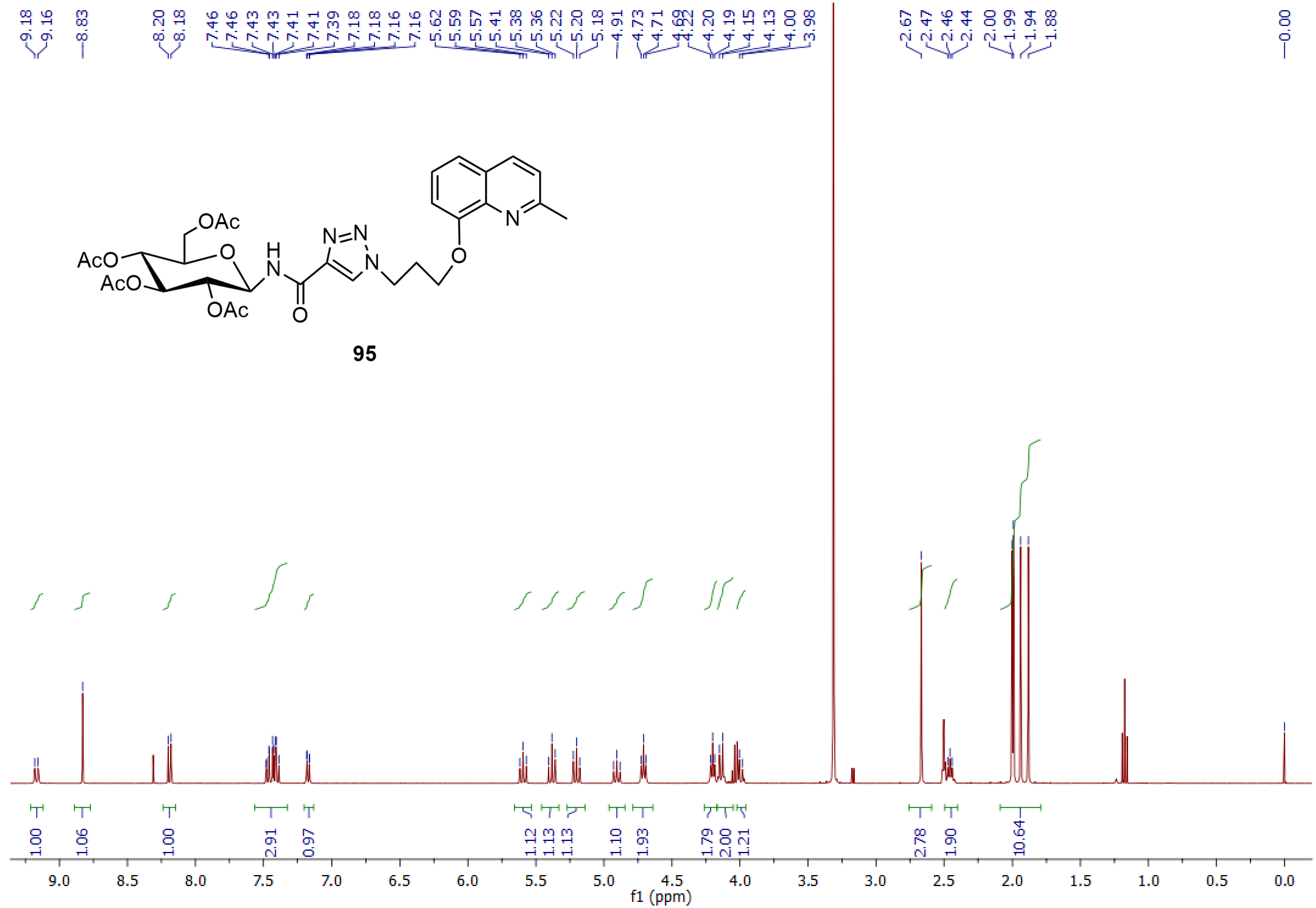

Fig. S181: ^{1}H NMR spectrum of glycoconjugate **95**.

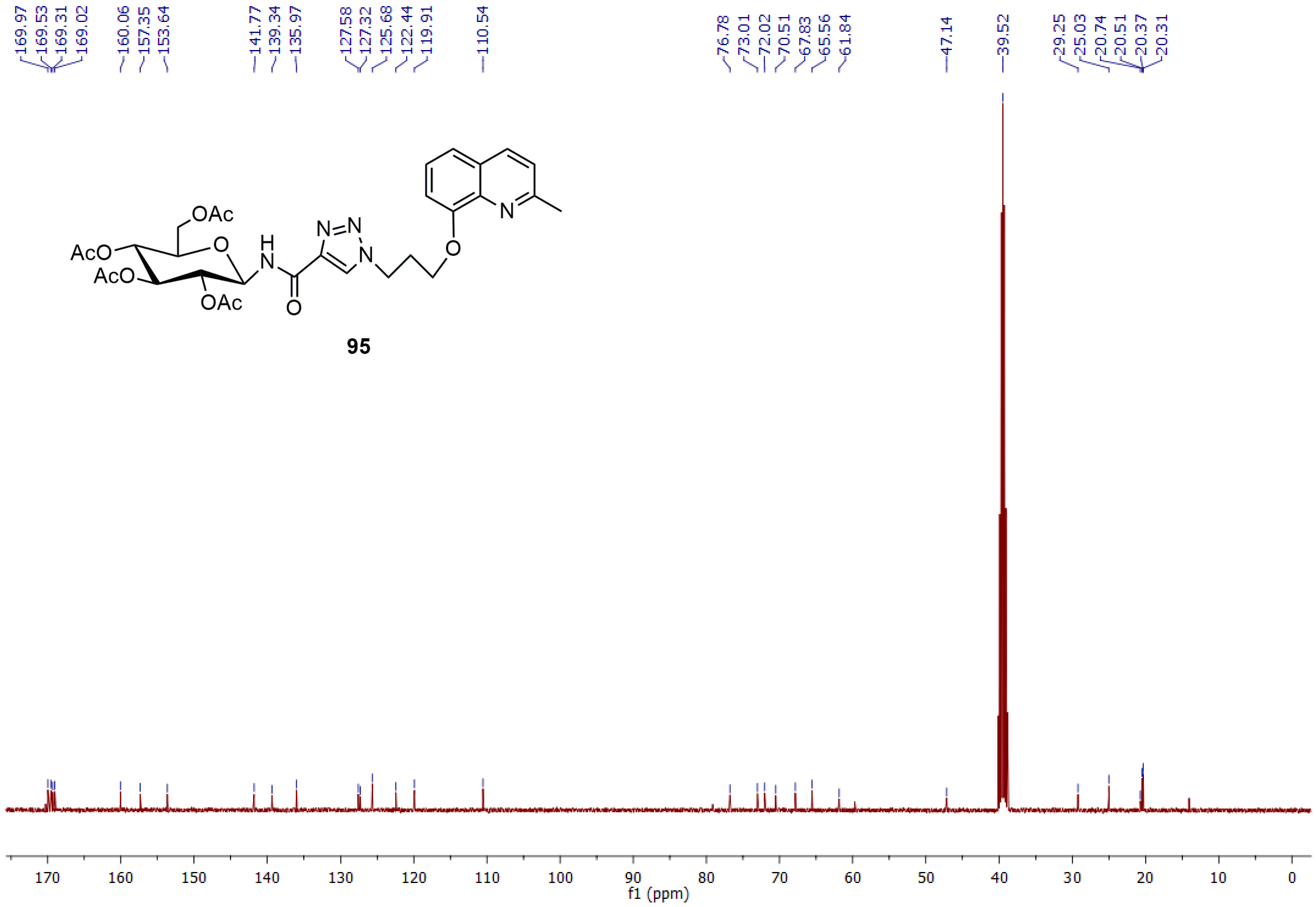

Fig. S182: ^{13}C NMR spectrum of glycoconjugate **95**.

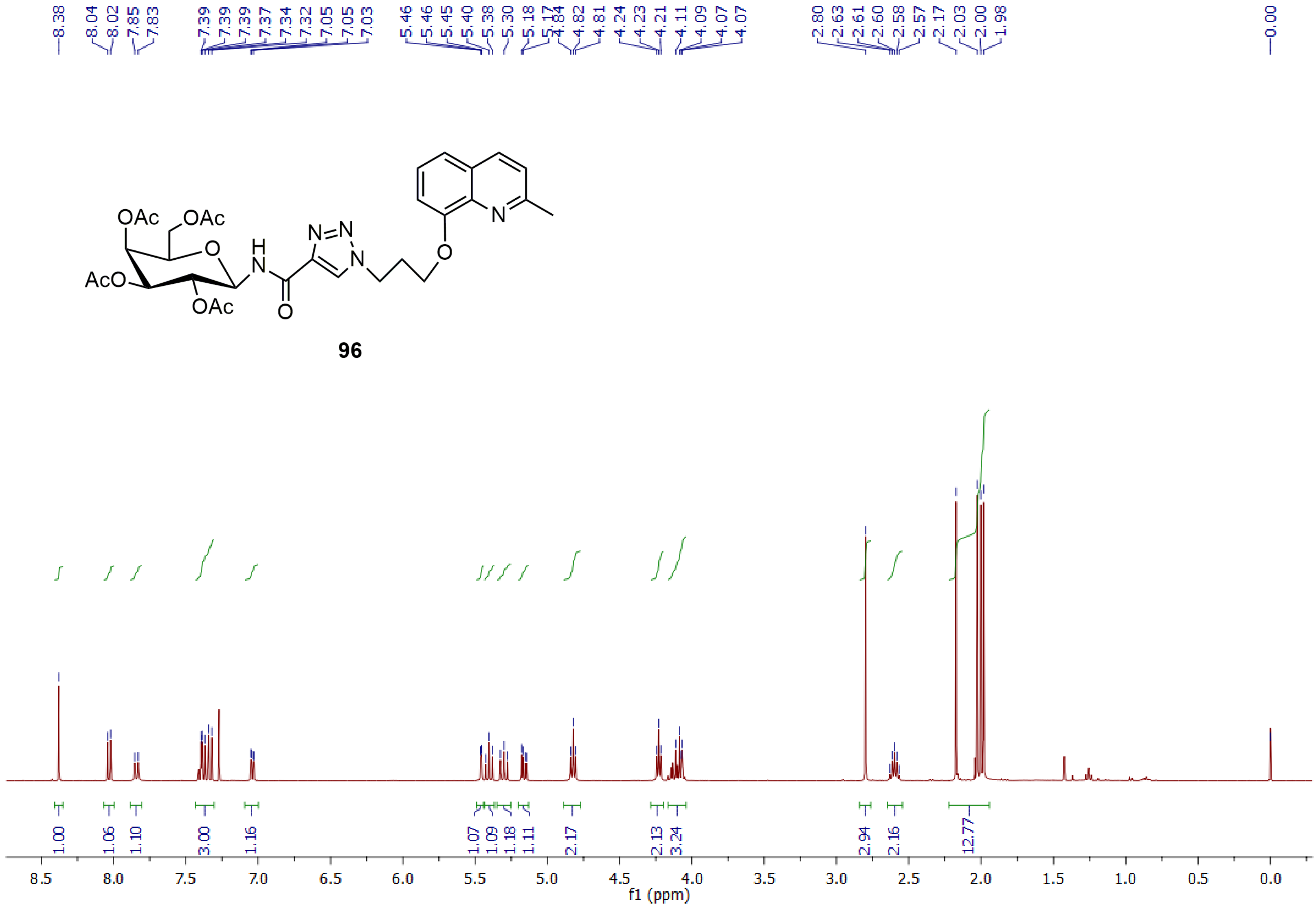

Fig. S183: ^{1}H NMR spectrum of glycoconjugate **96**.

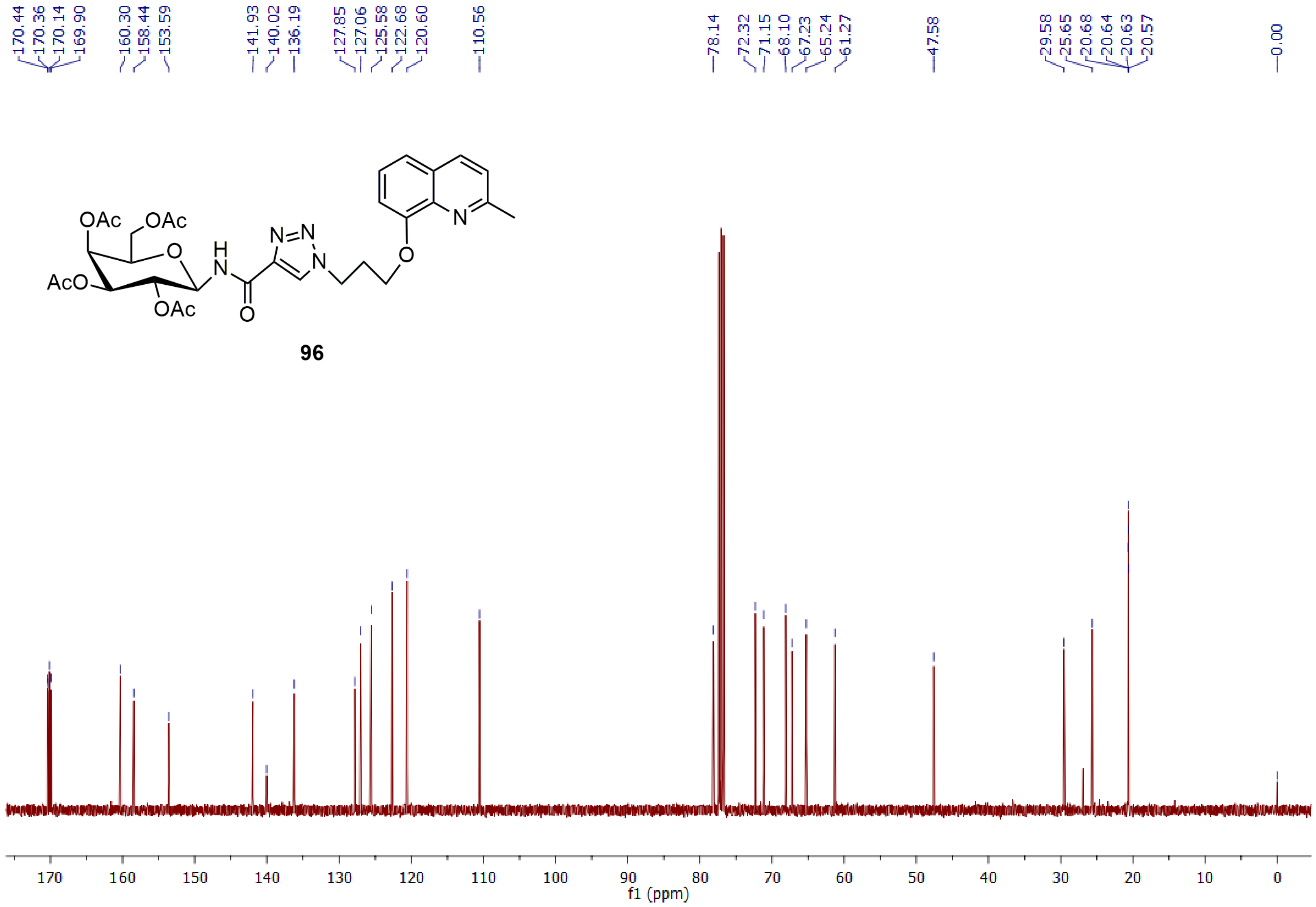

Fig. S184: ^{13}C NMR spectrum of glycoconjugate **96**.

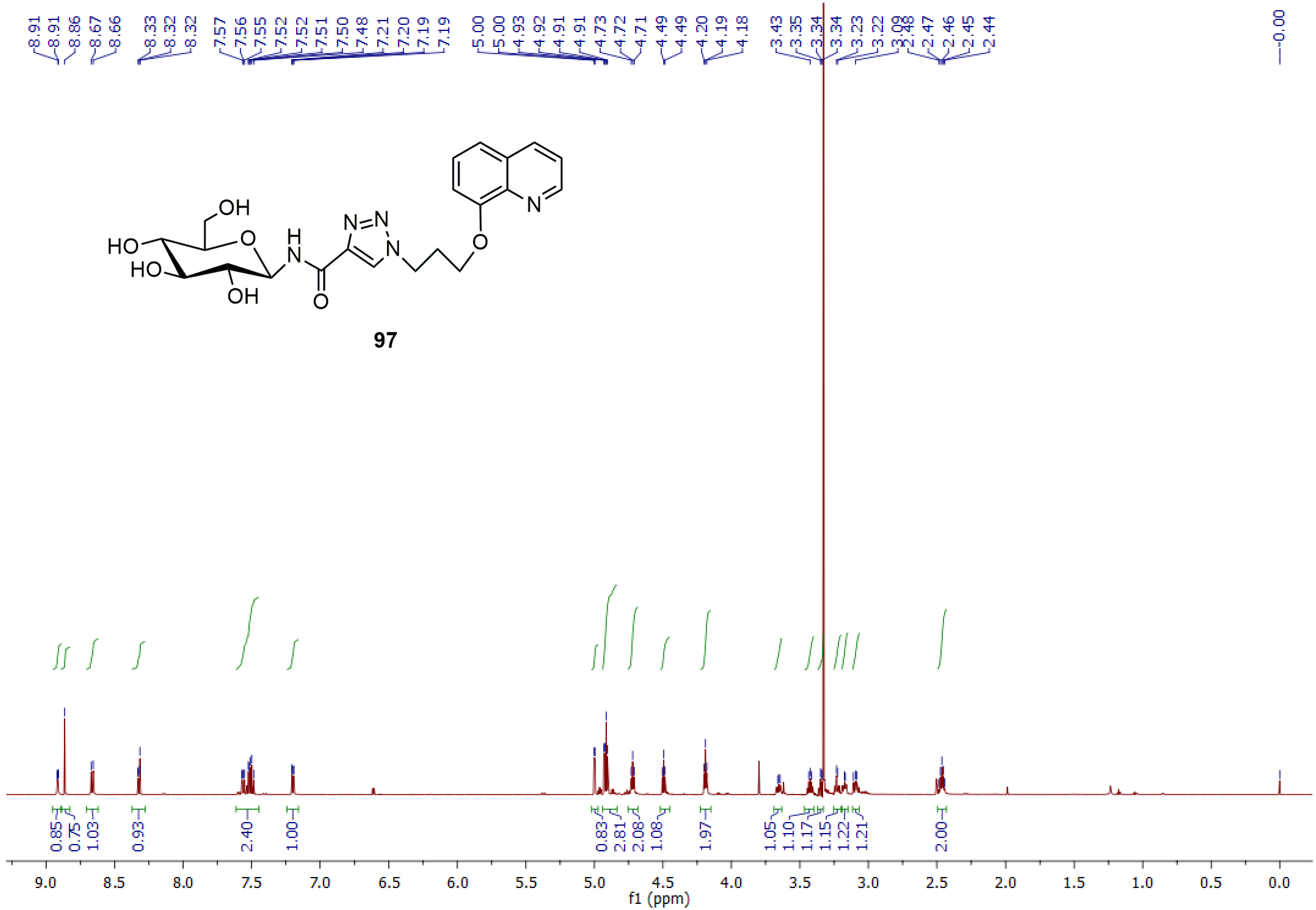

Fig. S185: ^{1}H NMR spectrum of glycoconjugate **97**.

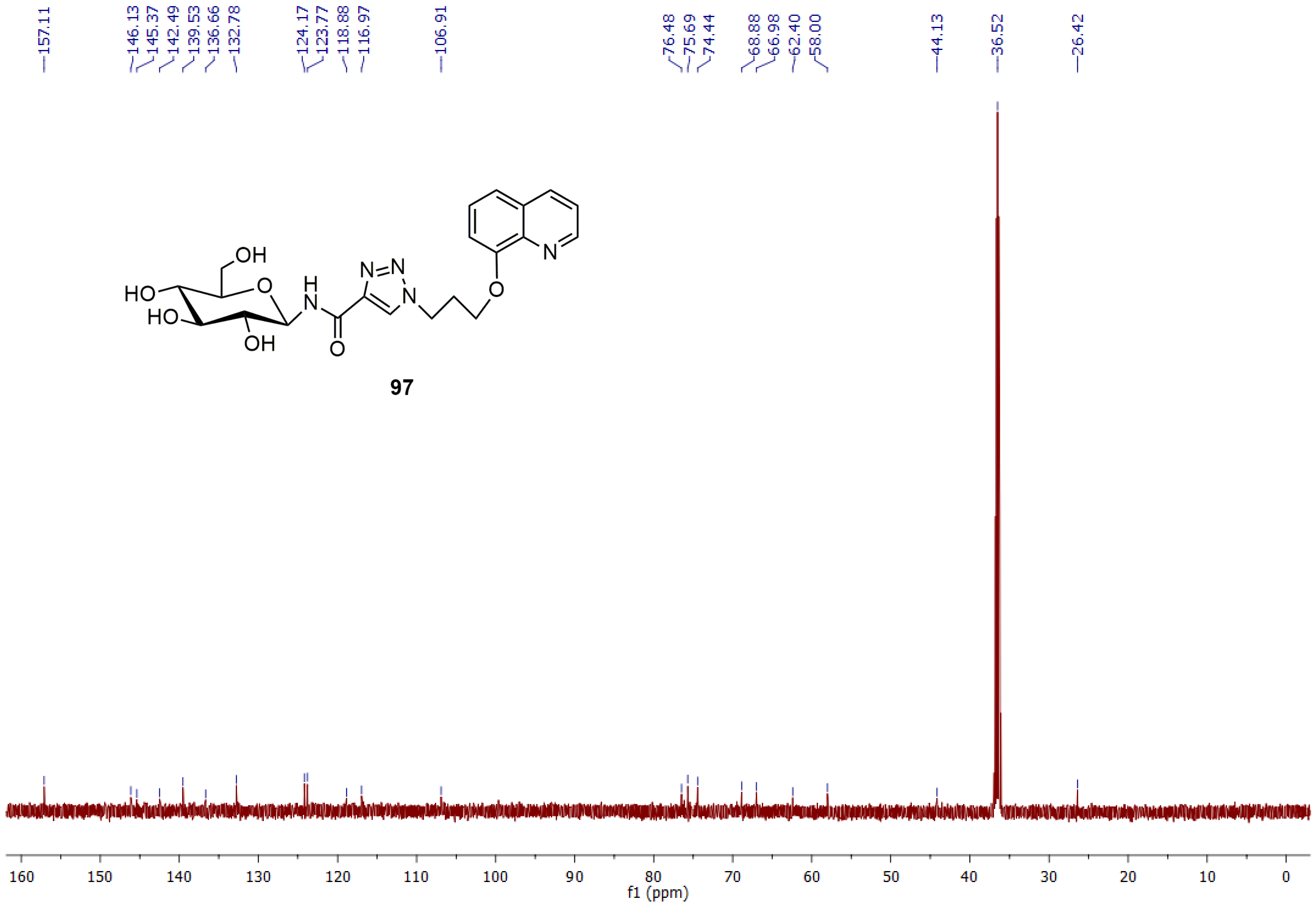

Fig. S186: ^{13}C NMR spectrum of glycoconjugate **97**.

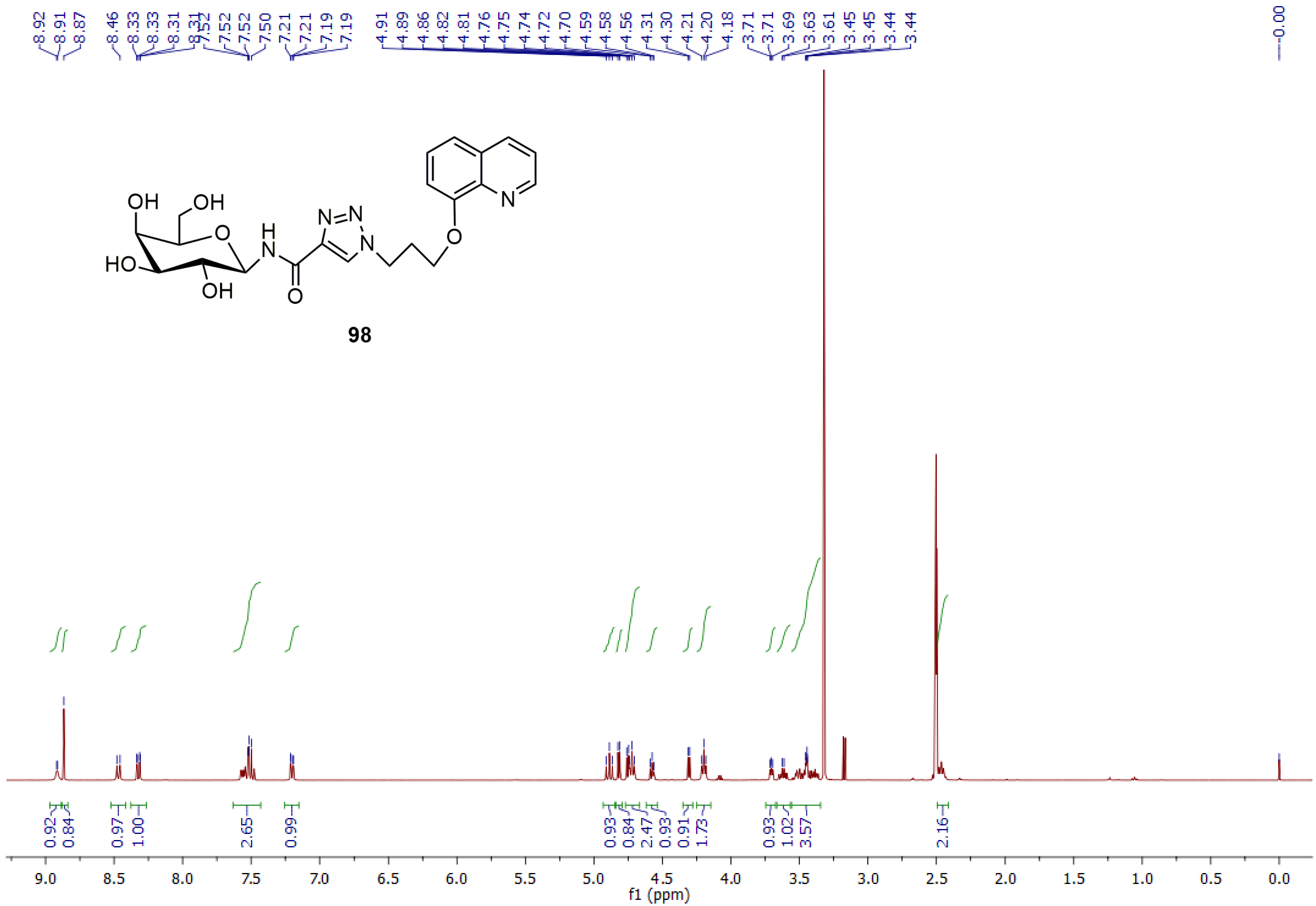

Fig. S187: ^{1}H NMR spectrum of glycoconjugate **98**.

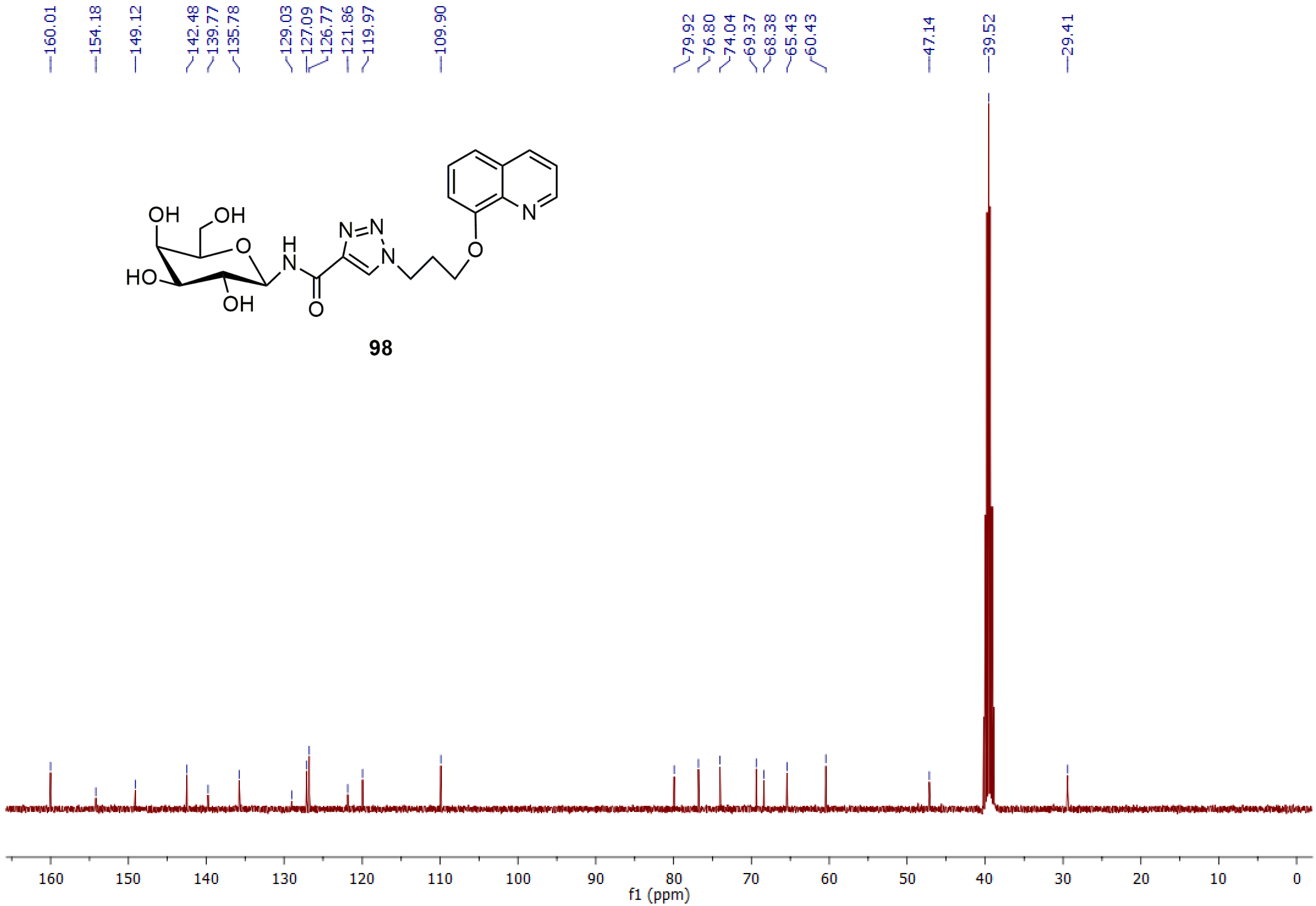

Fig. S188: ^{13}C NMR spectrum of glycoconjugate **98**.

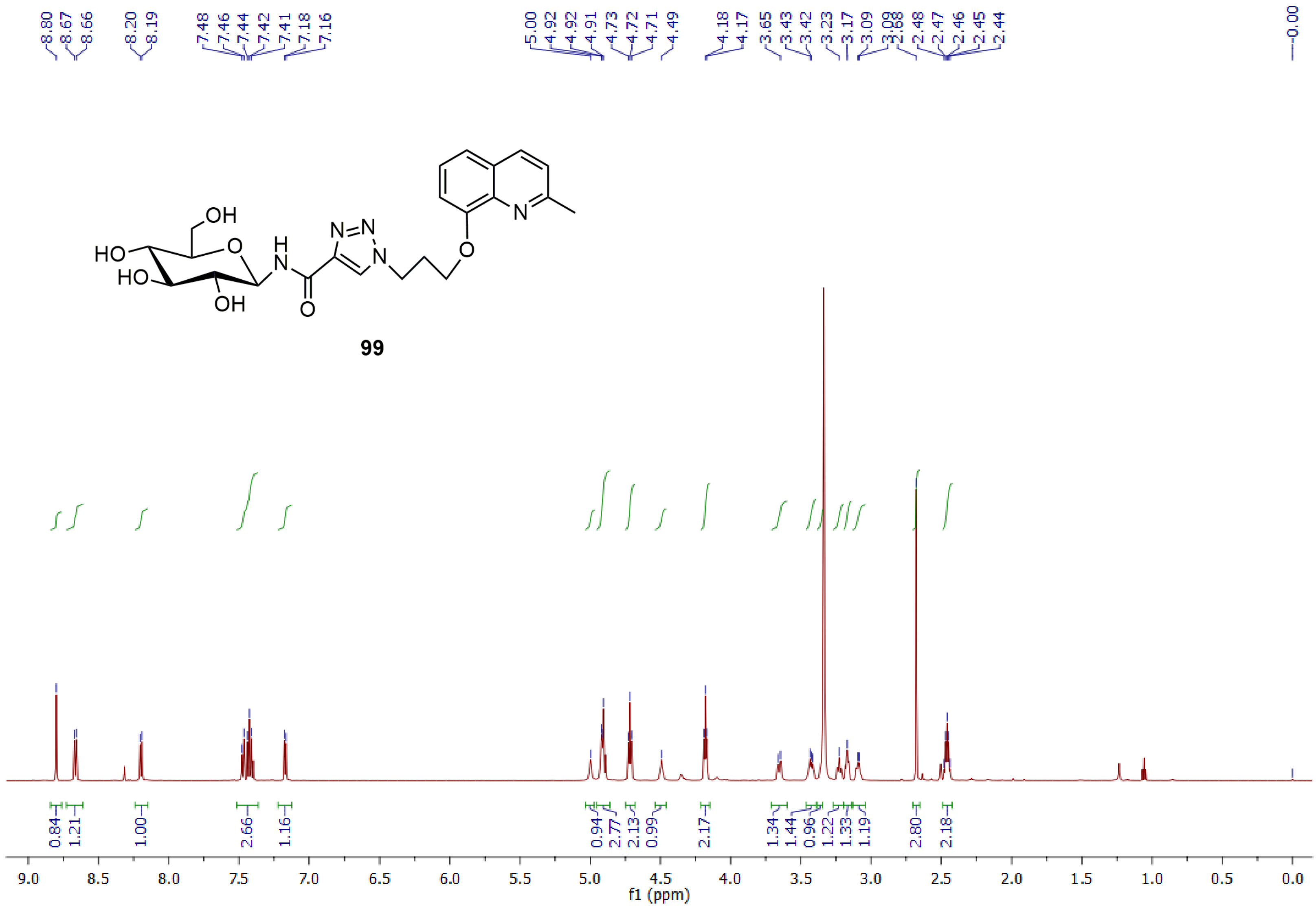

Fig. S189: ^{1}H NMR spectrum of glycoconjugate **99**.

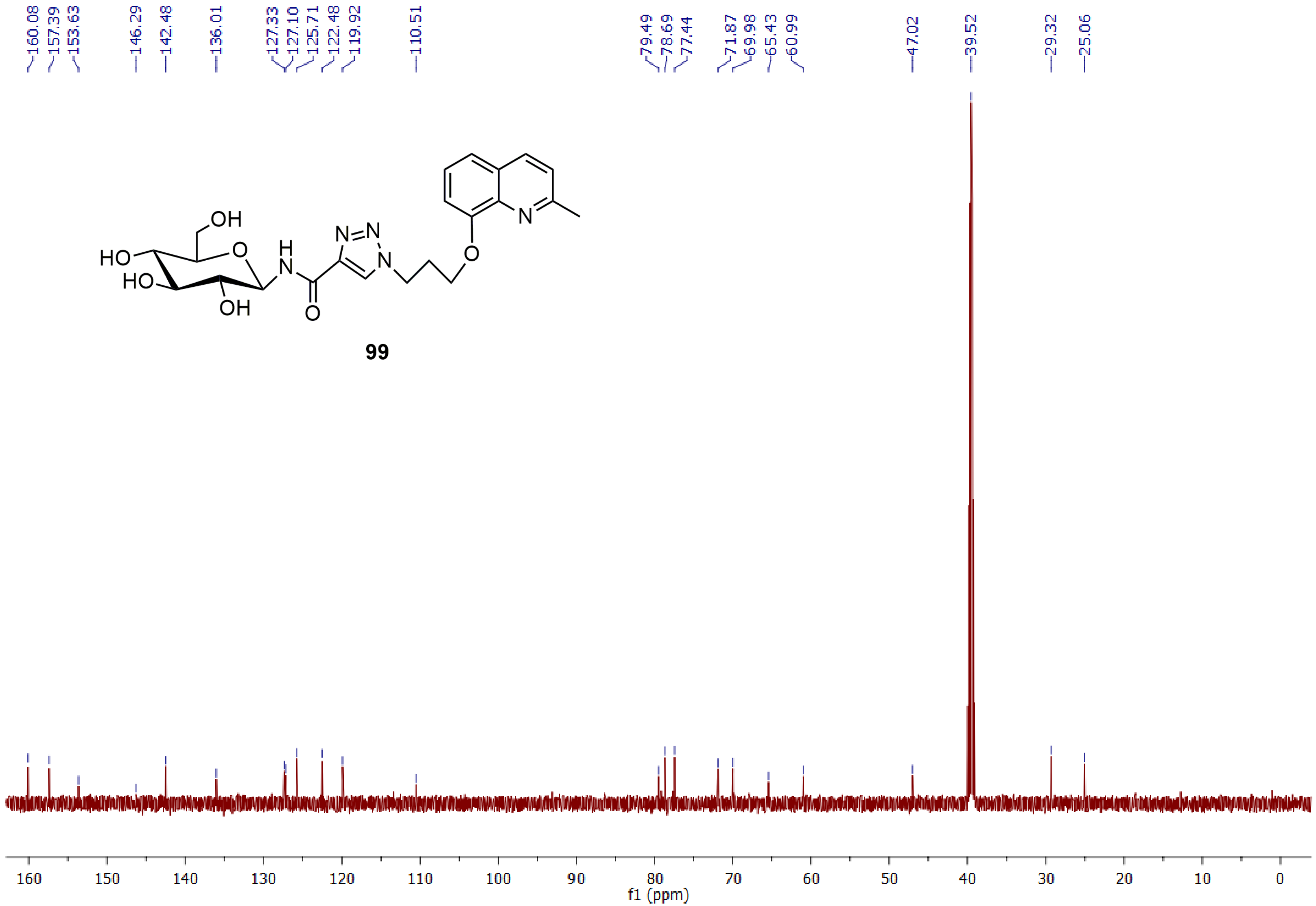

Fig. S190: ^{13}C NMR spectrum of glycoconjugate **99**.

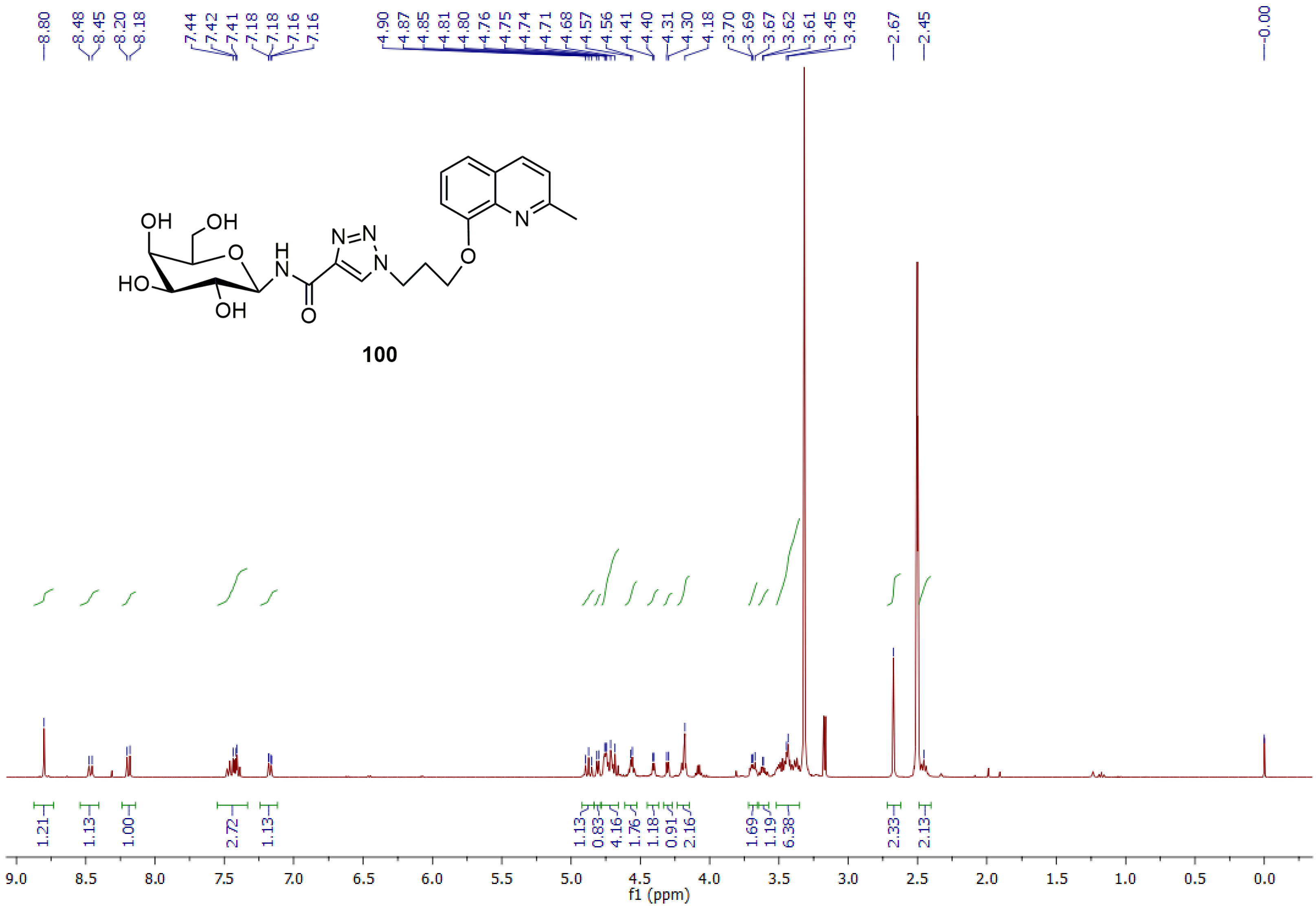

Fig. S191: ^{1}H NMR spectrum of glycoconjugate **100**.

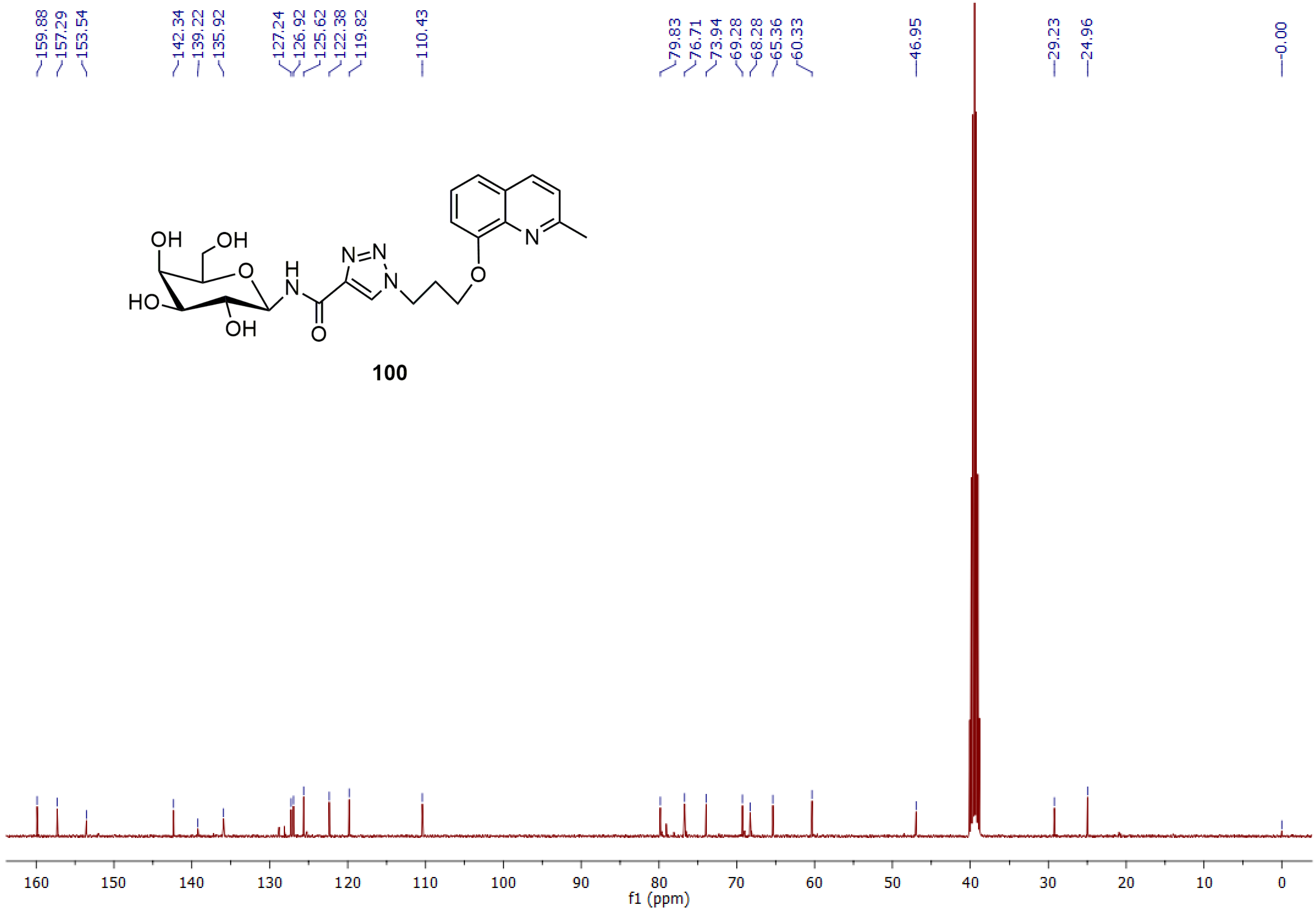

Fig. S192: ^{13}C NMR spectrum of glycoconjugate **100**.

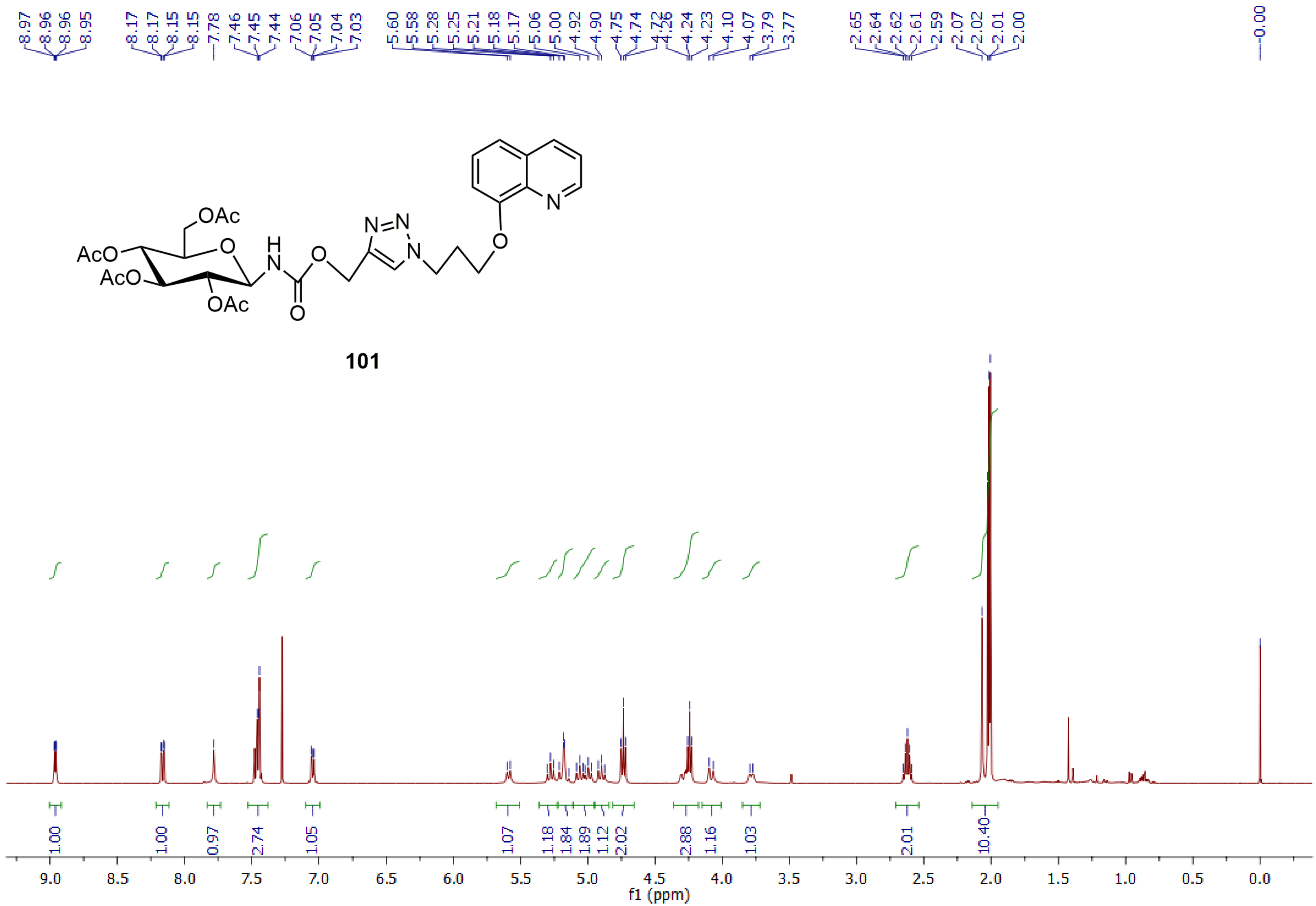

Fig. S193: ^{1}H NMR spectrum of glycoconjugate **101**.

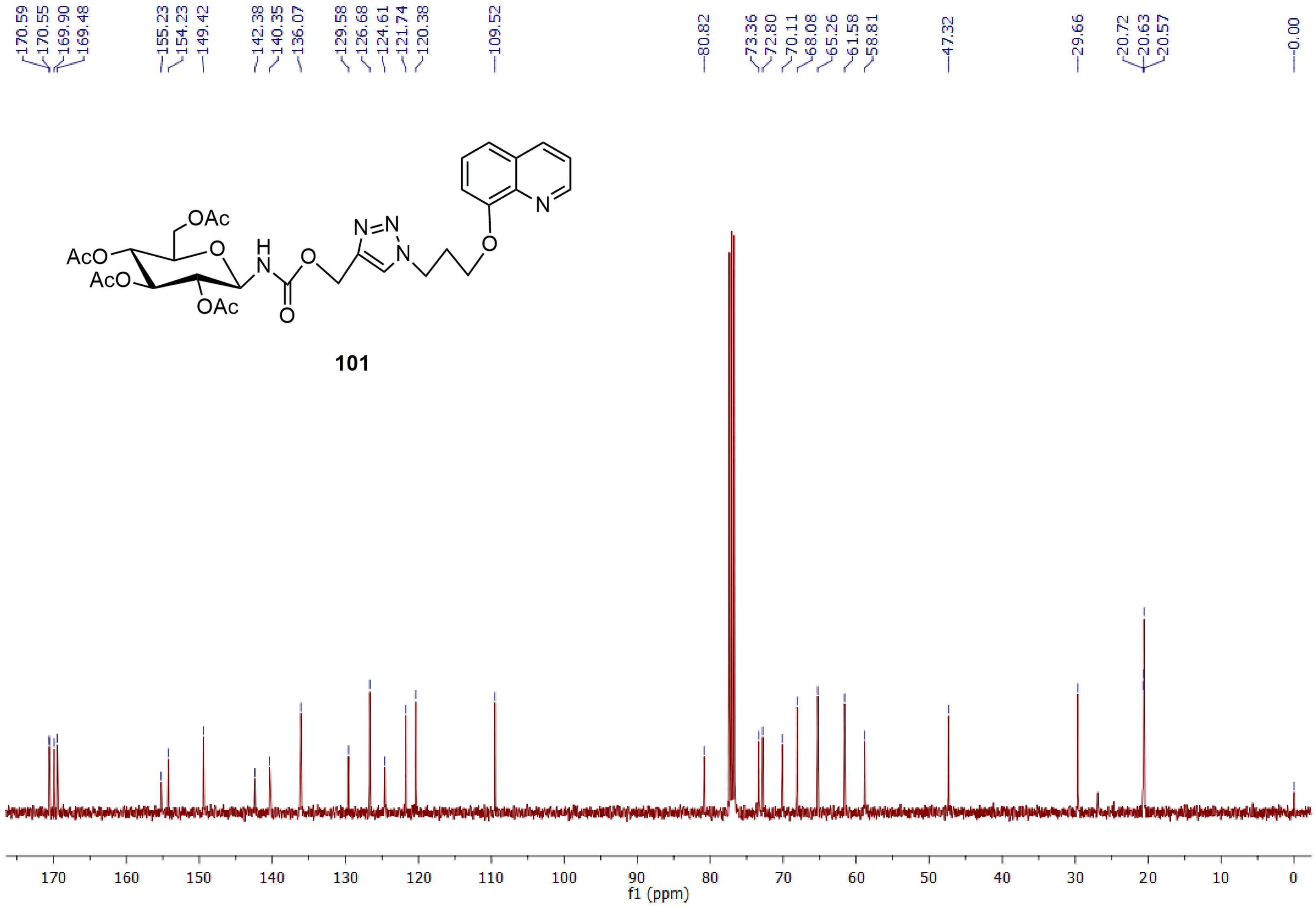

Fig. S194: ^{13}C NMR spectrum of glycoconjugate **101**.

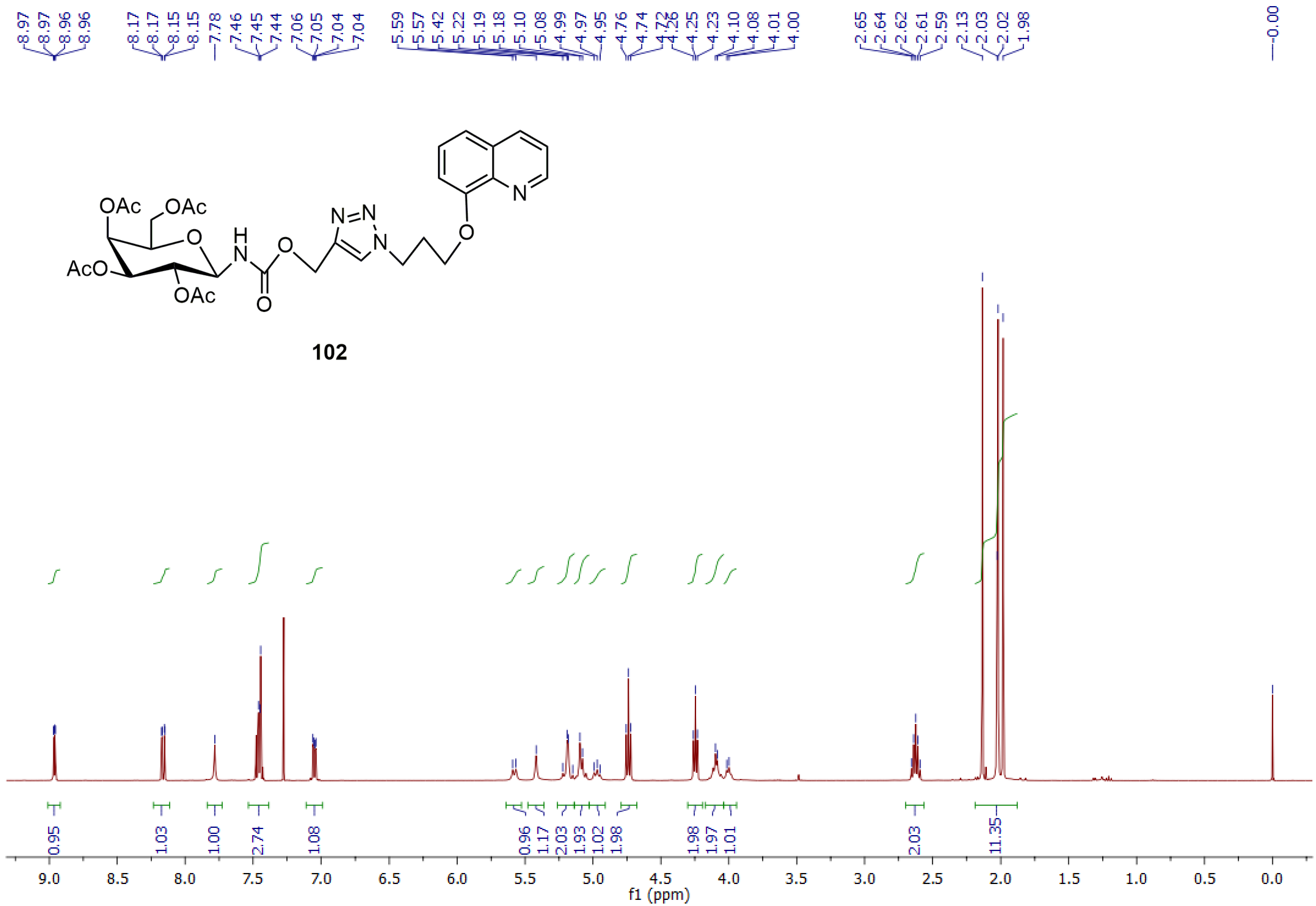

Fig. S195: ^{1}H NMR spectrum of glycoconjugate **102**.

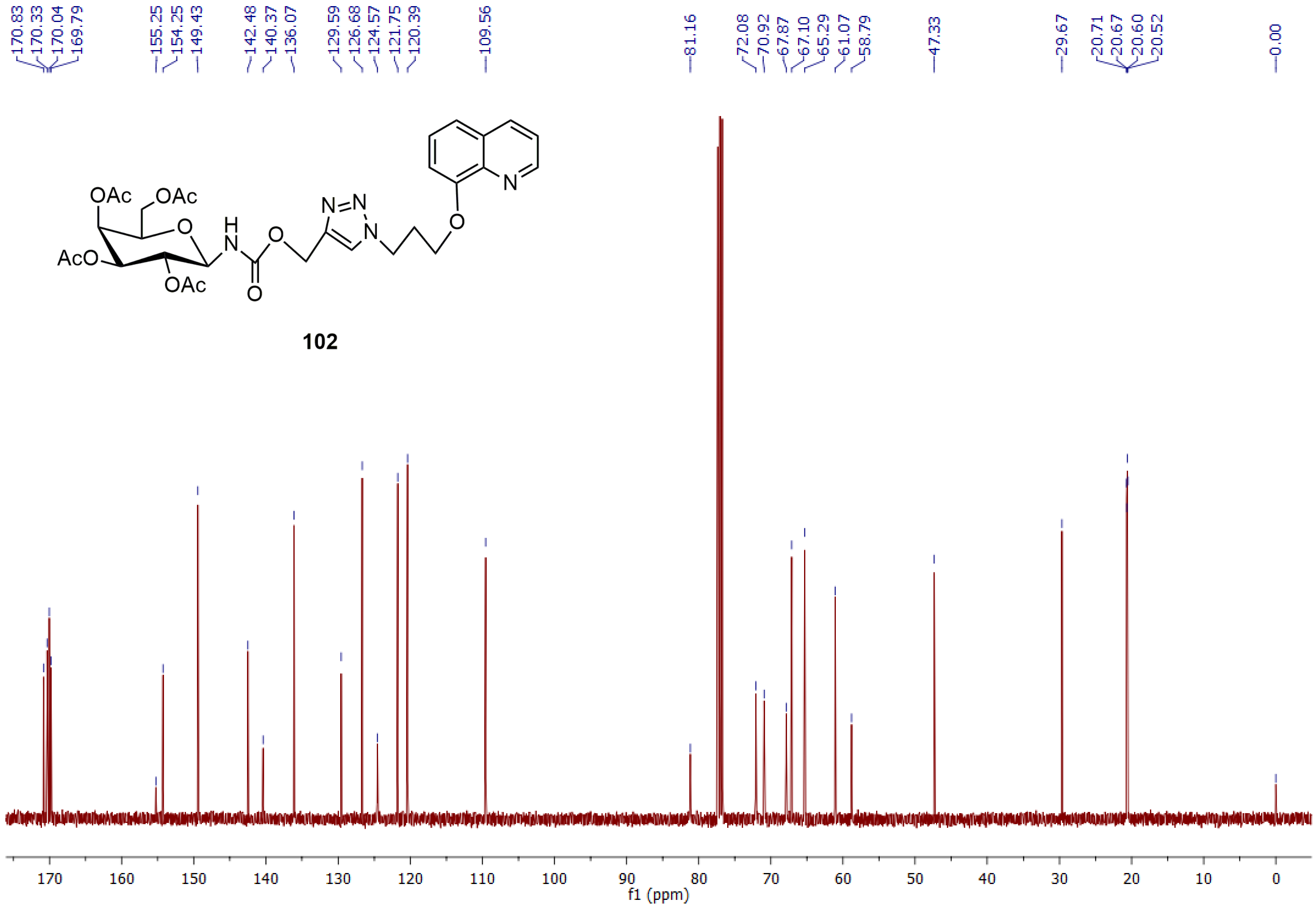

Fig. S196: ^{13}C NMR spectrum of glycoconjugate **102**.

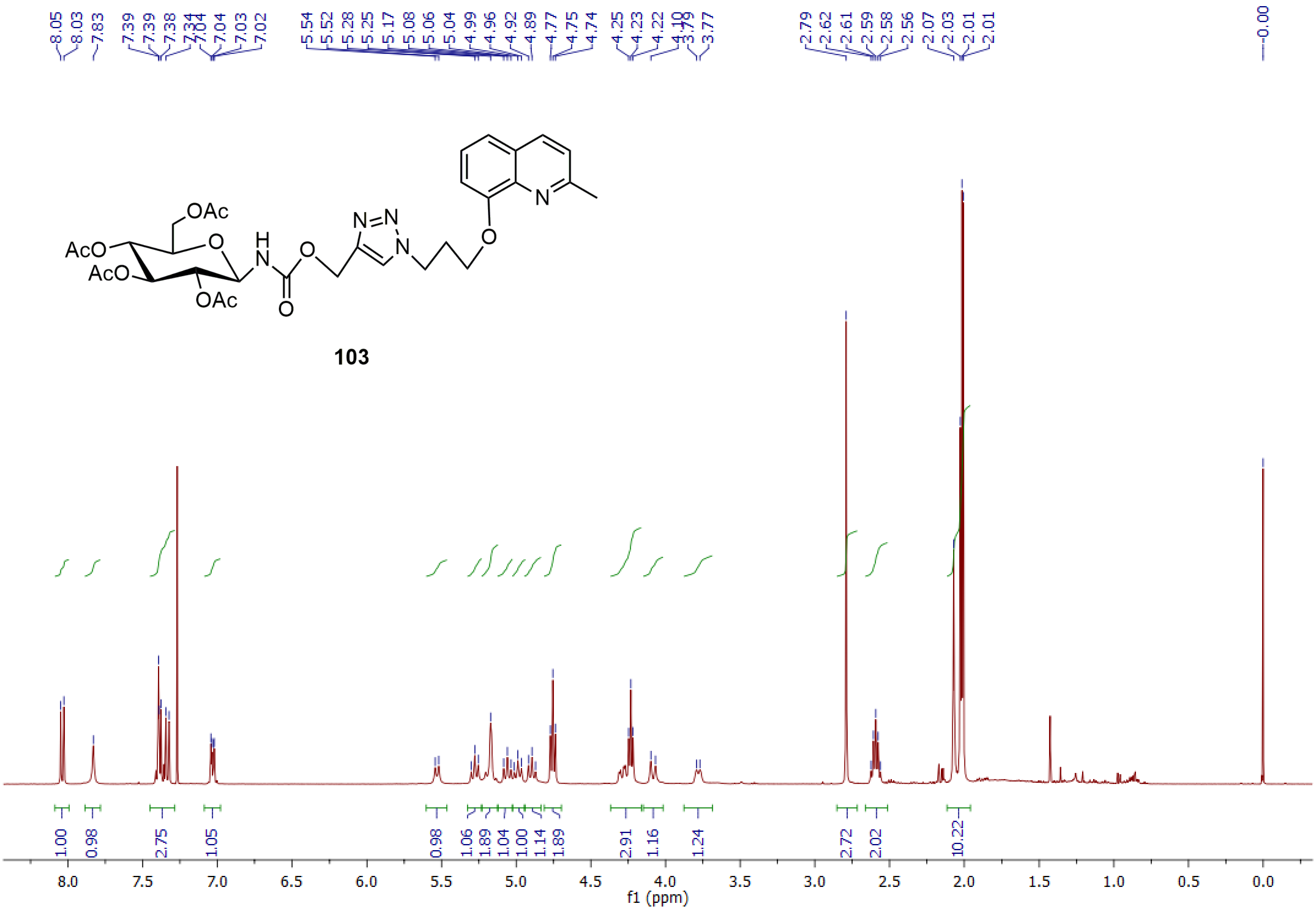

Fig. S197: ^{1}H NMR spectrum of glycoconjugate **103**.

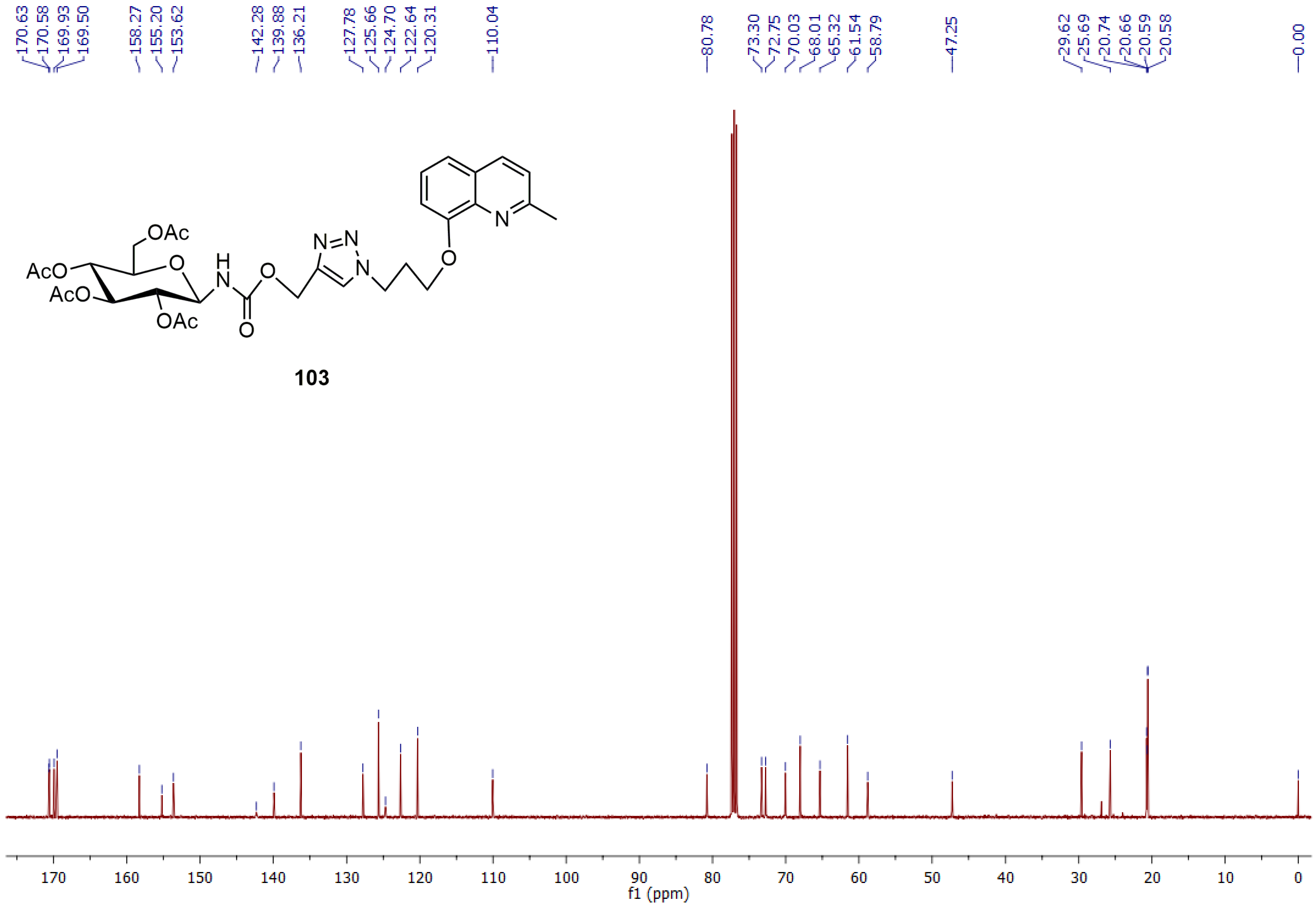

Fig. S198: ^{13}C NMR spectrum of glycoconjugate **103**.

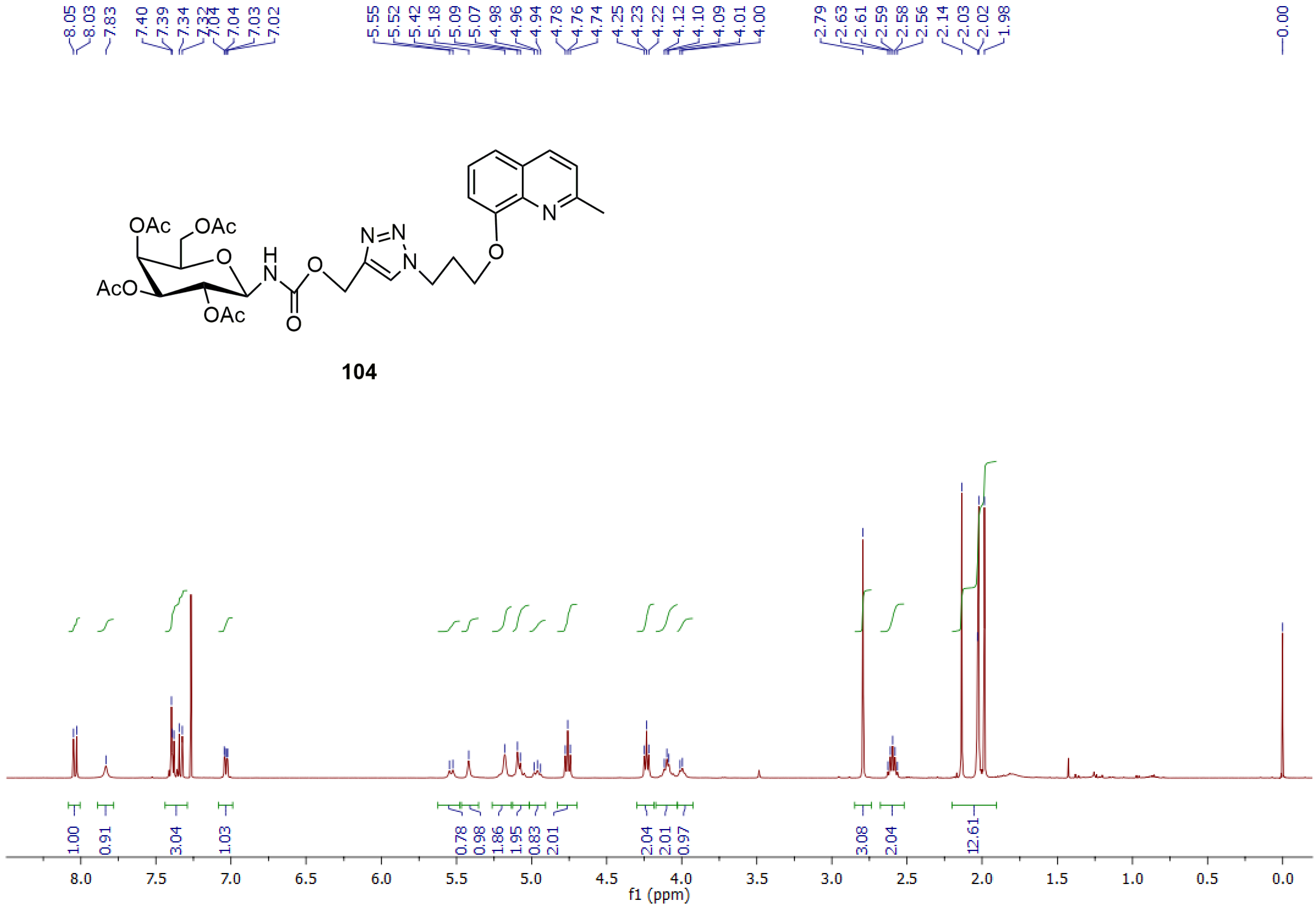

Fig. S199: ^{1}H NMR spectrum of glycoconjugate **104**.

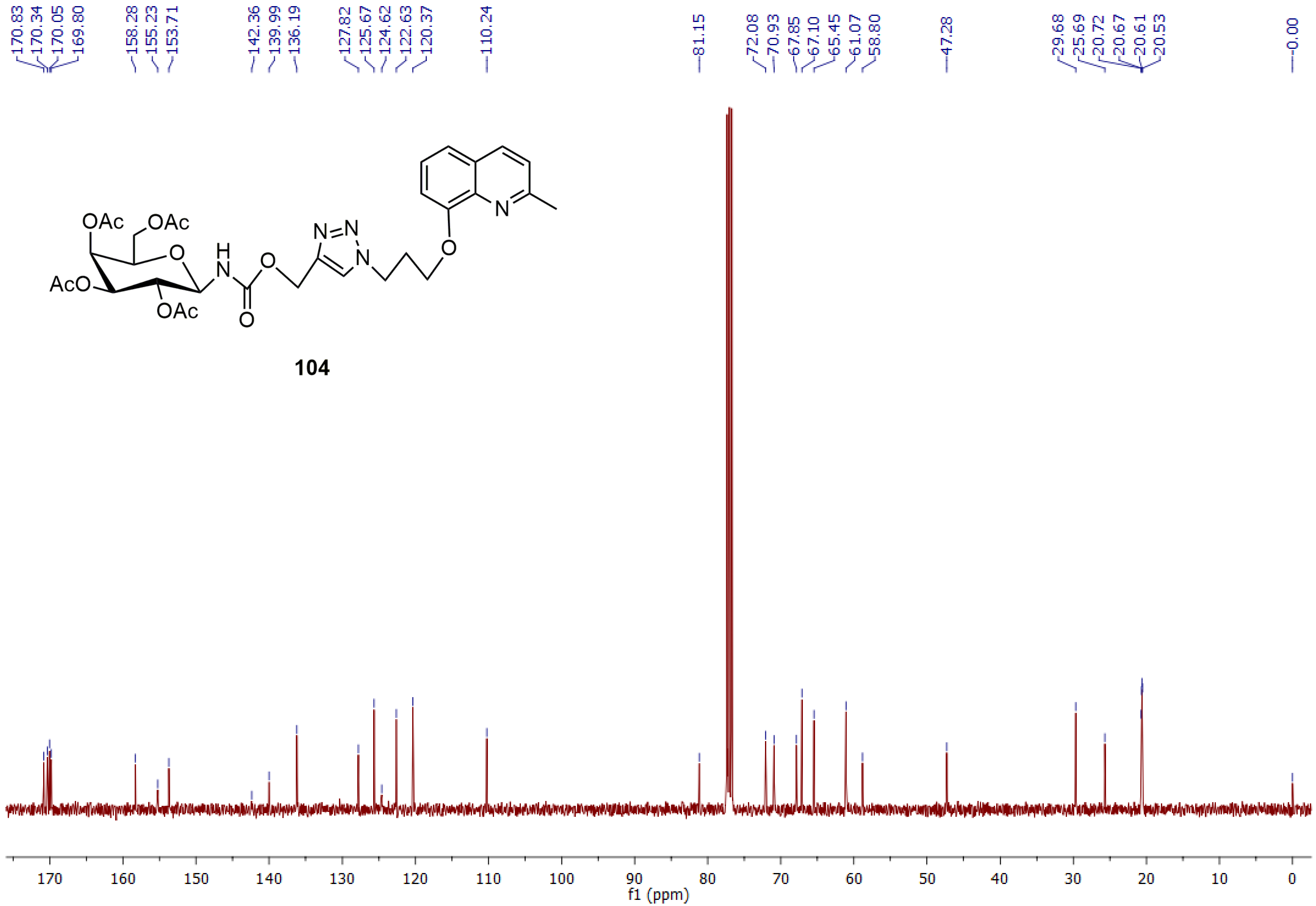

Fig. S200: ^{13}C NMR spectrum of glycoconjugate **104**.

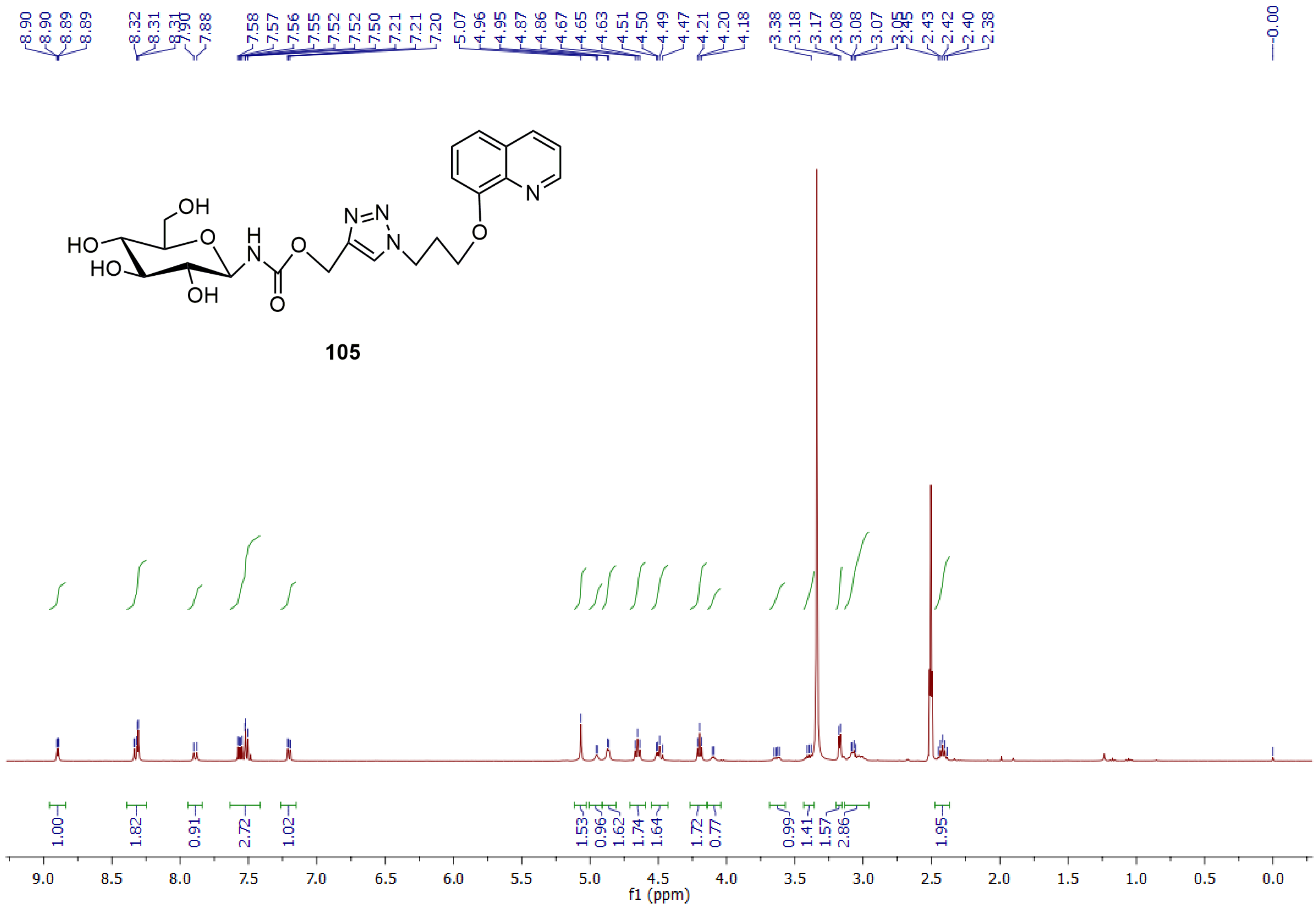

Fig. S201: ^{1}H NMR spectrum of glycoconjugate **105**.

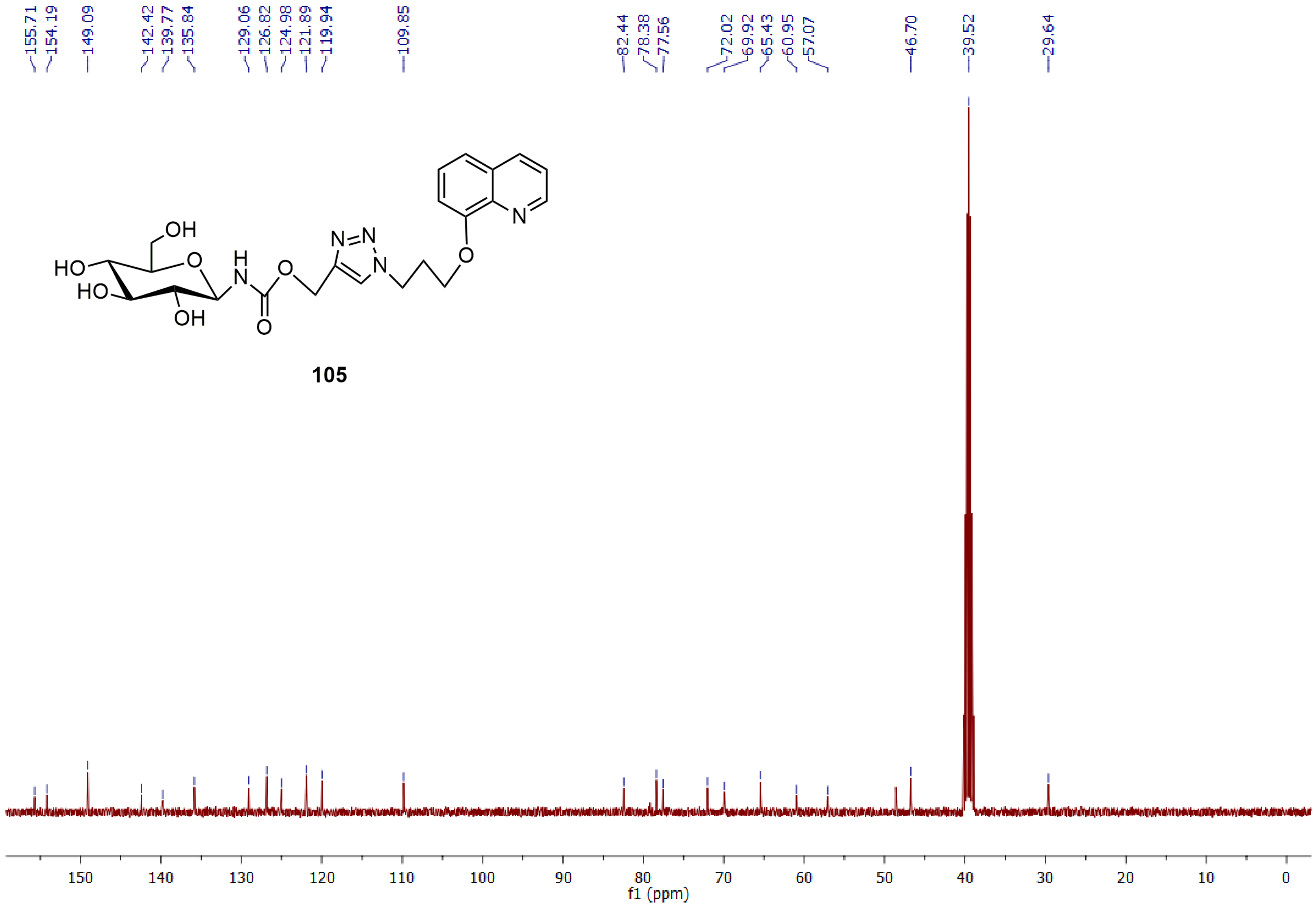

Fig. S202: ^{13}C NMR spectrum of glycoconjugate **105**.

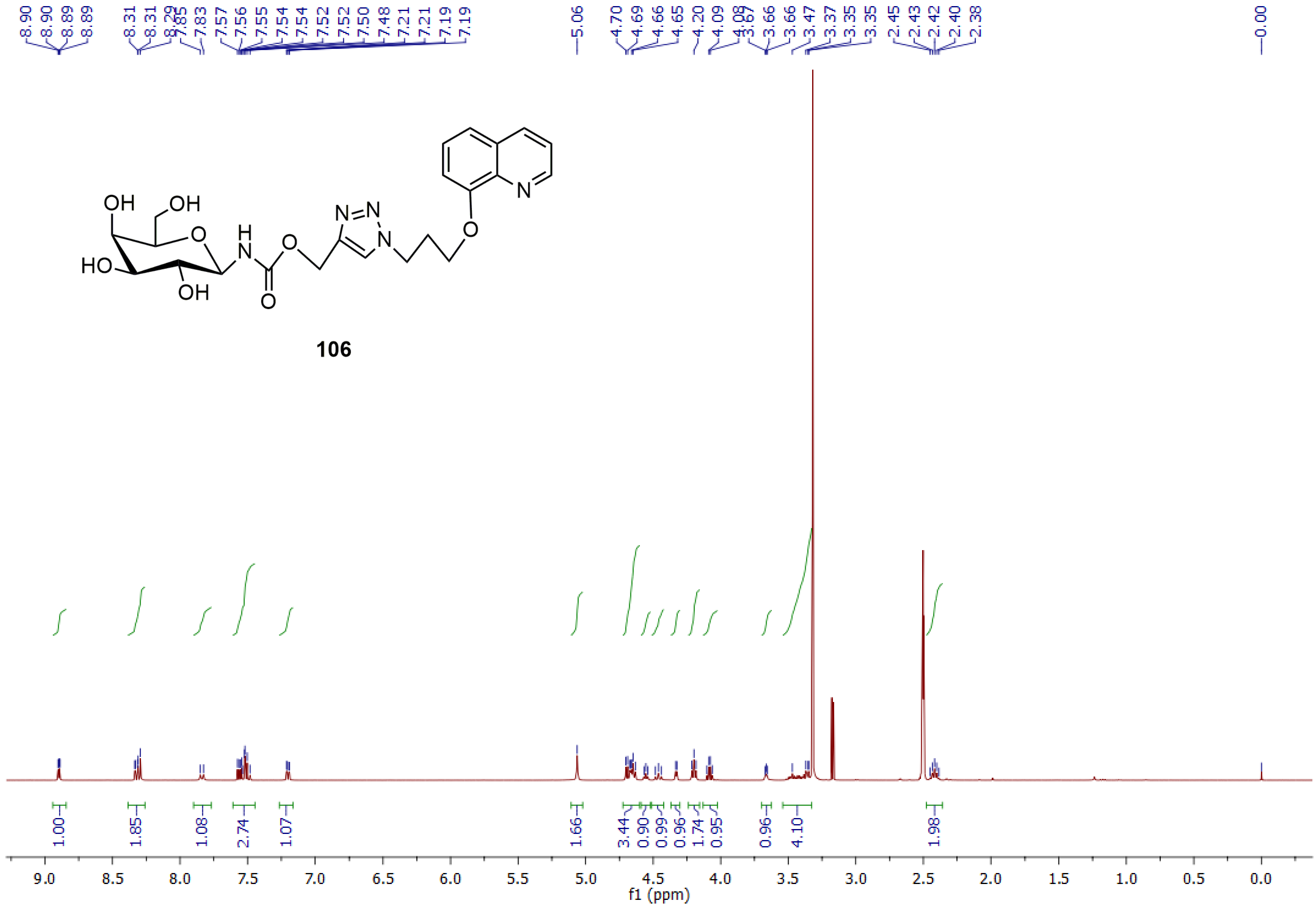

Fig. S203: ^{1}H NMR spectrum of glycoconjugate **106**.

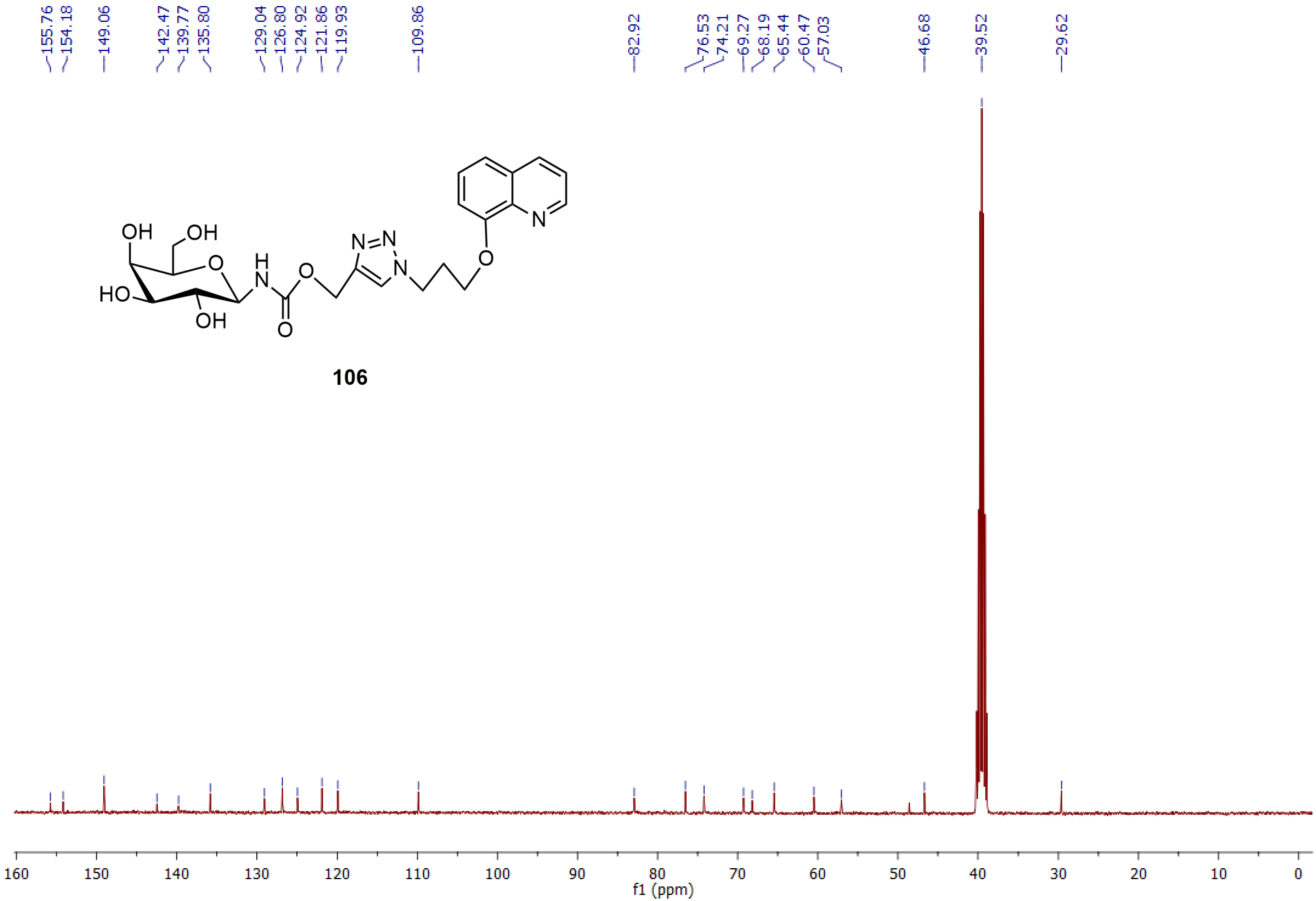

Fig. S204: ^{13}C NMR spectrum of glycoconjugate **106**.

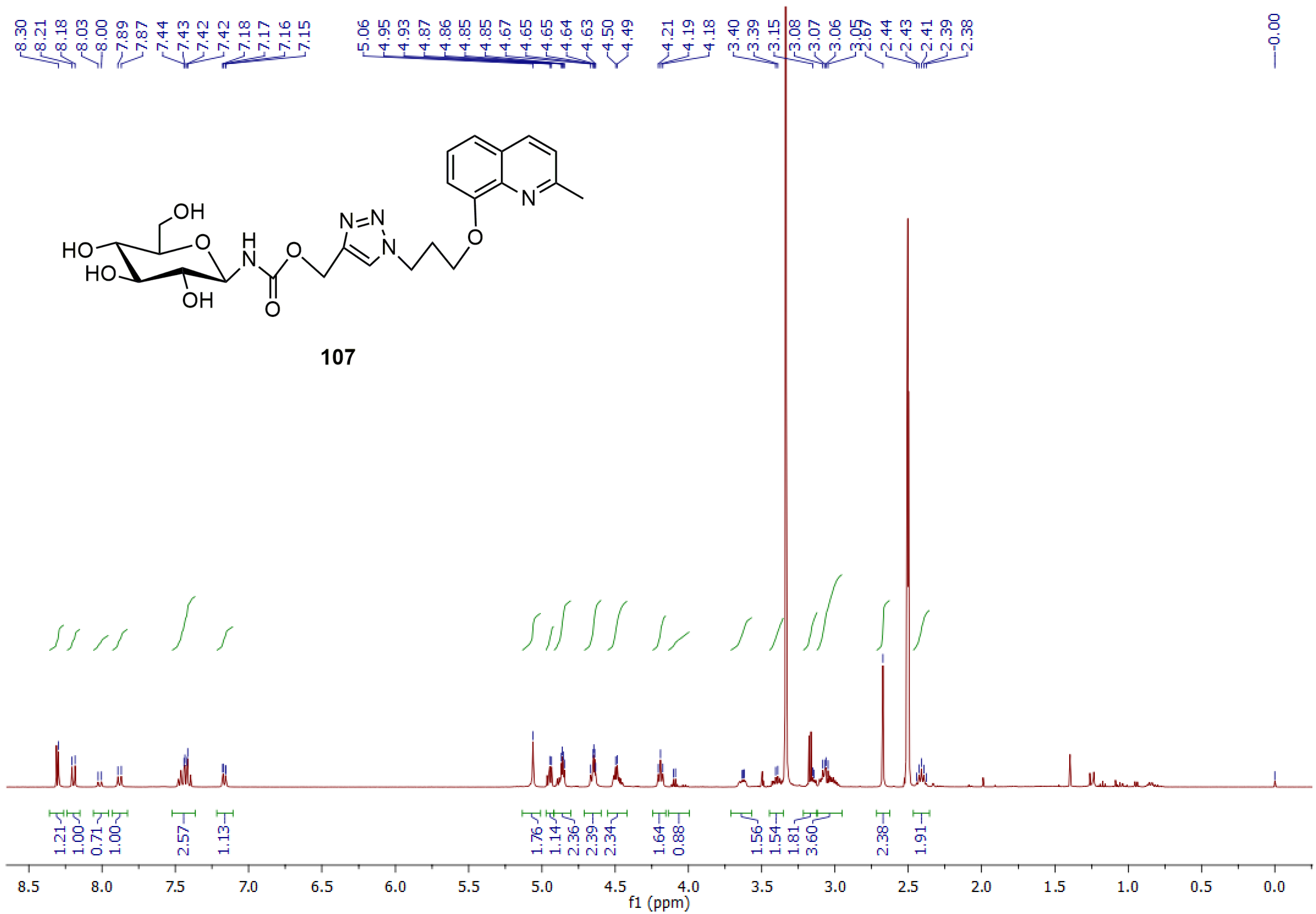

Fig. S205: ^{1}H NMR spectrum of glycoconjugate **107**.

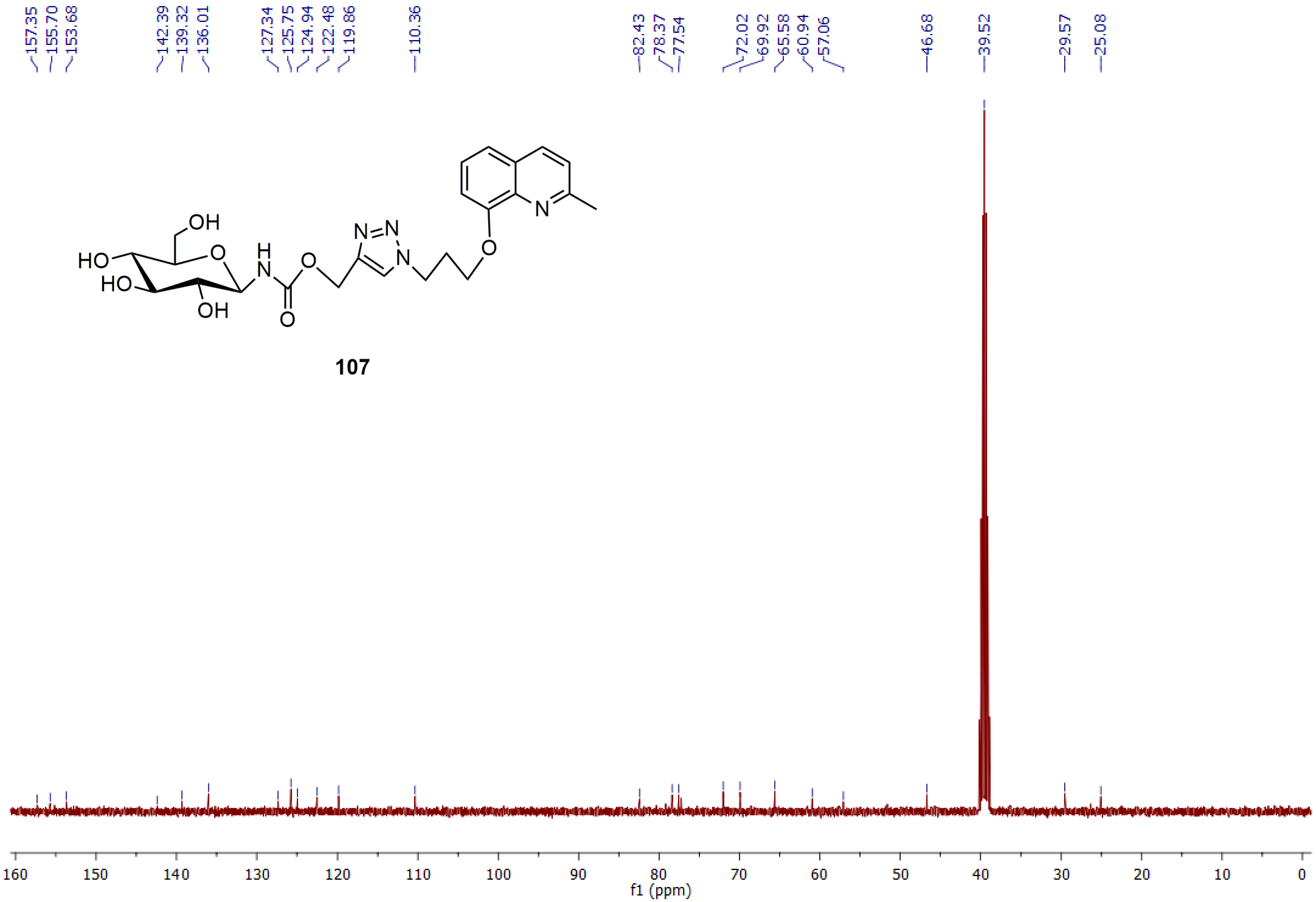

Fig. S206: ^{13}C NMR spectrum of glycoconjugate **107**.

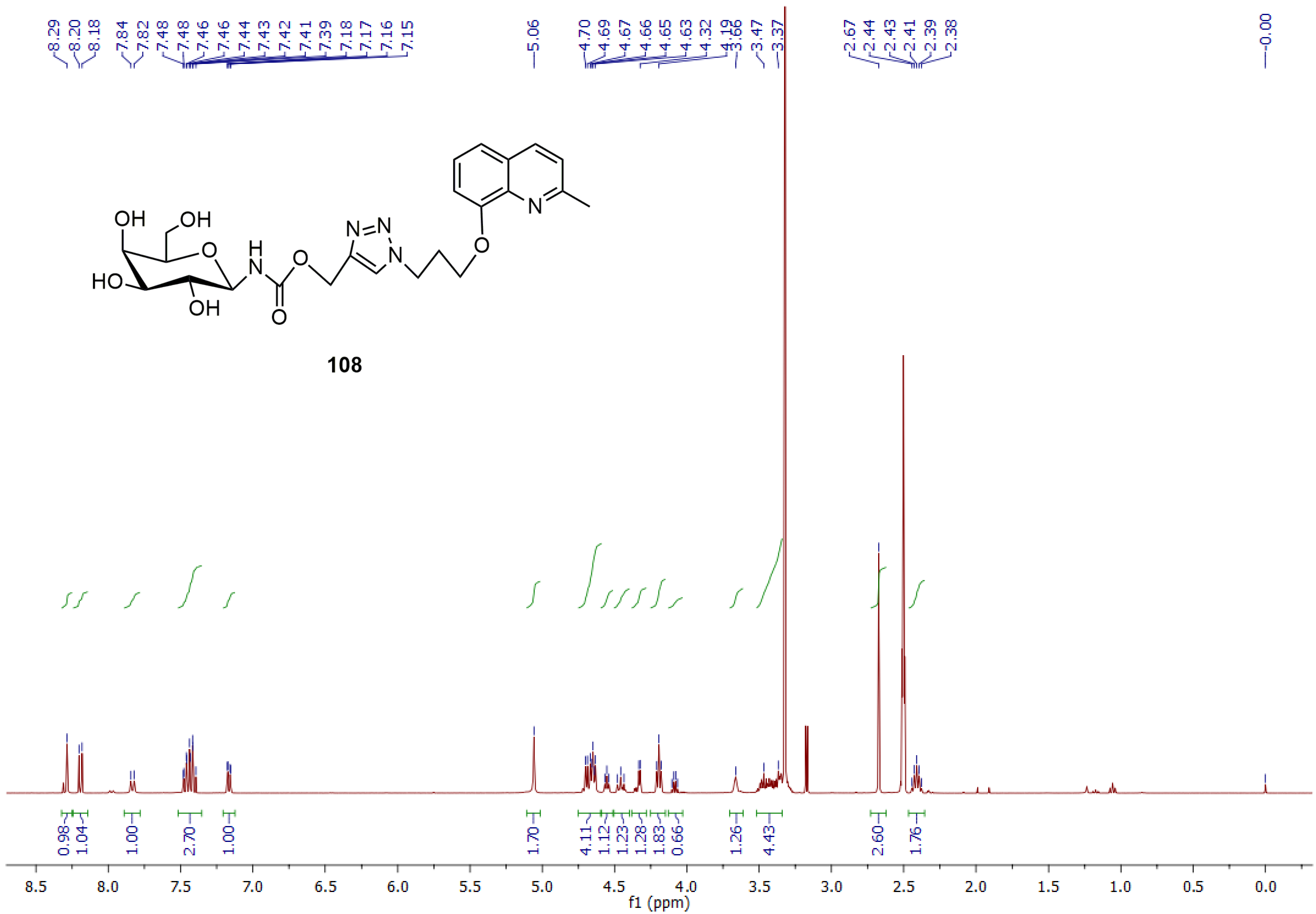

Fig. S207: ^{1}H NMR spectrum of glycoconjugate **108**.

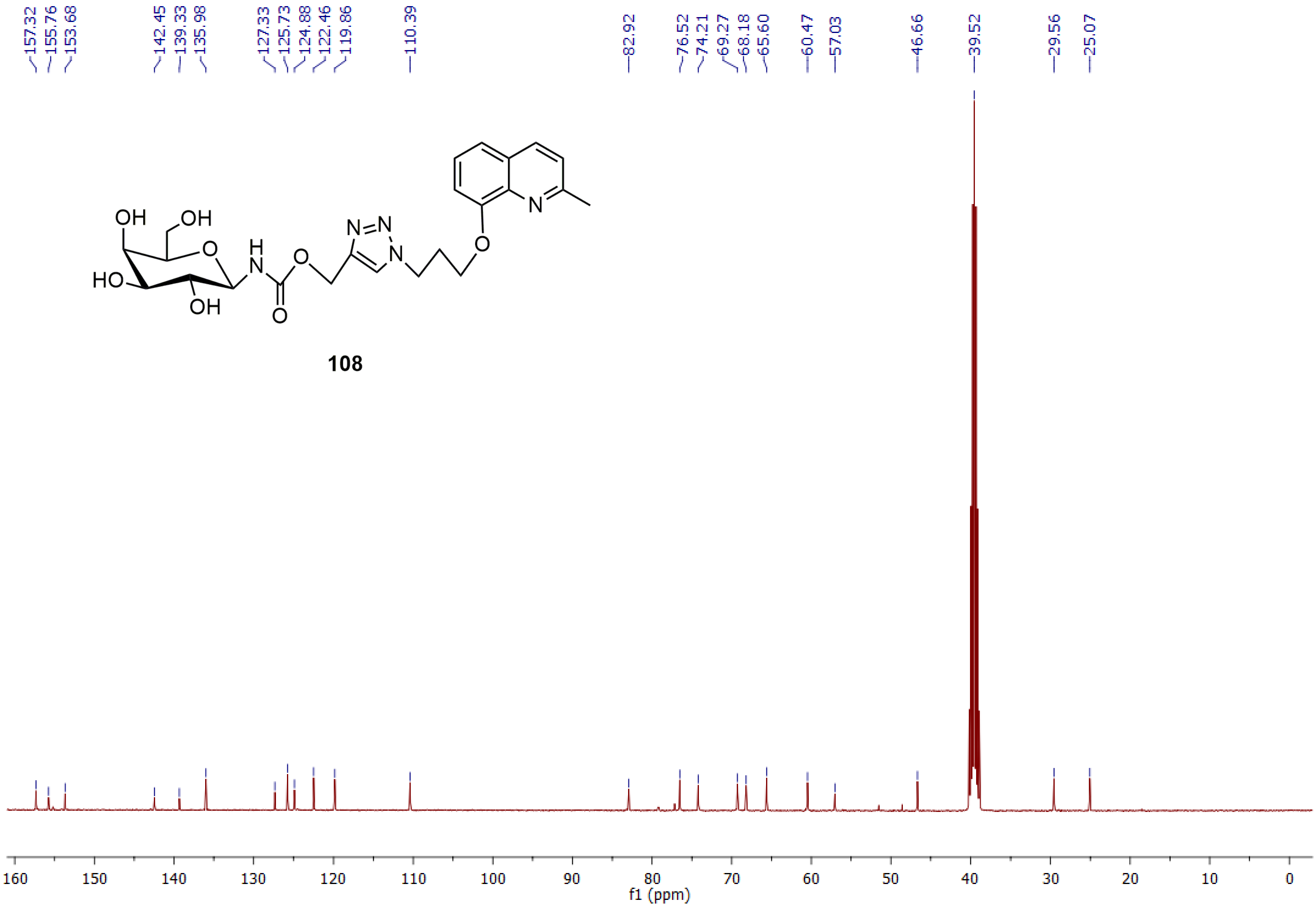

Fig. S208: ^{13}C NMR spectrum of glycoconjugate **108**.

2. Metal complexing properties of compound **93**

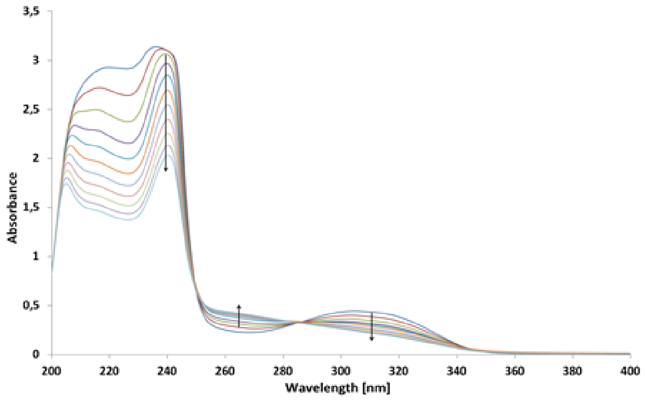

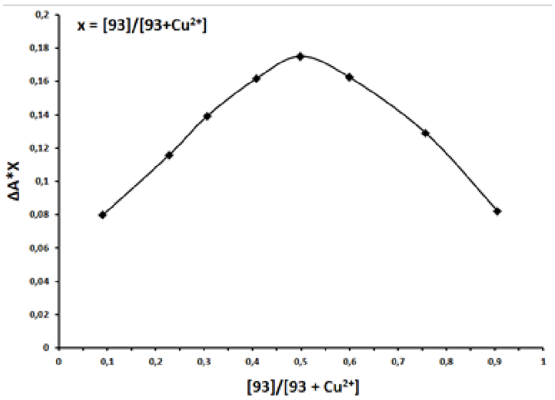

Figure S209: (a) UV spectrum of compound **93** with the addition of copper sulfate pentahydrate in methanol; **(b)** Job's plot of **93** with Cu^{2+}. The absorbance was monitored at 265 nm.

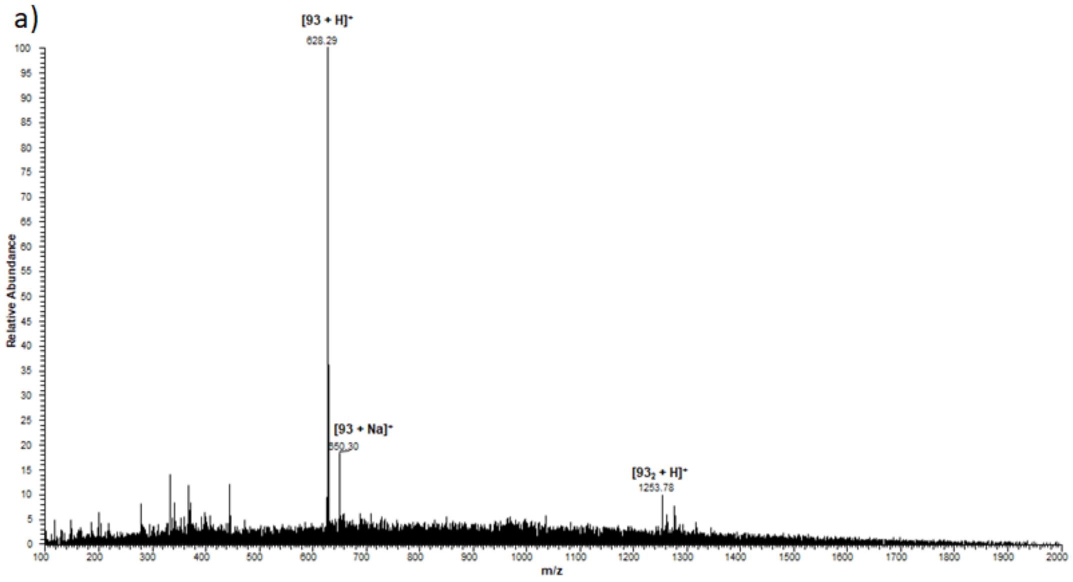

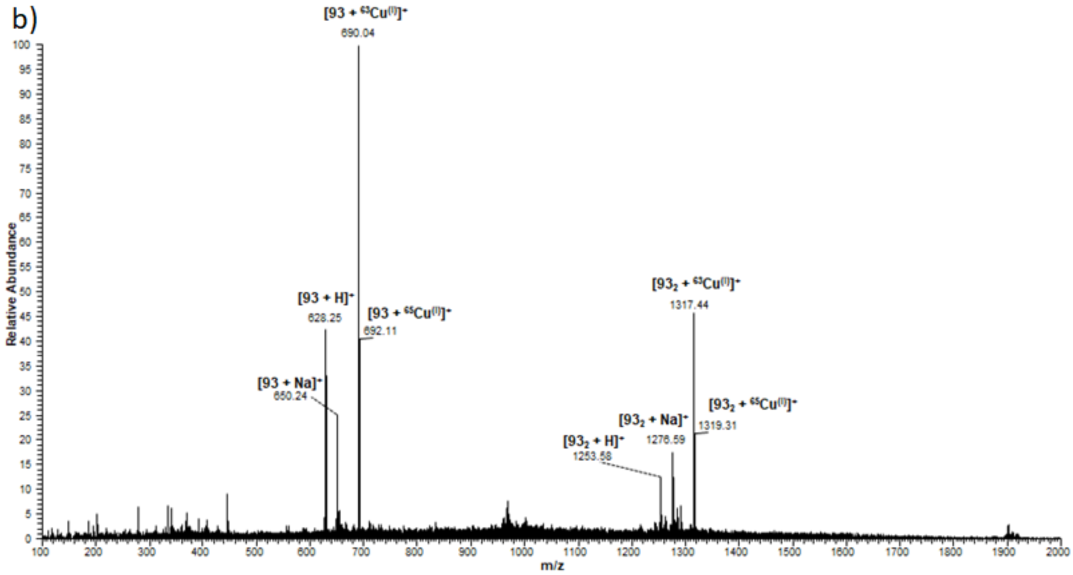

Figure S210: ESI-MS (positive-ion mode) spectrum of **(a) 93** compound and **(b) 93** after addition of Cu^{2+} ions into **93** solution.

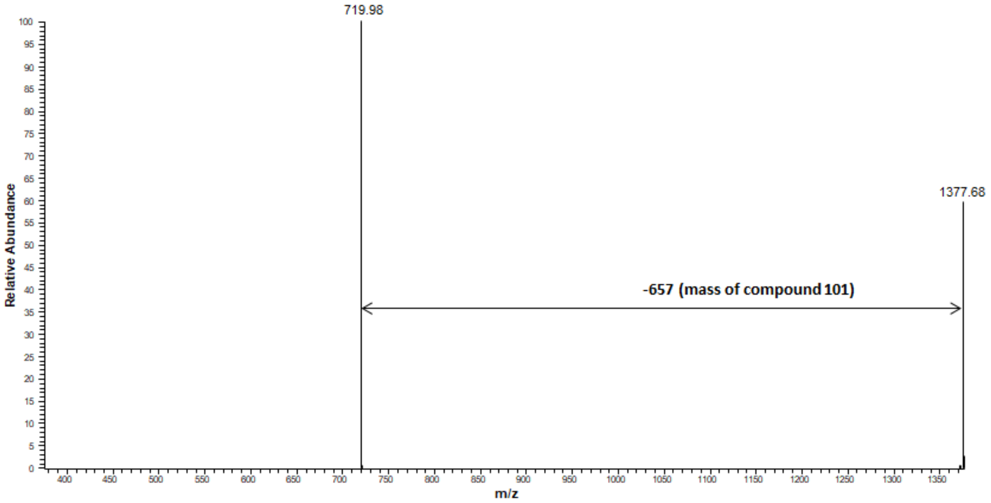

Figure S211: ESI-MS/MS fragmentation spectrum (positive-ion mode) of corresponding parent ion m/z = 1377.68.
